# Synthesis of novel 1,2,4-oxadiazole-isoxazoline hybrids and their in silico potential with adenosine receptors

**DOI:** 10.3762/bjoc.22.82

**Published:** 2026-07-06

**Authors:** Pshtiwan S Mohammed, Mohammed K S Dalo, Onur C Yazıcı, Muhammet Yildirim, Akın Sağırlı

**Affiliations:** 1 Department of Chemistry, College of Sciences, Charmo University, Peshawa Street, Chamchamal, Sulaymaniyah, 46023, Iraq; 2 Department of Pharmacy, Bright Technical and Vocational Institute, Sulaymaniyah, Kurdistan Region, 46001, Iraq; 3 Department of Chemistry, College of Sciences, Ibrahim Ahmed High School, Bardasur Street, Kalar, Sulaymaniyah, 46021, Iraq; 4 Department of Chemistry, Faculty of Arts and Sciences, Bolu Abant Izzet Baysal University, TR-14280, Bolu, Türkiye

**Keywords:** aldoxime, antiparkinson, 1,3-dipolar cycloaddition, isoxazoline, 1,2,4-oxadiazoles

## Abstract

A concise and efficient synthetic route to novel 1,2,4-oxadiazole-isoxazoline hybrids **7** has been developed via regioselective 1,3-dipolar cycloaddition of in situ-generated nitrile oxides with 3-(*p*-substituted-aryl)-5-vinyl-1,2,4-oxadiazoles **6**. The target compounds **7a–ay** were obtained in moderate to excellent yields (16–97%) and fully characterized by IR, NMR, and HRMS analyses. The reactions exhibited high regioselectivity, exclusively affording 5-isoxazoline derivatives, while substituent effects played a decisive role in modulating reaction efficiency. In silico studies revealed that all hybrids **7a–ay** display strong binding affinities toward the adenosine A₁ receptor (−10.0 to −8.3 kcal/mol), surpassing the co-crystallized ligand and engaging in key stabilizing interactions within the binding pocket. Furthermore, ADMET predictions indicated favorable drug-likeness, high gastrointestinal absorption, and suitable physicochemical properties. Overall, these findings identify 1,2,4-oxadiazole-isoxazoline hybrids as promising and tunable scaffolds for the development of adenosine A₁ receptor-targeted agents; however, further structural optimization and comprehensive biological evaluation are required to fully validate their therapeutic potential.

## Introduction

**Figure 1:**
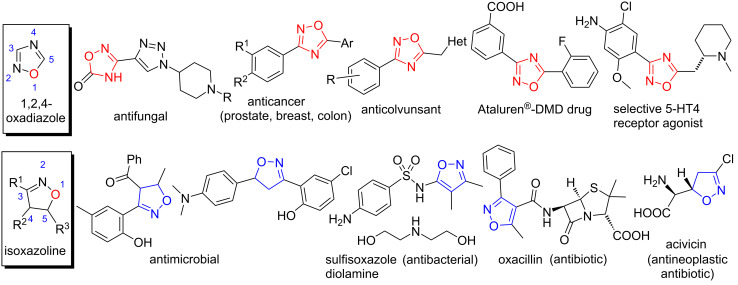
General structure of isoxazoline, 1,2,4-oxadiazole and biologically active 1,2,4-oxadizole, isoxazole and isoxazoline-based molecules or drugs.

1,2,4-Oxadiazoles are pharmacologically significant five-membered heterocyclic rings, primarily recognized for their bioisosteric relationship with ester and amide functional groups [1–5]. 1,2,4-Oxadiazole derivatives interact with various receptors as agonists or antagonists, demonstrating a wide spectrum of biological activities including anti-inflammatory, anticancer, antidepressant, anti-HIV, antifungal, and anticonvulsant properties [6–13]. A notable therapeutic application is found in Ataluren^®^, a drug for for the treatment of Duchenne Muscular Dystrophy (DMD), which features a 1,2,4-oxadiazole scaffold [14] (Figure 1).

In addition to oxadiazoles, numerous five-membered *O*,*N*-containing heterocycles have been developed as pharmacotherapeutic agents so far. Specifically, isoxazoline-based compounds have shown broad biological efficacy, including antimicrobial, anti-inflammatory, anticancer, and antidepressant effects [15–20] (Figure 1). Prominent drugs containing isoxazole or isoxazoline rings, such as sulfisoxazole, oxacillin, and acivicin, have been clinically utilized for many years [21–28] (Figure 1).

The synthesis of 1,2,4-oxadiazole derivatives can be achieved through various efficient methods, such as the cyclocondensation of amidoximes with carbonyl compounds, palladium-catalyzed reactions, anodic oxidations, transformations from other heterocycles or 1,3-dipolar cycloaddition [29–44].

For isoxazoline derivatives, the 1,3-dipolar cycloaddition (1,3-DC) of nitrile oxides serves as a fundamental synthetic tool. These reactions are generally governed by HOMO–LUMO orbital interactions between the dipole and dipolarophile. While substituent effects on the nitrile oxide dipole are minimal, monosubstituted alkenes typically yield 5-substituted isoxazolines with high regioselectivity due to steric and electronic factors. 5-Substituted isoxazolines (2-isoxazolines) have been synthesized in high to excellent yields (75–98%) through nitrile oxide cycloaddition reactions. These nitrile oxides are generated either from aldoximes using various oxidizing agents – such as NaOCl, *t*-BuOCl, NCS, oxone/NaCl, triflic acid–hypervalent iodine systems, oxone–silica, and chloramine-T – or from chloroaldoximes in the presence of bases including triethylamine, K_2_CO_3_, NaHCO_3_, and pyridine [45–54]. The combination of Et_3_N/NaOCl represents a particularly effective system for the in situ generation of nitrile oxides from aldoximes. In contrast to single-component oxidants such as NCS, oxone–NaCl, or chloramine-T, the Et_3_N/NaOCl system enables a stepwise and controlled formation of the reactive dipole via initial oxidation to the hydroxyimoyl chloride followed by rapid base-promoted dehydrohalogenation. This controlled release minimizes the accumulation of free nitrile oxide, thereby suppressing competitive furoxan dimerization, a common yield-limiting pathway in nitrile oxide cycloadditions. Moreover, the presence of triethylamine buffers the reaction medium and scavenges HCl, reducing over-oxidation and undesired chlorination that may occur with NCS or oxone-based systems. Compared with chloramine-T, which often requires elevated temperatures or longer reaction times, Et_3_N/NaOCl proceeds efficiently under milder conditions and displays improved tolerance toward sensitive or electron-rich dipolarophiles [54–57].

**Scheme 1:**
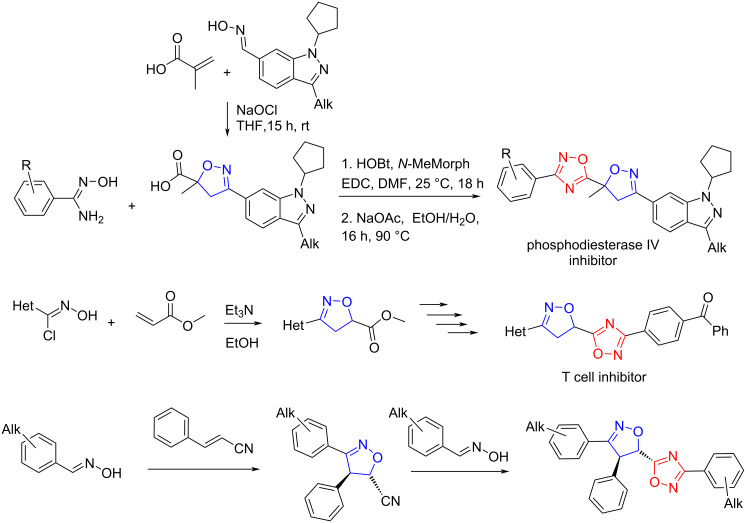
Recent studies for the synthesis of 1,2,4-oxadiazole-isoxazoline hybrids.

Despite the proven efficacy of both heterocyclic rings as pharmacophores, reports focusing on the synthesis of 1,2,4-oxadiazole-isoxazoline hybrids remain scarce. Previous studies have suggested that such hybrid structures may exhibit phosphodiesterase IV inhibitory activity or possess therapeutic potential against T-cell-mediated disorders, including rheumatoid arthritis and leukemia [58–60]. In this context, a series of 1,2,4-oxadiazole-indazolylisoxazoline derivatives were prepared as phosphodiesterase IV inhibitors via a multistep approach involving nitrile oxide cycloaddition of indazolyl nitrile oxides with methacrylic acid to afford indazolylisoxazoline esters, followed by cyclization with various aryl benzamidoximes [58]. In a related study, novel 1,2,4-oxadiazole-isoxazole hybrids were synthesized from isoxazoline esters generated through nitrile oxide cycloadditions of heterocyclic chlorooximes with methyl 2-propenoate [59]. Furthermore, sequential nitrile oxide cycloaddition reactions of benzonitrile and mesitonitrile oxides with cinnamonitrile were reported as an efficient route to structurally diverse 1,2,4-oxadiazole-isoxazole hybrids [60] (Scheme 1).

On the other hand, adenosine receptor agonists and antagonists exert diverse physiological effects depending on the targeted subtype (A_1_, A_2_A, A_2_B, A_3_). For instance, adenosine antagonists are commonly used as stimulants like caffeine, and in neurodegenerative diseases, asthma, and immunotherapy [61–63]. Adenosine receptors are key players in diverse physiological systems. The A_1_ and A_2_A subtypes are vital for managing heart function – specifically myocardial oxygen demand and coronary blood flow – and A_2_A receptors additionally play a crucial role in reducing systemic inflammation [62]. Beyond these peripheral roles, both receptors are key regulators of neurotransmitter activity, particularly concerning dopamine and glutamate levels in the brain. [63–66]. In contrast, the A_2_B and A_3_ receptors are mainly found in peripheral tissues, where they are central to immune and inflammatory processes.

**Figure 2:**
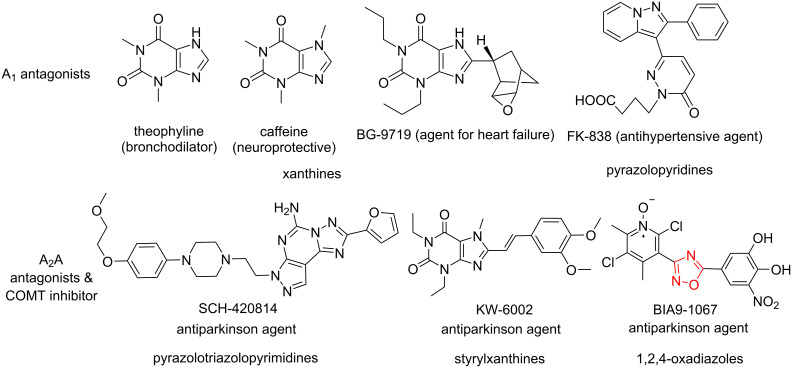
Various adenosine receptor (AR) antagonists, catechol-*O*-methyltransferase (COMT) and 1,2,4-oxadiazole type inhibitors in clinical trials.

Technological progress has enabled the creation of highly potent and selective adenosine receptor agonists and antagonists. These refined tools allow researchers to isolate the effects of specific receptor subtypes, providing the foundation for next-generation, targeted therapies. While many of these agents rely on traditional adenosine or xanthine foundations, the discovery of structurally diverse ligands – such as FK-838 and SCH-420814 – has significantly broadened the landscape for future drug development (Figure 2) [67–71]. Although certain 1,2,4-oxadiazole analogs (BIA9-1067) have been explored as A_2_A antagonists for neurodegenerative diseases like Parkinson’s, there are currently no reported studies on isoxazoline derivatives functioning as adenosine receptor modulators (Figure 2) [72–74]. Consequently, identifying new lead compounds with hybrid structures presents a significant opportunity for drug development.

**Scheme 2:**
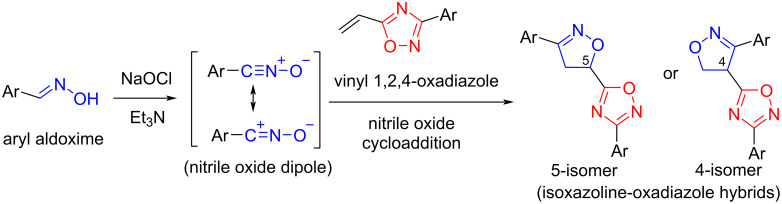
Synthesis of novel 1,2,4-oxadiazole-isoxazoline lead compounds by 1,3-DC.

In this study, we aimed to efficiently prepare novel 1,2,4-oxadiazole-isoxazole hybrids and evaluate their potential as adenosine receptor inhibitors through in silico analysis. Utilizing 1,3-dipolar cycloaddition reactions – a method previously established in our research for constructing diverse heterocycles – we synthesized target 3,5-diaryl-substituted isoxazoline-1,2,4-oxadiazole hybrids (4- and 5-isomer, Scheme 2) [75–79]. These hybrids were generated regioselectively via the cycloaddition of nitrile oxides derived from aryl aldoximes with aryl vinyl-1,2,4-oxadiazole derivatives in the presence of NaOCl/Et_3_N (Scheme 2). Finally, the binding affinities of these novel molecules were investigated in silico specifically regarding the A_1_ adenosine receptor.

## Results and Discussion

### Chemistry

**Scheme 3:**
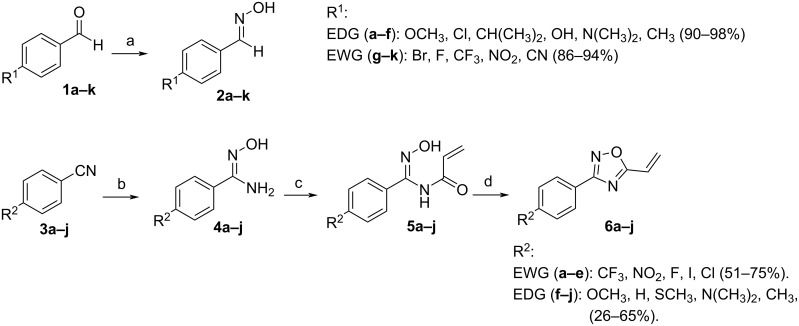
Synthetic methods for accessing the starting compounds (**2a–k**, **6a–j**). Reagents and conditions: a) NH_2_OH·HCl, NaOH, EtOH–H_2_O (5:1), 50–55 °C, 30 min; b) NH_2_OH·HCl, EtOH, Et_3_N, 80 °C, 24 h; c) acryloyl chloride, CH_2_Cl_2_, rt; d) K_2_CO_3_, 1,4-dioxane, 120 °C, 24 h.

Firstly, a series of electron-rich and electron-poor benzaldoximes **2a–k** were synthesized in excellent yields (86–98%) via the reaction of 4-substituted benzaldehydes **1a–k** with hydroxylamine under basic conditions. Secondly, a three-step synthetic sequence was employed, involving the conversion of benzonitriles **3a–j** to benzamidoximes **4a–j**, subsequent transformation into the corresponding acrylamides **5a–j**, and final cyclization to afford 3-(*p*-subsituted-aryl)-5-vinyl-1,2,4-oxadiazoles **6a–j** bearing both electron-donating and electron-withdrawing groups. These compounds were obtained in moderate to good overall yields (51–75%), with the exception of **6i** (Scheme 3, Table 1). The low yield of **6i** is likely attributed to the amidoxime formation step, where the electron-rich dimethylamino group in benzonitrile **3i** did not sufficiently facilitate amidoxime formation. Moreover, the final cyclization step, conducted under basic conditions at elevated temperature, may also contribute to reduced yields for compounds **6a–j**.

**Table 1:** Precursor compounds (**2a–k**, **6a–j**) and their yields.

*p*-Substituted benzaldoximes **2a–k**				3-Aryl-5-vinyl-1,2,4-oxadiazoles **6a–j**			

Entry	Product	R^1^	Yield (%)	Entry	Product	R^2^	Yield (%)

1	**2a**	OCH_3_	94	12	**6a**	CF_3_	54
2	**2b**	Cl	92	13	**6b**	NO_2_	59
3	**2c**	CH(CH_3_)_2_	90^a^	14	**6c**	F	66
4	**2d**	OH	98	15	**6d**	I	51
5	**2e**	N(CH_3_)_2_	90	16	**6e**	Cl	75
6	**2f**	CH_3_	94	17	**6f**	OCH_3_	58
7	**2g**	Br	92	18	**6g**	H	65
8	**2h**	F	86	19	**6h**	SCH_3_	60
9	**2i**	CF_3_	90	20	**6i**	N(CH_3_)_2_	26
10	**2j**	NO_2_	94	21	**6j**	CH_3_	52
11	**2k**	CN	88				

^a^Semi-solid.

The structures of benzaldoximes **2a–k** were simply characterized by IR stretching peaks corresponding to O–H, C=N, aromatic C=C, aromatic C–H, oxime C–H, and N–O groups (approximately 3250, 1600, 1500, 3040, and 1005 cm^−1^) (see Experimental section). Similarly, the structures of *p*-substituted phenyl vinyl 1,2,4-oxadiazoles **6a–j** were established primarily through IR absorptions attributed to C=N, C–O, and N–O stretching vibrations (approximately 1646, 1250, and 830 cm^−1^). Structural confirmation was further supported by the observation of vinylic proton signals at around 6.5, 5.9, and 6.7 ppm and aromatic proton resonances in the range of 7.0–8.5 ppm in the ¹H NMR spectra, as well as characteristic oxadiazole ring C=N carbon signals at approximately 168 and 175 ppm in the ¹³C NMR spectra. Furthermore, it was found that the obtained structural data for benzaldoximes (**2a–k**) and aryl vinyl 1,2,4-oxadiazoles (**6a–j**) are consistent with those reported in the literature [80–91].

Following the characterization of the precursors (**2a–k** and **6a–j**), we proceeded to the synthesis of 1,2,4-oxadiazole-isoxazoline hybrids (**7**) via 1,3-dipolar cycloaddition reactions of nitrile oxides generated in situ. To this end, a model reaction was designed using *p*-(dimethylamino)benzaldoxime (**2e**) and 3-(*p*-iodophenyl)-5-vinyl-1,2,4-oxadiazole (**6d**) in the presence of sodium hypochlorite and triethylamine to determine the optimal reaction conditions. A series of experiments was carried out by varying the equivalents of **2e** (1.2–1.5 equiv), **6d** (1.0–1.5 equiv), sodium hypochlorite (1.6–20 equiv), and triethylamine (1.2–2.4 equiv) (Table 2). In addition, control reactions employing either triethylamine or sodium hypochlorite alone at different concentrations were examined. With the exception of a single trial (entry 3, Table 2), the desired cycloaddition product **7a** was not obtained. Notably, when the equivalents of **2e**, **6d**, and triethylamine were kept constant and the amount of sodium hypochlorite was increased from 1.6 to 15 equivalents, a gradual improvement in the yield of **7a** was observed (entries 6–10, Table 2). However, further increases in the equivalents of **6d**, sodium hypochlorite, or triethylamine, as well as extended reaction times, did not lead to any additional enhancement in yield (entries 11 and 12, Table 2). As a result of these optimization studies, the target compound **7a** was obtained in moderate yield under the optimized conditions using 1.0 equivalent of **2e**, 1.5 equivalents of **6d**, 15 equivalents of sodium hypochlorite, and 1.0 equivalent of triethylamine in dichloromethane at room temperature (entry 10, Table 2).

**Table 2:** Reaction optimizations for the synthesis of **7a**.

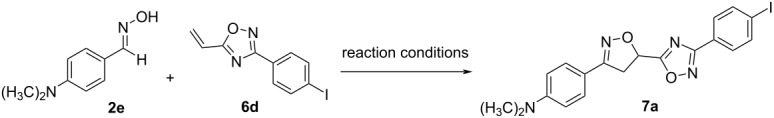							

Entry	**2e** (equiv)	**6d** (equiv)	NaOCl (equiv)	Et_3_N (equiv)	DCM (mL)	Time (h)	**7a** Yield (%)

1	1.2	1.0	1.6	–	10	8	–
2	1.2	1.0	3.2	–	10	16	–^a^
3	1.5	1.2	10	–	10	16	12
4	1.2	1.0	–	1.2	5	8	–
5	1.2	1.0	–	2.4	5	8	–
6	1.2	1.0	1.6	1.0	5	6	–
7	1.2	1.0	1.6	1.0	10	24	–
8	1.2	1.0	3.2	1.0	10	8	15
9	1.2	1.0	10	1.0	15	8	28
10	1.5	1.0	15	1.0	15	10	40
11	1.5	1.2	15	1.2	15	12	35
12	1.5	1.5	20	1.5	15	12	40^b^

^a^Trace amount of product. ^b^Some of **6d** remained unreacted.

Initially, electron-rich benzaldoximes **2a–f** were reacted with electron-poor vinyl-1,2,4-oxadiazoles **6a–e** under the optimized reaction conditions. The targeted 1,2,4-oxadiazolyl diarylisoxazoline derivatives **7a–x** were obtained within 7–16 h in moderate to good yields (32–96%) after purification by flash column chromatography using ethyl acetate/hexanes. Evaluation of the nitrile oxide cycloaddition results revealed that reactions involving *p*-chlorobenzaldoxime (**2b**), *p*-isopropylbenzaldoxime (**2c**), and *p*-tolylaldoxime (**2f**) proceeded with moderate to high efficiency, affording the corresponding products in 32–96% yields (Table 3). Notably, the highest yield (96%) was achieved in the cycloaddition of *p*-tolylaldoxime (**2f**) with 3-(*p*-fluorophenyl)-5-vinyl-1,2,4-oxadiazole (**6c**) (entry 15, Table 3). In contrast, cycloadditions of *p*-methoxybenzaldoxime (**2a**) with vinyl-1,2,4-oxadiazoles **6** displayed only moderate efficiency, providing yields of 44–60% (entries 3, 8, 13, 17, and 22, Table 3). All reactions involving *N,N*-dimethylaminobenzaldoxime (**2e**) and vinyl-1,2,4-oxadiazoles **6a–d** predominantly afforded lower yields (32–40%) (entries 1, 7, 11, and 20, Table 3). Moreover, the cycloaddition of **2e** with 3-(*p*-chlorophenyl)-5-vinyl-1,2,4-oxadiazole (**6e**) failed to yield any detectable product (entry 25, Table 3). Similarly, no cycloaddition products were observed in reactions of *p*-hydroxybenzaldoxime (**2d**) with any of the vinyl-1,2,4-oxadiazoles **6**. The low yields or complete failure to obtain cycloaddition products in reactions involving *N,N*-dimethylaminobenzaldoxime (**2e**) and *p*-hydroxybenzaldoxime (**2d**) can be ascribed to side reactions induced by excess sodium hypochlorite in the aqueous medium, leading to the formation of chlorinated amine salts or chlorophenol byproducts, respectively. These competing processes are likely to suppress or prevent the in situ generation of nitrile oxide dipoles, thereby hindering efficient cycloaddition. [92–93].

**Table 3:** Reactions of electron-rich aldoximes with electron-poor vinyl-1,2,4-oxadiazoles and yields for 1,2,4-oxadiazoyl-diarylisoxazolines **7a–x**.

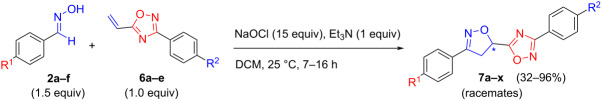					

Entry	Product	R^1^ (EDG)	R^2^ (EWG)	Time (h)	Yield (%)^*^

1	**7a**	N(CH_3_)_2_	I	10	40^a^
2	**7b**	Cl	I	10	56
3	**7c**	OCH_3_	I	9	44
4	**7d**	CH(CH_3_)_2_	I	10	38
5	**7e**	CH_3_	I	14	40
6	**7f**	Cl	NO_2_	10	51
7	**7g**	N(CH_3_)_2_	NO_2_	9	**32**
8	**7h**	OCH_3_	NO_2_	9.5	49
9	**7i**	CH(CH_3_)_2_	NO_2_	9	68
10	**7j**	CH_3_	NO_2_	14	86
11	**7k**	N(CH_3_)_2_	F	8	37^b^
12	**7l**	Cl	F	9	58
13	**7m**	OCH_3_	F	9	60
14	**7n**	CH(CH_3_)_2_	F	7	77
15	**7o**	CH_3_	F	16	**96**
16	**7p**	Cl	Cl	7.5	56
17	**7q**	OCH_3_	Cl	9.5	52
18	**7r**	CH(CH_3_)_2_	Cl	8	74
19	**7s**	CH_3_	Cl	15	66
20	**7t**	N(CH_3_)_2_	CF_3_	8	38^a^
21	**7u**	Cl	CF_3_	9	61
22	**7v**	OCH_3_	CF_3_	10	48
23	**7w**	CH(CH_3_)_2_	CF_3_	7	44
24	**7x**	CH_3_	CF_3_	16	57
25	**7y**	N(CH_3_)_2_	Cl	16	–^c^

^*^Yield after CC; ^a^semi-solid ; ^b^liquid; ^c^no product.

Secondly, electron-deficient benzaldoximes **2g–k** were subjected to cycloaddition with electron-rich vinyl-1,2,4-oxadiazoles **6f–j** under the optimized conditions for reaction times ranging from 5 to 24 h. The corresponding 1,2,4-oxadiazolyl-diarylisoxazoline derivatives **7aa–ay** were isolated in moderate to good yields after purification by flash column chromatography. The highest yield (97%) was obtained from the cycloaddition of *p*-bromobenzaldoxime (**2g**) with 3-(*p*-(dimethylamino)phenyl)-5-vinyl-1,2,4-oxadiazole (**6i**) (entry 16, Table 4). Notably, all cycloaddition reactions involving *p*-bromobenzaldoxime afforded at least moderate to good yields (50–68%). In general, nitrile oxide cycloadditions with 3-(*p*-(dimethylamino)phenyl)-5-vinyl-1,2,4-oxadiazole (**6i**) proceeded efficiently, delivering good to high yields across the examined substrates (Table 4, entries 16–20). In addition, reactions of *p*-trifluoromethylbenzaldoxime (**2i**) with vinyl-1,2,4-oxadiazoles **6** provided the desired products in moderate to good yields (52–78%) (Table 4). Cycloadditions involving *p*-fluorobenzaldoxime (**2h**) exhibited a broader range of efficiencies, with yields varying from low to high (35–91%) (Table 4). By contrast, cycloadditions of strongly electron-withdrawing *p*-nitrobenzaldoxime (**2j**) and *p*-cyanobenzaldoxime (**2k**) with the vinyl-1,2,4-oxadiazoles **6** generally did not exceed moderate yields (16–57%) (Table 4). For example, reactions of *p*-NO_2_- and *p*-CN-benzaldoximes (**2j** and **2k**) with 3-(*p*-(thiomethyl)phenyl)-5-vinyl-1,2,4-oxadiazole (**6h**) afforded the corresponding products **7an** and **7ao** in very low yields (16% and 22%, respectively; entries 14 and 15, Table 4). These reduced yields may be attributed to the strong electron-withdrawing *p*-cyano and *p*-nitro substituents, which decrease the nucleophilicity of the corresponding nitrile oxide dipoles through resonance effects, thereby diminishing cycloaddition efficiency. Prolonged reaction times of up to 24 hours may also contribute to the observed low yields.

**Table 4:** Reactions of electron-poor aldoximes with electron-rich vinyl-1,2,4-oxadiazoles and yields for 1,2,4-oxadiazoyl-diarylisoxazolines **7aa-ay**.

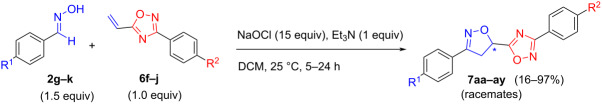					

Entry	Product	R^1^ (EWG)	R^2^ (EDG)	Time (h)	Yield (%)^*^

1	**7aa**	Br	OCH_3_	7	50
2	**7ab**	F	OCH_3_	7	35
3	**7ac**	CF_3_	OCH_3_	6	61
4	**7ad**	NO_2_	OCH_3_	24	49
5	**7ae**	CN	OCH_3_	24	55
6	**7af**	Br	H	5	54
7	**7ag**	F	H	5	71
8	**7ah**	CF_3_	H	5	78
9	**7ai**	NO_2_	H	24	42
10	**7aj**	CN	H	24	44
11	**7ak**	Br	SCH_3_	5	62
12	**7al**	F	SCH_3_	6	45
13	**7am**	CF_3_	SCH_3_	5	52
14	**7an**	NO_2_	SCH_3_	24	**16**
15	**7ao**	CN	SCH_3_	24	**22**
16	**7ap**	Br	N(CH_3_)_2_	7	**97**
17	**7aq**	F	N(CH_3_)_2_	7	91
18	**7ar**	CF_3_	N(CH_3_)_2_	7	75
19	**7as**	NO_2_	N(CH_3_)_2_	24	57
20	**7at**	CN	N(CH_3_)_2_	24	56
21	**7au**	Br	CH_3_	5	68
22	**7av**	F	CH_3_	6	62
23	**7aw**	CF_3_	CH_3_	6	75
24	**7ax**	NO_2_	CH_3_	24	51
25	**7ay**	CN	CH_3_	24	54

^*^Yield after CC.

**Scheme 4:**
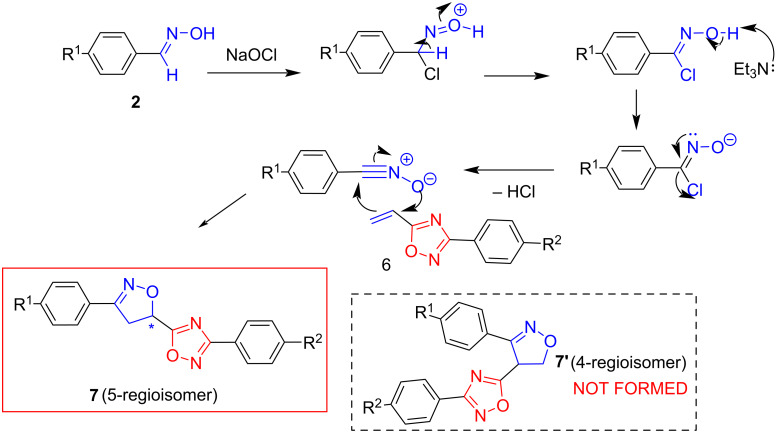
Mechanism for the formation of novel 3,5-disubstituted 1,2,4-oxadiazolyl diarylisoxazolines **7a–ay** (5-regioisomer).

The structures of all synthesized products **7a–ay** were fully determined using IR, NMR, mass spectrometry, and relevant physical data. In the IR spectra of compounds **7a–ay**, characteristic absorption bands were observed at approximately 3050, 2900, 1600, 1250, and 830 cm^−1^, corresponding to aromatic C–H, aliphatic C–H, C=N, C–O, and N–O stretching vibrations, respectively. In the ^1^H NMR spectra, the diastereotopic protons of the isoxazoline ring appeared as doublets of doublets (dd) at around 6.0, 4.0, and 3.9 ppm. The ^13^C NMR spectra further supported the proposed structures, displaying characteristic resonances for the 1,2,4-oxadiazole and isoxazoline ring carbons at approximately 175, 167, 156, 73, and 43 ppm. Analysis of the NMR data indicated that the cycloaddition products were formed predominantly as 5-regioisomers and isolated as racemic mixtures, demonstrating the high regioselectivity of the applied nitrile oxide cycloaddition reactions (Scheme 4). Moreover, the molecular masses of all compounds **7a–ay** were unambiguously confirmed by high-resolution mass spectrometry, which showed the expected M^+^ or [M + H]^+^ ions. (See Experimental section for physical, spectroscopic data and spectra of all products in Supporting Information File 1).

A plausible reaction mechanism for the formation of products **7** via 1,3-dipolar cycloaddition of nitrile oxides **2** with vinyl-1,2,4-oxadiazoles **6** is outlined in Scheme 4. In this process, the nitrile oxide dipole is generated in situ in the presence of sodium hypochlorite and subsequently undergoes rapid cycloaddition with vinyl-1,2,4-oxadiazole **6**. Although the dipolarophile can, in principle, react in two different orientations to afford two possible regioisomers, the present cycloaddition proceeds selectively to give only the 5-regioisomer as the observed product.

### In silico studies

#### Molecular docking studies with adenosine receptor

A molecular docking study was performed to obtain a comprehensive insight into the binding modes of the 1,2,4-oxadiazole-isoxazoline hybrids (**7a–ay**) and the co-crystallized reference ligand (1-butyl-3-(3-hydroxypropyl)-8-((2*R*,3a*S*,5*S*,6a*S*)-octahydro-2,5-methanopentalen-3a-yl)-1*H*-purine-2,6(3*H*,7*H*)-dione) within the adenosine A_1_ receptor. The A_1_ adenosine receptor is a key regulator of numerous physiological processes. Its activation (A_1_ agonism) is associated with reduced heart rate, neuroprotection, and relief of chronic and neuropathic pain, whereas its inhibition (A_1_ antagonism) leads to increased alertness and modulation of renal blood flow [63,65]. The predicted binding affinities of the **7a–ay** derivatives, evaluated in both possible enantiomeric forms (*R* and *S*), together with those of the co-crystallized reference ligand, against the A_1_ adenosine receptor, PDB ID 5N2S (https://doi.org/10.2210/pdb5N2S/pdb) [83], are summarized in Table 5 and Table 6.

**Table 5:** Binding affinities of 3,5-disubstituted 1,2,4-oxadiazolyl-diarylisoxazolines **7a–x** in two enantiomeric forms with human A_1_ adenosine receptor (PDB ID: 5N2S).

Product	Binding energy (kcal/mol)	Product	Binding energy (kcal/mol)

**7a**-(*R*)	−8,7	**7m**-(*R*)	−9,1
**7a**-(*S*)	−8,7	**7m**-(*S*)	−9,1
**7b**-(*R*)	−9	**7n**-(*R*)	**−9,5**
**7b**-(*S*)	−9	**7n**-(*S*)	**−9,5**
**7c**-(*R*)	−8,7	**7o**-(*R*)	−9,2
**7c**-(*S*)	−8,6	**7o-**(*S*)	**−9,8**
**7d**-(*R*)	−9,2	**7p**-(*R*)	−9,2
**7d-**(*S*)	**−9,5**	**7p**-(*S*)	−9,1
**7e**-(*R*)	−9,2	**7q**-(*R*)	−9
**7e**-(*S*)	−9,2	**7q**-(*S*)	−8,7
**7f**-(*R*)	−8,8	**7r**-(*R*)	−9,4
**7f**-(*S*)	−8,8	**7r**-(*S*)	−9,4
**7g**-(*R*)	−9,2	**7s**-(*R*)	−9,1
**7g**-(*S*)	−8,8	**7s-**(*S*)	**−9,7**
**7h**-(*R*)	−8,5	**7t-**(*R*)	**−9,6**
**7h**-(*S*)	−8,7	**7t**-(*S*)	−9,2
**7i**-(*R*)	−8,7	**7u-**(*R*)	**−9,7**
**7i**-(*S*)	−9,4	**7u**-(*S*)	**−9,5**
**7j**-(*R*)	−9,1	**7v**-(*R*)	−9,4
**7j-**(*S*)	**−9,9**	**7v**-(*S*)	−9,1
**7k**-(*R*)	−8,8	**7w-**(*R*)	**−9,8**
**7k**-(*S*)	−8,8	**7w-**(*S*)	**−9,6**
**7l**-(*R*)	−9,1	**7x-**(*R*)	**−9,8**
**7l**-(*S*)	−9,4	**7x-**(*S*)	**−10**
reference (co-cryst)	−8.3		

**Table 6:** Binding affinities of 3,5-disubstituted 1,2,4-oxadiazolyl-diarylisoxazolines **7aa–ay** in two enantiomeric forms with human A_1_ adenosine receptor (PDB ID: 5N2S).

Product	Binding energy (kcal/mol)	Product	Binding energy (kcal/mol)

**7aa**-(*R*)	−8,5	**7an**-(*R*)	−8,4
**7aa**-(*S*)	−8,7	**7an**-(*S*)	−8,3
**7ab**-(*R*)	−8,9	**7ao**-(*R*)	−8,8
**7ab**-(*S*)	−8,8	**7ao**-(*S*)	−9
**7ac**-(*R*)	−9,2	**7ap**-(*R*)	−8,7
**7ac**-(*S*)	−9,4	**7ap**-(*S*)	−8,8
**7ad**-(*R*)	−8,7	**7aq**-(*R*)	−8,9
**7ad**-(*S*)	−8,5	**7aq**-(*S*)	−8,9
**7ae**-(*R*)	−8,9	**7ar**-(*R*)	−9,4
**7ae**-(*S*)	−8,9	**7ar**-(*S*)	−9,2
**7af**-(*R*)	−8,8	**7as**-(*R*)	−8,5
**7af**-(*S*)	−8,9	**7as**-(*S*)	−8,6
**7ag**-(*R*)	−8,9	**7at**-(*R*)	−9
**7ag**-(*S*)	−9,2	**7at**-(*S*)	−9
**7ah**-(*R*)	−9,4	**7au**-(*R*)	−9,4
**7ah-**(*S*)	**−9,6**	**7au**-(*S*)	−9
**7ai**-(*R*)	−9	**7av**-(*R*)	−9,3
**7ai**-(*S*)	−8,8	**7av**-(*S*)	−9,2
**7aj**-(*R*)	−9,1	**7aw-**(*R*)	**−9,7**
**7aj**-(*S*)	−9,2	**7aw-**(*S*)	**−9,8**
**7ak**-(*R*)	−8,7	**7ax**-(*R*)	−8,9
**7ak**-(*S*)	−8,7	**7ax**-(*S*)	−9,4
**7al**-(*R*)	−8,7	**7ay**-(*R*)	−9,4
**7al**-(*S*)	−8,7	**7ay**-(*S*)	−9,4
**7am**-(*R*)	−9,4	reference (co-cryst)	−8.3
**7am**-(*S*)	−9,4		

**Figure 3:**
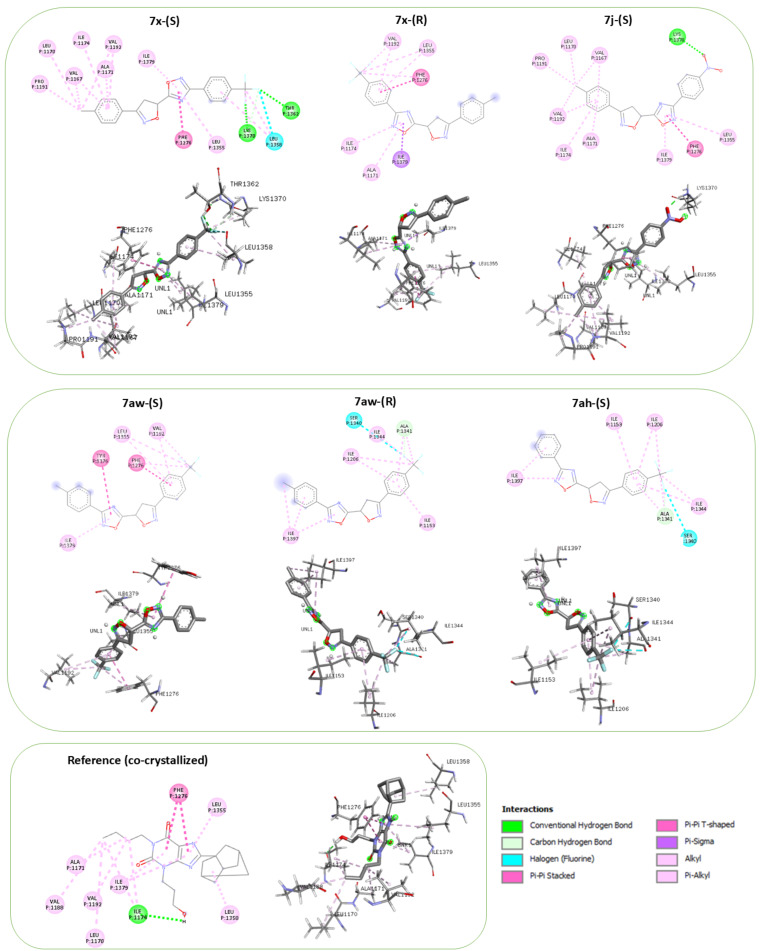
3D and 2D binding interactions of best scoring compounds (**7x**-(*S*), **7x**-(*R*), **7j**-(*S*)**, 7aw**-(*S*)**, 7aw**-(*R*)**, 7ah**-(*S*)) and reference (co-crystallized) ligand with A_1_ adenosine receptor. Green arrows: conventional hydrogen bonds; pink region: alkyl connections; light pink region: pi-alkyl connections; blue region: halogen interactions; light green region: carbon–hydrogen bond; dark-pink region: pi–pi stacked connections.

All compounds **7a–ay** exhibited high predicted binding affinities toward the adenosine A_1_ receptor, with binding energies ranging from −10.0 to −8.3 kcal/mol. Notably, the *S*-enantiomer of **7x** showed the strongest interaction with the receptor, displaying the highest binding energy (−10.0 kcal/mol). Other derivatives with comparably high affinities included the *S*-enantiomers of **7j** (−9.9 kcal/mol) and **7o** (−9.8 kcal/mol); the *R*-enantiomers of **7w** and **7x** (−9.8 kcal/mol) and **7t** (−9.6 kcal/mol); and the *S*-enantiomers of **7s** (−9.7 kcal/mol) and **7w** (−9.6 kcal/mol). In addition, both the *R*- and *S*-enantiomers of **7aw** (−9.8 and −9.7 kcal/mol, respectively), as well as the *S*-enantiomer of **7ah** (−9.6 kcal/mol), were predicted to bind strongly to the receptor. Overall, no substantial differences in binding affinity between the enantiomers of the same compounds were observed, with a few exceptions (**7d**, **7j**, **7o**, **7s**, **7t**, **7au**, and **7ax**). Furthermore, neither the *R*- nor the *S*-enantiomers showed a consistent preference or selectivity for receptor binding. The *S*- and *R*-enantiomers of **7x**, the *S*-enantiomers of **7j**, and the *S*-enantiomers of **7aw** shared similar binding modes, dominated by strong alkyl, π-alkyl, and π–π stacking interactions. Specifically, the *S*-enantiomers of **7x** and **7j** formed conventional hydrogen bonds with Lys1370, whereas the *R*-enantiomer of **7x** exhibited π–σ interactions with Ile1379, along with π–π stacking interactions between its oxadiazole ring and Phe1276. The *S*-enantiomers of **7aw** engaged in π–π interactions with Tyr1376, while the *R*-enantiomers of 7**aw** and the *S*-enantiomers of **7ah** formed C–H hydrogen bonding interactions with the Ala1341 residue. Additional alkyl and π-alkyl interactions were observed with Ile1174, Val1192, Val1167, Pro1191, and Leu1355 within hydrophobic regions of the binding pocket (Figure 3). Moreover, the *S*-enantiomer of **7aw** formed a halogen bond with Leu1358 and displayed π-alkyl interactions with Ala1171, Val1192, Ile1174, and Val1167. Its oxadiazole ring also participated in π–π stacking with Tyr1376 and alkyl interactions with Ile1379. Similarly, the CF_3_ group on the benzene ring of the *R*-enantiomer of **7aw** established a halogen bond with Ser1340, together with alkyl interactions involving Ile1153, Ile1206, and Ile1344, and π–σ interactions with Ile1397.

Moreover, the co-crystallized reference ligand exhibited a lower binding affinity (−8.3 kcal/mol) compared with all synthesized **7a–ay** derivatives. Its imidazolopyrimidine core was found to form conventional hydrogen bonds with the Ile1174 residue, along with π–π stacking interactions with Phe1276. In addition, several alkyl and π-alkyl interactions were observed with receptor residues Leu1379, Leu1355, Leu1358, Leu1170, Val1188, Val1192, and Ala1171.

Overall, these results demonstrate that four compounds (**7x**, **7j**, **7aw**, **7ah**) in both enantiomeric forms, exhibit strong binding affinities and favorable interaction profiles with the adenosine A₁ receptor. In particular, the highest binding energies observed for both the *R*- and *S*-enantiomers of **7x** suggest a more stable and robust interaction with the receptor compared to the other derivatives. Collectively, these in silico findings may contribute to drug discovery efforts by providing valuable insights for the design of more potent and selective inhibitors of the adenosine A_1_ receptor.

#### ADMET studies

An in silico ADMET analysis of the compounds with binding energy scores ≥ −9.5 kcal/mol (**7d**, **7j**, **7n**, **7o**, **7s**, **7t**, **7u**, **7w**, **7x**, **7ah**, and **7aw**) was performed using the SwissADME online tool [84] to evaluate their absorption, distribution, metabolism, excretion, and toxicity profiles. All selected derivatives complied with Lipinski’s Rule of Five, suggesting favorable drug-likeness and potential for good oral bioavailability. In addition, the compounds were predicted to exhibit high gastrointestinal (GI) absorption, indicating a strong likelihood of efficient uptake into systemic circulation following oral administration.

Lipophilicity, a key determinant of ADME behavior influencing membrane permeability and distribution, was also assessed. The predicted partition coefficients (iLOGP and XLOGP3) for all compounds were below the acceptable threshold of 5, supporting their suitability for oral drug development. Notably, only compound **7w** showed a slightly elevated XLOGP3 value (5.24), although its iLOGP value (3.90) remained within acceptable limits, suggesting an overall balanced lipophilicity profile (Table 7).

**Table 7:** Druggability predictions of high scoring compounds (>9.5 kcal/mol).

Product	MW (g/mol)	ILogP	XLogP3	GI absorption	Lipinski	Pains alert	Brenk alert

**7d**	459.28	3.92	5.00	high	yes	0	1: I
**7j**	350.33	2.80	3.42	high	yes	0	2: NO_2_, N-O
**7n**	351.37	3.76	4.46	high	yes	0	0
**7o**	323.32	3.36	3.69	high	yes	0	0
**7s**	339.78	3.46	4.22	high	yes	0	0
**7t**	402.37	3.55	4.24	high	yes	0	0
**7u**	393.75	3.44	4.74	high	yes	0	0
**7w**	401.38	3.90	5.24	high	yes	0	0
**7x**	373.33	3.46	4.48	high	yes	0	0
**7ah**	359.30	3.18	4.11	high	yes	0	0
**7aw**	373.33	3.49	4.48	high	yes	0	0

All compounds showed no PAINS alerts, indicating a low probability of assay interference and false-positive biological activity. Similarly, most compounds exhibited no BRENK alerts, which is favorable as these alerts typically indicate the presence of potentially toxic, reactive, or metabolically unstable structural motifs. However, two exceptions were observed: **7d** showed an iodine-related alert, while **7j** displayed alerts associated with nitro and oxygen–nitrogen functionalities. The nitro group, in particular, is known to be associated with potential mutagenic and toxic effects, and therefore may require further evaluation.

## Conclusion

The present study describes the regioselective synthesis of 49 novel 1,2,4-oxadiazole-isoxazoline derivatives **7a–ay** via efficient 1,3-dipolar nitrile oxide cycloaddition reactions with various arylvinyl-1,2,4-oxadiazoles. The use of the Et_3_N/NaOCl system proved particularly advantageous, as it enabled controlled in situ generation of the nitrile oxide dipole, suppressed undesired furoxan formation, and provided mild and chemoselective reaction conditions, ultimately contributing to generally good yields. From a biological perspective, molecular docking studies identified several derivatives (**7d**, **7j**, **7n**, **7o**, **7s**, **7t**, **7u**, **7w**, **7x**, 7**ah**, and **7aw**) exhibiting strong predicted binding affinities toward the adenosine A₁ receptor (A₁R), with docking scores ≥ −9.5 kcal/mol. In addition, in silico ADMET evaluations of these high-scoring compounds indicated generally favorable drug-like properties, including good oral bioavailability and acceptable physicochemical profiles, supporting their potential as orally active candidates. Overall, these computational findings suggest that the newly synthesized 1,2,4-oxadiazole-isoxazoline derivatives may serve as promising scaffolds for adenosine A₁ receptor modulation, with potential relevance to therapeutic applications such as cardiovascular regulation (e.g., heart rate control and contractility modulation) and central nervous system effects via neurotransmitter regulation. However, it is important to emphasize that these results are based on theoretical in silico predictions. While the synthetic methodology demonstrates efficiency and regioselectivity, and the computational results indicate promising bioactivity and drug-likeness, experimental validation remains essential. Future studies should therefore focus on in vitro and in vivo biological evaluation to confirm receptor activity, clarify agonist versus antagonist behavior, establish structure–activity relationships (SAR), and assess pharmacokinetic stability and toxicity profiles.

## Supporting Information

File 1 Experimental sections, general procedures, IR, NMR, HRMS data and spectra for precursors **2a–k, 6a–j** and all products **7a–ay**.

## Data Availability

Data generated and analyzed during this study is available from the corresponding author upon reasonable request.
