# Supplementary material for: Synthesis of novel 1,2,4-oxadiazole-isoxazoline hybrids and their in silico potential with adenosine receptors
Source: Beilstein J Org Chem. 2026 Jul 6;22:1033–47. doi: 10.3762/bjoc.22.82 (PMC13358917; doi:10.3762/bjoc.22.82)
Supplement: File 1 — Experimental sections, general procedures, IR, NMR, HRMS data and spectra for precursors 2a–k, 6a–j and all products 7a–ay. [file Beilstein_J_Org_Chem-22-1033-s001.pdf]

BEILSTEIN JOURNAL OF ORGANIC CHEMISTRY

Supporting Information

for

Synthesis of novel 1,2,4-oxadiazole-isoxazoline hybrids and their in silico potential with adenosine receptors

Pshtiwan S. Mohammed, Mohammed K. S. Dalo, Onur C. Yazıcı, Muhammet Yildirim and Akın Sağırlı

Beilstein J. Org. Chem. **2026,** *22,* 1033–1047. doi:10.3762/bjoc.22.82

Experimental sections, general procedures, IR, NMR, HRMS data and spectra for precursors 2a–k, 6a–j and all products 7a–ay

License and Terms: This is a supporting information file under the terms of the Creative Commons Attribution License (https://creativecommons.org/licenses/by/4.0). Please note that the reuse, redistribution and reproduction in particular requires that the author(s) and source are credited and that individual graphics may be subject to special legal provisions.
The license is subject to the *Beilstein Journal of Organic Chemistry* terms and conditions: (https://www.beilstein-journals.org/bjoc/terms)

Table of contents

Experimental section S3

References.. S26

Spectra (IR, NMR, HRMS) for the precursors (**2**,**6**) and products (**7**)................................. S27

Experimental section

General

All reagents were used as received from Merck, Sigma–Aldrich, and Isolab without further purification. Reaction progress was monitored using silica gel 60 F_{254} plates (0.2 mm, Merck), visualized under ultraviolet light and by staining with permanganate solution. All the products (**7**) were purified using silica gel FCC (Merck Silica Gel 60). Melting points were determined by using an Electrothermal (Gallen Kamp) melting point apparatus and are uncorrected. Infrared spectra (IR) were obtained on a Thermo Scientific Nicolet iS20 FT-IR spectrometer instrument. The ^{1}H and ^{13}C NMR spectra were obtained on Bruker DPX 400 spectrometers (400 MHz for proton and 100 MHz for carbon) at ambient temperature. Chemical shifts (δ) are given in ppm relative to tetramethylsilane as downfield, the solvent utilized were $CDCl_3$ or DMSO-d_6, and the coupling constants (J) are reported in hertz (Hz). High-resolution mass measurements (HRMS) were recorded on a Waters Lct Premier XE oa-TOF mass spectrometer.

General Procedure for the synthesis of p-substituted benzaldoximes (**2a-k**) [1,2]

$NH_2OH.HCl$ (1.2 equiv, 0.12 mole, 8.3 g) in EtOH: H_2O (5:1) was dissolved in a round bottom flask, and NaOH (1.2 equiv, 0.12 mol, 4.8 g) was added and the resulting mixture was stirred until complete dissolution, then 4-substituted benzaldehyde **1** (1 equiv, 0.1 mol) was added slowly to the mixture. The resulting mixture is heated at 50–55 °C for 30 minutes, then reaction progress was controlled by TLC. After completion of the reaction, the solvent was evaporated by a rotary evaporator and, the aqueous phase was extracted with DCM (2 × 30 mL) and dried over anhydrous sodium sulfate, filtered, and DCM was evaporated until dryness. Solid products (**2a–k**) were isolated in excellent yields and used in the next step without purification.

(*E*)-4-Methoxybenzaldehyde oxime (**2a**)

White solid. Yield: (14.20 g, 94%). m.p. 64-66 ºC. IR (ATR, cm^{-1}): 3297 (OH), 2938, 1607 (OCH_3), 1573, 1514 (C=N), 1457, 1250, 1167.

(*E*)-4-Chlorobenzaldehyde oxime (**2b**)

White solid. Yield: (12.9g, 92%). m.p. 110-112 ºC. IR (ATR, cm^{-1}): 3258 (OH), 2920, 1921 (C=N), 1493, 1316, 1216 (Cl), 628, 631, 511.

(*E*)-4-Isopropylbenzaldehyde oxime (**2c**)

Light brown semi-solid. Yield: (14.68 g, 90%). IR (ATR, cm^{-1}): 3252 (OH), 2927, 1609 (C=N), 1453, 1417, 1363 ($CH(CH_3)$), 1384, 1216.

(*E*)-4-Hydroxybenzaldehyde oxime (**2d**)

White solid. Yield: (13.47 g, 98%). m.p. 112-114 ºC. IR (ATR, cm^{-1}): 3360 (OH), 3063 (OH), 2996, 1607 (C=N), 1453, 1476, 1301, 1210.

(*E*)-4-(Dimethylamino)benzaldehyde oxime (**2e**)

Light brown solid. Yield: (13.5g, 90%). m.p. 72-74 ºC. IR (ATR, cm^{-1}): 3132 (OH), 2911, 1601 (C=N), 1522, 1427, 1351, 1301 (C-N), 1223, 1125.

(*E*)-4-Methylbenzaldehyde oxime (**2f**)

White solid. Yield: (12.67 g, 94%). m.p. 72-74 ºC. IR (ATR, cm^{-1}): 3185 (C-H), 3108, 2988 (OH), 2915, 1608 (C=N), 1513, 1435, 1308, 1208.

(*E*)-4-Bromobenzaldehyde oxime (**2g**)

White solid. Yield: 9.2g (92%). m.p. 115-117°C. IR (liquid film, cm^{-1}): ν = 3256 (O-H), 3051, 2995 (arom CH), 2917 (aliph CH), 1587 (C=N), 1488 (arom C=C), 1396, 1315, 1066, 1007, 952, 870, 817 (N-O), 681, 506 (Br), 449.

(*E*)-4-Fluorobenzaldehyde oxime (**2h**)

Beige solid. Yield: 12 g (86%). m.p. 89-91°C. IR (liquid film, cm^{-1}): 3256 (O-H), 3051, 3017 (arom CH), 2791 (aliph CH), 1605, 1595 (C=N), 1509 (arom C=C), 1322, 1295, 1228, 1155, 1094 (C-F), 1015, 956, 879, 823 (N-O), 578, 513, 471.

(*E*)-4-(Trifluoromethyl)benzaldehyde oxime (**2i**)

White solid. Yield: 8.5 g (90%). m.p. 103-105°C. IR (liquid film, cm^{-1}): ν = 3266 (O-H), 3056, 3009 (arom CH), 2955-2923 (aleph CH), 1617 (C=N), 1467 (arom C=C), 1412, 1319, 1156, 1107, 1064 (CF_3), 1011, 970, 938, 871, 833 (N-O), 766, 681, 592, 466.

(*E*)-4-Nitrobenzaldehyde oxime (**2j**)

Yellowish solid. Yield: 7.8 g (94%). m.p. 129-131°C. IR (liquid film, cm^{-1}): ν = 3258 (O-H), 3104, 3078, 3044, 3012 (arom CH), 2943, 2838, 2789 (aliph CH), 1603 (C=N), 1518 (NO_2), 1495, 1481 (arom C=C), 1405, 1343-1318 (NO_2), 1214, 1106, 1008, 942, 909, 842 (N-O), 747, 686, 534, 488, 455.

(E)-4-Cyanobenzaldehyde oxime (**2k**)

White solid. Yield: 6.5 g (88%). m.p. 171-173°C. IR (liquid film, cm^{-1}): v = 3224 (O-H), 3054, 3024 (arom CH), 2933 (aleph CH), 2227 (C≡N), 1605 (C=N), 1507 (arom C=C), 1472 (arom C=C), 1404, 1307, 1218, 969, 938, 875, 824 (N-O), 700, 550, 460.

General procedure for the synthesis of benzamidoximes (**4a–j**)

In a round bottom flask $NH_2OH.HCl$ (20.25 mmole, 1.41g) was dissolved in EtOH (75 ml) then Et_3N (4.5 ml) was added to the mixture, and the 4-substituted benzonitrile **3** (13.5 mmol) was added to the mixture slowly and stirred at 80 °C for 24 hours. The reaction progress was controlled by TLC (mobile phase 5:1 hexane/ethyl acetate), after the reaction was completed, EtOH was evaporated at 70 °C by rotary evaporator, and the desired product was extracted twice by ethyl acetate (2 ×3 0 ml), and dried with Na_2SO_4 and filtered and ethyl acetate was rotaevaporated under vacuum. Solid products (**4a–j**) were obtained in 90–95 % yield and used in next step without purification.

General procedure for the synthesis of acrylamides (**5a–j**)

In the round bottom flask, a solution of benzamidoxime **4** (2.09 mmole) in DCM (20 ml) acryloyl chloride (2.5 mmol) was added and the mixture was stirred for 3 hours at room temperature. Then the reaction was quenched by adding 2 ml of saturated $NaHCO_3$. The organic layer was separated and the aqueous layer was extracted with DCM twice (2 × 30 ml), the organic extracts were combined, dried over Na_2SO_4, filtered, and solid products (**5a–j**) were collected in 90–95 % yield.

General procedure for the synthesis of arylvinyl-1,2,4-oxadiazoles (**6a–j**) [3]

A mixture of acrylamide **5** from previous step (1.78 mmol), and K_2CO_3 (3.56 mmol) in dioxane (20 ml) was heated at 120 °C for 24 hours. The reaction progress was monitored by TLC and, upon completion, the reaction mixture was allowed to cool to room temperature and the solvent was rotaevaporated. The resulting reaction mixture was purified by silica gel column chromatography using EtOAc/hexane mixtures, arylvinyl-1,2,4-oxadiazoles **6a–j** were isolated in moderate to good yields.

3-(4-(Trifluoromethyl)phenyl)-5-vinyl-1,2,4-oxadiazole (**6a**)

Light yellow solid. Yield: (268.5 mg, 54%). M.p. 53-55 ºC. IR (ATR, cm^{-1}): 3083(=CH), 2927 (arom. CH), 1915, 1646 (C=N), 1620, 1536 (C=C), 1482, 1417, 1358, 1321 (C-O), 1164, 1101 (CF_3), 1064, 1016, 967, 8854 (C-O), 849, 790 (N-O), 779, 711, 704, 594. ^{1}H

NMR (400 MHz, $CDCl_3$) δ 8.22 (d, *J* = 8.2 Hz, 2H), 7.75 (d, *J* = 8.3 Hz, 2H), 6.77 (d, *J* = 6.7 Hz, 1H), 6.62 (d, *J* = 17.6 Hz, 1H), 6.03 (d, *J* = 11.0 Hz, 1H). ^{13}C NMR (101 MHz, $CDCl_3$) δ 175.0, 167.8, 132.9, 130.3, 129.4, 126.0, 125.9, 125.9, 120.4.

3-(4-Nitrophenyl)-5-vinyl-1,2,4-oxadiazole (**6b**)

Cream-colored crystalline solid. Yield: (268 mg, 59%). M.p. 110-113. ºC. IR (ATR, cm^{-1}): 3103(=CH), 3081 (arom. CH), 1924, 1643(C=N), 1607, 1543 (C=C), 1511 (NO_2), 1418, 1340 (NO_2) 1331, 1312, 1290 (C-O), 1104, 1017 (C-O), 977, 956, 888 (N-O), 866, 788, 762, 710. ^{1}H NMR (400 MHz, $CDCl_3$) δ 8.35 (d, *J* = 9.0 Hz, 2H), 8.30 (d, *J* = 9.0 Hz, 2H), 6.79 (dd, *J* = 17.7, 10.9 Hz, 1H), 6.65 (d, *J* = 17.5 Hz, 1H), 6.07 (d, *J* = 10.9 Hz, 1H). ^{13}C NMR (101 MHz, $CDCl_3$) δ 175.2, 167.1, 149.4, 132.6, 129.6, 128.4, 124.1, 120.1.

3-(4-Fluorophenyl)-5-vinyl-1,2,4-oxadiazole (**6c**)

White solid. Yield: (261 mg, 66%). M.p. 50-52 ºC. IR (ATR, cm^{-1}): 3062(=CH), 2924 (arom. CH), 1950, 1651 (C=N), 1606, 1564, 1544 (C=C), 1534, 1486, 1351, 1214 (C-O), 1161, 1128, 1098 (C-O), 989, 978, 913 (N-O), 841, 778, 734, 713. (F), 601, 512. ^{1}H NMR (400 MHz, $CDCl_3$) δ 8.10 (dd, *J* = 8.8, 5.4 Hz, 2H), 7.17 (t, *J* = 8.7 Hz, 2H), 6.76 (dd, *J* = 17.7, 11.0 Hz, 1H), 6.59 (d, *J* = 17.4 Hz, 1H), 6.01 (d, *J* = 11.1 Hz, 1H). ^{13}C NMR (101 MHz, $CDCl_3$) δ 167.9, 165.9, 163.4, 129.7, 129.6, 123.1, 120.5, 116.0.

3-(4-Iodophenyl)-5-vinyl-1,2,4-oxadiazole (**6d**)

White solid. Yield: (315 mg, 51%). M.p. 82-84 ºC. IR (ATR, cm^{-1}): 3077(=CH), 3001 (arom. CH), 1908, 1642 (C=N), 1569, 1532(C=C), 1486, 1454, 1400, 1349, 1281 (C-O), 1117, 1107, 1054 (C-O), 989, 980, 951 (N-O), 838, 785, 729, 690 (C-I), 667, 501. ^{1}H NMR (400 MHz, $CDCl_3$) δ 7.85 (d, *J* = 9.4 Hz, 2H), 7.37 (d, *J* = 8.4 Hz, 2H), 6.77 (d, *J* = 6.7 Hz, 1H), 6.60 (d, *J* = 17.6 Hz, 1H), 6.02 (d, *J* = 11.0 Hz, 1H). ^{13}C NMR (101 MHz, $CDCl_3$) δ 174.7, 168.2, 138.2, 133.2, 129.0, 126.3, 120.5, 98.0.

3-(4-Chlorophenyl)-5-vinyl-1,2,4-oxadiazole (**6e**)

Cream-colored solid. Yield: (325 mg, 75 %). M.p. 63-65 ºC. IR (ATR, cm^{-1}): 3120-3058 (=CH), 3014-2958 (arom. CH), 2858, 1920, 1645 (C=N), 1559, 1541 (C=C), 1488, 1408, 1349, 1289 (C-O), 1178, 1107, 1089 (C-O), 1013, 957, 904 (N-O), 836, 785, 757, 730. (Cl), 568, 519. ^{1}H NMR (400 MHz, $CDCl_3$) δ 8.05 (d, *J* = 8.6 Hz, 2H), 7.47 (d, *J* = 6.7 Hz, 2H), 6.75 (d, *J* = 11.0 Hz, 1H), 6.60 (d, *J* = 17.5 Hz, 1H), 6.02 (d, *J* = 11.1 Hz, 1H). ^{13}C NMR (101 MHz, $CDCl_3$) δ 174.7, 168.0, 137.4, 129.3, 129.1, 128.8, 125.3, 120.5.

3-(4-Methoxyphenyl)-5-vinyl-1,2,4-oxadiazole (**6f**)

White solid. Yield: (630 mg, 58%). M.p. 59-61°C. Rf (25% EtOAc/hexane): 0.72. IR (ATR, cm^{-1}): 3107(=CH), 3075-3002 (arom. CH), 2971, 2840 (CH_3), 1651, 1611(C=N), 1585 (C=N), 1538 (arom C=C), 1480 (arom C=C), 1441, 1419, 1365, 1255 (C-O), 1177, 1107, 1027 (C-O), 994, 948, 904, 839 (N-O), 814, 787, 740, 685, 614, 518. ^{1}H NMR (400 MHz, $CDCl_3$) δ 8.04 (d, *J* = 8.9 Hz, 2H), 6.99 (d, *J* = 8.9 Hz, 2H), 6.75 (dd, *J* = 17.7, 11.0 Hz, 1H), 6.57 (d, *J* = 18.3 Hz, 1H), 5.98 (d, *J* = 11.1 Hz, 1H), 3.86 (s, 3H). ^{13}C NMR (101 MHz, $CDCl_3$) δ 174.2, 168.4, 161.9, 129.0, 128.6, 120.6, 119.2, 114.2, 55.4.

3-Phenyl-5-vinyl-1,2,4-oxadiazole (**6g**)

Colorless oil. Yield: (500 mg, 65%). B. p. (294°C). Rf (25% EtOAc/hexane). IR (neat cm^{-1}): V_{max} = 3070 (arom. CH), 2926, 1646 (C=N), 1543 (arom C=C), 1445, 1360, 956, 910, 770, 705. ^{1}H NMR (400 MHz, $CDCl_3$) δ 8.15-8.06 (m, 2H), 7.57-7.44 (m, 3H), 6.83-6.55 (m, 2H), 6.01-5.97 (m, 1H).

3-(4-(Methylthio)phenyl)-5-vinyl-1,2,4-oxadiazole (**6h**)

White solid. Yield: (700 mg, 60%). M.p. 58-60°C. Rf (25% EtOAc/hexane) 0.71. IR (ATR, cm^{-1}): 3075-3000 (arom. CH), 2923 (CH_3), 1645, 1595 (C=N), 1580 (C=N), 1566 (arom C=C), 1537 (arom C=C), 1437, 1409, 1339, 1280 (C-O), 1183, 1117, 1088 (C-O), 1019, 946, 905, 829 (N-O), 784, 735, 673, 507, 488. ^{1}H-NMR (400 MHz, $CDCl_3$). ^{1}H NMR (400 MHz, $CDCl_3$) δ 8.00 (d, *J* = 8.5 Hz, 2H), 7.32 (d, *J* = 8.5 Hz, 2H), 6.75 (dd, *J* = 17.7, 11.0 Hz, 1H), 6.58 (d, *J* = 17.7 Hz, 1H), 5.99 (d, *J* = 11.0 Hz, 1H), 2.52 (s, 3H). ^{13}C NMR (101 MHz, $CDCl_3$) δ 174.4, 168.3, 142.9, 128.8, 127.7, 125.8, 123.0, 120.5, 15.1.

N,*N*-Dimethyl-4-(5-vinyl-1,2,4-oxadiazol-3-yl)aniline (**6i**)

White solid. Yield: (650 mg, 26%). M.p. 89-91°C. Rf (25% EtOAc/hexane) 0.79. IR (ATR, cm^{-1}): 3075 (arom. CH), 2910, 2813 (CH_3), 1647 (C=N), 1607 (C=N), 1566, 1534 (arom C=C), 1480 (arom C=C), 1430, 1412, 1357, 1281 (C-O), 1189, 1114, 1070 (C-O), 997, 960, 946, 901, 831 (N-O), 785, 741, 700, 676, 593, 521, 486. **Q** ^{1}H NMR (400 MHz, $CDCl_3$) δ 7.96 (d, *J* = 9.1 Hz, 2H), 6.78 (d, *J* = 9.1 Hz, 2H), 6.75 – 6.70 (m, 1H), 6.56 (d, *J* = 17.7 Hz, 1H), 5.96 (d, *J* = 11.1 Hz, 1H), 3.04 (s, 6H). ^{13}C NMR (101 MHz, $CDCl_3$) δ 173.8, 168.8, 152.2, 128.6, 128.2, 120.8, 113.9, 111.7, 40.2.

3-(*p*-Tolyl)-5-vinyl-1,2,4-oxadiazole (**6j**)

White solid. Yield: (430 mg, 52%). M.p. 51-53°C. Rf (25% EtOAc/hexane) 0.89. IR (ATR, cm^{-1}): 3074, 3048, 3029 (arom. CH), 2919 (CH_3), 1647, 1610 (C=N), 1581 (C=N), 1538 (arom C=C), 1477 (arom C=C), 1412, 1362, 1280 (C-O), 1181, 1114, 1018 (C-O), 991, 961, 909, 827 (N-O), 786, 731, 684, 606, 504, 426. ^{1}H NMR (400 MHz, $CDCl_3$) δ 8.00 (d, *J* = 8.2 Hz, 2H), 7.30 (d, *J* = 8.0 Hz, 2H), 6.77 (dd, *J* = 17.7, 11.0 Hz, 1H), 6.59 (d, *J* = 17.7 Hz, 1H), 6.00 (d, *J* = 11.0 Hz, 1H), 2.43 (s, 3H). ^{13}C NMR (101 MHz, $CDCl_3$) δ 174.3, 168.7, 141.5, 129.6, 128.63, 127.3, 123.9, 120.6, 21.6.

General procedure for the synthesis of 1,2,4-oxadiazole-isoxazoline hybrids (***7a–ay***)

Aryl vinyl-1,2,4-oxadiazole **6** (0.75 mmoles, 1.5 equiv) was dissolved in 5 ml of DCM and stirred for 5 minutes. Then Et_3N (0.5 mmoles, 1 equiv) and NaOCl (7.5 mmol, 15 equiv) were added into the mixture successively. While stirring, aldoxime **2** was added portionwise within 15 minutes. The resulting mixture was stirred at room temperature, controlled by TLC until consumption of starting materials. Then, the crude product was extracted twice with DCM (2 × 30 mL), dried over Na_2SO_4, filtered and DCM was evaporated at 30 °C using a rotary evaporator. The resulting solid residue was purified by silica gel column chromatography using EtOAc/hexane mixtures to afford the target product in moderate yields.

4-(5-(3-(4-Iodophenyl)-1,2,4-oxadiazol-5-yl)-4,5-dihydroisoxazol-3-yl)-*N*,*N*-dimethylaniline (**7a**)

Brown semi-solid. Yield: (92 mg, 40%). IR (ATR, cm^{-1}): 3085 (arom.CH) 2942-2869 (aliph.CH), 2836, 2788, 1660 (C=N), 1590(C=N), 1554, 1505, 1435 (C=C arom), 1400, 1357, 1335, 1242 (C-O), 1163, 1137, 1055 (C-O), 1007, 963, 885 (N-O), 815, 758, 727, 682, 594. ^{1}H NMR (400 MHz, $CDCl_3$) δ 7.81 (d, *J* = 6.2 Hz, 2H), 7.72 (dd, *J* = 15.6, 5.5 Hz, 2H), 7.57 (dd, *J* = 8.5, 2.0 Hz, 2H), 6.71 (d, *J* = 8.9 Hz, 2H), 5.93 (dd, *J* = 11.2, 6.5 Hz, 1H), 3.97 (dd, *J* = 16.7, 6.5 Hz, 1H), 3.84 (dd, *J* = 16.7, 11.2 Hz, 1H), 3.09 (s, 3H), 2.90 (s, 3H). ^{13}C NMR (101 MHz, $CDCl_3$) δ 176.7, 168.1, 155.2, 138.2, 129.6, 129.1, 127.5, 126.3, 119.8, 111.1, 98.4, 73.0, 40.2, 40.1. HRMS (ESI^+): m/z calcd for $C_{19}H_{17}IN_4O_2$ M^+: 460.0396; found: 460.0393.

5-(3-(4-Chlorophenyl)-4,5-dihydroisoxazol-5-yl)-3-(4-iodophenyl)-1,2,4-oxadiazole (**7b**)

White solid. Yield: (125 mg, 56%). M.p. 150-152°C. IR (ATR, cm^{-1}): 3070 (arom.CH), 2953, 2928, 2865 (aliph.CH), 1590 (C=N), 1563(C=N), 1520 (C=C arom), 1467 (C=C arom), 1417,

1355, 1301, 1275 (C-O), 1114, 1058 (C-O), 1005, 916, 834 (N-O), 794, 687, 562. ^{1}H NMR (400 MHz, $CDCl_3$) δ 7.83 (d, *J* = 8.6 Hz, 2H), 7.79 (d, *J* = 8.6 Hz, 2H), 7.67 (d, *J* = 8.6 Hz, 2H), 7.42 (d, *J* = 8.6 Hz, 2H), 5.96 (dd, *J* = 11.3, 6.6 Hz, 1H), 4.00 (dd, *J* = 16.8, 6.6 Hz, 1H), 3.87 (dd, *J* = 16.8, 11.3 Hz, 1H). ^{13}C NMR (101 MHz, $CDCl_3$) δ 176.59, 168.13, 155.58, 138.29, 137.06, 129.36, 129.11, 128.42, 126.74, 125.72, 98.51, 73.26, 39.99. HRMS (ESI-): m/z calcd for $C_{17}H_{10}ClIN_3O_2$: $[M-H]^+$:449.9506; found: 449.9530.

3-(4-Iodophenyl)-5-(3-(4-methoxyphenyl)-4,5-dihydroisoxazol-5-yl)-1,2,4-oxadiazole (**7c**)

Light brown solid. Yield: (98 mg, 44%). M.p. 140-142ºC. IR (ATR, cm^{-1}): 3060 (arom.CH), 2950, 2851, 2832 (aliph.CH), 1593 (C=N), 1562(C=N), 1514 (C=C arom), 1462 (C=C arom), 1441, 11403, 1355, 1251(C-O), 1174, 11022 (C-O), 1007, 927, 866 (N-O), 747, 545, 451. ^{1}H NMR (400 MHz, $CDCl_3$) δ 7.85 – 7.76 (m, 4H), 7.67 (d, *J* = 8.8 Hz, 2H), 6.95 (d, *J* = 8.8 Hz, 2H), 5.92 (dd, *J* = 11.1, 6.5 Hz, 1H), 4.00 (d, *J* = 6.6 Hz, 1H), 3.96 (d, *J* = 6.6 Hz, 1H), 3.86 (s, 3H). ^{13}C NMR (101 MHz, $CDCl_3$) δ 176.9, 168.1, 161.7, 156.0, 138.2, 129.1, 128.8, 125.8, 120.7, 114.4, 98.4, 72.8, 55.5, 40.4. HRMS (ESI$^+$): m/z calcd for $C_{18}H_{15}IN_3O_3$ M+H$^+$: 448.0158; found: 448.0167.

3-(4-Iodophenyl)-5-(3-(4-isopropylphenyl)-4,5-dihydroisoxazol-5-yl)-1,2,4-oxadiazole (**7d**)

Cream-colored solid. Yield: (86 mg, 38%). M.p. 142-144 ºC. IR (ATR, cm^{-1}): 3093 (arom.CH), 3059, 2944 (aliph.CH), 1590 (C=N), 1562(C=N), 1515 (C=C arom), 1468 (C=C arom), 1439, 1397, 1334, 1239(C-O), 1180, 1092 (C-O), 1005, 913, 877 (N-O), 756, 543, 437. ^{1}H NMR (400 MHz, $CDCl_3$) δ 7.83 – 7.76 (m, 4H), 7.65 (d, *J* = 8.3 Hz, 2H), 7.32 – 7.24 (m, 2H), 5.92 (dd, *J* = 11.2, 6.5 Hz, 1H), 4.00 (dd, *J* = 16.7, 6.5 Hz, 1H), 3.87 (dd, *J* = 16.7, 11.2 Hz, 1H), 2.95 (dt, *J* = 13.8, 6.9 Hz, 1H), 1.26 (d, *J* = 6.9 Hz, 6H). ^{13}C NMR (101 MHz, $CDCl_3$) δ 176.9, 168.0, 156.3, 152.2, 138.2, 129.1, 127.3, 127.1, 125.8, 125.7, 98.4, 72.9, 40.3, 34.2, 23.9. HRMS (ESI$^+$): m/z calcd for $C_{20}H_{18}IN_3O_2$ M$^+$: 459.0443; found 459.0432.

3-(4-Iodophenyl)-5-(3-(p-tolyl)-4,5-dihydroisoxazol-5-yl)-1,2,4-oxadiazole (**7e**)

Yellow crystalline solid. Yield: (86 mg, 40%). M.p. 102-104 ºC. IR (ATR, cm^{-1}): 3033 (arom.CH), 2912, 2853 (aliph.CH), 1594 (C=N), 1565 (C=N), 1515 (C=C arom), 1463 (C=C arom), 1404, 1349, 1339, 1220 (C-O), 1185, 1057 (C-O), 1007, 928, 888 (N-O), 747, 543, 454. ^{1}H NMR (400 MHz, $CDCl_3$) δ 7.84 – 7.76 (m, 4H), 7.61 (d, *J* = 8.2 Hz, 2H), 7.25 (d, *J* = 8.1 Hz, 2H), 5.93 (dd, *J* = 11.2, 6.6 Hz, 1H), 3.99 (dd, *J* = 16.8, 6.6 Hz, 1H), 3.87 (dd, *J* = 16.8, 11.2 Hz, 1H), 2.40 (s, 3H). ^{13}C NMR (101 MHz, $CDCl_3$) δ 176.9, 168.0, 156.4, 141.3,

138.2, 129.7, 129.1, 127.1, 125.8, 125.3, 98.4, 72.9, 40.2, 21.6. HRMS (ESI^+): m/z calcd for $C_{18}H_{14}IN_3O_2$ M^+: 431.0130; found 431.0119.

5-(3-(4-Chlorophenyl)-4,5-dihydroisoxazol-5-yl)-3-(4-nitrophenyl)-1,2,4-oxadiazole (**7f**)

Cream-colored crystal. Yield: (94 mg, 51%). M.p. 164-166 ºC. IR (ATR, cm^{-1}): 3098 (arom.CH), 2920, 2850 (aliph.CH), 1610 (C=N), 1576, 1526 (C=N), 1516 (NO_2), 1439 (C=C arom), 1416, 1339 (NO_2), 1308, 1287 (C-O), 1121, 1106, 1089 (C-O), 1009, 887, 861 (N-O), 747, 728, 681, 511, 448. ^{1}H NMR (400 MHz, $CDCl_3$) δ 8.34 (d, *J* = 8.9 Hz, 2H), 8.27 (d, *J* = 8.9 Hz, 2H), 7.67 (d, *J* = 8.6 Hz, 2H), 7.43 (d, *J* = 8.6 Hz, 2H), 6.00 (dd, *J* = 11.2, 6.5 Hz, 1H), 4.01 (dd, *J* = 16.8, 6.5 Hz, 1H), 3.90 (dd, *J* = 16.8, 11.2 Hz, 1H). ^{13}C NMR (101 MHz, $CDCl_3$) δ 177.2, 167.1, 155.6, 149.7, 137.2, 132.1, 129.4, 128.7, 128.4, 126.6, 124.3, 73.1, 40.0. HRMS (ESI^+): m/z calcd for $C_{17}H_{11}ClN_4O_4$ M^+: 370.0468; found 370.0499.

N,*N*-Dimethyl-4-(5-(3-(4-nitrophenyl)-1,2,4-oxadiazol-5-yl)-4,5-dihydroisoxazol-3-yl)aniline (**7g**)

Orange solid. Yield: (60 mg, 32%). M.p. 116-118 ºC. IR (ATR, cm^{-1}): 3098 (arom.CH), 2923, 2849, 2793 (aliph.CH), 1615 (C=N), 1599, 1576 (C=N), 1514 (NO_2), 1455 (C=C arom), 1431, 1415, 1342(NO_2), 1275 (C-O), 1169, 1103, 1047 (C-O), 1012, 945, 911, 861 (N-O), 785, 736, 590, 513, 478. ^{1}H NMR (400 MHz, $CDCl_3$) 8.33 (d, *J* = 8.9 Hz, 2H), 8.27 (d, *J* = 8.9 Hz, 2H), 7.57 (dd, *J* = 8.5, 2.0 Hz, 2H), 7.18 (d, *J* = 70.2 Hz, 2H), 5.97 (dd, *J* = 11.1, 6.3 Hz, 1H), 3.98 (dd, *J* = 16.7, 6.3 Hz, 1H), 3.88 (dd, *J* = 16.7, 11.1 Hz, 1H), 2.90 (s, 6H). ^{13}C NMR (101 MHz, $CDCl_3$) δ 177.4, 167.1, 155.3, 149.7, 132.1, 129.7, 128.6, 127.5, 126.3, 124.2, 119.8, 72.9, 43.4, 40.1. HRMS (ESI^+): m/z calcd for $C_{19}H_{17}N_5O_4$ M^+: 379.1280; found 379.1268.

5-(3-(4-Methoxyphenyl)-4,5-dihydroisoxazol-5-yl)-3-(4-nitrophenyl)-1,2,4-oxadiazole (**7h**)

Cream-colored solid. Yield: (90 mg, 49%). M.p. 164-165ºC. IR (ATR, cm^{-1}): 3103-3019 (arom.CH), 2967, 2936, 2837 (aliph.CH), 1608 (C=N), 1599, 1569 (C=N), 1515 (NO_2), 1480 (C=C arom), 1429, 1412, 1332(NO_2), 1288 (C-O), 1180, 1102, 1041 (C-O), 1023, 949, 912, 872 (N-O), 839, 708, 601, 549, 440. ^{1}H NMR (400 MHz, $CDCl_3$) δ 8.35 (d, *J* = 8.9 Hz, 2H), 8.29 (d, *J* = 8.9 Hz, 2H), 7.69 (d, *J* = 8.8 Hz, 2H), 6.98 (d, *J* = 8.8 Hz, 2H), 5.97 (dd, *J* = 11.1, 6.4 Hz, 1H), 4.00 (d, *J* = 6.4 Hz, 1H), 3.94 (d, *J* = 11.1 Hz, 1H), 3.88 (s, 3H). ^{13}C NMR (101 MHz, $CDCl_3$) δ 177.6, 167.1, 161.8, 156.0, 149.7, 132.2, 128.8, 128.6, 124.2, 120.5,

114.4, 72.7, 55.5, 40.4. HRMS (ESI$^+$): m/z calcd for $C_{18}H_{15}N_4O_5$ [M+H]$^+$: 367.1042; found 367.1051.

5-(3-(4-Isopropylphenyl)-4,5-dihydroisoxazol-5-yl)-3-(4-nitrophenyl)-1,2,4-oxadiazole (**7i**)

Cream-colored solid. Yield: (127 mg, 68%). M.p. 131-133ºC. IR (ATR, cm^{-1}): 3109 (arom.CH), 2961, 2927, 2869 (aliph.CH), 1609 (C=N), 1583, 1524 (C=N), 1474 (NO_2), 1437 (C=C arom), 1415, 1338 (NO_2), 1312, 1267 (C-O), 1156, 1105, 1054 (C-O), 1011, 968, 921, 893 (N-O), 833, 744, 706, 604, 560, 475. ^{1}H NMR (400 MHz, $CDCl_3$) δ 8.35 (d, J = 8.9 Hz, 2H), 8.29 (d, J = 8.9 Hz, 2H), 7.69 (d, J = 8.3 Hz, 2H), 7.33 (d, J = 8.3 Hz, 2H), 5.99 (dd, J = 11.1, 6.4 Hz, 1H), 4.09 – 4.01 (m, 1H), 3.94 (dd, J = 16.8, 11.1 Hz, 1H), 2.98 (dt, J = 13.8, 6.9 Hz, 1H), 1.29 (d, J = 6.9 Hz, 6H). ^{13}C NMR (101 MHz, $CDCl_3$) δ 177.6, 167.1, 156.4, 152.3, 149.6, 132.1, 128.6, 127.3, 127.1, 125.6, 124.2, 72.8, 40.3, 34.2, 23.8. HRMS (ESI$^+$): m/z calcd for $C_{20}H_{18}N_4O_4$ M$^+$: 378.1328; found 378.1321.

3-(4-Nitrophenyl)-5-(3-(*p*-tolyl)-4,5-dihydroisoxazol-5-yl)-1,2,4-oxadiazole (**7j**)

Cream-colored solid. Yield: (150 mg, 86%). M.p. 141-143 ºC. IR (ATR, cm^{-1}): 3106-3081 (arom.CH), 3039, 2937, 2854 (aliph.CH), 1603 (C=N), 1584, 1536 (C=N), 1477 (NO_2), 1430 (C=C arom), 1415, 1350 (NO_2), 1332, 1296 (C-O), 1187, 1110, 1102 (C-O), 1015, 943, 862, 851 (N-O), 808, 728, 711, 636, 540, 451. ^{1}H NMR (400 MHz, $CDCl_3$) δ 8.34 (d, J = 8.8 Hz, 2H), 8.28 (d, J = 8.9 Hz, 2H), 7.64 (d, J = 8.1 Hz, 2H), 7.27 (d, J = 7.8 Hz, 2H), 5.98 (dd, J = 11.1, 6.5 Hz, 1H), 4.03 (dd, J = 16.8, 6.5 Hz, 1H), 3.93 (dd, J = 16.8, 11.1 Hz, 1H), 2.42 (s, 3H). ^{13}C NMR (101 MHz, $CDCl_3$) δ 177.6, 167.1, 156.4, 149.6, 141.5, 132.1, 129.7, 128.6, 127.1, 125.2, 124.2, 72.8, 40.3, 21.6. HRMS (ESI$^+$): m/z calcd for $C_{18}H_{14}N_4O_4$ M$^+$: 350.1015; found 350.1003.

4-(5-(3-(4-Fluorophenyl)-1,2,4-oxadiazol-5-yl)-4,5-dihydroisoxazol-3-yl)-*N*,*N*-dimethylaniline (**7k**)

Brown liquid. Yield: (65 mg, 37%). IR (ATR, cm^{-1}): 3075 (arom.CH), 2921, 2870, 2810 (aliph.CH), 1645 (C=N), 1596 (C=N), 1526, 1476 (C=C arom), 1416, 1355, 1223 (C-O), 1189, 1153, 1093 (C-O), 1010, 946, 844 (N-O), 805, 760, 681, 517. ^{1}H NMR (400 MHz, $CDCl_3$) δ 7.80 (d, J = 10.2 Hz, 2H), 7.72 – 7.63 (m, 2H), 7.25 (d, J = 6.6 Hz, 2H), 6.81 (dd, J = 16.7, 8.6 Hz, 2H), 6.02 (dd, J = 11.2, 6.5 Hz, 1H), 4.06 (dd, J = 16.7, 6.5 Hz, 1H), 3.95 (d, J = 11.3 Hz, 1H), 2.99 (s, 6H). ^{13}C NMR (101 MHz, $CDCl_3$) δ 176.6, 167.7, 155.2, 129.9,

129.8, 129.6, 128.5, 127.5, 126.3, 119.8,116.3, 116.1, 73.0, 43.4, 40.0. HRMS (ESI$^+$): m/z calcd for $C_{19}H_{17}FN_4O_2$ M$^+$: 352.1335; found 352.1313.

5-(3-(4-Chlorophenyl)-4,5-dihydroisoxazol-5-yl)-3-(4-fluorophenyl)-1,2,4-oxadiazole (**7l**)

Cream-colored solid. Yield: (100 mg, 58%). M.p. 120-122 °C. IR (ATR, cm^{-1}): 3050 (arom.CH), 2983 (aliph.CH), 1605 (C=N), 1595 (C=N), 1538, 1494 (C=C arom), 1419, 1347, 1240 (C-O), 1158, 1116, 1092 (C-O), 1010, 966, 885 (N-O), 823, 764, 613, 521, 458. ^{1}H NMR (400 MHz, $CDCl_3$) δ 8.06 (dd, *J* = 8.8, 5.4 Hz, 2H), 7.65 (d, *J* = 8.6 Hz, 2H), 7.41 (d, *J* = 8.6 Hz, 2H), 7.14 (t, *J* = 8.7 Hz, 2H), 5.95 (dd, *J* = 11.3, 6.6 Hz, 1H), 3.98 (dd, *J* = 16.8, 6.6 Hz, 1H), 3.86 (dd, *J* = 16.8, 11.3 Hz, 1H). ^{13}C NMR (101 MHz, $CDCl_3$) δ 176.2, 167.8, 166.0, 163.5, 155.6, 137.0, 129.8, 129.3, 128.4, 126.7, 122.4, 116.1, 73.2, 39.9. HRMS (ESI$^+$): m/z calcd for $C_{17}H_{11}ClFN_3O_2$ M$^+$: 343.0523; found 343.0516.

3-(4-Fluorophenyl)-5-(3-(4-methoxyphenyl)-4,5-dihydroisoxazol-5-yl)-1,2,4-oxadiazole (**7m**)

Cream-colored solid. Yield: (101 mg, 60%). M.p.108-110ºC. IR (ATR, cm^{-1}): 3024-3008 (arom.CH), 2973, 2938, 2838 (aliph.CH), 1606 (C=N), 1595 (C=N), 1539, 1478 (C=C arom), 1419, 1372, 1237 (C-O), 1175, 1156, 1021 (C-O), 1006, 960, 894 (N-O), 846, 825, 761, 602, 541, 449.^{1}H NMR (400 MHz, $CDCl_3$) δ 8.09 (dd, *J* = 8.9, 5.4 Hz, 2H), 7.69 (d, *J* = 8.9 Hz, 2H), 7.17 (t, *J* = 8.7 Hz, 2H), 6.97 (d, *J* = 8.9 Hz, 2H), 5.93 (dd, *J* = 11.1, 6.5 Hz, 1H), 4.02 (d, *J* = 6.6 Hz, 1H), 3.98 (d, *J* = 6.5 Hz, 1H), 3.87 (s, 3H). ^{13}C NMR (101 MHz, $CDCl_3$) δ 176.7, 167.8, 161.7, 159.9, 129.9, 128.8, 122.5, 120.7, 116.3, 116.1, 114.5, 114.4, 72.8, 55.5, 40.4. HRMS (ESI$^+$): m/z calcd for $C_{18}H_{14}FN_3O_3$ M$^+$: 339.1019; found 339.1011.

3-(4-Fluorophenyl)-5-(3-(4-isopropylphenyl)-4,5-dihydroisoxazol-5-yl)-1,2,4-oxadiazole (**7n**)

Yellow solid. Yield: (135 mg, 77%). M.p. 86-88ºC. IR (ATR, cm^{-1}): 3070 (arom.CH), 2960, 2869 (aliph.CH), 1606 (C=N), 1581 (C=N), 1537, 1475 (C=C arom), 1416, 1355, 1218 (C-O), 1155, 1113, 1055 (C-O), 1012, 919, 887 (N-O), 846, 766, 601, 527, 455. ^{1}H NMR (400 MHz, $CDCl_3$) δ 8.09 (dd, *J* = 8.8, 5.4 Hz, 2H), 7.68 (d, *J* = 8.3 Hz, 2H), 7.32 (d, *J* = 8.3 Hz, 2H), 7.17 (t, *J* = 8.7 Hz, 2H), 5.94 (dd, *J* = 11.2, 6.5 Hz, 1H), 4.02 (dd, *J* = 16.7, 6.5 Hz, 1H), 3.89 (dd, *J* = 16.7, 11.2 Hz, 1H), 2.97 (dt, *J* = 13.8, 6.9 Hz, 1H), 1.28 (d, *J* = 6.9 Hz, 6H). ^{13}C NMR (101 MHz, $CDCl_3$) δ 176.6, 167.8, 166.0, 163.5, 156.4, 152.2, 129.9, 129.8, 127.2, 125.7, 122.6, 116.1, 72.9, 40.2, 34.2, 23.8. HRMS (ESI$^+$): m/z calcd for $C_{20}H_{19}FN_3O_2$ [M+H]$^+$: 352.1461; found 352.1468.

3-(4-Fluorophenyl)-5-(3-(p-tolyl)-4,5-dihydroisoxazol-5-yl)-1,2,4-oxadiazole (**7o**)

Cream-colored solid. Yield: (154 mg, 96%). M.p. 97-99ºC. IR (ATR, cm^{-1}): 3060 (arom.CH), 2952, 2920, 2851 (aliph.CH), 1607 (C=N), 1596 (C=N), 1538, 1478 (C=C arom), 1417, 1353, 1295 (C-O), 1227, 1186, 1155 (C-O), 1004, 912, 877 (N-O), 817, 765, 616, 543, 456. ^{1}H NMR (400 MHz, $CDCl_3$) δ 8.09 (dd, *J* = 8.8, 5.4 Hz, 2H), 7.64 (d, *J* = 8.2 Hz, 2H), 7.27 (d, *J* = 8.4 Hz, 2H), 7.17 (t, *J* = 8.7 Hz, 2H), 5.95 (dd, *J* = 11.2, 6.6 Hz, 1H), 4.02 (dd, *J* = 16.8, 6.6 Hz, 1H), 3.89 (dd, *J* = 16.8, 11.2 Hz, 1H), 2.42 (s, 3H). ^{13}C NMR (101 MHz, $CDCl_3$) δ 176.80, 167.82, 166.06, 163.55, 156.42, 141.37, 129.82, 129.73, 127.14, 122.56, 116.12, 72.94, 40.28, 21.65. HRMS (ESI^+): m/z calcd for $C_{18}H_{14}FN_3O_2$ M^+: 323.1070; found 323.1066.

3-(4-Chlorophenyl)-5-(3-(4-chlorophenyl)-4,5-dihydroisoxazol-5-yl)-1,2,4-oxadiazole (**7p**)

Cream-colored crystals. Yield: (100 mg, 56%). M.p. 122-124ºC. IR (ATR, cm^{-1}): 3064 (arom.CH), 2928 (aliph.CH), 1596 (C=N), 1567 (C=N), 1494, 1472 (C=C arom), 1405, 1347, 1280 (C-O), 1241, 1114, 1092 (C-O), 1013, 933, 826 (N-O), 750, 687, 506, 452. ^{1}H NMR (400 MHz,$CDCl_3$) δ 8.02 (d, *J* = 8.5 Hz, 2H), 7.67 (d, *J* = 8.5 Hz, 2H), 7.44 (dd, *J* = 10.6, 8.5 Hz, 4H), 5.97 (dd, *J* = 11.3, 6.6 Hz, 1H), 4.01 (dd, *J* = 16.8, 6.6 Hz, 1H), 3.88 (dd, *J* = 16.8, 11.3 Hz, 1H).^{13}C NMR (101 MHz,$CDCl_3$) δ 176.5, 167.8, 155.5, 137.8, 137.0, 129.3, 129.3, 128.9, 128.4, 126.7, 124.7, 73.2, 39.9. HRMS (ESI^+): m/z calcd for $C_{17}H_{11}Cl_2N_3O_2$ M^+: 359.0228; found 359.0216.

3-(4-Chlorophenyl)-5-(3-(4-methoxyphenyl)-4,5-dihydroisoxazol-5-yl)-1,2,4-oxadiazole (**7q**)

White solid. Yield: (92 mg, 52%). M.p. 111-113ºC. IR (ATR, cm^{-1}): 3022 (arom.CH), 2979, 2936, 2840 (aliph.CH), 1598 (C=N), 1588 (C=N), 1514, 1468 (C=C arom), 1409, 1339, 1279 (C-O), 1221, 1176, 1064 (C-O), 1022, 999, 883 (N-O), 820, 751, 686, 519, 449. ^{1}H NMR (400 MHz,$CDCl_3$) δ 8.00 (d, *J* = 8.5 Hz, 2H), 7.66 (d, *J* = 8.8 Hz, 2H), 7.44 (d, *J* = 8.5 Hz, 2H), 6.94 (d, *J* = 8.8 Hz, 2H), 5.91 (dd, *J* = 11.1, 6.5 Hz, 1H), 3.98 (dd, *J* = 16.7, 6.5 Hz, 1H), 3.88 (d, *J* = 11.1 Hz, 1H), 3.85 (s, 3H).^{13}C NMR (101 MHz, $CDCl_3$) δ 176.9, 167.8, 161.7, 156.0, 137.7, 129.3, 128.9, 128.7, 124.8, 120.7, 114.4, 72.8, 55.5, 40.4. HRMS (ESI^+): m/z calcd for $C_{18}H_{14}ClN_3O_3$ M^+: 355.0723; found 355.0724.

3-(4-Chlorophenyl)-5-(3-(4-isopropylphenyl)-4,5-dihydroisoxazol-5-yl)-1,2,4-oxadiazole (**7r**)

Light yellow crystal solid. Yield: (135 mg, 74%). M.p. 116-118ºC. IR (ATR, cm^{-1}): 3070 (arom.CH), 2956, 2926, 2868 (aliph.CH), 1591 (C=N), 1569 (C=N), 1464, 1438 (C=C arom), 1407, 1353, 1277 (C-O), 1171, 1088, 1056 (C-O), 1012, 918, 859 (N-O), 763, 689, 562, 482. ^{1}H NMR (400 MHz, $CDCl_3$) δ 8.02 (d, *J* = 8.5 Hz, 2H), 7.67 (d, *J* = 8.2 Hz, 2H), 7.45 (d, *J* = 8.5 Hz, 2H), 7.31 (d, *J* = 8.2 Hz, 2H), 5.94 (dd, *J* = 11.2, 6.5 Hz, 1H), 4.01 (dd, *J* = 16.7, 6.5 Hz, 1H), 3.89 (dd, *J* = 16.7, 11.2 Hz, 1H), 2.96 (dt, *J* = 13.8, 6.9 Hz, 1H), 1.28 (d, *J* = 6.9 Hz, 6H). ^{13}C NMR (101 MHz, $CDCl_3$) δ 176.9, 167.8, 156.4, 152.2, 137.7, 129.3, 128.9, 127.3, 127.1, 125.7, 124.8, 72.9, 40.3, 34.2, 23.8. HRMS (ESI^+): m/z calcd for $C_{20}H_{19}ClN_3O_2$ $[M+H]^+$: 368.1165; found 368.1172.

3-(4-Chlorophenyl)-5-(3-(*p*-tolyl)-4,5-dihydroisoxazol-5-yl)-1,2,4-oxadiazole (**7s**)

Cream-colored crystals. Yield: (111 mg, 66%). M.p. 99-101 °C. IR (ATR, cm^{-1}): 3080-3032 (arom.CH), 2981, 2920, 2858 (aliph.CH), 1598 (C=N), 1571 (C=N), 1515, 1467 (C=C arom), 1409, 1370, 1221 (C-O), 1116, 1087, 1037 (C-O), 1014, 920, 909 (N-O), 816, 751, 634, 514, 454. ^{1}H NMR (400 MHz, $CDCl_3$) δ 8.01 (d, *J* = 8.6 Hz, 2H), 7.61 (d, *J* = 8.2 Hz, 2H), 7.44 (d, *J* = 8.6 Hz, 2H), 7.24 (d, *J* = 8.0 Hz, 2H), 5.92 (dd, *J* = 11.2, 6.6 Hz, 1H), 3.99 (dd, *J* = 16.8, 6.6 Hz, 1H), 3.87 (dd, *J* = 16.8, 11.2 Hz, 1H), 2.39 (s, 3H). ^{13}C NMR (101 MHz, $CDCl_3$) δ 176.9, 167.8, 156.4, 141.3, 137.7, 129.7, 129.3, 128.9, 127.1, 125.3, 124.8, 72.9, 40.2, 21.6. HRMS (ESI^+): m/z calcd for $C_{18}H_{14}ClN_3O_2$ M^+: 339.0774; found 339.0766.

N,*N*-Dimethyl-4-(5-(3-(4-(trifluoromethyl)phenyl)-1,2,4-oxadiazol-5-yl)-4,5-dihydroisoxazol-3-yl)aniline (**7t**)

Dark red semi-solid. Yield: (75 mg, 38%). IR (ATR, cm^{-1}): 3095 (arom.CH), 2923, 2801 (aliph.CH), 1662 (C=N), 1595 (C=N), 1527, 1507 (C=C arom), 1437, 1416, 1321 (C-O), 1163, 1124, 1105 (CF_3), 1064, 1016 (C-O), 978, 945, 854 (N-O), 817, 767, 697, 594, 464. ^{1}H NMR (400 MHz, $CDCl_3$) δ 8.22 (d, *J* = 8.2 Hz, 2H), 7.75 (d, *J* = 6.7 Hz, 2H), 7.61 (t, *J* = 5.8 Hz, 2H), 6.72 (d, *J* = 9.0 Hz, 2H), 5.97 (dd, *J* = 11.2, 6.4 Hz, 1H), 4.00 (dd, *J* = 16.8, 6.4 Hz, 1H), 3.92 (d, *J* = 3.9 Hz, 1H), 3.10 (s, 6H). ^{13}C NMR (101 MHz, $CDCl_3$) δ 176.9, 167.7, 155.3, 129.7, 128.5, 128.0, 126.3, 126.0, 125.9, 119.8, 111.1, 72.9, 72.5, 43.4, 40.5, 40.2, 40.1. HRMS (ESI^+): m/z calcd for $C_{20}H_{17}F_3N_4O_2$ M^+: 402.1303; found 402.1300.

5-(3-(4-Chlorophenyl)-4,5-dihydroisoxazol-5-yl)-3-(4-(trifluoromethyl) phenyl)-1,2,4-oxadiazole (**7u**)

White solid. Yield: (119 mg, 61%). M.p. 128-130ºC. IR (ATR, cm^{-1}): 3090 (arom.CH), 2996 (aliph.CH), 1593 (C=N), 1569 (C=N), 1539, 1496 (C=C arom), 1445, 1355, 1326 (C-O), 1283, 1171, 1141 (CF_3), 1123, 1098, 1067, 1018 (C-O), 1018, 912, 884, 854 (N-O), 830, 711, 601, 540, 463. ^{1}H NMR (400 MHz, $CDCl_3$) δ 8.20 (d, *J* = 8.2 Hz, 2H), 7.74 (d, *J* = 8.3 Hz, 2H), 7.68 (d, *J* = 8.5 Hz, 2H), 7.43 (d, *J* = 8.5 Hz, 2H), 5.99 (dd, *J* = 11.2, 6.6 Hz, 1H), 4.02 (dd, *J* = 16.8, 6.6 Hz, 1H), 3.90 (dd, *J* = 16.8, 11.3 Hz, 1H). ^{13}C NMR (101 MHz, $CDCl_3$) δ 176.9, 167.7, 155.6, 137.1, 133.1, 129.6, 129.3, 128.4, 128.0, 126.7, 126.1, 126.0, 73.2, 40.0. HRMS (ESI$^+$): m/z calcd for $C_{18}H_{11}ClF_3N_3O_2$ M$^+$: 393.0491; found 393.0493.

5-(3-(4-Methoxyphenyl)-4,5-dihydroisoxazol-5-yl)-3-(4-(trifluoromethyl)phenyl)-1,2,4-oxadiazole (**7v**)

White solid. Yield: (93 mg, 48%). M.p. 119-121 ºC. IR (ATR, cm^{-1}): 3026 (arom.CH), 2974, 2948, 2849 (aliph.CH), 1597 (C=N), 1566 (C=N), 1515, 1489 (C=C arom), 1463, 1418, 1357 (C-O), 1308, 1250, 1161 (CF_3), 1114, 1061, 1021, 998 (C-O), 969, 927, 836 (N-O), 825, 778, 625, 545, 467. ^{1}H NMR (400 MHz, $CDCl_3$) δ 8.22 (d, *J* = 8.2 Hz, 2H), 7.75 (d, *J* = 8.3 Hz, 2H), 7.69 (d, *J* = 8.8 Hz, 2H), 6.97 (d, *J* = 8.9 Hz, 2H), 5.96 (dd, *J* = 11.1, 6.5 Hz, 1H), 4.02 (dd, *J* = 16.7, 6.5 Hz, 1H), 3.92 (d, *J* = 11.1 Hz, 1H), 3.87 (s, 3H). ^{13}C NMR (101 MHz, $CDCl_3$) δ 177.3, 167.6, 161.7, 156.0, 129.7, 128.8, 128.0, 126.0, 125.9, 125.9, 120.6, 114.4, 72.8, 55.5, 40.4. HRMS (ESI$^+$): m/z calcd for $C_{19}H_{14}F_3N_3O_3$ M$^+$: 389.0987; found 389.0978.

5-(3-(4-Isopropylphenyl)-4,5-dihydroisoxazol-5-yl)-3-(4-(trifluoromethyl) phenyl)-1,2,4-oxadiazole (**7w**)

Cream-colored crystals. Yield: (88 mg, 44%). M.p. 139-141 ºC. IR (ATR, cm^{-1}): 3075 (arom.CH), 2962, 2930, 2872 (aliph.CH), 1622 (C=N), 1598 (C=N), 1576, 1541 (C=C arom), 1465, 1417, 1356 (C-O), 1320, 1243, 1185 (CF_3), 1167, 1119, 1106, 1063 (C-O), 1016, 975, 918, 854, 834 (N-O), 768, 698, 561, 464. ^{1}H NMR (400 MHz, $CDCl_3$) δ 8.21 (d, *J* = 8.2 Hz, 2H), 7.74 (d, *J* = 8.3 Hz, 2H), 7.67 (d, *J* = 8.2 Hz, 2H), 7.32 (d, *J* = 8.2 Hz, 2H), 5.96 (dd, *J* = 11.2, 6.4 Hz, 1H), 4.03 (dd, *J* = 16.8, 6.4 Hz, 1H), 3.91 (dd, *J* = 16.8, 11.2 Hz, 1H), 2.96 (dt, *J* = 13.8, 6.9 Hz, 1H), 1.28 (d, *J* = 6.9 Hz, 6H). ^{13}C NMR (101 MHz, $CDCl_3$) δ 177.2, 167.6, 156.4, 152.3, 129.7, 128.0, 127.3, 127.1, 126.0, 126.0, 125.7, 125.1, 72.8, 40.3, 34.2, 23.8. HRMS (ESI$^+$): m/z calcd for $C_{21}H_{19}F_3N_3O_2$ [M+H]$^+$: 402.1429; found 402.1427.

5-(3-(*p*-Tolyl)-4,5-dihydroisoxazol-5-yl)-3-(4-(trifluoromethyl)phenyl)-1,2,4-oxadiazole (**7x**)

White crystal solid. Yield: (105 mg, 57%). M.p. 129-131 ºC. IR (ATR, cm^{-1}): 3027 (arom.CH), 2923 (aliph.CH), 1623 (C=N), 1600 (C=N), 1576, 1540 (C=C arom), 1484, 1442, 1417 (C-O), 1356, 1321, 1287 (CF_3), 1170, 1154, 1116, 1063 (C-O), 1016, 985, 922, 820 (N-O), 790 , 764, 697, 544, 465. ^{1}H NMR (400 MHz, $CDCl_3$) δ 8.20 (d, *J* = 8.2 Hz, 2H), 7.73 (d, *J* = 8.3 Hz, 2H), 7.62 (d, *J* = 8.1 Hz, 2H), 7.24 (d, *J* = 7.9 Hz, 2H), 5.95 (dd, *J* = 11.2, 6.5 Hz, 1H), 4.01 (dd, *J* = 16.8, 6.5 Hz, 1H), 3.89 (dd, *J* = 16.8, 11.2 Hz, 1H), 2.39 (s, 3H). ^{13}C NMR (101 MHz, $CDCl_3$) δ 177.2, 167.6, 156.4, 141.4, 133.4, 129.7, 128.0, 127.1, 126.0, 126.0, 125.3, 122.4, 72.9, 40.3, 21.6. HRMS (ESI$^+$): m/z calcd for $C_{19}H_{14}F_3N_3O_2$ M$^+$: 373.1038; found 373.1035.

5-(3-(4-Bromophenyl)-4,5-dihydroisoxazol-5-yl)-3-(4-methoxyphenyl)-1,2,4-oxadiazole (**7aa**)

White solid. Yield:100 mg, (50.0%). M.p. 141-143°C. Rf (33.3% EtOAc/hexane): 0.70. IR (ATR, cm^{-1}): 3019 (arom.CH), 2964, 2934, 2853 (aliph.CH), 1591 (C=N), 1574(C=N), 1539 (C=C arom), 1472 (C=C arom), 1411, 1337, 1304, 1251(C-O), 1105, 1069 (C-O), 1005, 899, 824 (N-O), 765, 537 (Br), 460. ^{1}H NMR (400 MHz, $CDCl_3$) δ 8.01 (d, *J* = 8.9 Hz, 2H), 7.70 – 7.46 (m, 4H), 6.98 (d, *J* = 8.8 Hz, 2H), 5.96 (dd, *J* = 11.3, 6.7 Hz, 1H), 4.00 (dd, *J* = 16.8, 6.7 Hz, 1H), 3.92 – 3.74 (m, 4H). ^{13}C NMR (101 MHz, $CDCl_3$) δ 175.9, 168.2, 162.1, 155.6, 132.2, 129.2, 128.5, 127.2, 125.2, 118.5, 114.3, 73.3, 55.4, 39.8. HRMS (ESI$^+$): m/z calcd for $C_{18}H_{15}BrN_3O_3$ [M+H]$^+$: 400.0296; found 400.0305.

5-(3-(4-Fluorophenyl)-4,5-dihydroisoxazol-5-yl)-3-(4-methoxyphenyl)-1,2,4-oxadiazole (**7ab**)

Yellowish solid. Yield: 60 mg (35.4%). M.p. 108-110°C. Rf (33.3% EtOAc/hexane) 0.66. IR (ATR, cm^{-1}): 3064, 3017(arom.CH), 2971, 2940, 2838 (aliph.CH), 1619, 1596 (C=N), 1575(C=N), 1513 (C=C arom), 1482 (C=C arom), 1411, 1341, 1301, 1264 (C-O), 1227, 1168 (C-F), 1106, 1028 (C-O), 986, 933, 847 (N-O), 752, 604, 546, 518, 457. ^{1}H NMR (400 MHz, $CDCl_3$) δ 8.00 (d, *J* = 8.8 Hz, 2H), 7.79 – 7.63 (m, 2H), 7.20 – 7.06 (m, 2H), 6.96 (d, *J* = 8.8 Hz, 2H), 5.93 (dd, *J* = 11.2, 6.6 Hz, 1H), 3.99 (dd, *J* = 16.7, 6.7 Hz, 1H), 3.90 – 3.78 (m, 4H). ^{13}C NMR (101 MHz, $CDCl_3$) δ 176.0, 168.2, 165.4, 162.9, 162.1, 155.4, 129.2, 129.0, 124.5, 118.6, 116.2, 116.0, 114.3, 73.2, 55.4, 40.1. HRMS (ESI$^+$): m/z calcd for $C_{18}H_{14}FN_3O_3$ M$^+$: 339.1019; found 339.1015.

3-(4-Methoxyphenyl)-5-(3-(4-(trifluoromethyl)phenyl)-4,5-dihydroisoxazol-5-yl)-1,2,4-oxadiazole (**7ac**)

White-cream solid. Yield: 120 mg (61.6%). M.p. 159-161°C. Rf (33.3% EtOAc/hexane) 0.62. IR (ATR, cm^{-1}): 3011 (arom.CH), 2970, 2933, 2838 (aliph.CH), 1607 (C=N), 1573(C=N), 1520 (C=C arom), 1482 (C=C arom), 1426, 1324, 1308, 1255(C-O), 1222,1159 (CF_3), 1105, 1066, 1025 (C-O), 1009, 964, 921, 894, 839 (N-O), 778, 745, 703, 636, 609, 526, 462. ^{1}H NMR (400 MHz, $CDCl_3$) δ 8.10 – 7.96 (m, 2H), 7.87 (d, *J* = 8.2 Hz, 2H), 7.72 (d, *J* = 8.3 Hz, 2H), 7.03 – 6.94 (m, 2H), 6.00 (dd, *J* = 11.3, 6.7 Hz, 1H), 4.06 (dd, *J* = 16.8, 6.7 Hz, 1H), 3.96 – 3.89 (m, 1H), 3.87 (s, 3H). ^{13}C NMR (101 MHz, $CDCl_3$) δ 173.3, 165.9, 159.8, 153.0, 130.2, 129.9, 129.3, 126.8, 125.0, 124.3, 123.5, 116.1, 111.9, 71.1, 53.0, 37.3. HRMS (ESI^+): m/z calcd for $C_{19}H_{14}F_3N_3O_3$ M^+: 389.0987; found 389.0980.

3-(4-Methoxyphenyl)-5-(3-(4-nitrophenyl)-4,5-dihydroisoxazol-5-yl)-1,2,4-oxadiazole (**7ad**)

Yellow solid. Yield: 90 mg (49%). M.p. 182-184°C. Rf (33.3% EtOAc/hexane) 0.5. IR (ATR, cm^{-1}): 3091, 3067, 3024 (arom.CH), 2972, 2931, 2837 (aliph.CH), 1609,1598 (C=N), 1584, 1574(C=N), 1514 (NO_2), 1474 (C=C arom), 1426, 1337 (NO_2), 1317, 1257 (C-O), 1226, 1172, 1107, 1026 (C-O), 966, 921, 891, 839 (N-O), 751, 609, 527, 445. ^{1}H NMR (400 MHz, $CDCl_3$) δ 8.34 (dd, *J* = 12.0, 8.6 Hz, 2H), 8.12 – 7.99 (m, 2H), 7.98 – 7.84 (m, 2H), 7.10 – 6.87 (m, 2H), 6.06 (dd, *J* = 10.2, 3.9 Hz, 1H), 4.11 (dd, *J* = 16.5, 6.7 Hz, 1H), 4.01 – 3.65 (m, 4H). ^{13}C NMR (101 MHz, $CDCl_3$) δ 175.6, 168.5, 162.4, 155.0, 149.1, 134.4, 129.4, 128.1, 127.5, 124.4, 124.1, 118.6, 114.5, 74.0, 55.6, 39.6, 29.9. HRMS (ESI^+): m/z calcd for $C_{18}H_{14}N_4O_5$ M^+: 366.0964; found 366.0970.

4-(5-(3-(4-Methoxyphenyl)-1,2,4-oxadiazol-5-yl)-4,5-dihydroisoxazol-3-yl) benzonitrile (**7ae**)

Yellow solid. Yield: 95 mg (54.9%). M.p. 149-151°C. Rf (33.3% EtOAc/hexane) 0.47. IR (ATR, cm^{-1}): 3077, 3036 (arom.CH), 2940, 2842 (aliph.CH), 2224 (C≡N), 1606 (C=N), 1589 (C=N), 1539 (C=C arom), 1474 (C=C arom), 1418, 1344, 1304, 1255 (C-O), 1218, 1195, 1174, 1109 (C-O), 1023, 890, 826 (N-O), 758, 702, 611, 555, 529, 448. ^{1}H NMR (400 MHz, $CDCl_3$) δ 8.00 (d, *J* = 8.6 Hz, 2H), 7.84 (d, *J* = 8.2 Hz, 2H), 7.74 (d, *J* = 8.2 Hz, 2H), 6.98 (d, *J* = 8.6 Hz, 2H), 6.01 (dd, *J* = 11.3, 6.7 Hz, 1H), 4.04 (dd, *J* = 16.8, 6.7 Hz, 1H), 3.96 – 3.75 (m, 4H). ^{13}C NMR (101 MHz, $CDCl_3$) δ 175.5, 168.3, 162.2, 155.1, 132.7, 132.5, 132.4,

129.2, 127.5, 127.1, 118.4, 118.2, 114.3, 114.1, 73.7, 55.4, 39.4. HRMS (ESI$^+$): m/z calcd for $C_{19}H_{14}N_4O_3$ M$^+$: 346.1065; found 346.1076.

5-(3-(4-Bromophenyl)-4,5-dihydroisoxazol-5-yl)-3-phenyl-1,2,4-oxadiazole (**7af**)

White powder solid. Yield: 100 mg (54.1%). M.p. 126-128°C. Rf (33.3% EtOAc/hexane) 0.68. IR (ATR, cm^{-1}): 3043 (arom.CH), 1592 (C=N), 1572 (C=N), 1527, 1491 (C=C arom), 1445, 1400, 1346, 1289 (C-O), 1221, 1167, 1067,1029 (C-O), 1008, 932, 882, 823 (N-O), 750, 691, 538(Br), 449. ^{1}H NMR (400 MHz, $CDCl_3$) δ 8.09 (dd, *J* = 8.1, 1.5 Hz, 2H), 7.65 – 7.57 (m, 4H), 7.56 – 7.46 (m, 3H), 5.99 (dd, *J* = 11.3, 6.6 Hz, 1H), 4.03 (dd, *J* = 16.8, 6.6 Hz, 1H), 3.88 (dd, *J* = 16.8, 11.3 Hz, 1H). ^{13}C NMR (101 MHz, $CDCl_3$) δ 176.2, 168.6, 155.6, 132.2, 131.6, 128.9, 128.5, 127.6, 127.2, 126.1, 125.3, 73.3, 39.8. HRMS (ESI$^+$): m/z calcd for $C_{17}H_{12}BrN_3O_2$ M$^+$: 369.0112; found 369.0082.

5-(3-(4-Fluorophenyl)-4,5-dihydroisoxazol-5-yl)-3-phenyl-1,2,4-oxadiazole (**7ag**)

White crystal solid. Yield: 110 mg (71.1%). M.p. 99-101°C. Rf (33.3% EtOAc/hexane) 0.66. IR (ATR, cm^{-1}): 3075 (arom.CH), 2998, 2953 (aliph.CH), 1602 (C=N), 1593 (C=N), 1570, 1513 (C=C arom), 1446, 1361, 1281 (C-O), 1233, 1162 (C-F), 1116, 1171 (C-O), 1007, 883, 832 (N-O), 762, 691, 603, 541, 456. ^{1}H NMR (400 MHz, $CDCl_3$) δ 8.09 (dd, *J* = 8.0, 1.5 Hz, 2H), 7.80 – 7.67 (m, 2H), 7.58 – 7.41 (m, 3H), 7.23 – 6.90 (m, 2H), 5.97 (dd, *J* = 11.2, 6.6 Hz, 1H), 4.03 (dd, *J* = 16.8, 6.6 Hz, 1H), 3.89 (dd, *J* = 16.8, 11.3 Hz, 1H). ^{13}C NMR (101 MHz, $CDCl_3$) δ 176.3, 168.6, 165.4, 162.9, 155.4, 131.5, 129.2, 129.1, 128.9, 127.5, 126.2, 124.5, 124.5, 116.3, 116.0, 73.1, 40.1. HRMS (ESI$^+$): m/z calcd for $C_{17}H_{13}FN_3O_2$ [M+H]$^+$: 310.0991; found 310.0992.

3-Phenyl-5-(3-(4-(trifluoromethyl)phenyl)-4,5-dihydroisoxazol-5-yl)-1,2,4-oxadiazole (**7ah**)

Cream-white solid. Yield: 140 mg (78 %). M.p. 132-134°C. Rf (33.3% EtOAc/hexane) 0.58. IR (ATR, cm^{-1}): 3061 (arom.CH), 1618, 1601 (C=N), 1584, 1567 (C=N), 1529, 1474 (C=C arom), 1446, 1414, 1363, 1322, 1249 (C-O), 1156, 1110, 1069 (CF_3), 1030, 1004 (C-O), 897, 836 (N-O), 750, 695, 601, 532, 460. ^{1}H NMR (400 MHz, $CDCl_3$) δ 8.13 – 7.99 (m, 2H), 7.86 (d, *J* = 8.2 Hz, 2H), 7.72 (d, *J* = 8.3 Hz, 2H), 7.60 – 7.41 (m, 3H), 6.02 (dd, *J* = 11.4, 6.6 Hz, 1H), 4.07 (dd, *J* = 16.8, 6.6 Hz, 1H), 3.92 (dd, *J* = 16.8, 11.4 Hz, 1H). ^{13}C NMR (101 MHz, $CDCl_3$) δ 176.0, 168.6, 155.4, 132.6, 132.3, 131.6, 131.6, 128.9, 127.5, 127.4, 126.1, 126.0,

125.9, 125.9, 125.9, 73.5, 39.7. HRMS (ESI^+): m/z calcd for $C_{18}H_{12}F_3N_3O_2$ M^+: 359.0881; found 359.0832.

5-(3-(4-Nitrophenyl)-4,5-dihydroisoxazol-5-yl)-3-phenyl-1,2,4-oxadiazole (**7ai**)

Yellow solid. Yield: 70 mg (42%). M.p. 181-183°C. Rf (33.3% EtOAc/hexane) 0.51. IR (ATR, cm^{-1}): 3115, 3091 (arom.CH), 2922, 2849 (aliph.CH), 1597 (C=N), 1580 (C=N), 1508 (NO_2), 1474 (C=C arom), 1446, 1414, 1360 and 1343 (NO_2), 1284 (C-O), 1152, 1115, 1023 (C-O), 963, 935, 892, 848 (N-O), 753, 740, 685, 538, 486, 438. ^1H NMR (400 MHz, $CDCl_3$) δ 8.34 (d, J = 8.7 Hz, 2H), 8.14 – 8.04 (m, 2H), 7.94 (d, J = 8.7 Hz, 2H), 7.60 – 7.41 (m, 3H), 6.07 (dd, J = 11.4, 6.7 Hz, 1H), 4.11 (dd, J = 16.9, 6.7 Hz, 1H), 3.95 (dd, J = 16.8, 11.4 Hz, 1H). ^{13}C NMR (101 MHz, DMSO-d_6) δ 177.7, 168.3, 156.8, 149.0, 134.8, 132.4, 129.9, 128.9, 127.7, 126.2, 124.7, 74.4, 40.7. HRMS (ESI^+): m/z calcd for $C_{17}H_{12}N_4O_4$ M^+: 336.0858; found 336.0831.

4-(5-(3-Phenyl-1,2,4-oxadiazol-5-yl)-4,5-dihydroisoxazol-3-yl)benzonitrile (**7aj**)

Cream-white solid. Yield: 70 mg (44 %). M.p. 161-163°C. Rf (33.3% EtOAc/hexane) 0.56. IR (ATR, cm^{-1}): 3075, 3052 (arom.CH), 2966, 2824, 2853 (aliph.CH), 2222 (C≡N) 1598, 1582 (C=N), 1574 (C=N), 1527, 1474 (C=C arom), 1447, 1407, 1347, 1290, 1256 (C-O), 1219, 1119, 1074 (C-O), 1017, 936, 888, 829 (N-O), 790, 771, 693, 562, 499, 449. ^1H NMR (400 MHz, $CDCl_3$) δ 8.08 (dd, J = 8.1, 1.5 Hz, 2H), 7.86 (d, J = 8.4 Hz, 2H), 7.76 (d, J = 8.4 Hz, 2H), 7.59 – 7.39 (m, 3H), 6.04 (dd, J = 11.4, 6.6 Hz, 1H), 4.06 (dd, J = 16.8, 6.6 Hz, 1H), 3.90 (dd, J = 16.8, 11.4 Hz, 1H). ^{13}C NMR (101 MHz, DMSO-d_6) δ 171.3, 164.0, 150.5, 128.1, 127.9, 127.0, 127.0, 124.4, 124.3, 123.0, 122.9, 121.4, 113.5, 109.6, 69.1, 34.8. HRMS (ESI^+): m/z calcd for $C_{18}H_{12}N_4O_2$ M^+: 316.0960; found 316.0927.

5-(3-(4-Bromophenyl)-4,5-dihydroisoxazol-5-yl)-3-(4-(methylthio)phenyl)-1,2,4-oxadiazole (**7ak**)

White solid. Yield: 130 mg (62%). M.p. 165-167°C. Rf (33.3% EtOAc/hexane) 0.58. IR (ATR, cm^{-1}): 3012 (arom.CH), 2953, 2920, 2852 (aliph.CH), 1589 (C=N), 1557, 1522 (C=N), 1489 (C=C arom), 1465 (C=C arom), 1446, 1399, 1346, 1288 (C-O), 1188, 1114, 1074 (C-O), 1007, 963, 890, 820 (N-O), 760, 694, 533 (Br), 511,450. ^1H NMR (400 MHz, $CDCl_3$) δ 7.99 – 7.89 (m, 2H), 7.61 – 7.48 (m, 4H), 7.30 – 7.19 (m, 2H), 5.94 (dd, J = 11.3, 6.7 Hz, 1H), 3.98 (dd, J = 16.8, 6.7 Hz, 1H), 3.84 (dd, J = 16.8, 11.3 Hz, 1H), 2.50 (s, 3H). ^{13}C NMR (101

MHz, $CDCl_3$) δ 176.1, 168.2, 155.6, 143.4, 132.2, 131.6, 128.5, 128.0, 127.7, 127.1, 125.7, 125.2, 122.3, 73.2, 39.8, 15.0. HRMS (ESI^+): m/z calcd for $C_{18}H_{14}BrN_3O_2S$ M^+: 414.9990; found 414.9970.

5-(3-(4-Fluorophenyl)-4,5-dihydroisoxazol-5-yl)-3-(4-(methylthio)phenyl)-1,2,4-oxadiazole (**7al**)

Yellowish solid. Yield: 80 mg (45.0%). M.p. 138-140°C. Rf (33.3% EtOAc/hexane) 0.57. IR (ATR, cm^{-1}): 3068, 3050 (arom.CH), 2982, 2916, 2855 (aliph.CH), 1602, 1589 (C=N), 1557(C=N), 1514 (C=C arom), 1466 (C=C arom), 1437, 1410, 1350, 1287, 1245 (C-O), 1187 (C-F), 1157, 1115, 1087 (C-O), 1007, 961, 914, 891, 832 (N-O), 762, 697, 605, 538, 512, 460. ^1H NMR (400 MHz, $CDCl_3$) δ 7.98 (d, *J* = 8.4 Hz, 2H), 7.74 (dd, *J* = 8.6, 5.3 Hz, 2H), 7.29 (t, *J* = 8.2 Hz, 2H), 7.13 (dd, *J* = 20.1, 11.5 Hz, 2H), 5.96 (dd, *J* = 11.2, 6.6 Hz, 1H), 4.01 (dd, *J* = 16.7, 6.6 Hz, 1H), 3.88 (dd, *J* = 16.7, 11.3 Hz, 1H), 2.53 (s, 3H). ^{13}C NMR (101 MHz, $CDCl_3$) δ 176.2, 168.2, 165.4, 162.9, 155.4, 143.4, 129.2, 129.1, 127.8, 125.8, 124.5, 122.4, 116.3, 116.0, 73.1, 40.1, 15.0. HRMS (ESI^+): m/z calcd for $C_{18}H_{14}FN_3O_2S$ M^+: 355.0790; found 355.0779.

3-(4-(Methylthio)phenyl)-5-(3-(4-(trifluoromethyl)phenyl)-4,5-dihydro isoxazol-5-yl)-1,2,4-oxadiazole (**7am**)

White-cream solid. Yield: 105 mg (52%). M.p. 139-141°C. Rf (33.3% EtOAc/hexane) 0.6. IR (ATR, cm^{-1}): 2988 (arom CH), 2925, 2855 (aliph.CH), 1593, 1558 (C=N), 1521 (C=C arom), 1470 (C=C arom), 1435, 1411, 1319, 1252 (C-O), 1166, 1141, 1111 (CF_3), 1072, 1034 (C-O), 1007, 968, 904, 833 (N-O), 761, 691, 596, 531, 506, 461. ^1H NMR (400 MHz, $CDCl_3$) δ 7.97 (d, *J* = 8.5 Hz, 2H), 7.85 (d, *J* = 8.2 Hz, 2H), 7.71 (d, *J* = 8.3 Hz, 2H), 7.30 (d, *J* = 8.5 Hz, 2H), 6.00 (dd, *J* = 11.3, 6.7 Hz, 1H), 4.05 (dd, *J* = 16.8, 6.7 Hz, 1H), 3.90 (dd, *J* = 16.8, 11.4 Hz, 1H), 2.52 (s, 3H). ^{13}C NMR (101 MHz, $CDCl_3$) δ 175.9, 168.6, 155.4, 143.5, 132.6, 132.3, 131.6, 127.8, 127.4, 125.9, 125.7, 122.3, 73.5, 39.6, 15.0. HRMS (ESI^+): m/z calcd for $C_{19}H_{13}F_3N_3O_2S$ $[M-H]^+$: 404.0680; found 404.0668.

3-(4-(Methylthio)phenyl)-5-(3-(4-nitrophenyl)-4,5-dihydroisoxazol-5-yl)-1,2,4-oxadiazole (**7an**)

Yellowish solid. Yield: 30 mg (16%). M.p. 154-156°C. Rf (33.3% EtOAc/hexane) 0.5. IR (ATR, cm^{-1}): 3113, 3089, 3064 (arom.CH), 2956, 2921, 2850 (aliph.CH), 1597, 1583 (C=N),

1558 (C=N), 1517 (NO_2), 1474 (C=C arom), 1437, 1408, 1340 (NO_2), 1225 (C-O), 1189, 1115, 1089 (C-O), 1013, 952, 913, 843 (N-O), 779, 751, 690, 506, 458. ^{1}H NMR (400 MHz, $CDCl_3$) δ 8.33 (d, *J* = 8.9 Hz, 2H), 8.01 (dd, *J* = 21.0, 8.7 Hz, 2H), 7.91 (dd, *J* = 19.7, 8.8 Hz, 2H), 7.32 (d, *J* = 8.5 Hz, 2H), 6.05 (dd, *J* = 11.4, 6.7 Hz, 1H), 4.09 (dd, *J* = 16.8, 6.7 Hz, 1H), 3.94 (dd, *J* = 16.8, 11.4 Hz, 1H), 2.54 (s, 3H). ^{13}C NMR (101 MHz, DMSO- d_6) δ 177.4, 168.8, 167.9, 156.7, 148.9, 143.9, 134.6, 128.8, 128.2, 127.9, 126.2, 124.6, 124.5, 122.1, 74.3, 41.8, 14.5. HRMS (ESI$^+$): m/z calcd for $C_{18}H_{15}N_4O_4S$ $[M+H]^+$: 383.0814; found 383.0815.

4-(5-(3-(4-(Methylthio)phenyl)-1,2,4-oxadiazol-5-yl)-4,5-dihydroisoxazol-3-yl) benzonitrile (**7ao**)

Yellow solid. Yield: 40 mg (22%). M.p. 129-131°C. Rf (33.3% EtOAc/hexane) 0.47. IR (ATR, cm^{-1}): 3063, 2995 (arom.CH), 2917, 2869, 2790 (aliph.CH), 2239 (C≡N), 1584, 1556 (C=N), 1508 (C=C arom), 1466 (C=C arom), 1438, 1408, 1352, 1311, 1281 (C-O), 1187, 1114, 1086 (C-O), 1004, 957, 908, 890, 842 (N-O), 757, 696, 561, 503, 455. ^{1}H NMR (400 MHz, $CDCl_3$) δ 7.94 (d, *J* = 8.5 Hz, 2H), 7.82 (d, *J* = 8.4 Hz, 2H), 7.72 (d, *J* = 8.4 Hz, 2H), 7.31 – 7.22 (m, 2H), 6.00 (dd, *J* = 11.4, 6.7 Hz, 1H), 4.02 (dd, *J* = 16.8, 6.7 Hz, 1H), 3.87 (dd, *J* = 16.8, 11.4 Hz, 1H), 2.50 (s, 3H). ^{13}C NMR (101 MHz, $CDCl_3$) δ 175.7, 168.2, 155.1, 143.6, 132.6, 132.4, 127.7, 127.5, 125.7, 122.2, 118.1, 114.1, 73.7, 39.3, 15.0. HRMS (ESI$^+$): m/z calcd for $C_{19}H_{14}N_4O_2S$ M^+: 362.0837; found 362.0820.

4-(5-(3-(4-Bromophenyl)-4,5-dihydroisoxazol-5-yl)-1,2,4-oxadiazol-3-yl)-*N*,*N*-dimethylaniline (**7ap**)

Yellowish semi-solid. Yield: 200 mg (97%). M.p. 131-133°C. Rf (33.3% EtOAc/hexane) 0.69. IR (ATR, cm^{-1}): 2981 (arom.CH), 2943, 2870, 2838, 2789 (aliph.CH), 1605 (C=N), 158 (C=N), 1553 (C=C arom), 1480 (C=C arom), 1467, 1432, 1398, 1322, 1269 (C-O), 1212, 1168, 1137, 1069 (C-O), 1008, 947, 887, 832 (N-O), 817, 755, 717, 609, 538 (Br), 446. ^{1}H NMR (400 MHz, $CDCl_3$) δ 8.04 – 7.70 (m, 2H), 7.56 – 7.40 (m, 4H), 7.31 – 6.89 (m, 2H), 5.85 (dd, *J* = 11.3, 6.7 Hz, 1H), 3.91 (dd, *J* = 16.8, 6.7 Hz, 1H), 3.77 (dd, *J* = 16.8, 11.3 Hz, 1H), 2.81 (s, 6H). ^{13}C NMR (101 MHz, $CDCl_3$) δ 176.0, 167.4, 155.5, 152.8, 132.1, 130.0, 128.4, 127.3, 127.0, 126.6, 125.1, 120.0, 119.6, 73.1, 43.2, 39.7. HRMS (ESI$^+$): m/z calcd for $C_{19}H_{17}BrN_4O_2$ M^+: 412.0534; found 412.2582.

4-(5-(3-(4-Fluorophenyl)-4,5-dihydroisoxazol-5-yl)-1,2,4-oxadiazol-3-yl)-*N*,*N*-dimethylaniline (**7aq**)

Yellowish semi-solid. Yield: 160 mg (91%). M.p. 104-106°C. Rf (33.3% EtOAc/hexane) 0.67. IR (ATR, cm^{-1}): 3077 (arom.CH), 2946, 2839, 2788 (aliph.CH), 1600 (C=N), 1584, 1551 (C=N), 1509 (C=C arom), 1481 (C=C arom), 1427, 1393, 1337, 1213 (C-O), 1157, 1137, 1098 (C-F) 1117, 1098, 1054 (C-O), 947, 879, 847 (N-O), 760, 686, 599, 544, 461. ^{1}H NMR (400 MHz, $CDCl_3$) δ 8.10 – 7.80 (m, 2H), 7.71 (dd, *J* = 8.8, 5.3 Hz, 2H), 7.12 (ddd, *J* = 28.9, 25.0, 16.2 Hz, 4H), 5.92 (dd, *J* = 11.3, 6.6 Hz, 1H), 3.99 (dd, *J* = 16.8, 6.6 Hz, 1H), 3.85 (dd, *J* = 16.8, 11.3 Hz, 1H), 2.88 (s, 6H). ^{13}C NMR (101 MHz, $CDCl_3$) δ 176.2, 167.5, 165.4, 162.9, 155.4, 152.8, 130.0, 129.1, 129.1, 127.4, 126.6, 124.5, 124.4, 119.7, 116.2, 116.0, 73.1, 43.3, 40.0. HRMS (ESI^+): m/z calcd for $C_{19}H_{17}FN_4O_2$ M^+: 352.1335; found 352.1324.

N,*N*-Dimethyl-4-(5-(3-(4-(trifluoromethyl)phenyl)-4,5-dihydroisoxazol-5-yl)-1,2,4-oxadiazol-3-yl) aniline (**7ar**)

Yellowish semi-solid. Yield: 150 mg (75%). M.p. 93-95°C. Rf (33.3% EtOAc/hexane) 0.70. IR (ATR, cm^{-1}): 2988 (arom.CH), 2945, 2873, 2843, 2797 (aliph.CH), 1603 (C=N), 1590 (C=N), 1556 (C=C arom), 1483 (C=C arom), 1432, 1412, 1320, 1268 (C-O), 1160, 1111 (C-F) 1067, 1031 (C-O), 946, 894, 842 (N-O), 809, 775, 682, 583, 455. ^{1}H NMR (400 MHz, $CDCl_3$) δ 7.92 (ddd, *J* = 36.7, 14.7, 5.1 Hz, 4H), 7.71 (d, *J* = 8.3 Hz, 2H), 7.39 – 7.01 (m, 2H), 5.99 (dd, *J* = 11.4, 6.6 Hz, 1H), 4.05 (dd, *J* = 16.8, 6.6 Hz, 1H), 3.90 (dd, *J* = 16.8, 11.4 Hz, 1H), 2.90 (s, 6H). ^{13}C NMR (101 MHz, $CDCl_3$) δ 176.0, 167.5, 155.4, 152.8, 132.6, 132.3, 131.6, 131.6, 130.1, 127.4, 127.4, 126.7, 126.0, 125.9, 125.9, 125.8, 125.0, 122.3, 119.8, 73.4, 43.3, 39.6. HRMS (ESI^+): m/z calcd for $C_{20}H_{17}F_3N_5O_2$ $[M+14]^+$: 416.1334; found 416.1320.

N,*N*-Dimethyl-4-(5-(3-(4-nitrophenyl)-4,5-dihydroisoxazol-5-yl)-1,2,4-oxadiazol-3-yl) aniline (**7as**)

Yellow powder solid. Yield: 110 mg (57%). M.p. 167-169°C. Rf (33.3% EtOAc/hexane) 0.39. IR (ATR, cm^{-1}): 3078-3055 (arom.CH), 2955, 2843, 2794 (aliph.CH), 1603 (C=N), 1581, 1553 (C=N), 1520 (NO_2), 1478 (C=C arom), 1410, 1337 and 1317 (NO_2), 1269 (C-O), 1170, 1148, 1044 (C-O), 1011, 885, 847 (N-O), 760, 685, 610, 539, 439. ^{1}H NMR (400 MHz, $CDCl_3$) δ 8.33 (d, *J* = 8.8 Hz, H), 8.15 – 7.78 (m, 4H), 7.48 – 7.02 (m, 2H), 6.05 (dd, *J* = 11.4, 6.7 Hz, 1H), 4.09 (dd, *J* = 16.9, 6.7 Hz, 1H), 3.94 (dd, *J* = 16.9, 11.4 Hz, 1H), 2.94 (s, 6H). ^{13}C NMR (101 MHz, $CDCl_3$) δ 175.7, 167.5, 154.9, 148.9, 134.2, 130.1, 127.9, 127.4, 126.7,

124.2, 120.0, 120.0, 73.8, 43.4, 39.4. HRMS (ESI^+): m/z calcd for $C_{19}H_{17}N_5O_4$ M^+: 379.1280; found 379.1259.

4-(5-(3-(4-(Dimethylamino)phenyl)-1,2,4-oxadiazol-5-yl)-4,5-dihydroisoxazol-3-yl) benzonitrile (**7at**)

Cream-white powder solid. Yield: 100 mg (56%). M.p. 143-145°C. Rf (33.3% EtOAc/hexane) 0.40. IR (ATR, cm^{-1}): 2939 (arom.CH), 2875, 2849, 2799 (aliph.CH), 2227 (C≡N), 1604 (C=N), 1589 (C=N), 1549 (C=C arom), 1484 (C=C arom), 1433, 1392, 1347, 1323, 1258 (C-O), 1168, 1130 (C-O), 1058, 939, 842 (N-O), 756, 682, 564, 461. ^{1}H NMR (400 MHz, $CDCl_3$) δ 8.10 – 7.81 (m, 4H), 7.78 – 7.71 (m, 2H), 7.36 – 7.00 (m, 2H), 6.01 (dd, *J* = 11.4, 6.7 Hz, 1H), 4.04 (dd, *J* = 16.8, 6.7 Hz, 1H), 3.89 (dd, *J* = 16.9, 11.4 Hz, 1H), 2.91 (s, 6H). ^{13}C NMR (101 MHz, $CDCl_3$) δ 175.8, 167.5, 155.2, 153.0, 132.7, 132.5, 130.1, 127.6, 127.4, 126.7, 119.9, 119.7, 118.1, 114.2, 73.7, 43.3, 39.4. HRMS (ESI^+): m/z calcd for $C_{20}H_{17}N_5O_2$ M^+: 359.1382; found 359.1357.

5-(3-(4-Bromophenyl)-4,5-dihydroisoxazol-5-yl)-3-(p-tolyl)-1,2,4-oxadiazole (**7au**)

White solid. Yield: 130 mg (68%). M.p. 137-139°C. Rf (33.3% EtOAc/hexane) 0.78. IR (ATR, cm^{-1}): 3030 (arom.CH), 2917 (aliph.CH), 1615, 1588 (C=N), 1562 (C=N), 1539 (C=C arom), 1481 (C=C arom), 1400, 1347, 1282 (C-O), 1160, 1108, 1068 (C-O), 1006, 943, 901, 822 (N-O), 749, 691, 540 (Br), 503, 446. ^{1}H NMR (400 MHz, $CDCl_3$) δ 7.96 (d, *J* = 8.1 Hz, 2H), 7.78 – 7.41 (m, 4H), 7.28 (d, *J* = 8.3 Hz, 2H), 5.96 (dd, *J* = 11.3, 6.7 Hz, 1H), 4.01 (dd, *J* = 16.8, 6.7 Hz, 1H), 3.86 (dd, *J* = 16.8, 11.3 Hz, 1H), 2.42 (s, 3H). ^{13}C NMR (101 MHz, $CDCl_3$) δ 176.0, 168.6, 155.6, 141.9, 132.2, 129.6, 128.5, 127.5, 127.2, 125.2, 123.30, 73.3, 39.8, 21.6. HRMS (ESI^+): m/z calcd for $C_{18}H_{14}BrN_3O_2$ M^+: 383.0269; found 383.0253.

5-(3-(4-Fluorophenyl)-4,5-dihydroisoxazol-5-yl)-3-(*p*-tolyl)-1,2,4-oxadiazole (**7av**)

White solid. Yield: 100 mg (62%). M.p. 113-115°C. Rf (33.3% EtOAc/hexane) 0.71. IR (ATR, cm^{-1}): 3064 (arom.CH), 2925 (aliph.CH), 1596, 1573 (C=N), 1537 (C=N), 1513 (C=C arom), 1481 (C=C arom), 1434, 1409, 1349, 1301, 1227 (C-O), 1163 (C-F), 1108, 1066, 1020 (C-O), 984, 934, 846 (N-O), 744, 600, 546, 507, 464. ^{1}H NMR (400 MHz, $CDCl_3$) δ 7.96 (d, *J* = 8.1 Hz, 2H), 7.73 (dd, *J* = 8.8, 5.3 Hz, 2H), 7.28 (d, *J* = 8.3 Hz, 2H), 7.14 (t, *J* = 8.6 Hz, 2H), 5.95 (dd, *J* = 11.3, 6.6 Hz, 1H), 4.01 (dd, *J* = 16.8, 6.6 Hz, 1H), 3.86 (dd, *J* = 16.8, 11.3 Hz, 1H), 2.41 (s, 3H). ^{13}C NMR (101 MHz, $CDCl_3$) δ 176.1, 168.5, 165.4, 162.9, 155.4,

141.9, 129.6, 129.2, 129.1, 127.7, 124.5, 124.5, 123.3, 116.2, 116.0, 73.2, 40.1, 21.6. HRMS (ESI$^+$): m/z calcd for $C_{18}H_{14}FN_3O_2$ M$^+$: 323.1070; found 323.1060.

3-(*p*-Tolyl)-5-(3-(4-(trifluoromethyl)phenyl)-4,5-dihydroisoxazol-5-yl)-1,2,4-oxadiazole (**7aw**)

Yellow solid. Yield: 140 mg (75%). M.p. 135-137°C. Rf (33.3% EtOAc/hexane) 0.88. IR (ATR, cm^{-1}): 2928 (arom CH), 2869 (aliph.CH), 1616, 1596 (C=N), 1565 (C=N), 1540 (C=C arom), 1480 (C=C arom), 1412, 1319, 1242 (C-O), 1166, 1123 (C-F), 1068, 1013, 982 (C-O), 894, 832 (N-O), 761, 694, 598, 534, 505, 463. ^{1}H NMR (400 MHz, $CDCl_3$) δ 7.96 (d, *J* = 8.2 Hz, 2H), 7.86 (d, *J* = 8.2 Hz, 2H), 7.71 (d, *J* = 8.3 Hz, 2H), 7.28 (d, *J* = 8.0 Hz, 2H), 6.01 (dd, *J* = 11.4, 6.7 Hz, 1H), 4.06 (dd, *J* = 16.8, 6.7 Hz, 1H), 3.90 (dd, *J* = 16.8, 11.4 Hz, 1H), 2.41 (s, 3H). ^{13}C NMR (101 MHz, $CDCl_3$) δ 175.9, 168.6, 155.4, 142.0, 132.6, 132.2, 131.7, 129.6, 127.5, 127.4, 126.0, 125.9, 125.9, 125.8, 123.2, 73.5, 39.6, 21.6. HRMS (ESI$^+$): m/z calcd for $C_{19}H_{14}F_3N_3O_2$ M$^+$: 373.1038; found 373.1010.

5-(3-(4-Nitrophenyl)-4,5-dihydroisoxazol-5-yl)-3-(p-tolyl)-1,2,4-oxadiazole (**7ax**)

Yellowish solid. Yield: 90 mg (51%). M.p. 173-175°C. Rf (33.3% EtOAc/hexane) 0.67. IR (ATR, cm^{-1}): 3124, 3093, 3069 (arom.CH), 2924, 2849 (aliph.CH), 1612, 1585 (C=N), 1567 (C=N), 1517 (NO_2), 1478 (C=C arom), 1438, 1410, 1381, 1338 (NO_2), 1253 (C-O), 1225, 1179, 1113 (C-O), 962, 923, 899, 845 (N-O), 750, 688, 603, 506, 448. ^{1}H-NMR (400 MHz, $CDCl_3$). ^{1}H NMR (400 MHz, $CDCl_3$) δ 8.29 (d, *J* = 8.7 Hz, 2H), 7.93 (d, *J* = 8.1 Hz, 2H), 7.89 (d, *J* = 8.7 Hz, 2H), 7.26 (d, *J* = 8.1 Hz, 2H), 6.02 (dd, *J* = 11.4, 6.7 Hz, 1H), 4.06 (dd, *J* = 16.8, 6.7 Hz, 1H), 3.90 (dd, *J* = 16.8, 11.4 Hz, 1H), 2.39 (s, 3H). ^{13}C NMR (101 MHz, $CDCl_3$) δ 175.7, 168.7, 155.0, 149.0, 142.1, 134.4, 129.8, 128.0, 127.6, 124.3, 123.3, 73.9, 39.5, 21.7. HRMS (ESI$^+$): m/z calcd for $C_{18}H_{14}N_4O_4$ M$^+$: 350.1015; found 350.1000.

4-(5-(3-(*p*-Tolyl)-1,2,4-oxadiazol-5-yl)-4,5-dihydroisoxazol-3-yl) benzonitrile (**7ay**)

White solid. Yield: 90 mg (54%). M.p. 157-159°C. Rf (33.3% EtOAc/hexane) 0.56. IR (ATR, cm^{-1}): 2955 (arom CH), 2926, 2856 (aliph.CH), 2224 (C≡N), 1611, 1590 (C=N), 1563(C=N), 1538, 1481(C=C arom), 1413, 1356, 1323, 1289 (C-O), 1110, 1030 (C-O), 933, 880, 835(N-O), 749, 558, 502, 454. ^{1}H NMR (400 MHz, $CDCl_3$) δ 7.96 (d, *J* = 8.0 Hz, 2H), 7.85 (d, *J* = 8.2 Hz, 2H), 7.75 (d, *J* = 8.3 Hz, 2H), 7.29 (d, *J* = 7.8 Hz, 2H), 6.03 (dd, *J* = 11.3, 6.7 Hz, 1H), 4.05 (dd, *J* = 16.8, 6.7 Hz, 1H), 3.90 (dd, *J* = 16.8, 11.4 Hz, 1H), 2.42 (s, 3H). ^{13}C NMR

(101 MHz, $CDCl_3$) δ 175.6, 168.6, 155.1, 142.0, 132.7, 132.5, 129.7, 127.6, 127.5, 123.2, 118.1, 114.1, 73.8, 39.4, 21.6. HRMS (ESI^+): m/z calcd for $C_{19}H_{15}N_4O_2$ $[M+H]^+$: 331.1195; found 331.1195.

In silico studies

Molecular docking studies

First, the co-crystallized structure was downloaded from the Protein Data Bank (PDB ID: 5N2S)[4]. Then, the ligand in the protein was removed using Autodock Vina 4.2.6 so that our own ligands could enter it. Then, the surrounding solvent was removed. Finally, the protein was prepared by performing the necessary charge assignments and optimization processes. Additionally, the ligands to be bound were appropriately optimized with Open Babel using MMFF94 (Merck Molecular Force Field). Docking simulations were performed using AutoDock Vina 4.2.6 with a grid box centered at coordinates X: 97.8195, Y: 134.183, Z: 46.1548 and dimensions (Angstrom) X: 63.6714, Y: 54.8516, Z: 103.1327. This large grid facilitated the exploration of alternative binding conformations.

ADMET studies

A computational analysis was performed on the 14 top-scoring compounds (**7d**, **7j**, **7n**, **7r**, **7t**, **7u**, **7w**, **7x**, **7ah**, **7ar**, **7av**, **7aw**, **7ax**, **7ay**) to predict their absorption, distribution, metabolism, excretion and toxicity (ADMET) using SwissADME online tool[5].

References

(1) Aakeröy, C. B.; Sinha, A. S.; Epa, K. N.; Spartz, C. L.; Desper, J. A Versatile and Green Mechanochemical Route for Aldehyde–Oxime Conversions. *Chemical Communications* **2012**, *48* (92), 11289–11291.

(2) Baláž, M.; Kudličková, Z.; Vilková, M.; Imrich, J.; Balážová, L.; Daneu, N. Mechanochemical Synthesis and Isomerization of N-Substituted Indole-3-Carboxaldehyde Oximes. *Molecules* **2019**, *24* (18), 3347.

(3) Pitasse-Santos, P.; Sueth-Santiago, V.; Lima, M. E. 1, 2, 4-and 1, 3, 4-Oxadiazoles as Scaffolds in the Development of Antiparasitic Agents. *Journal of the Brazilian Chemical Society* **2018**, *29* (3), 435–456.

(4) Cheng, R. K.; Segala, E.; Robertson, N.; Deflorian, F.; Doré, A. S.; Errey, J. C.; Fiez-Vandal, C.; Marshall, F. H.; Cooke, R. M. Structures of Human A1 and A2A Adenosine Receptors with Xanthines Reveal Determinants of Selectivity. *Structure* **2017**, *25* (8), 1275–1285. https://doi.org/doi:%20https://doi.org/10.1016/j.str.2017.06.012.

(5) Daina, A.; Michielin, O.; Zoete, V. SwissADME: A Free Web Tool to Evaluate Pharmacokinetics, Drug-Likeness and Medicinal Chemistry Friendliness of Small Molecules. *Scientific reports* **2017**, *7* (1), 42717.

Spectra (IR, NMR, HRMS) for the precursors (2,6) and products (7)

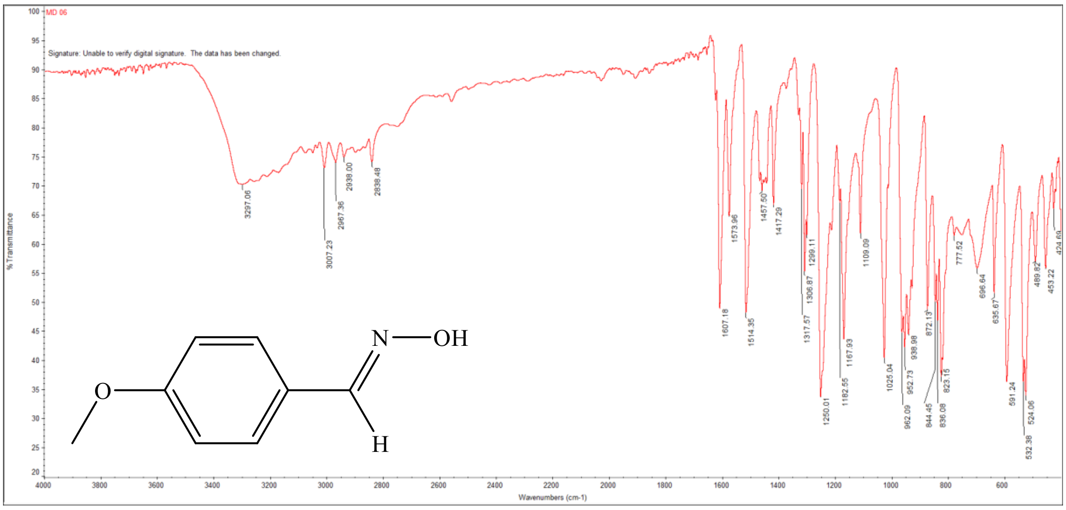

Figure S1. IR Spectrum of compound **2a**

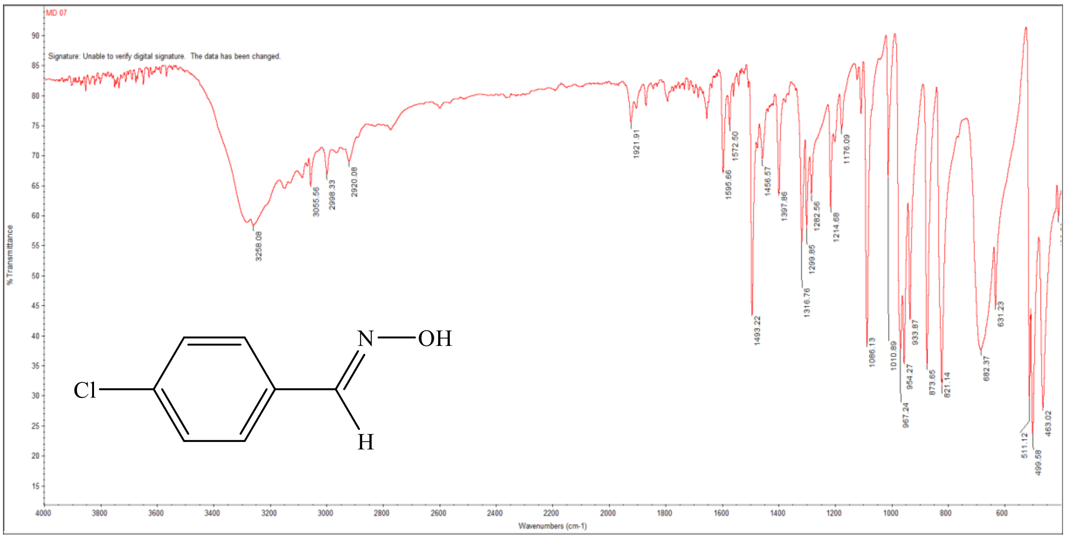

Figure S2. IR Spectrum of compound **2b**

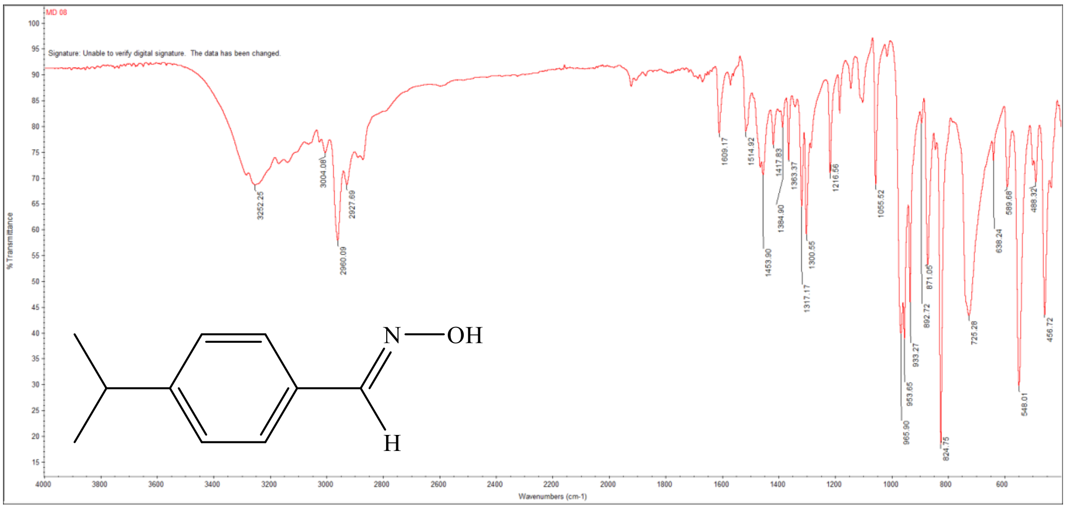

Figure S3. IR Spectrum of compound **2c**

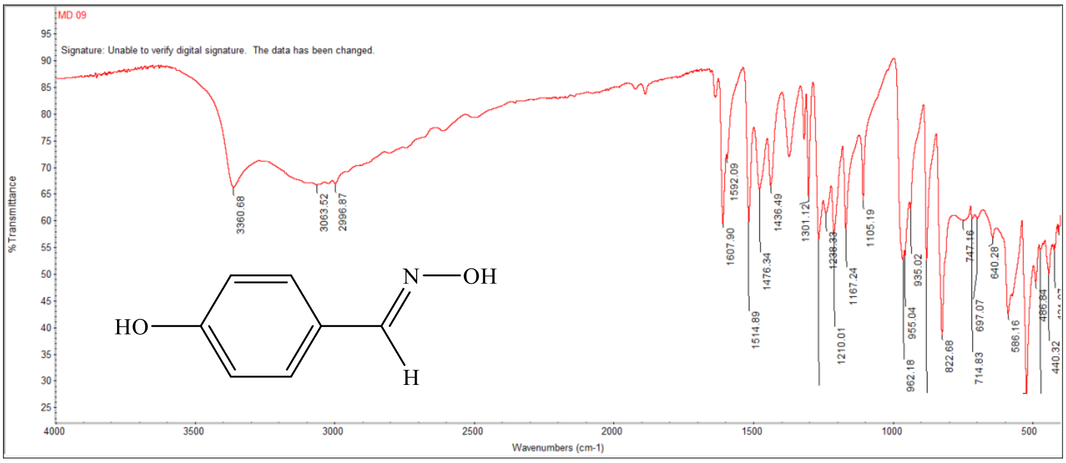

Figure S4. IR Spectrum of compound **2d**

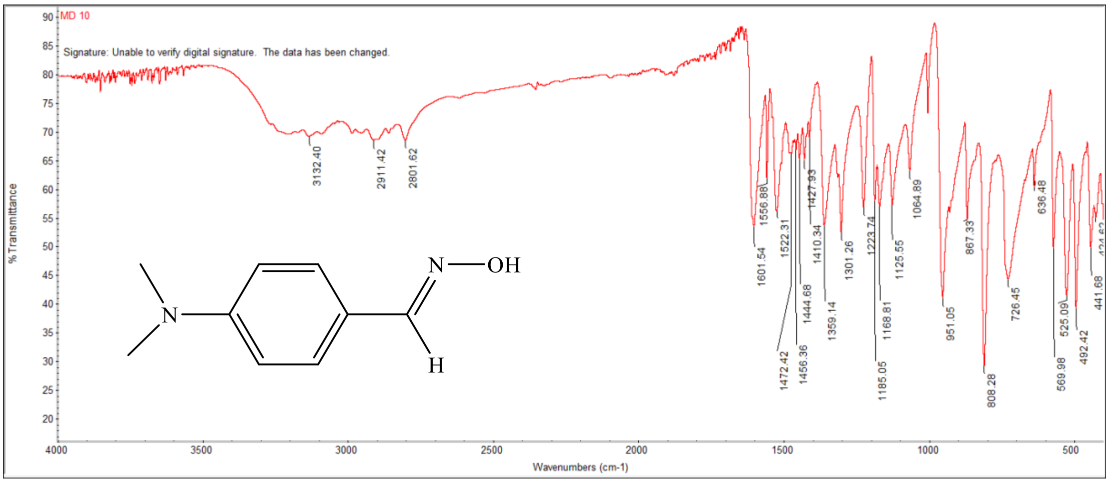

Figure S5. IR Spectrum of compound **2e**

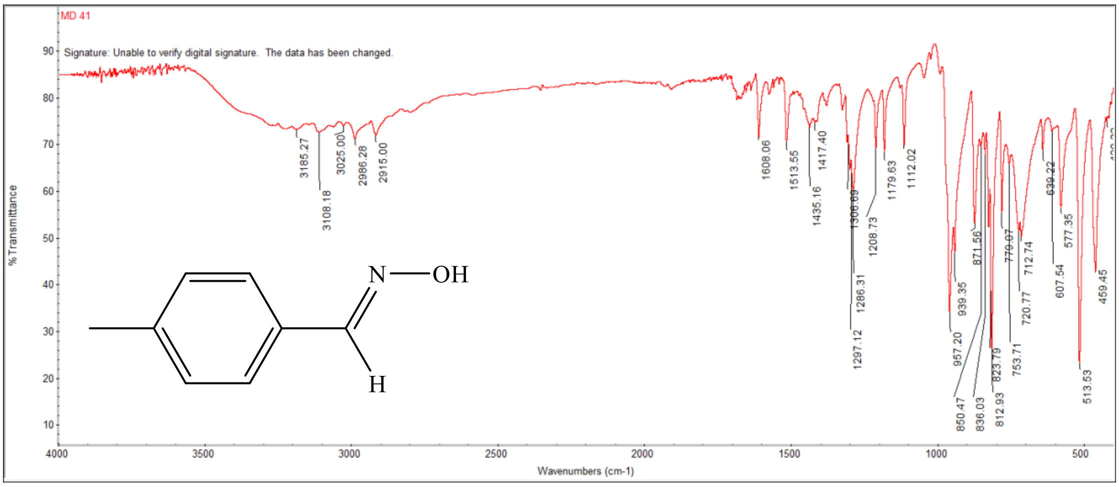

Figure S6. IR Spectrum of compound **2f**

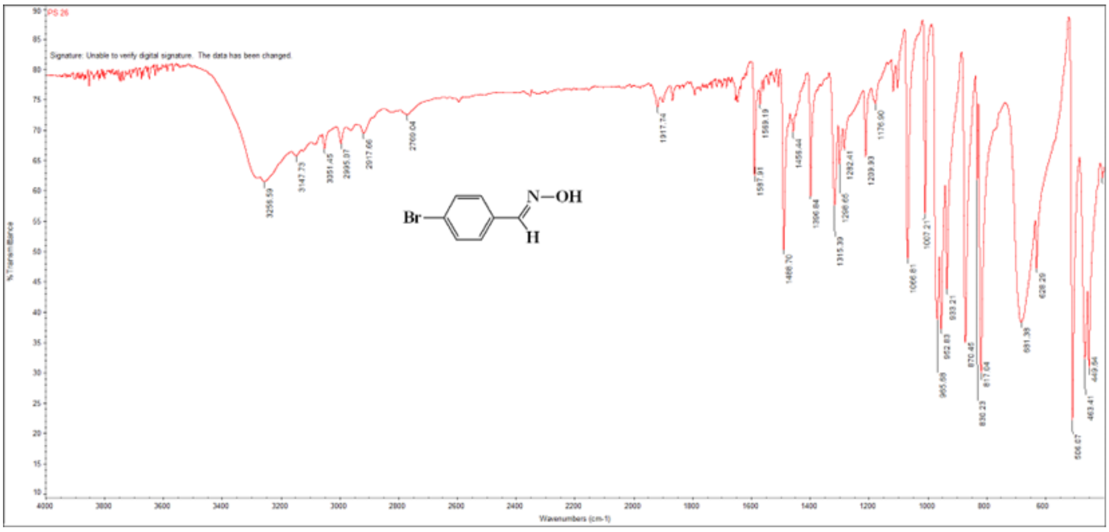

Figure S7. IR Spectrum of compound **2g**

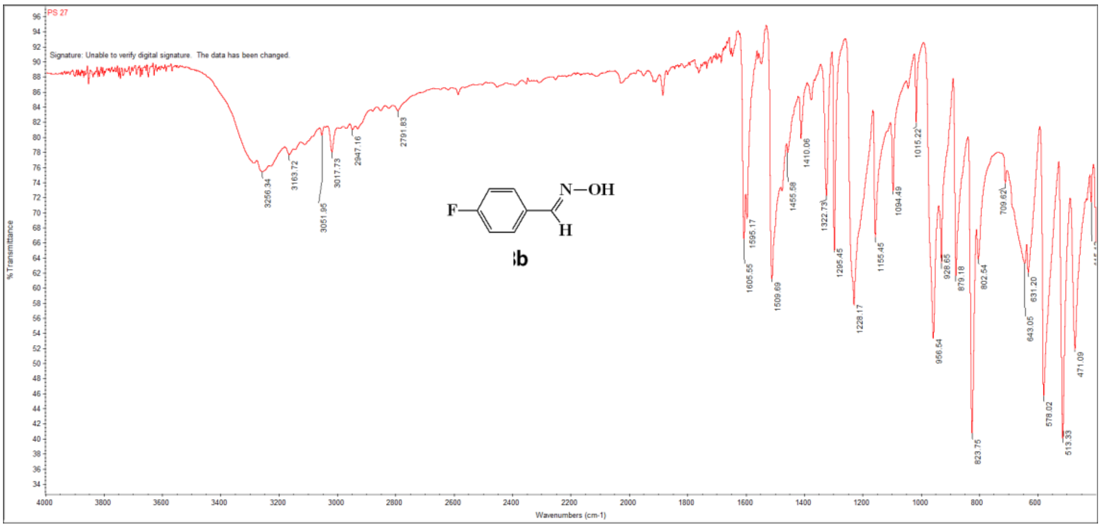

Figure S8. IR Spectrum of compound **2h**

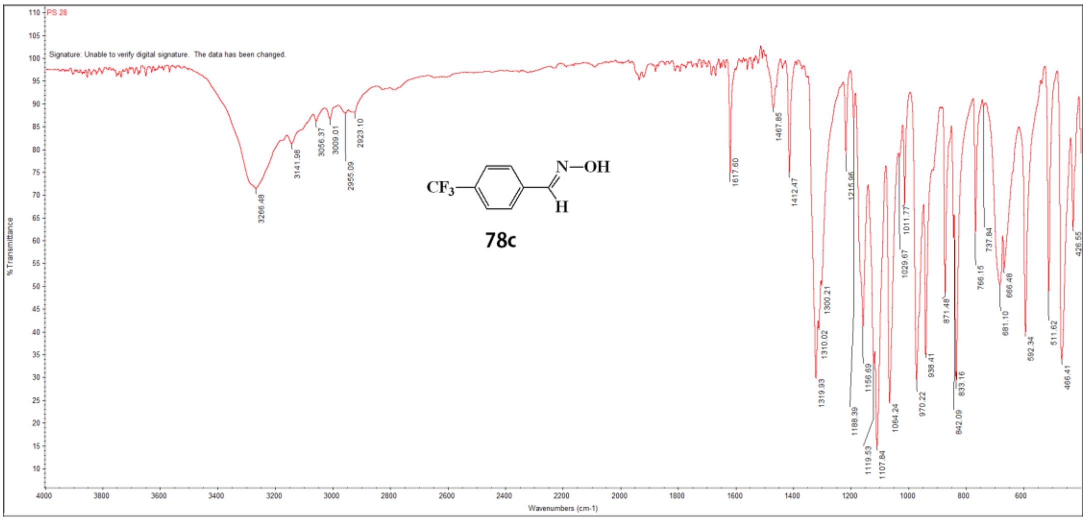

Figure S9. IR Spectrum of compound **2i**

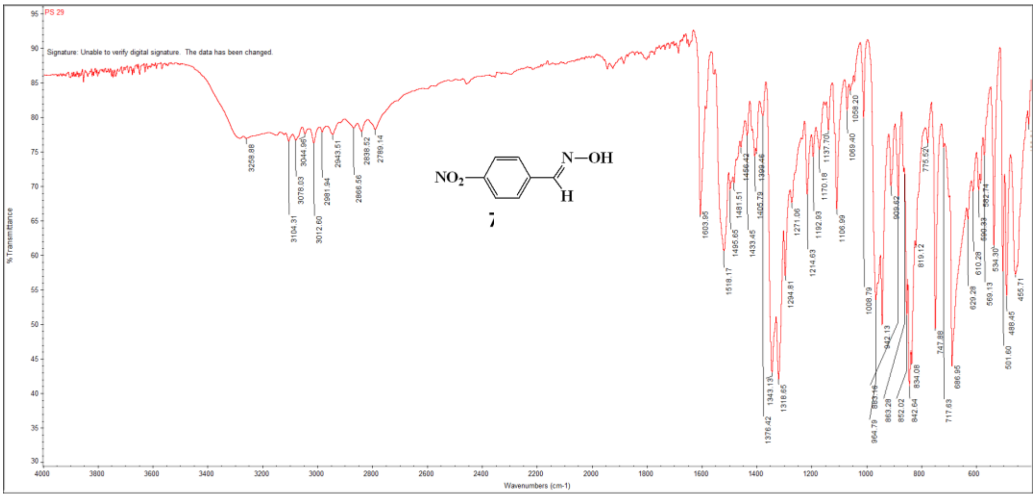

Figure S10. IR Spectrum of compound **2j**

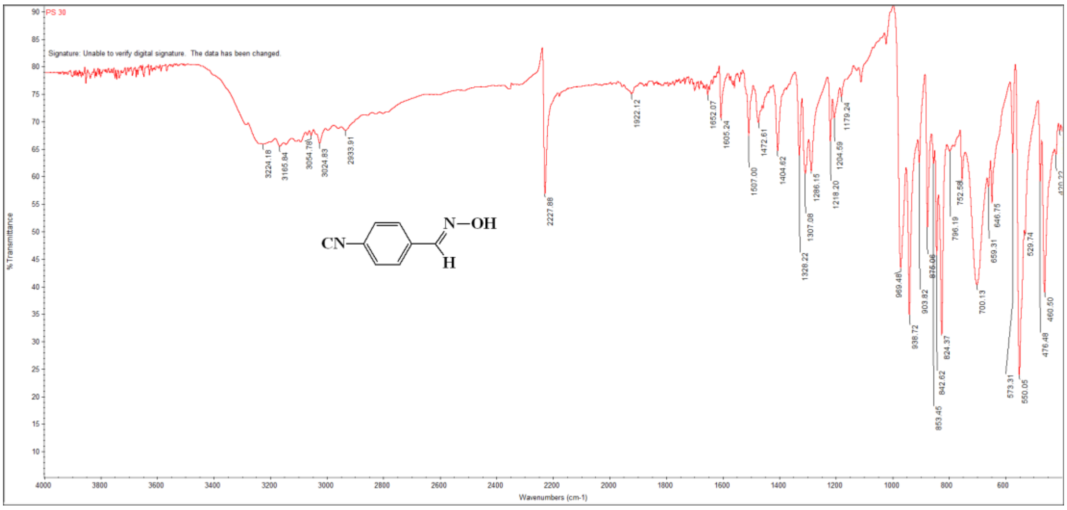

Figure S11. IR Spectrum of compound **2k**

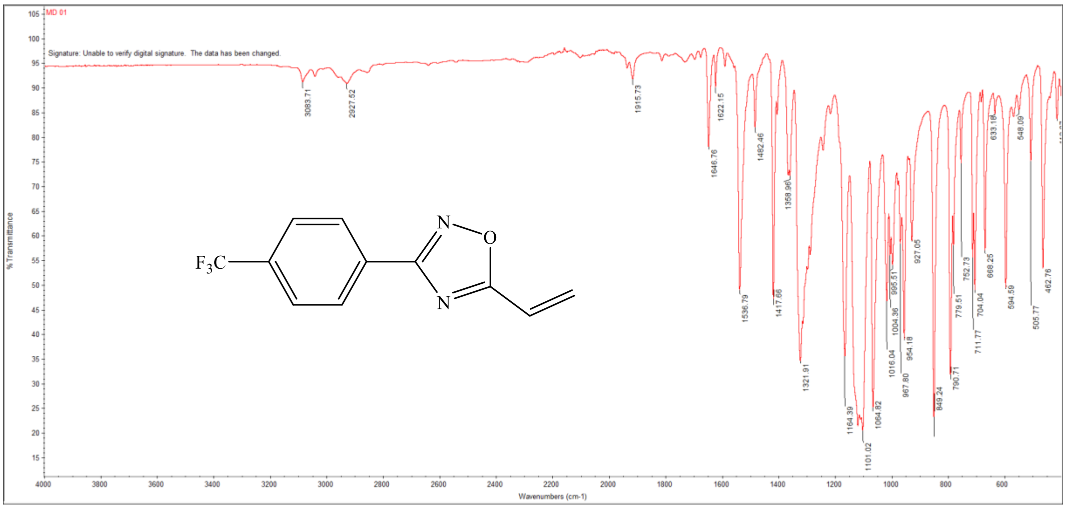

Figure S12. IR Spectra of compound **6a**

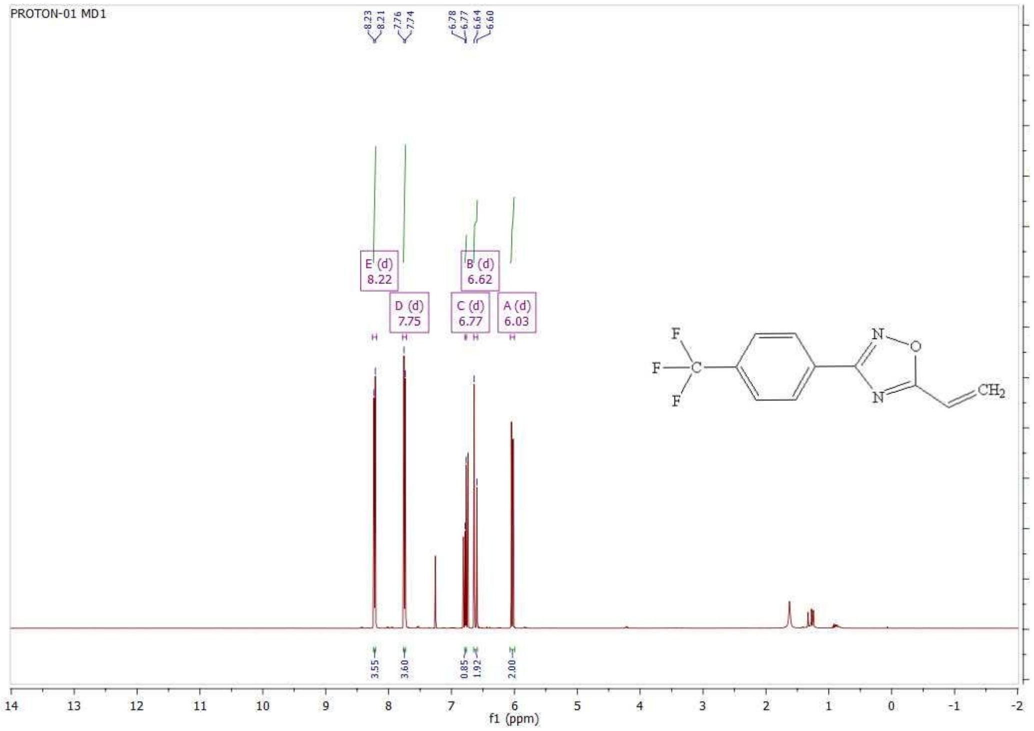

Figure S13. ^{1}H-NMR Spectrum of compound **6a**

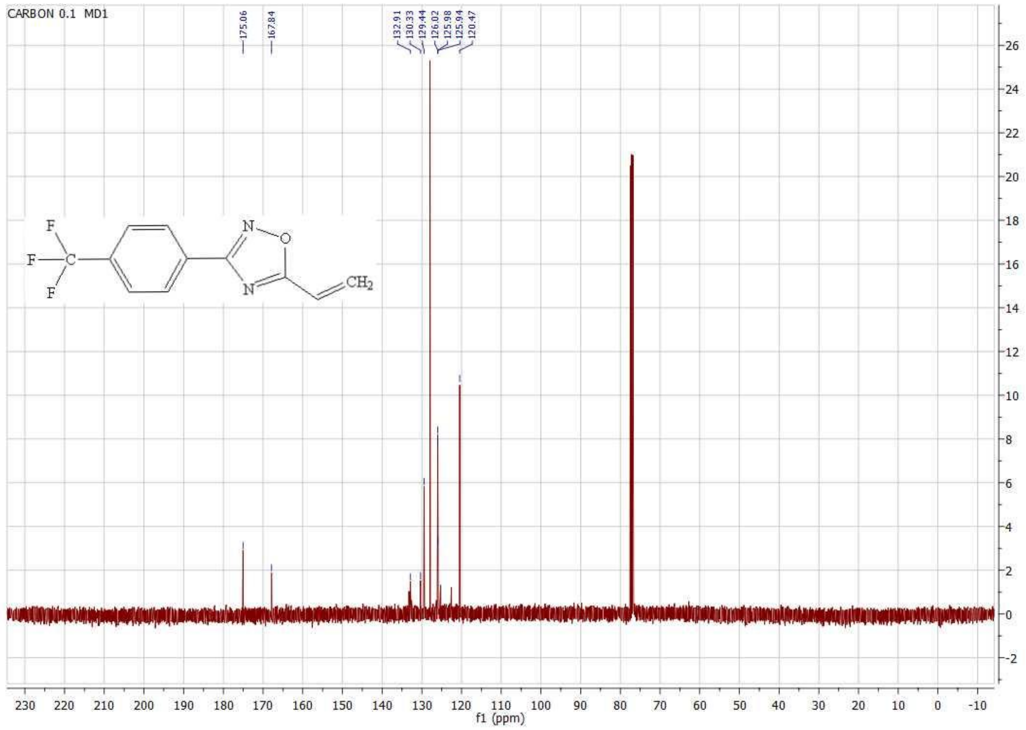

Figure S14. ^{13}C-NMR Spectrum of compound **6a**

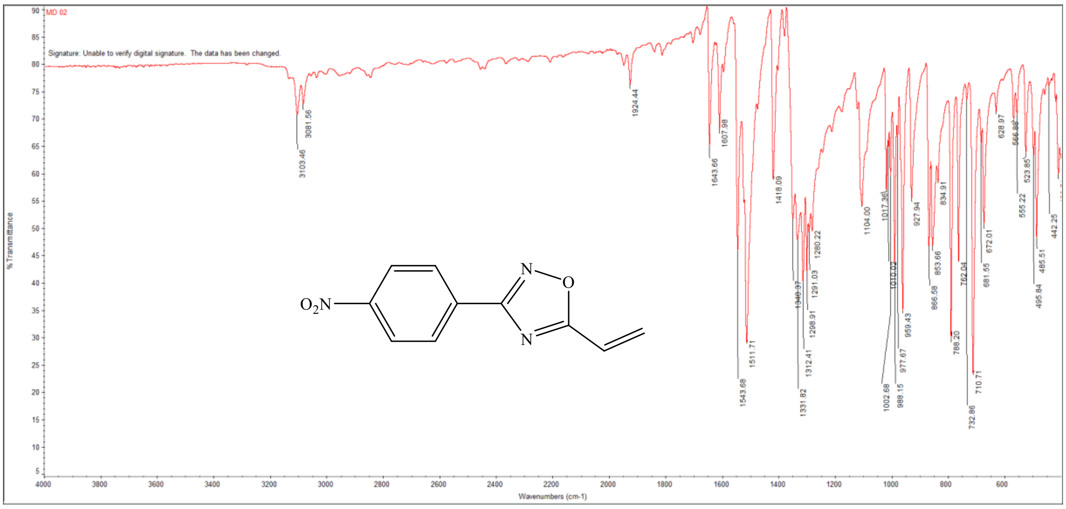

Figure S15. IR Spectrum of compound **6b**

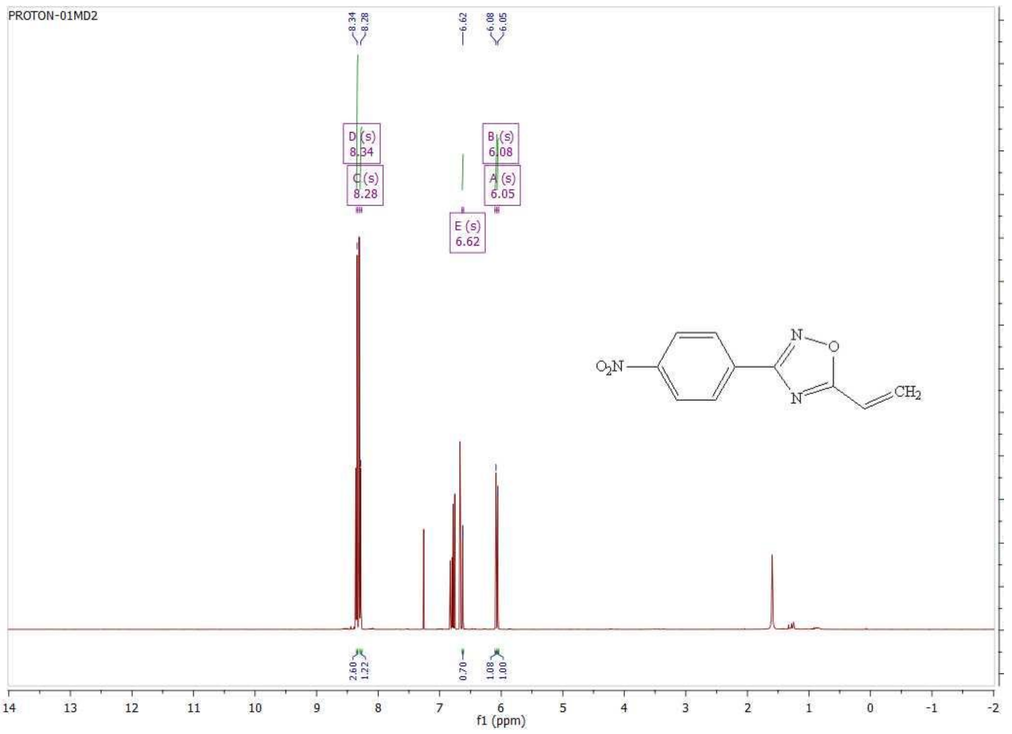

Figure S16. ^{1}H-NMR Spectrum of compound **6b**

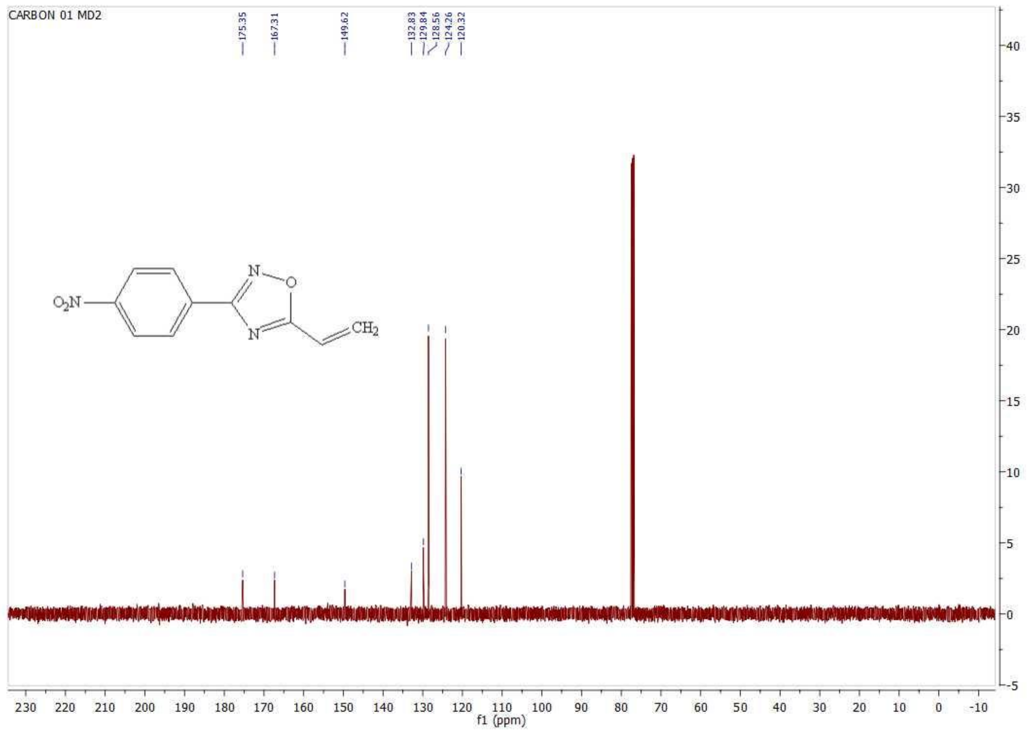

Figure S17. ^{13}C-NMR Spectrum of compound **6b**

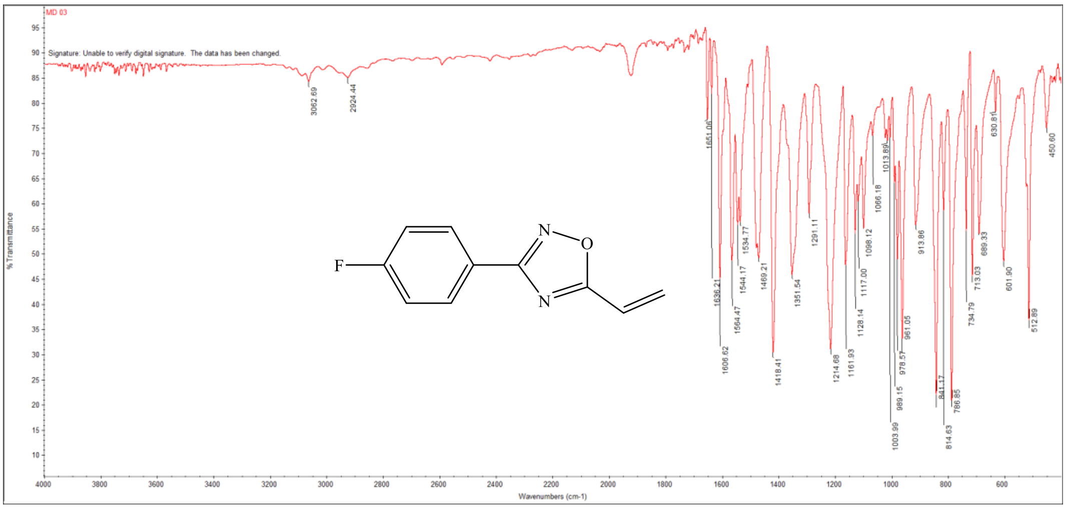

Figure S18. IR Spectrum of compound **6c**

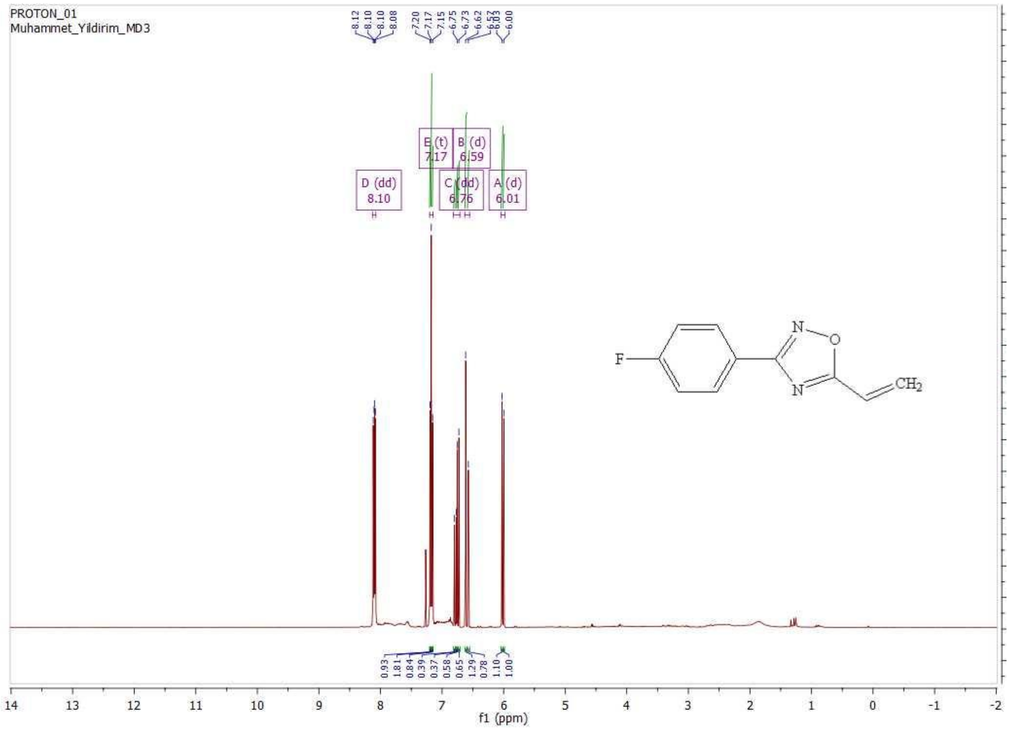

Figure S19. ^{1}H-NMR Spectrum of compound **6c**

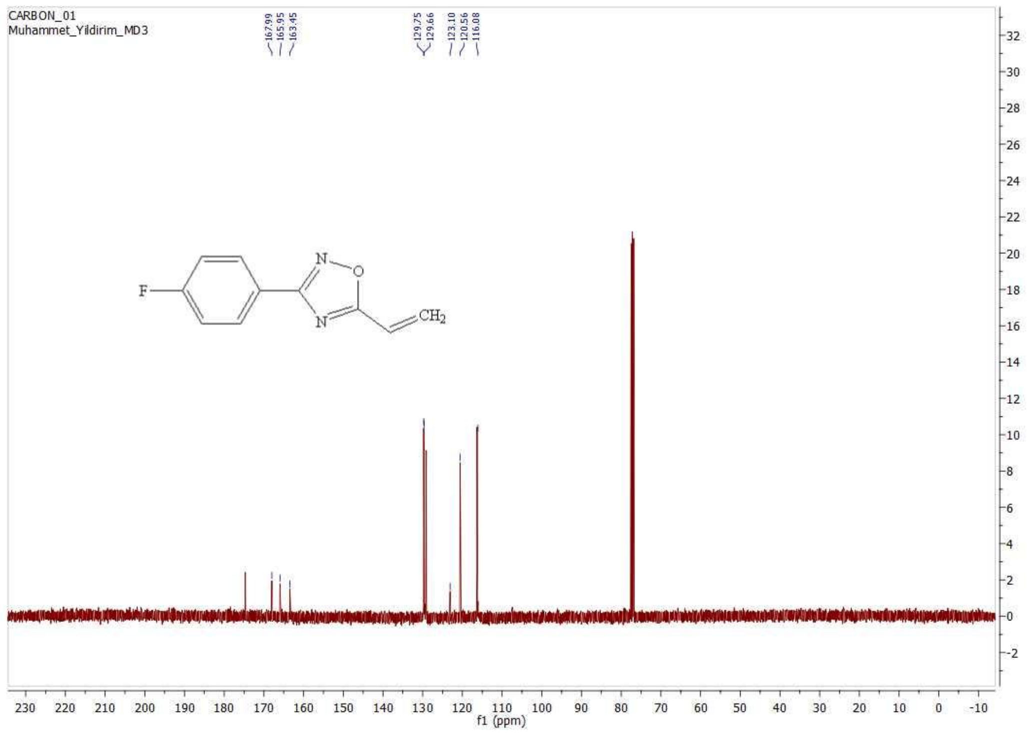

Figure S20. ^{13}C-NMR Spectrum of compound **6c**

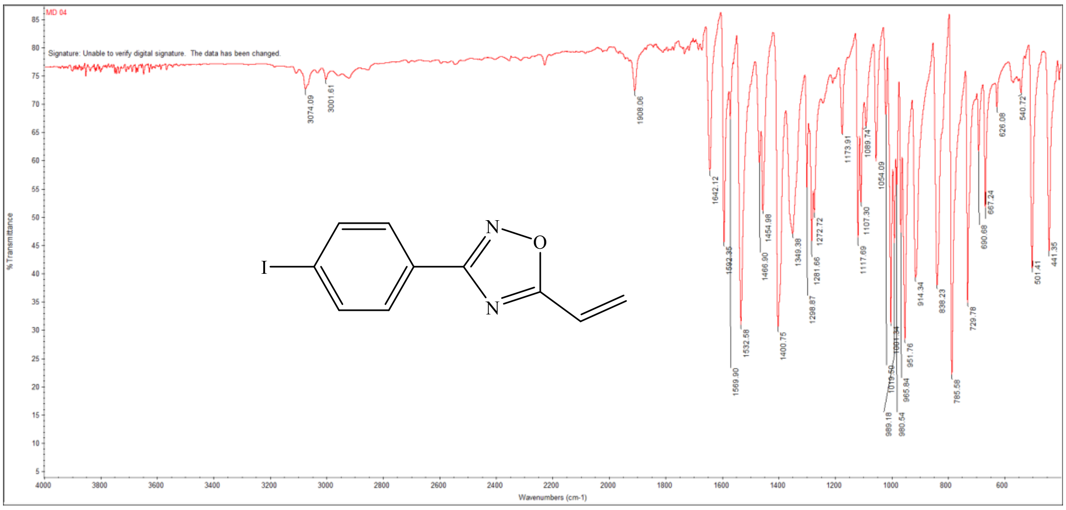

Figure S21. IR Spectrum of compound **6d**

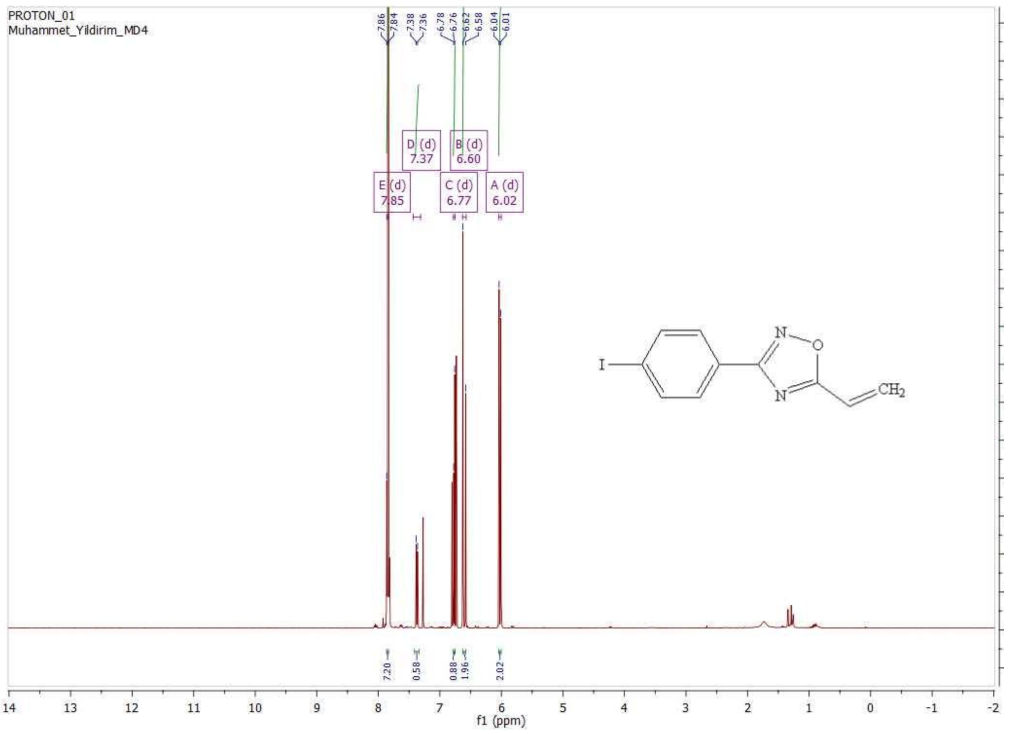

Figure S22. ^{1}H-NMR Spectrum of compound **6d**

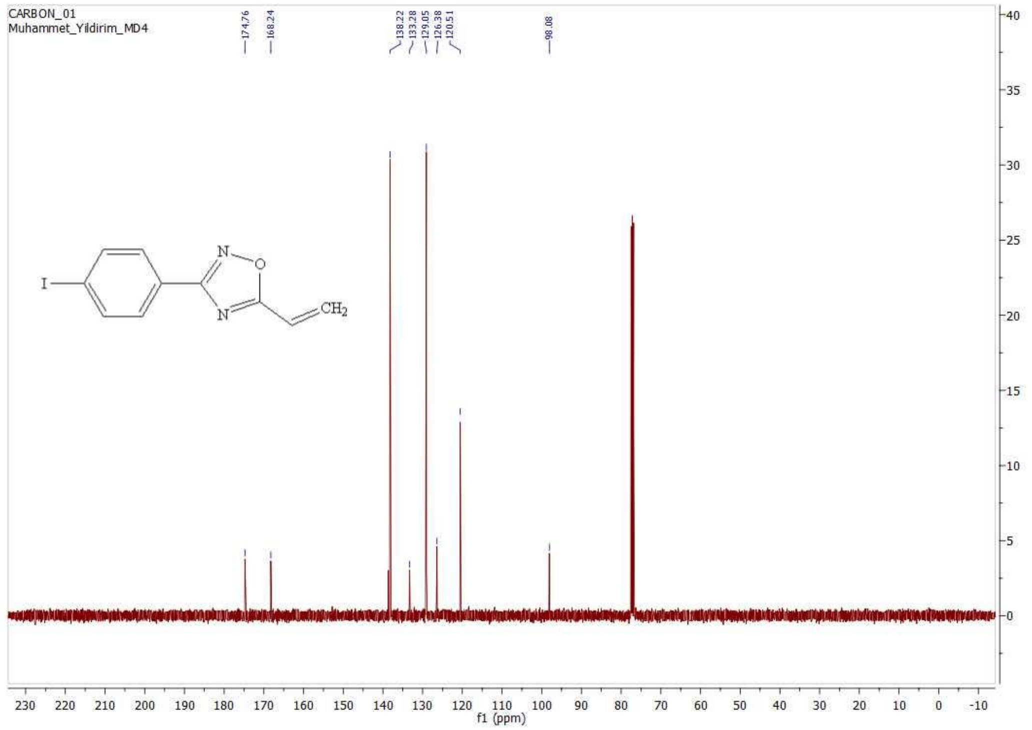

Figure S23. ^{13}C-NMR Spectrum of compound **6d**

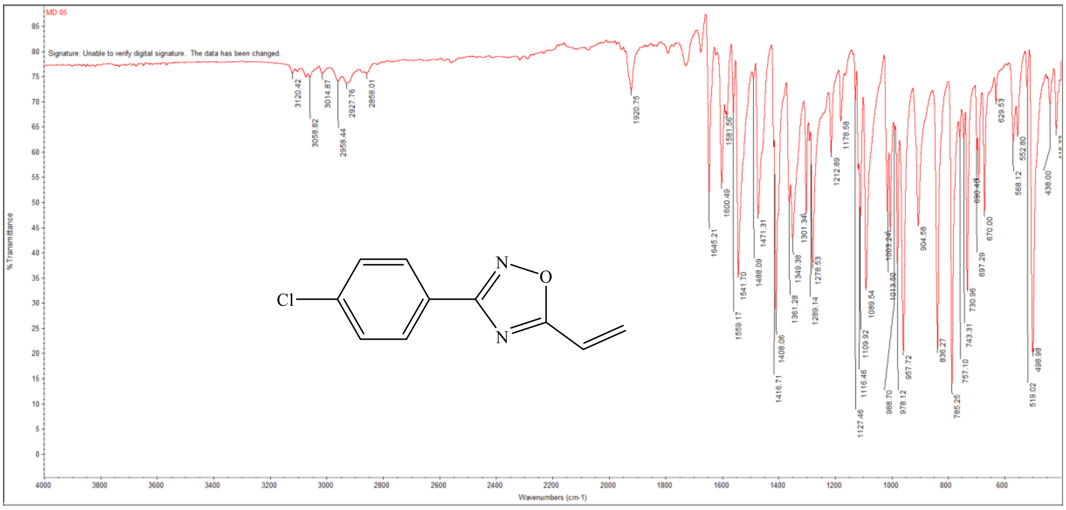

Figure S24. IR Spectrum of compound **6e**

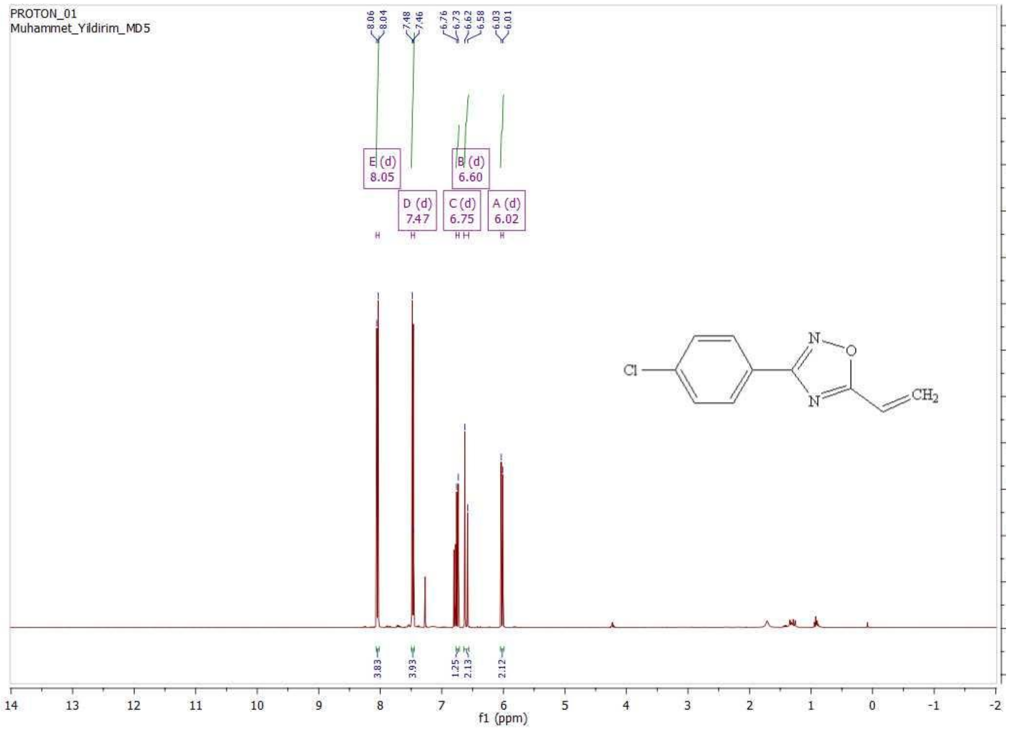

Figure S25. ^{1}H-NMR Spectrum of compound **6e**

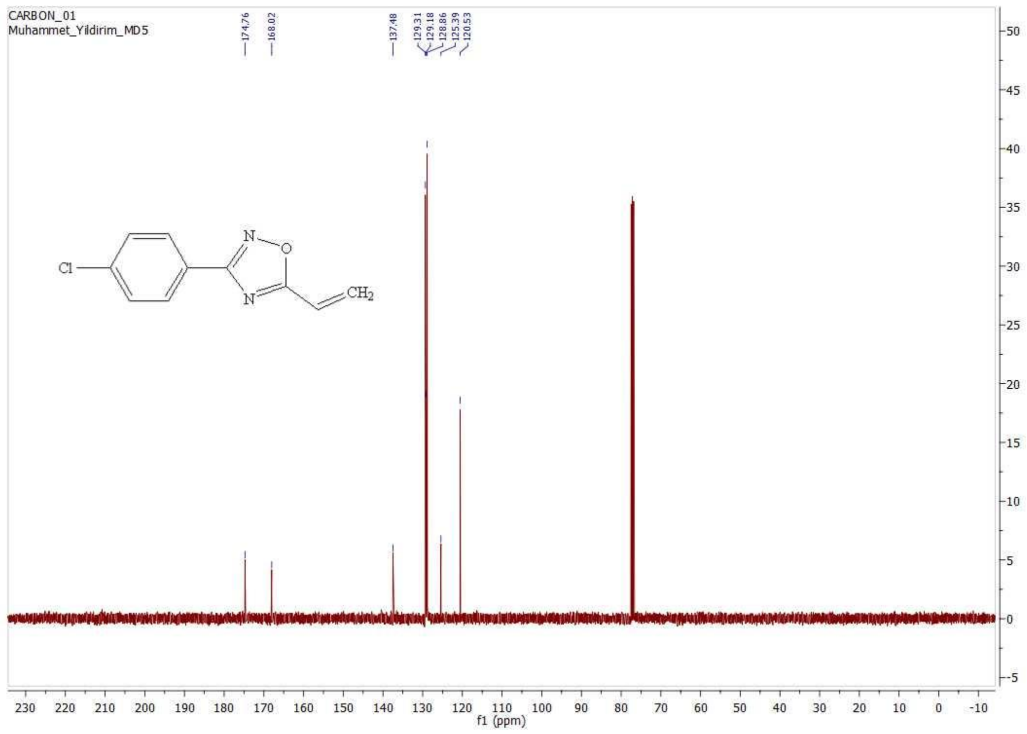

Figure S26. ^{13}C-NMR Spectrum of compound **6e**

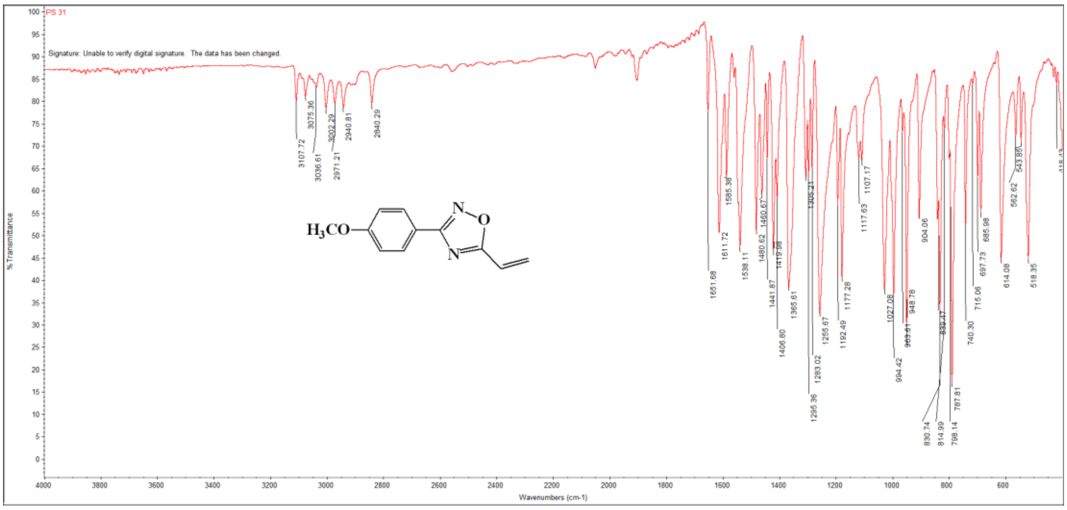

Figure S27. IR Spectrum of compound **6f**

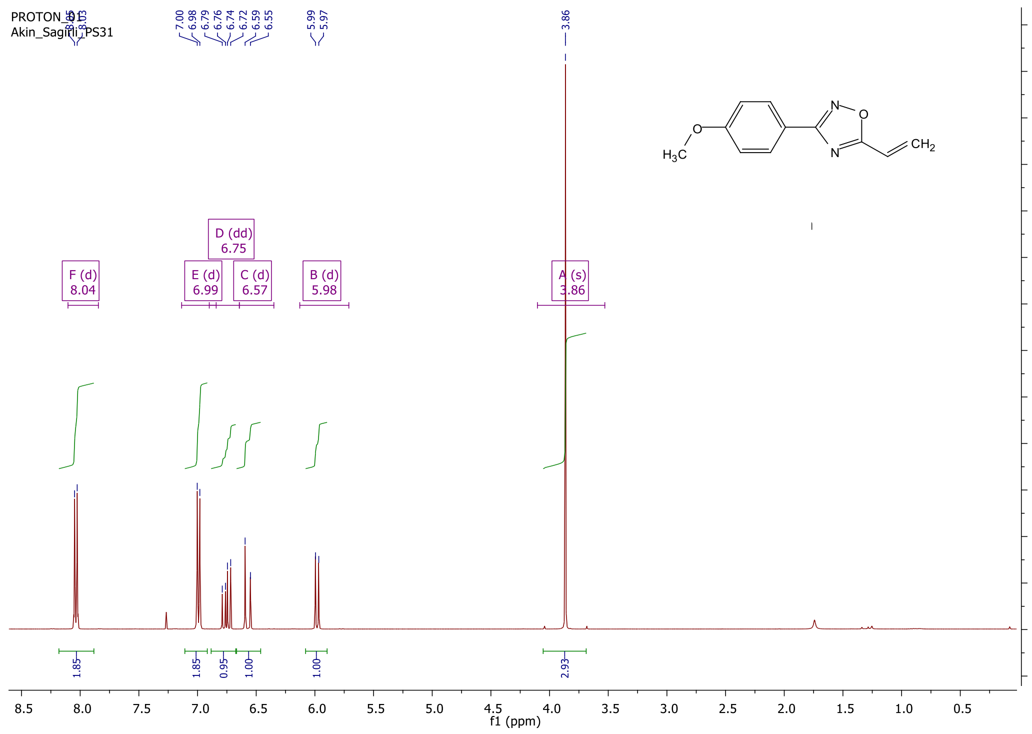

Figure S28. ^{1}H-NMR Spectrum of compound **6f**

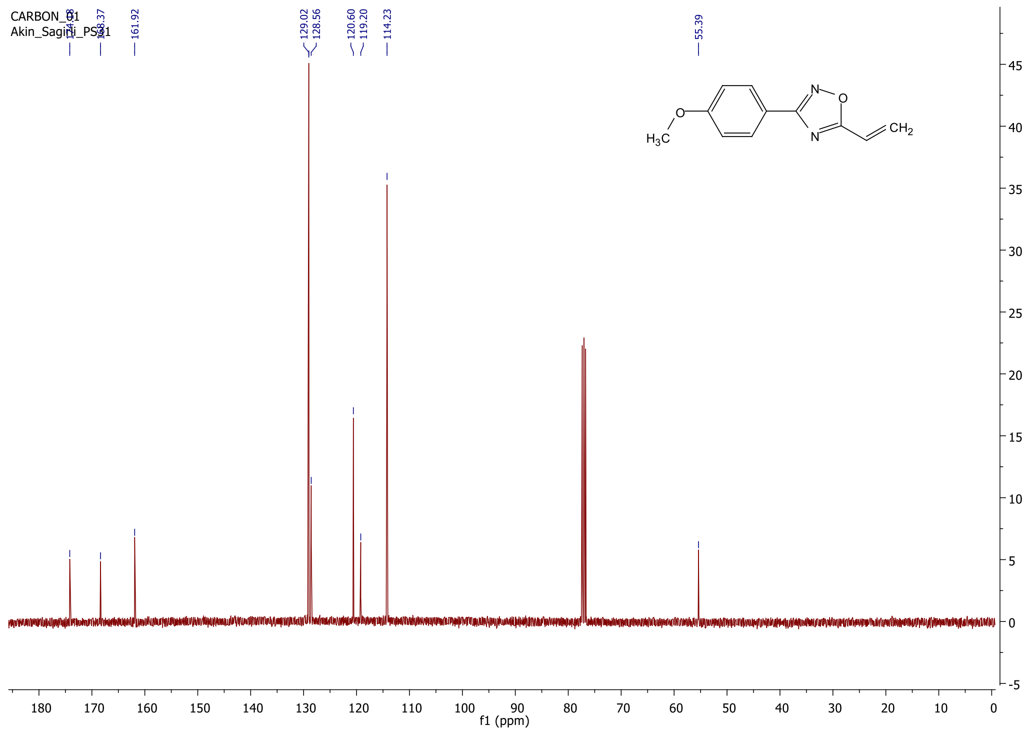

Figure S29. ^{13}C-NMR Spectrum of compound **6f**

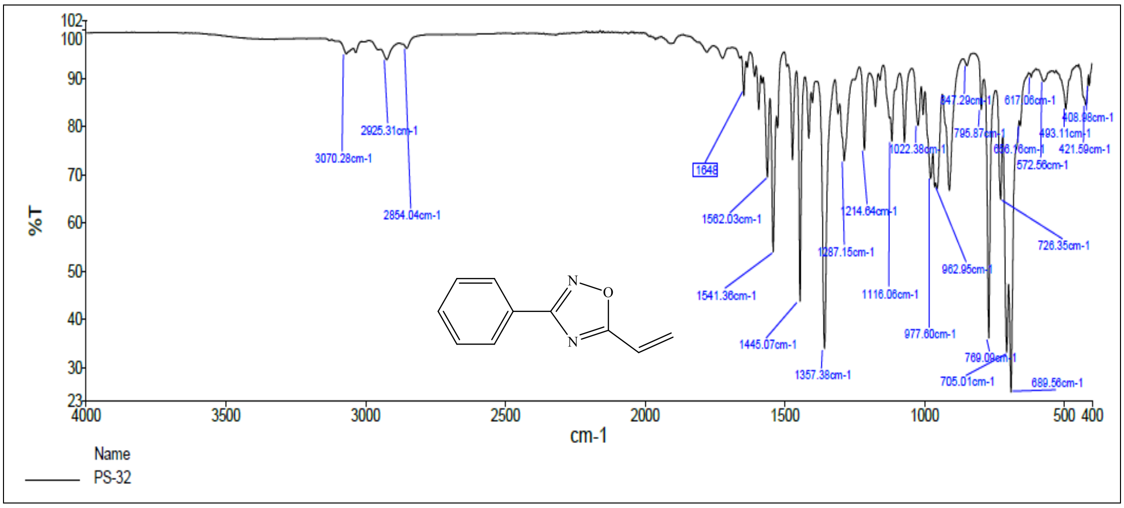

Figure S30. IR Spectrum of compound **6g**

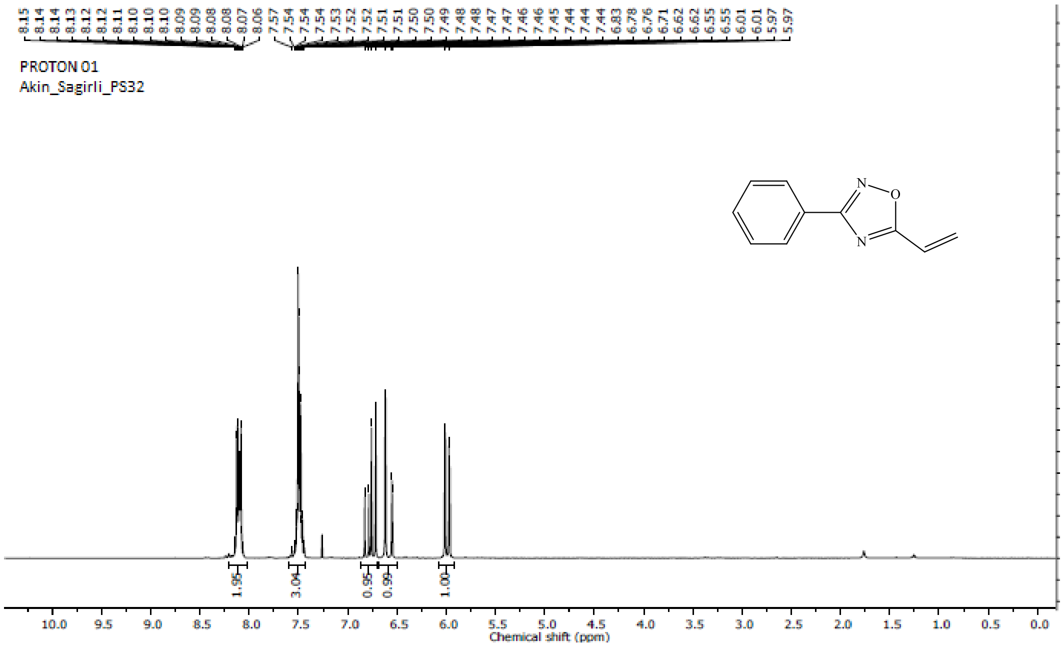

Figure S31. ^{1}H-NMR Spectrum of compound **6g**

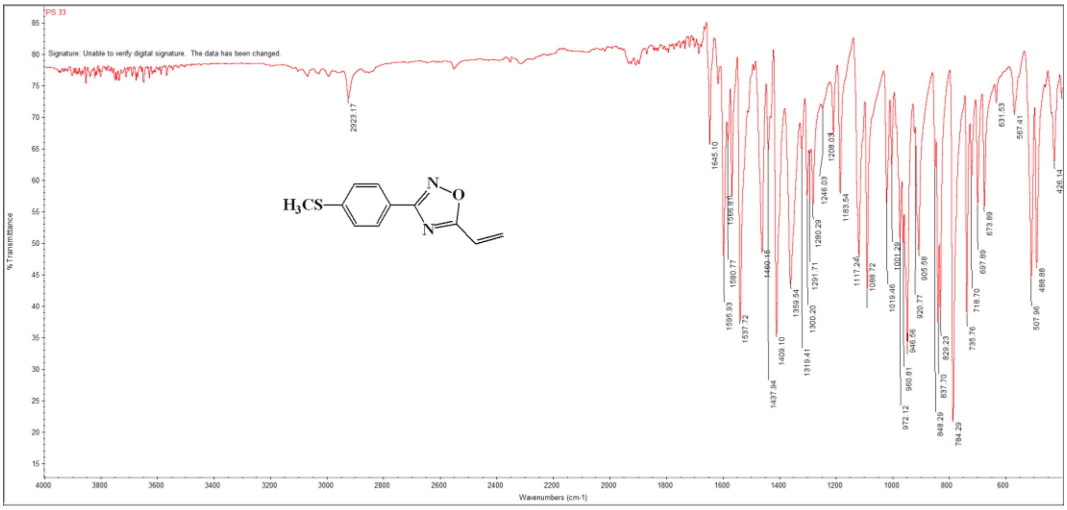

Figure S32. IR Spectrum of compound **6h**

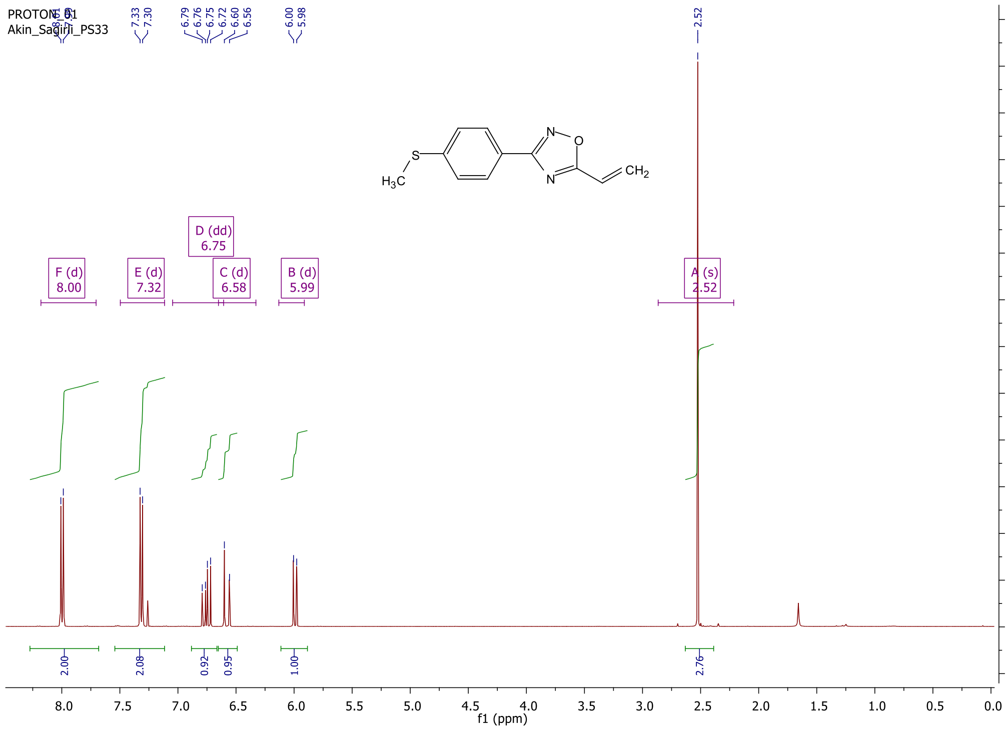

Figure S33. ^{1}H-NMR Spectrum of compound **6h**

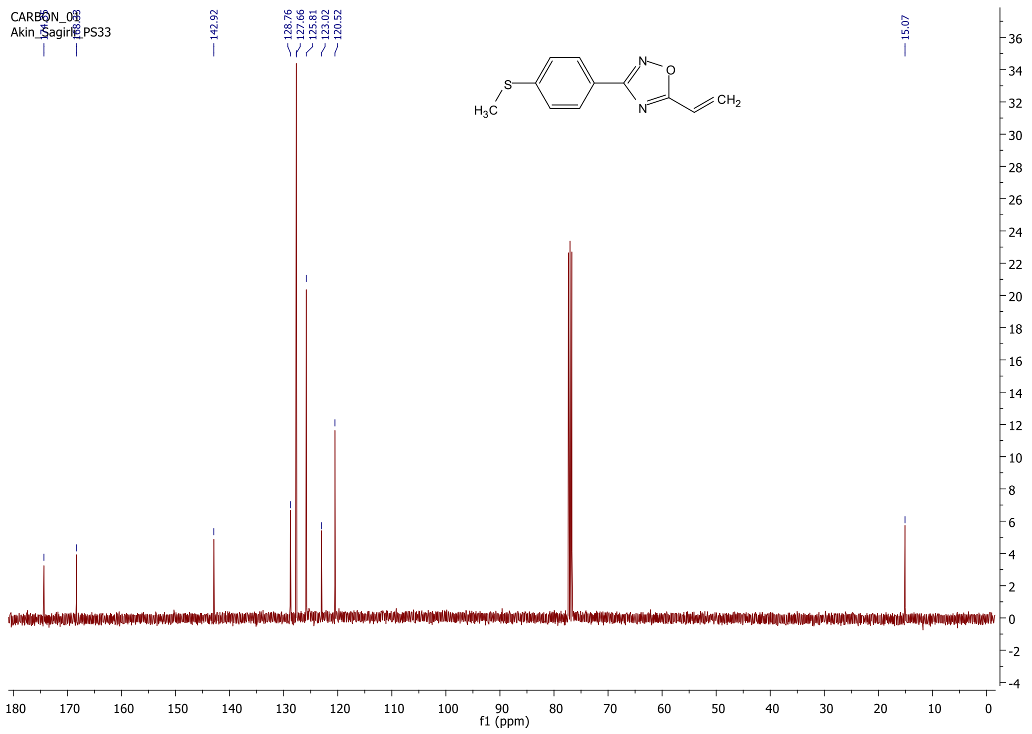

Figure S34. ^{13}C-NMR Spectrum of compound **6h**

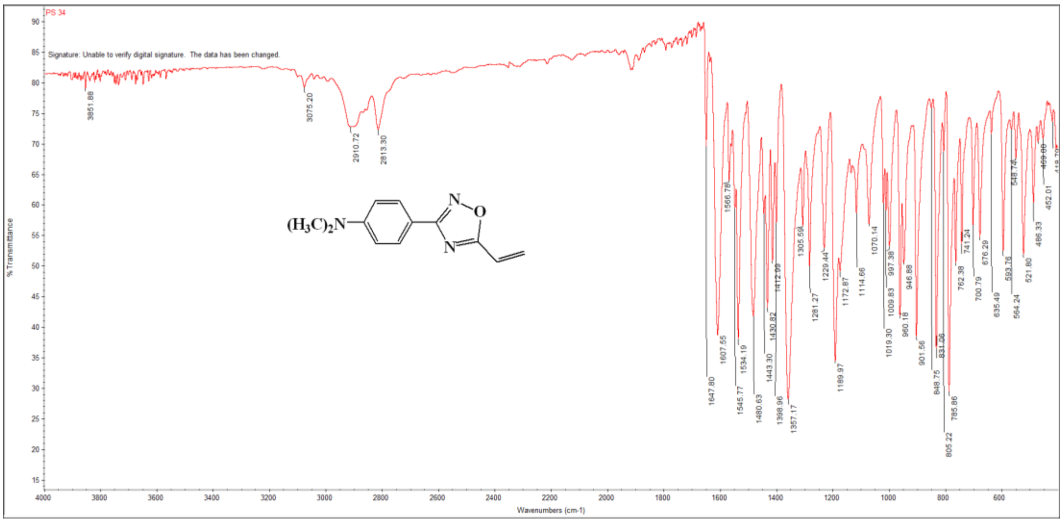

Figure S35. IR Spectrum of compound **6i**

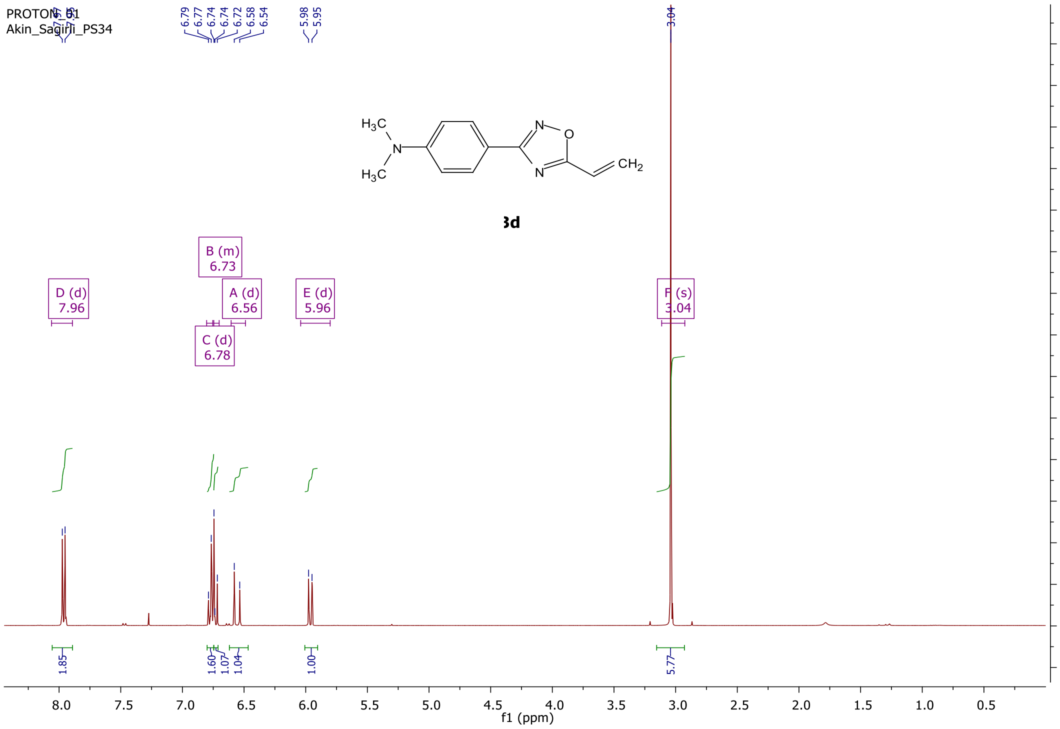

Figure S36. ^{1}H-NMR Spectrum of compound **6i**

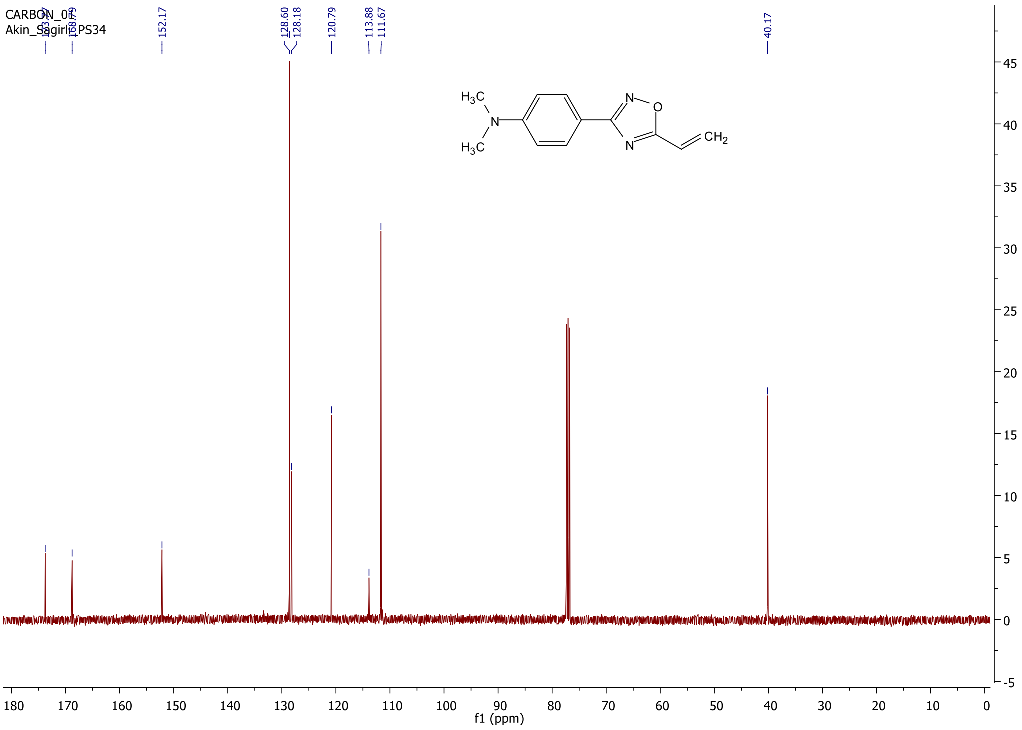

Figure S37. ^{13}C-NMR Spectrum of compound **6i**

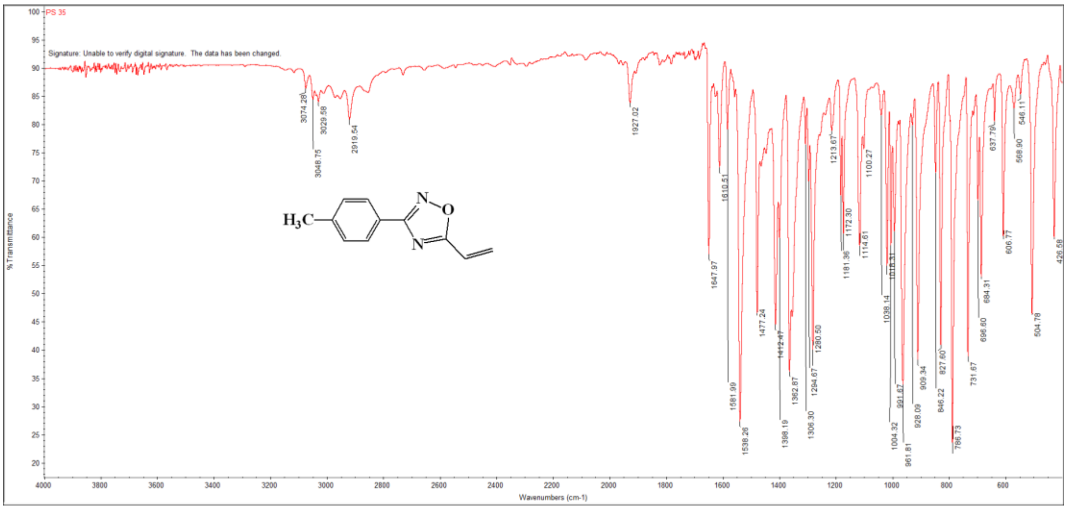

Figure S38. IR Spectrum of compound **6j**

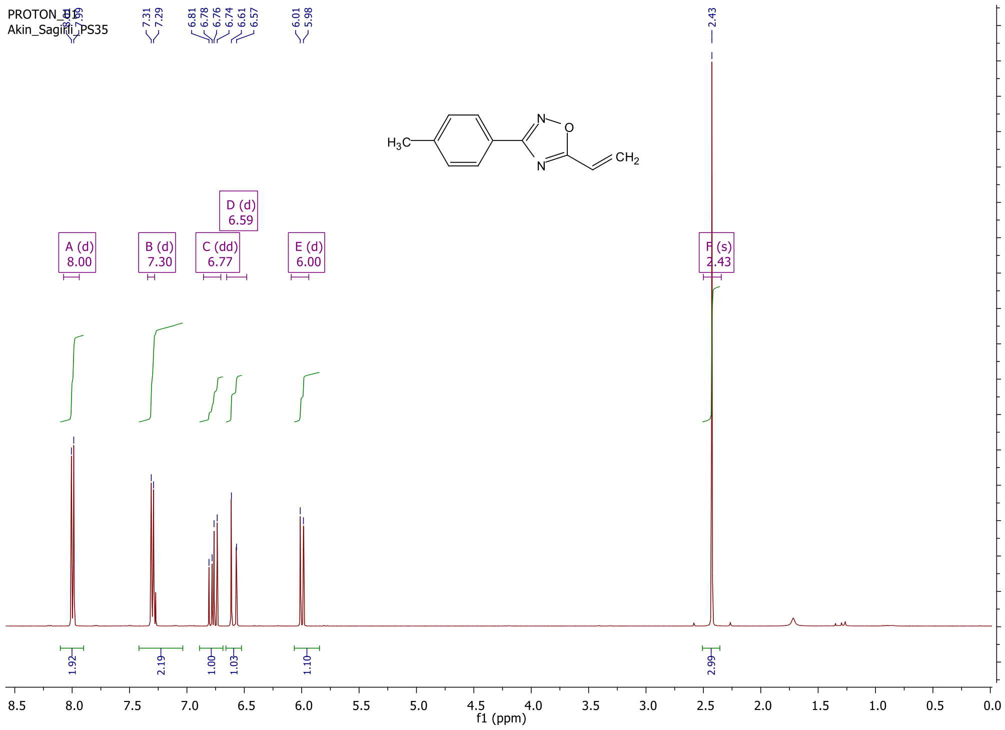

Figure S39. ^{1}H-NMR Spectrum of compound **6j**

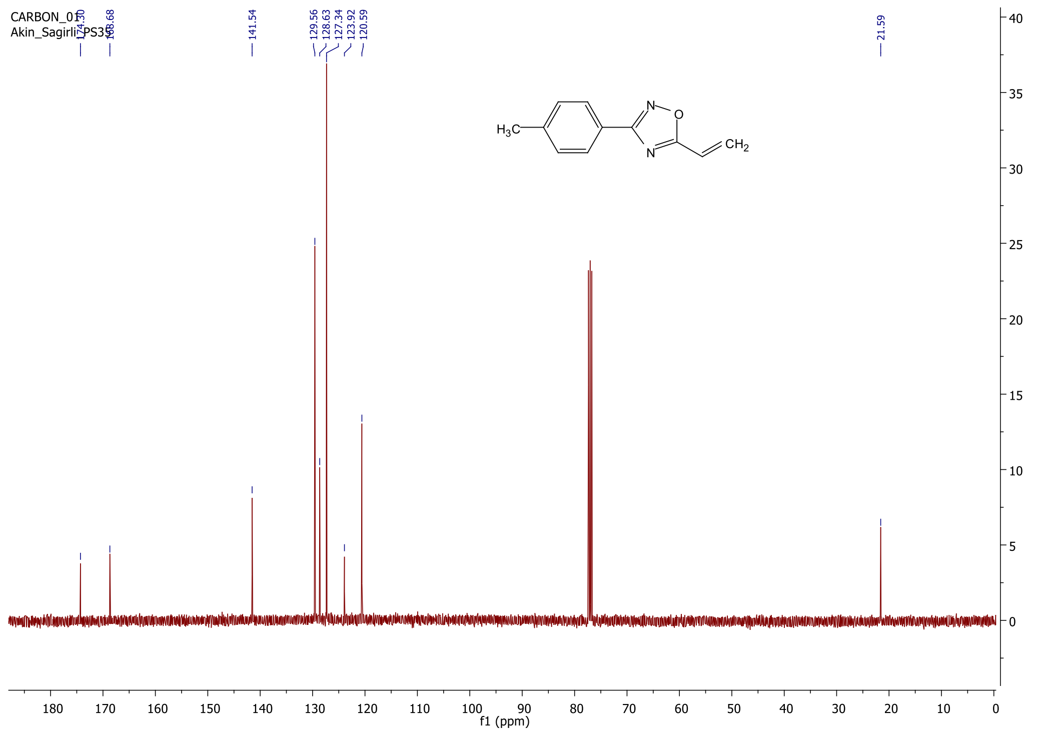

Figure S40. ^{13}C-NMR Spectrum of compound **6j**

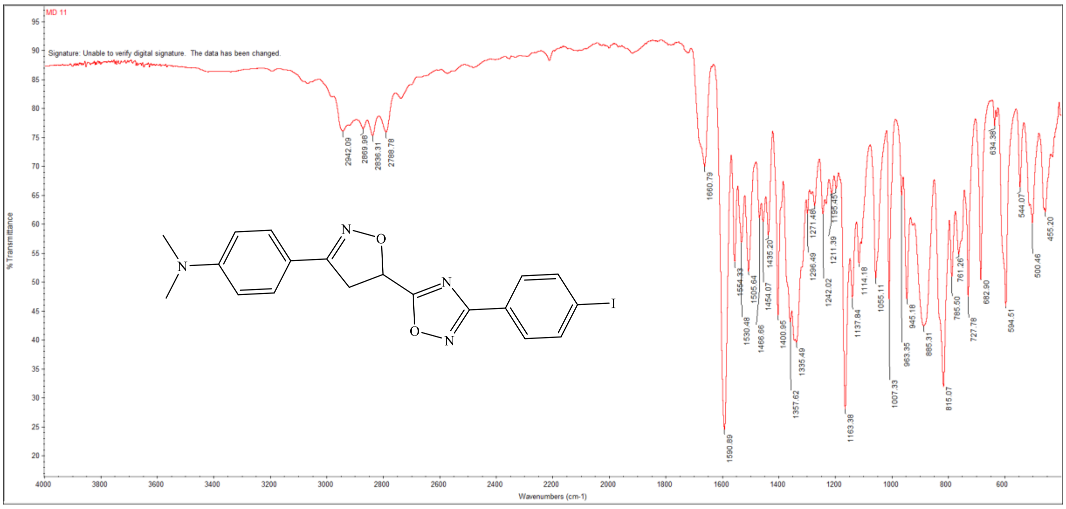

Figure S41. IR Spectrum of compound **7a**

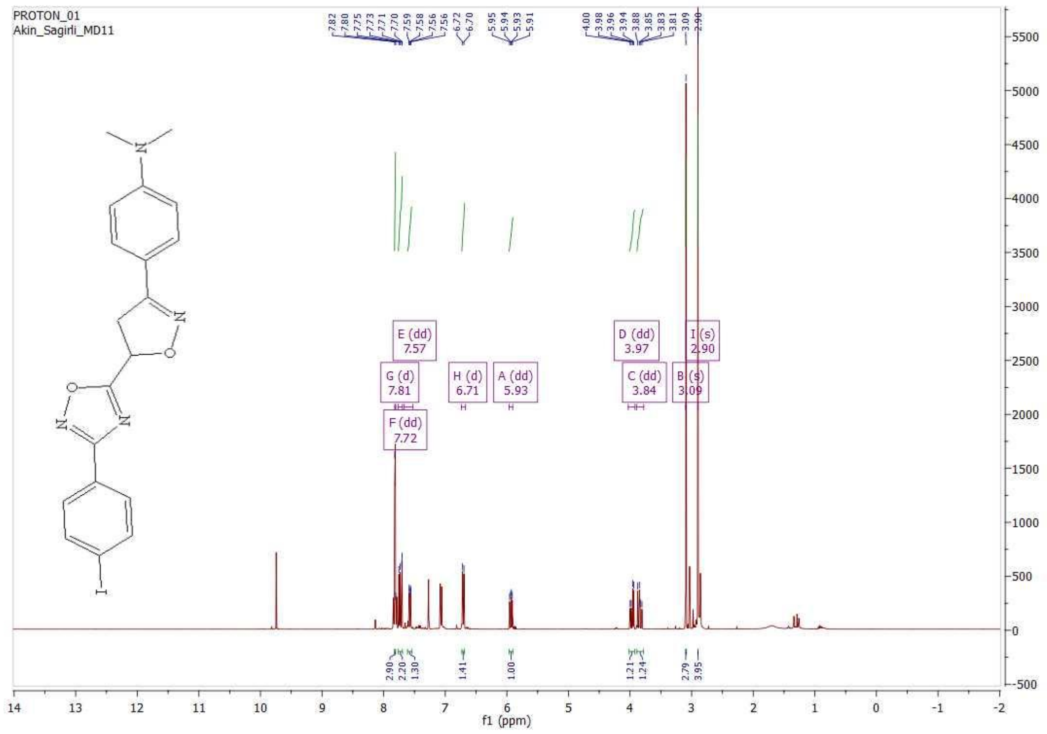

Figure S42. ^{1}H-NMR Spectrum of compound **7a**

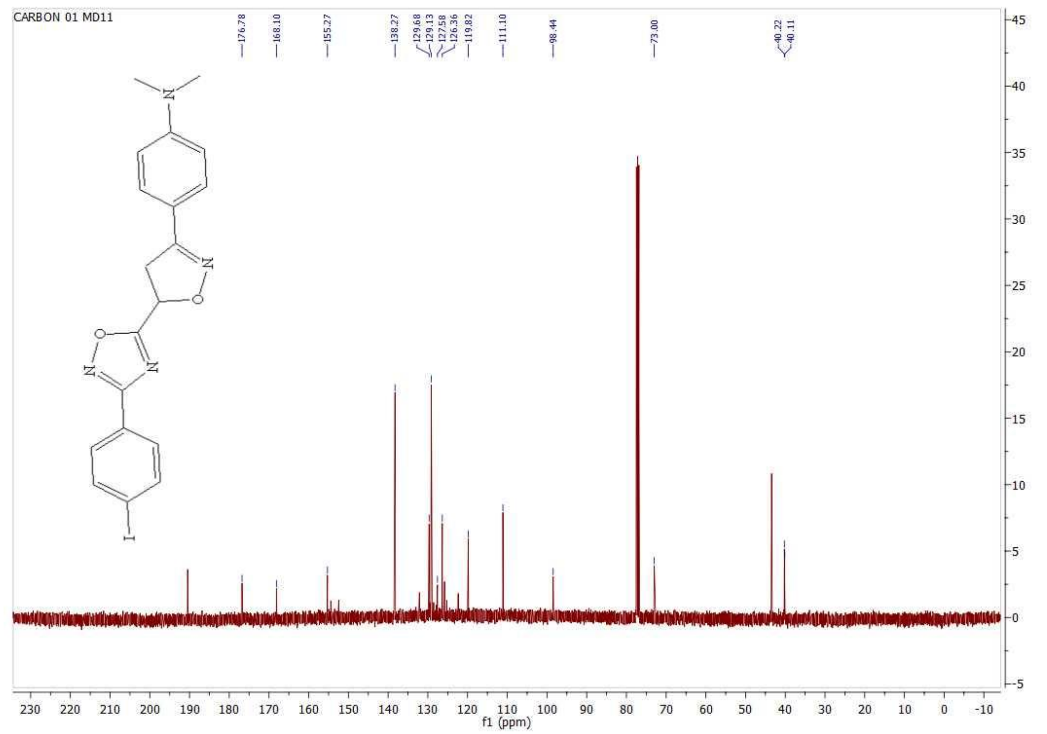

Figure S43. ^{13}C-NMR Spectrum of compound **7a**

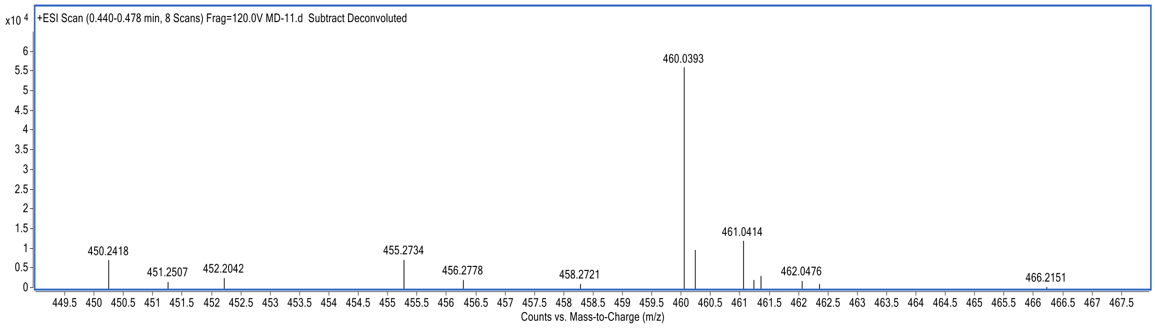

Figure S44. HRMS Spectrum of compound **7a**

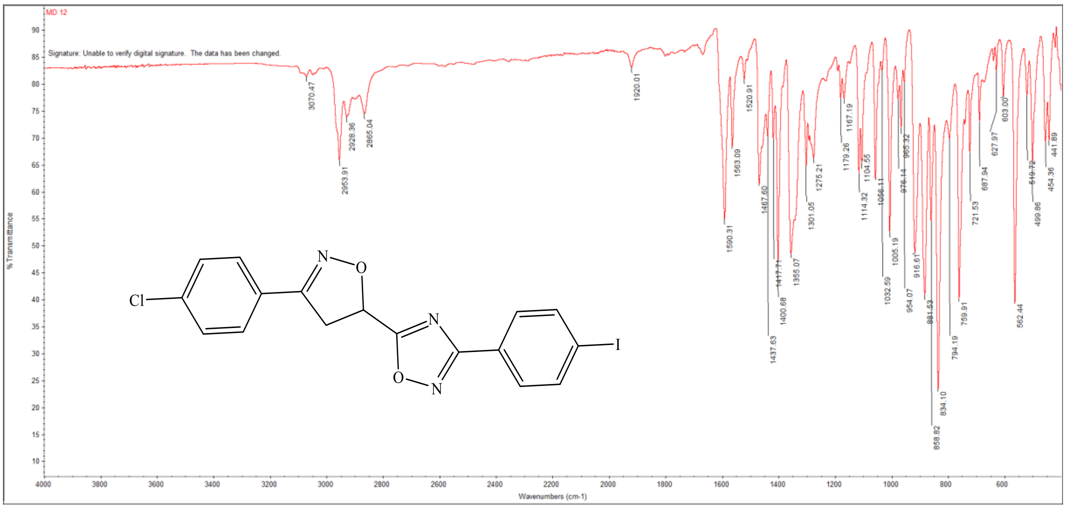

Figure S45. IR Spectrum of compound **7b**

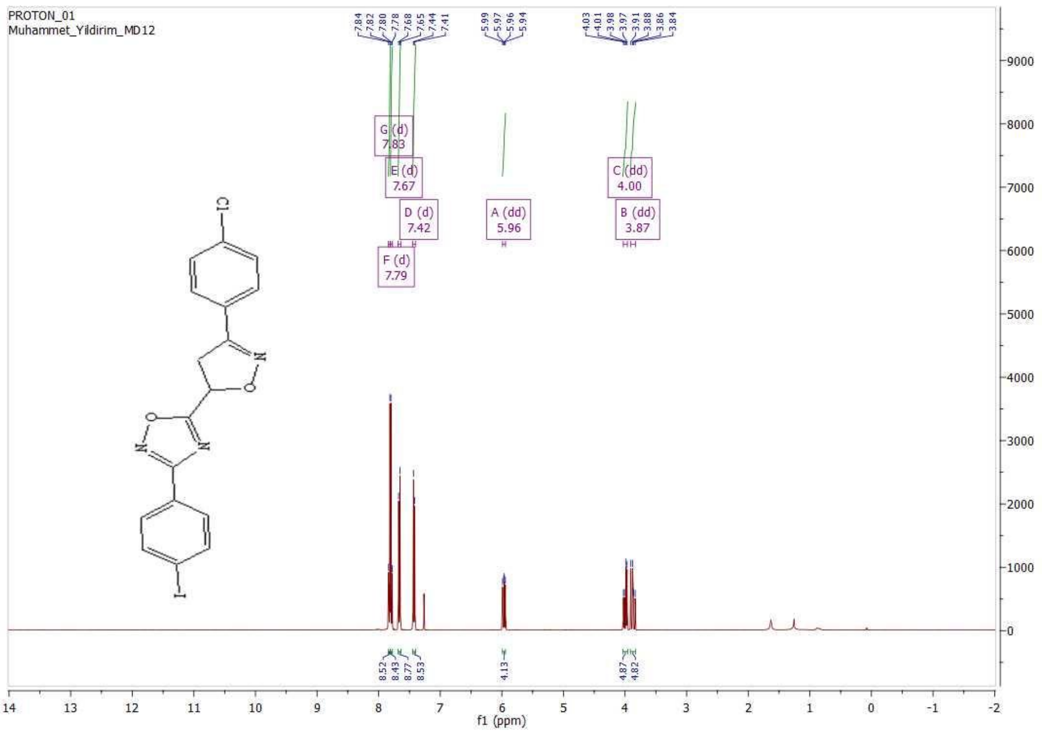

Figure S46. ^{1}H-NMR Spectrum of compound **7b**

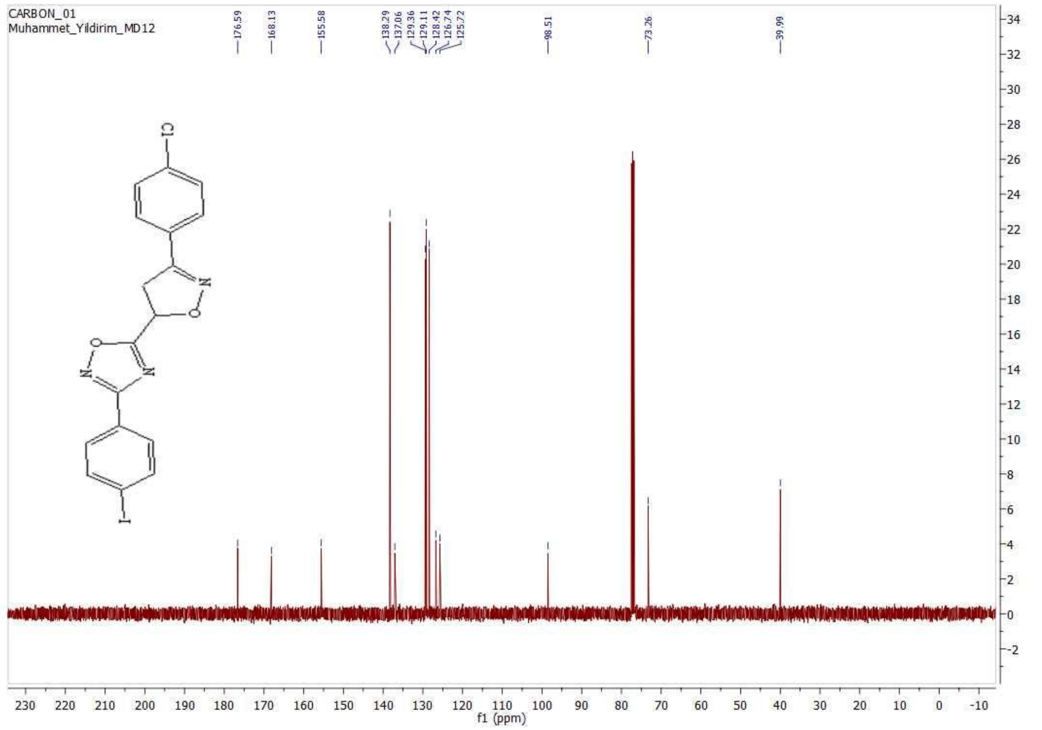

Figure S47. ^{13}C-NMR Spectrum of compound **7b**

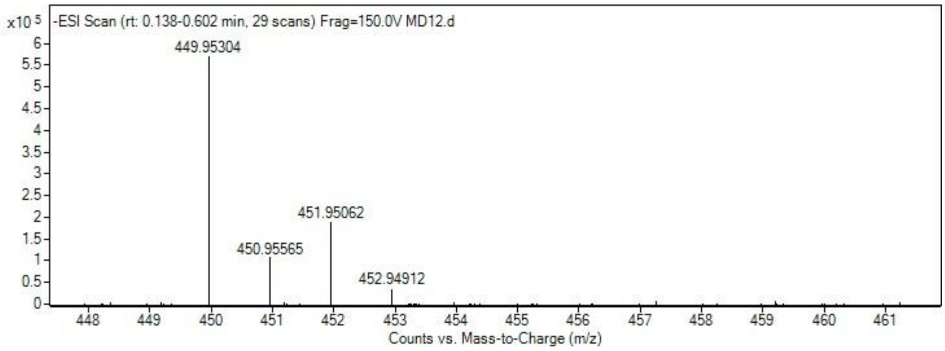

Figure S48. HRMS Spectrum of compound **7b**

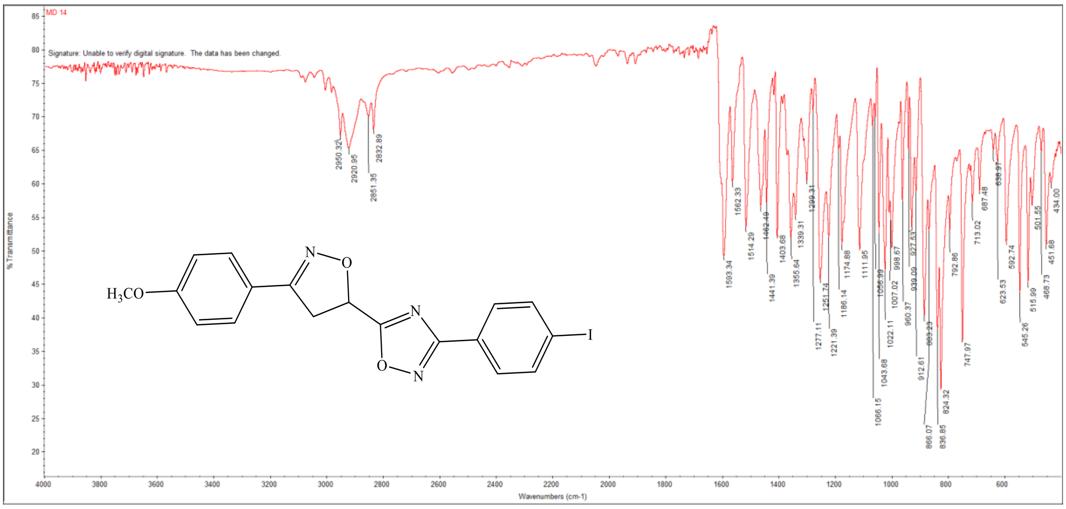

Figure S49. IR Spectrum of compound **7c**

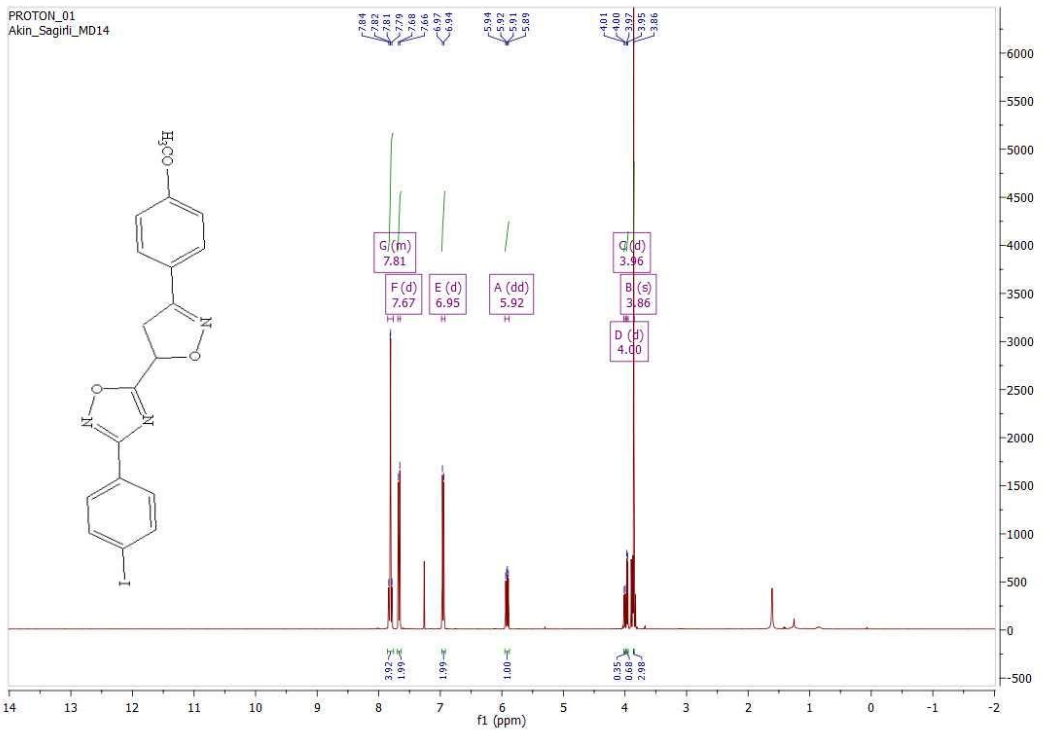

Figure S50. ^{1}H-NMR Spectrum of compound **7c**

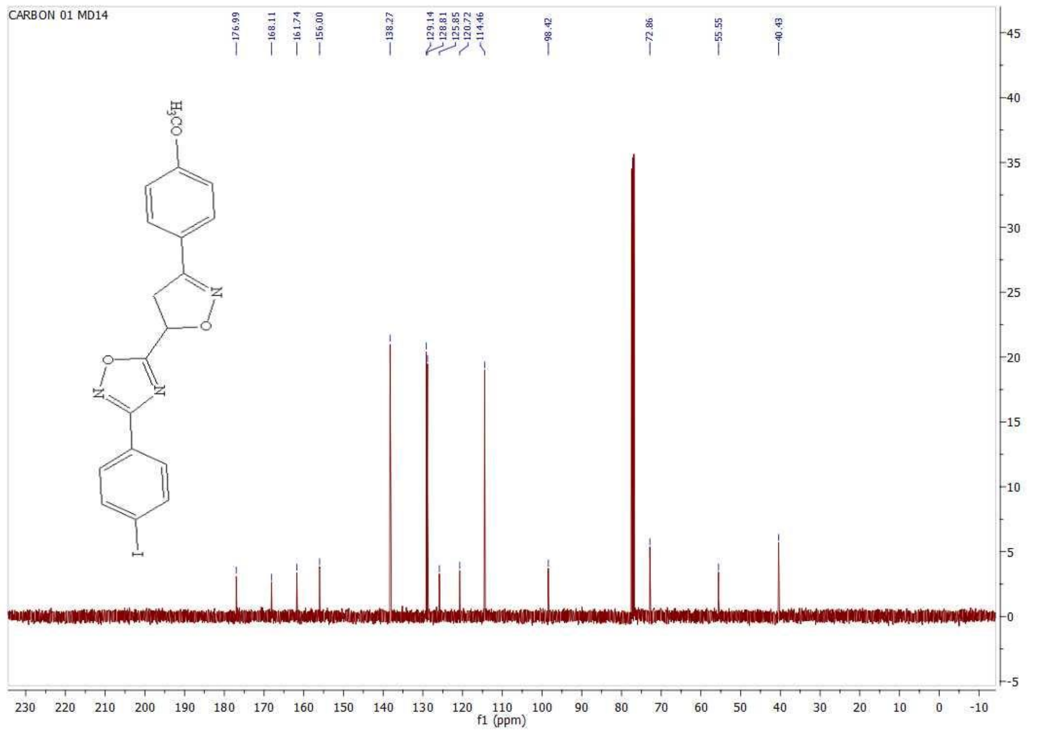

Figure S51. ^{13}C-NMR Spectrum of compound **7c**

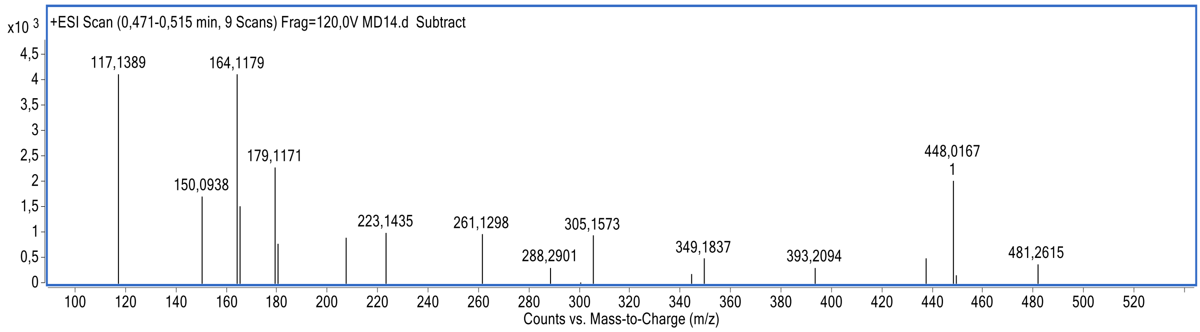

Figure S52. HRMS Spectrum of compound **7c**

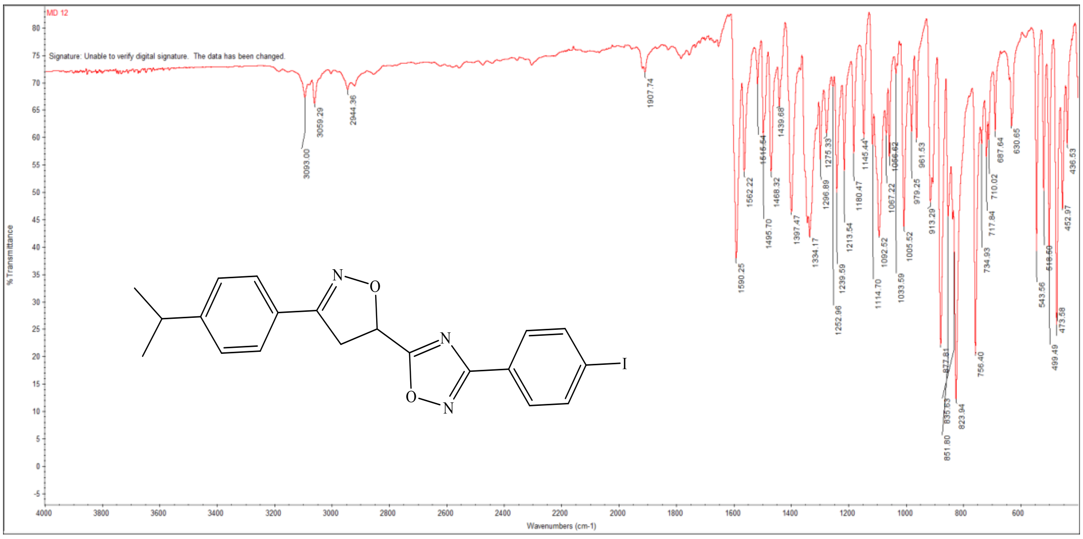

Figure S53. IR Spectrum of compound **7d**

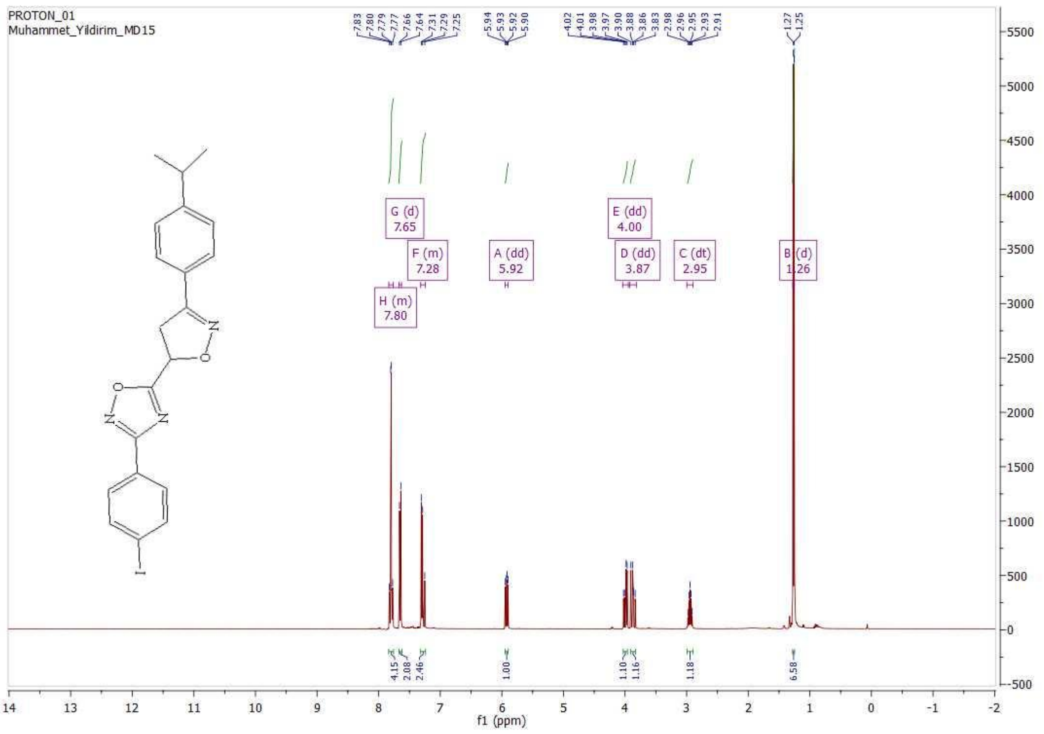

Figure S54. ^{1}H-NMR Spectrum of compound **7d**

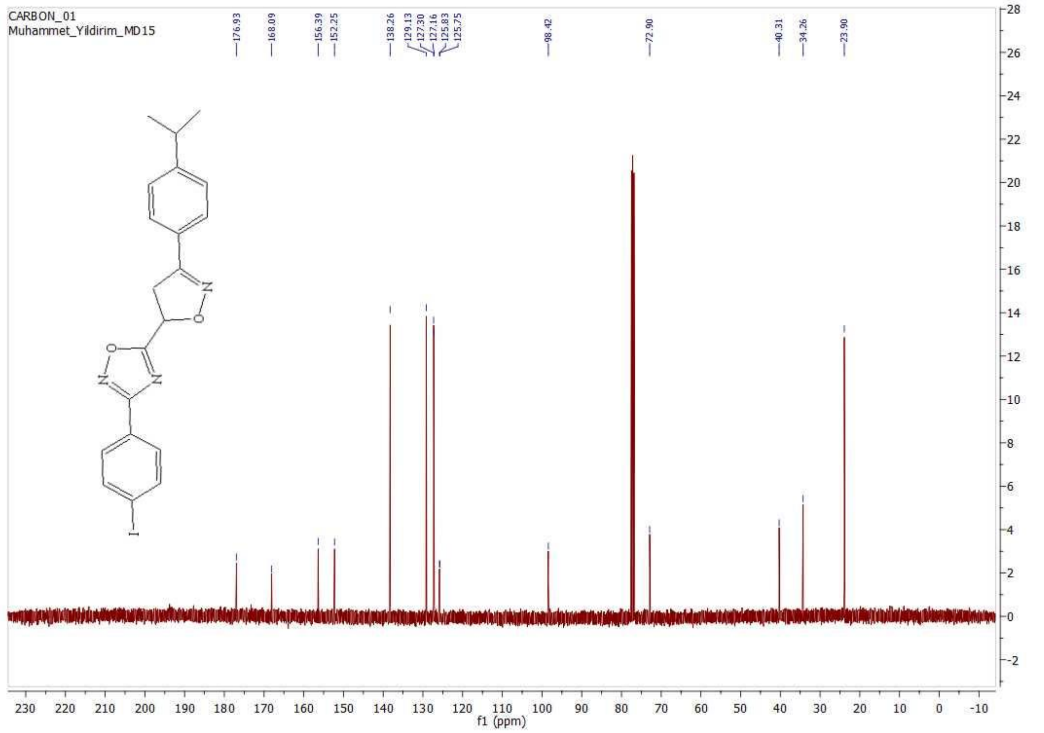

Figure S55. ^{13}C-NMR Spectrum of compound **7d**

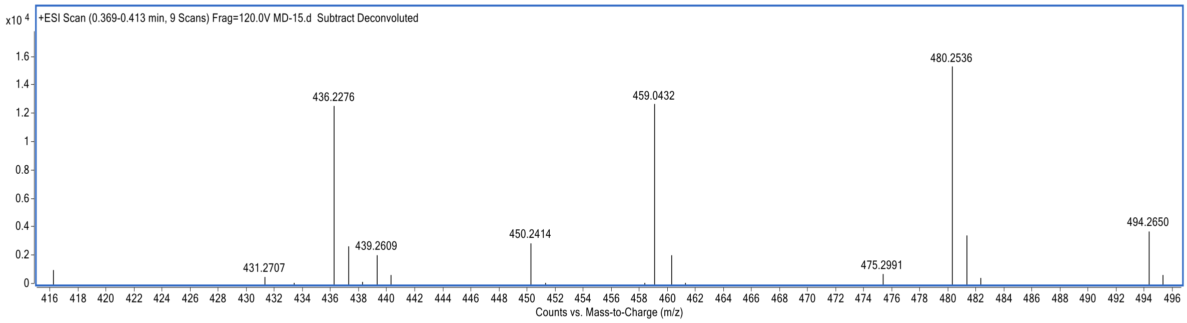

Figure S56. HRMS Spectrum of compound **7d**

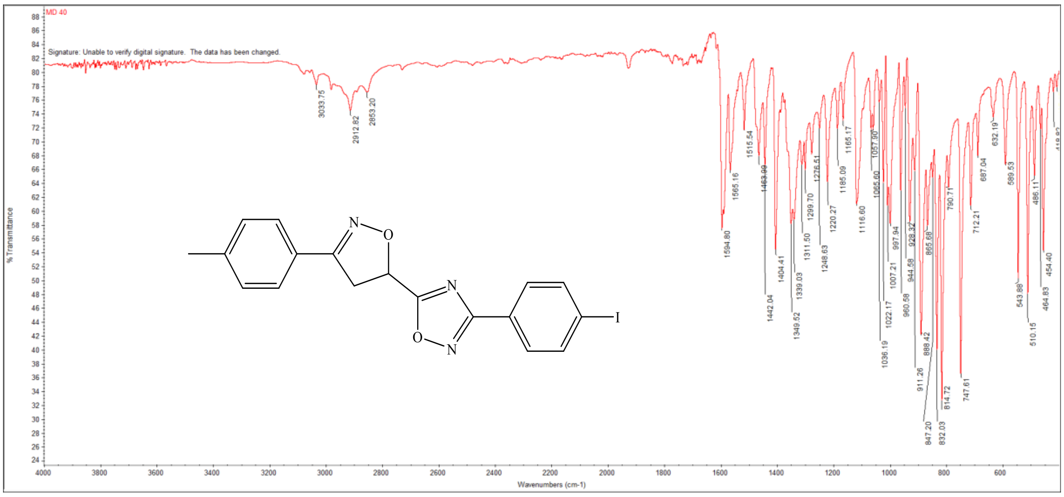

Figure S57. IR Spectrum of compound **7e**

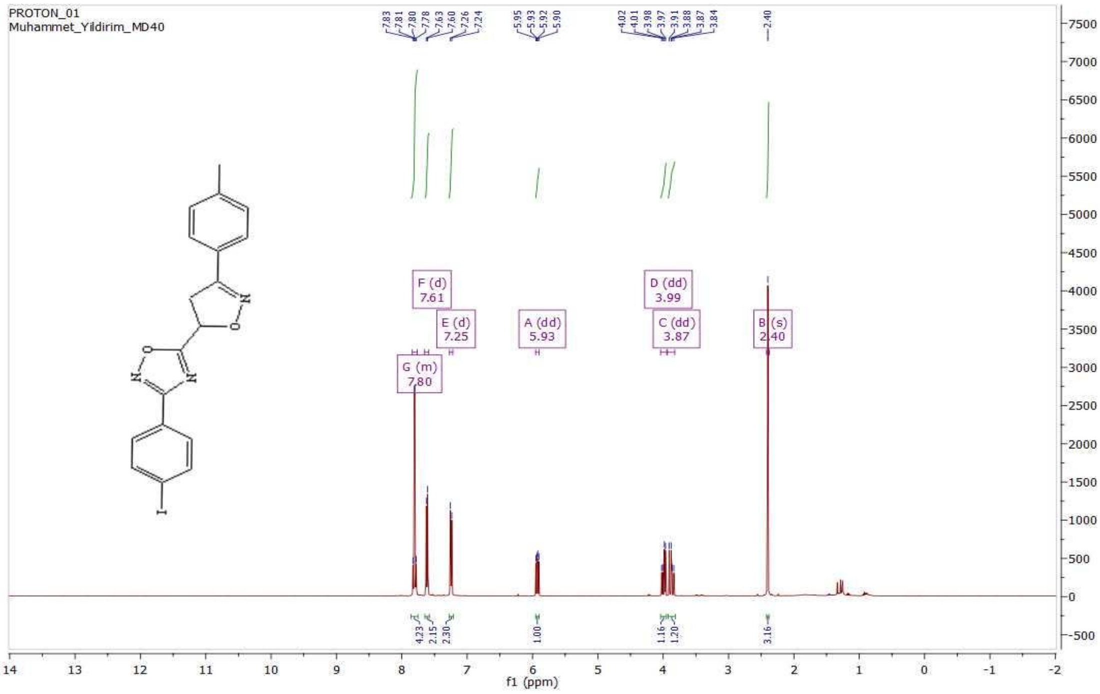

Figure S58. ^{1}H-NMR Spectrum of compound **7e**

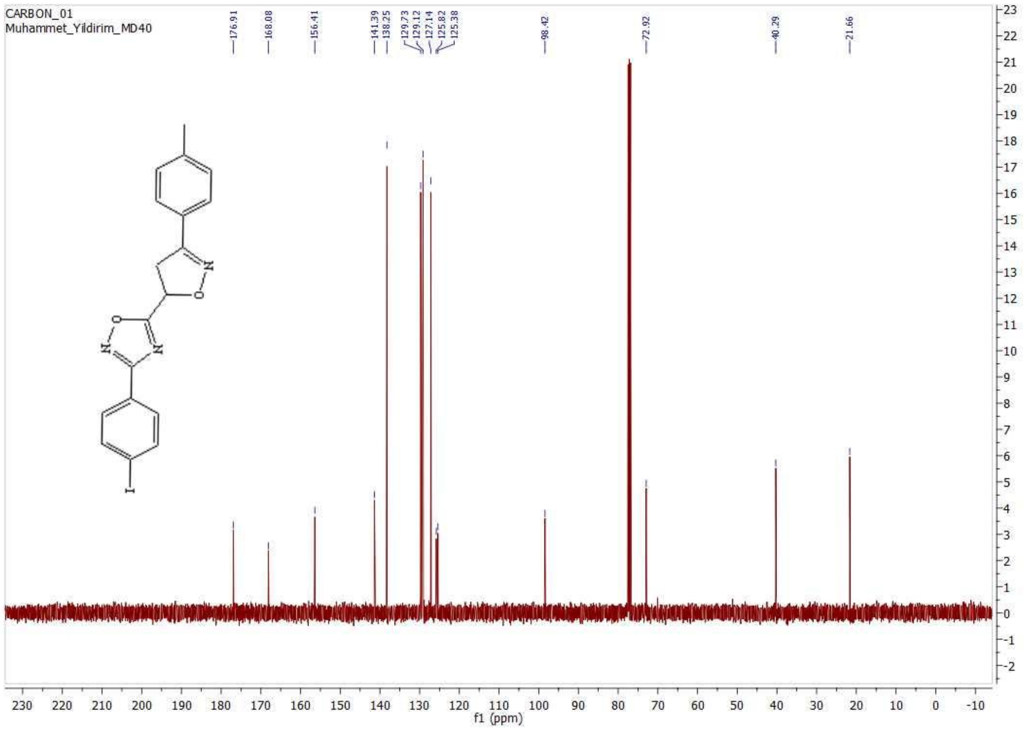

Figure S59. ^{13}C-NMR Spectrum of compound **7e**

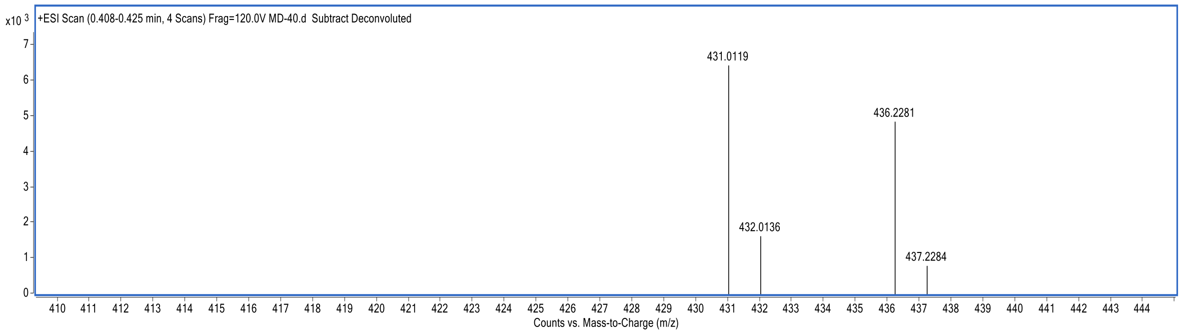

Figure S60. HRMS Spectrum of compound **7e**

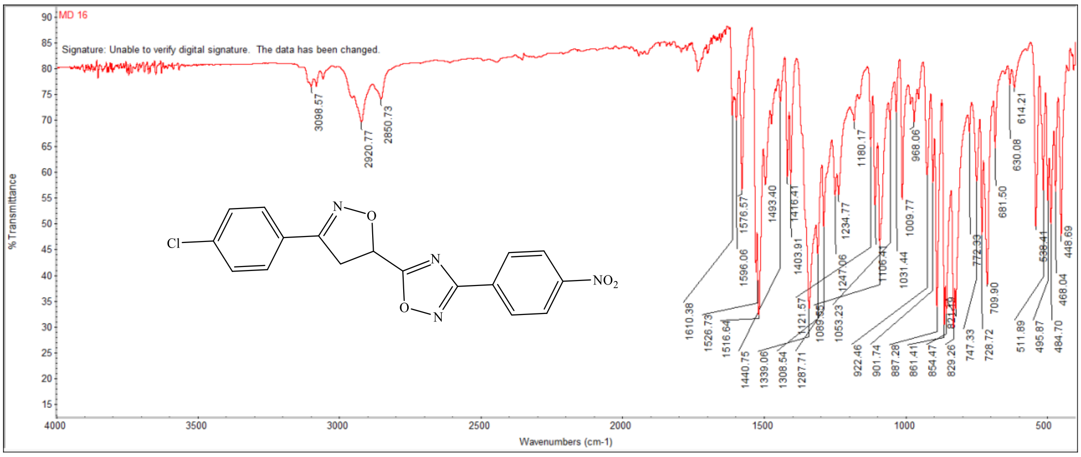

Figure S61. IR Spectrum of compound **7f**

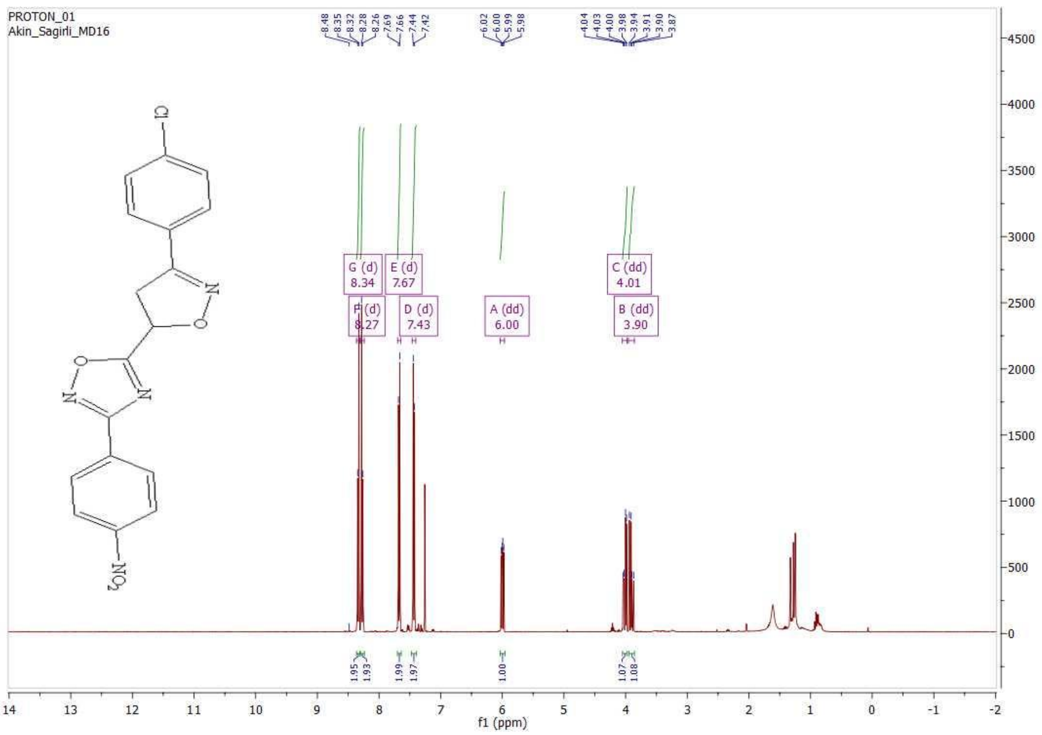

Figure S62. ^{1}H-NMR Spectrum of compound **7f**

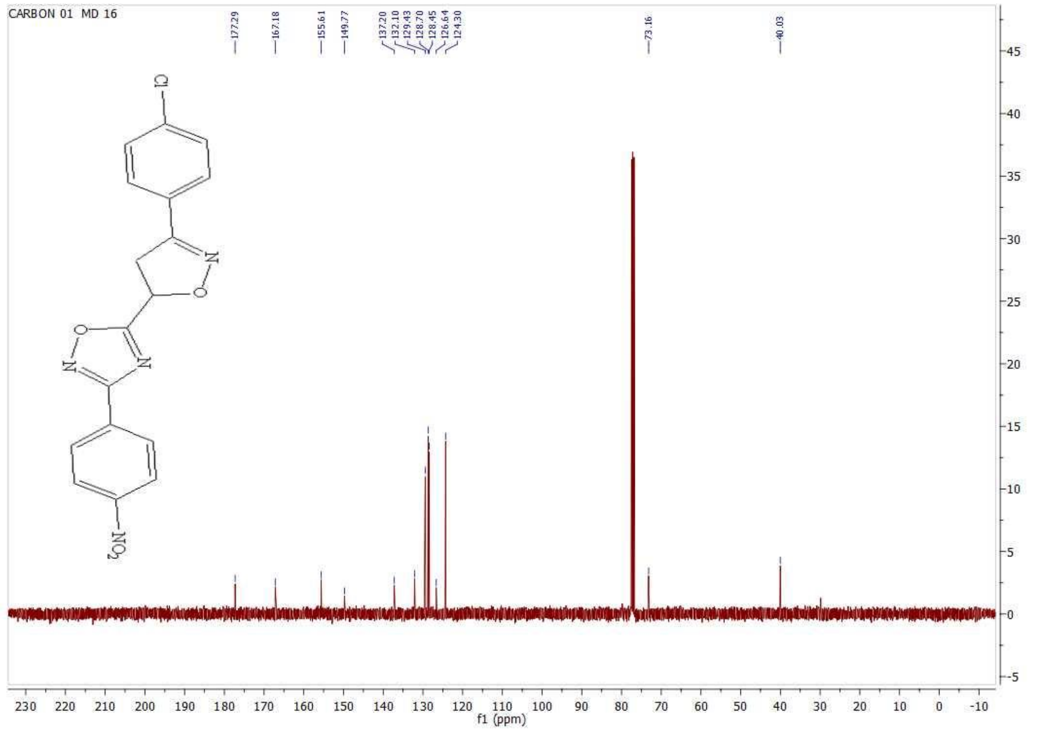

Figure S63. ^{13}C-NMR Spectrum of compound **7f**

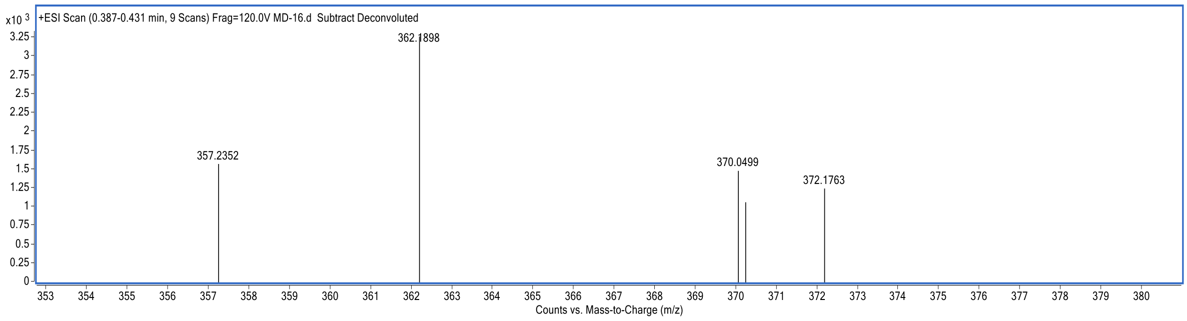

Figure S64. HRMS Spectrum of compound **7f**

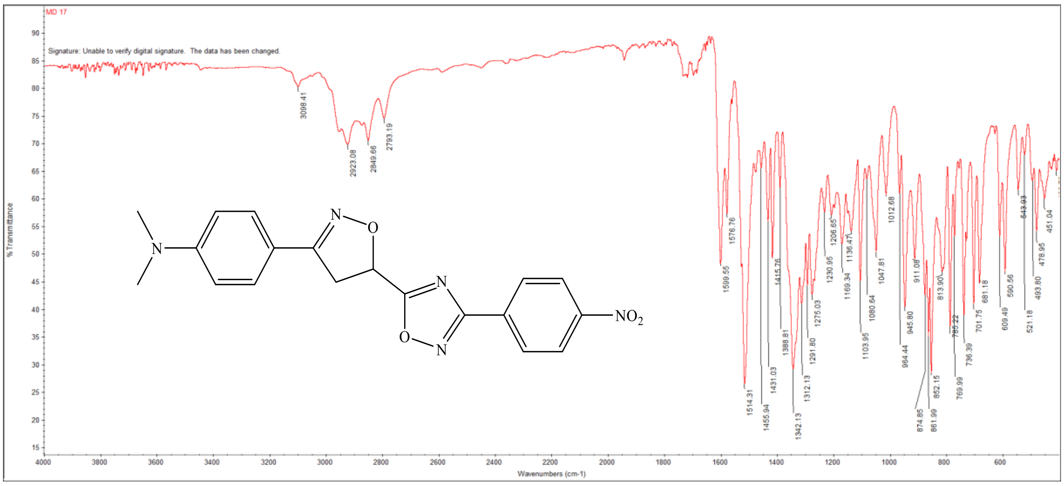

Figure S65. IR Spectrum of compound **7g**

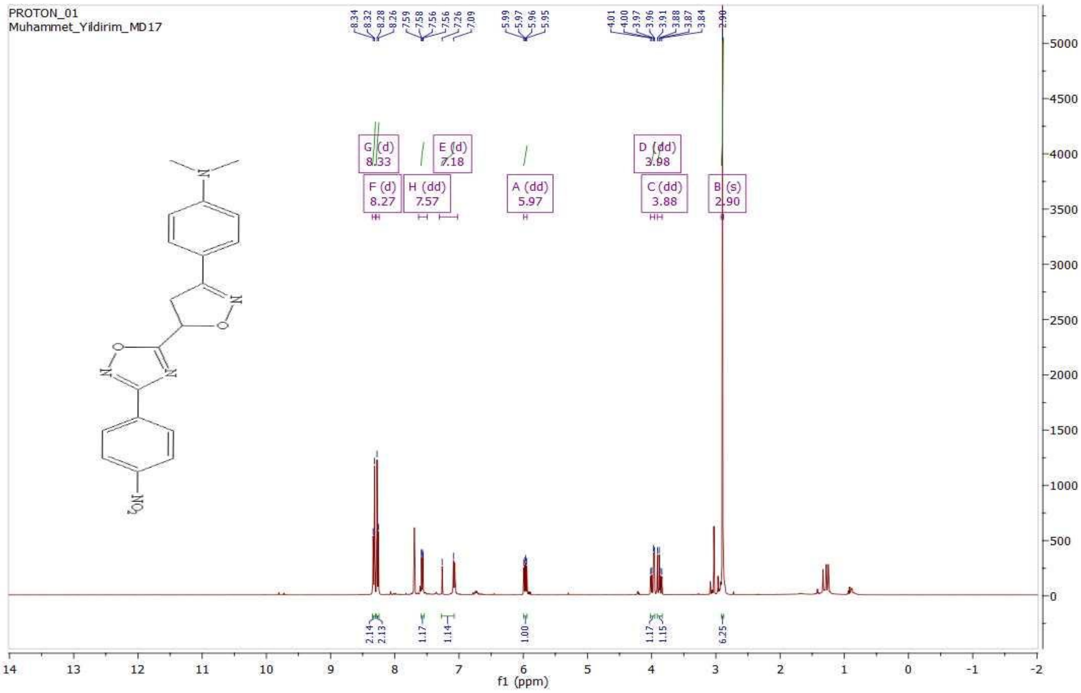

Figure S66. ^{1}H-NMR Spectrum of compound **7g**

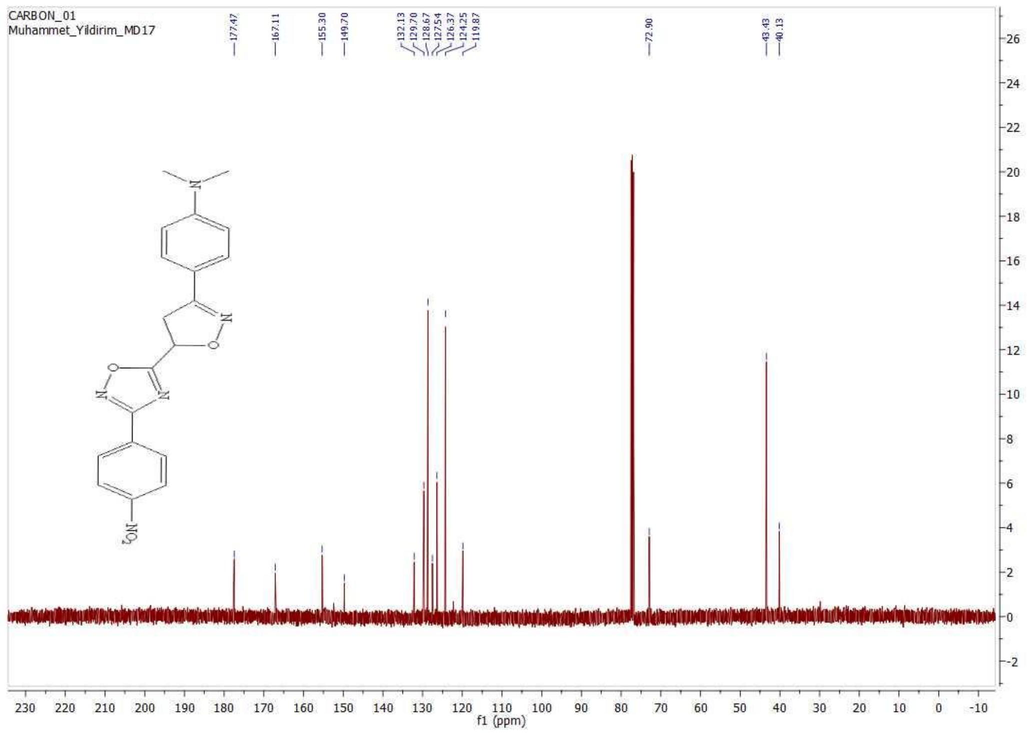

Figure S67. ^{13}C-NMR Spectrum of compound **7g**

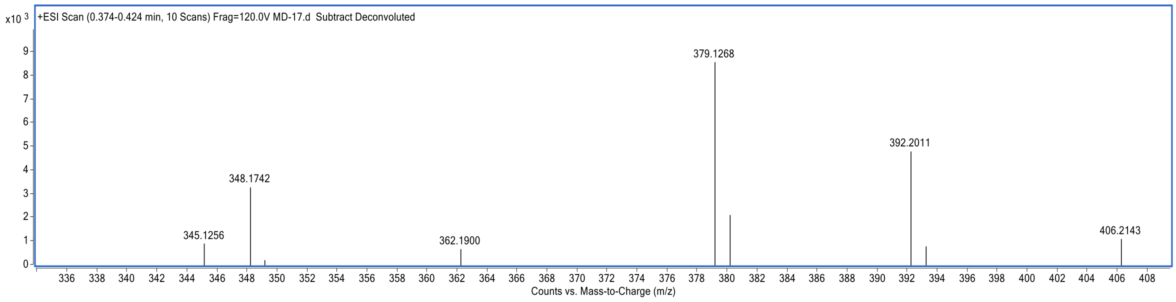

Figure S68. HRMS Spectrum of compound **7g**

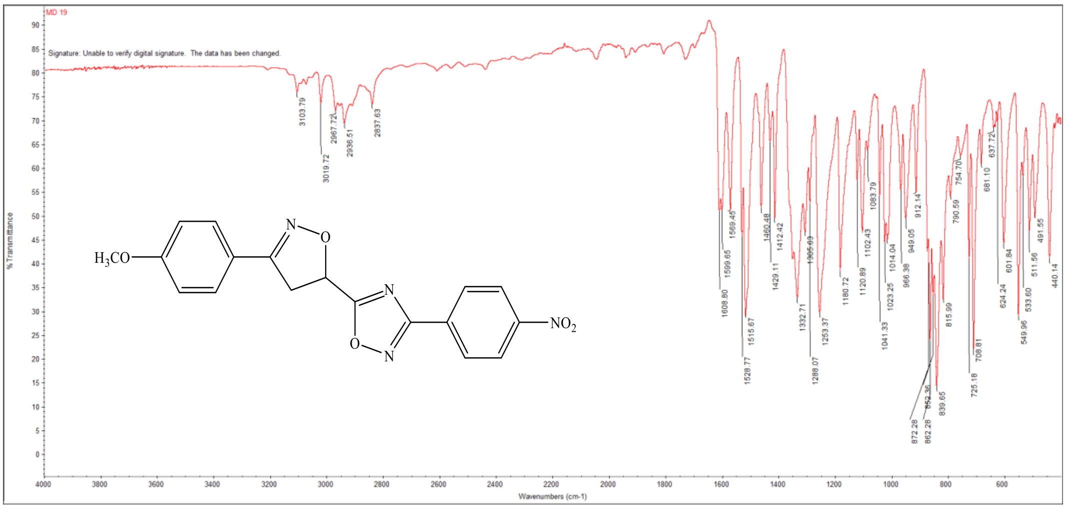

Figure S69. IR Spectrum of compound **7h**

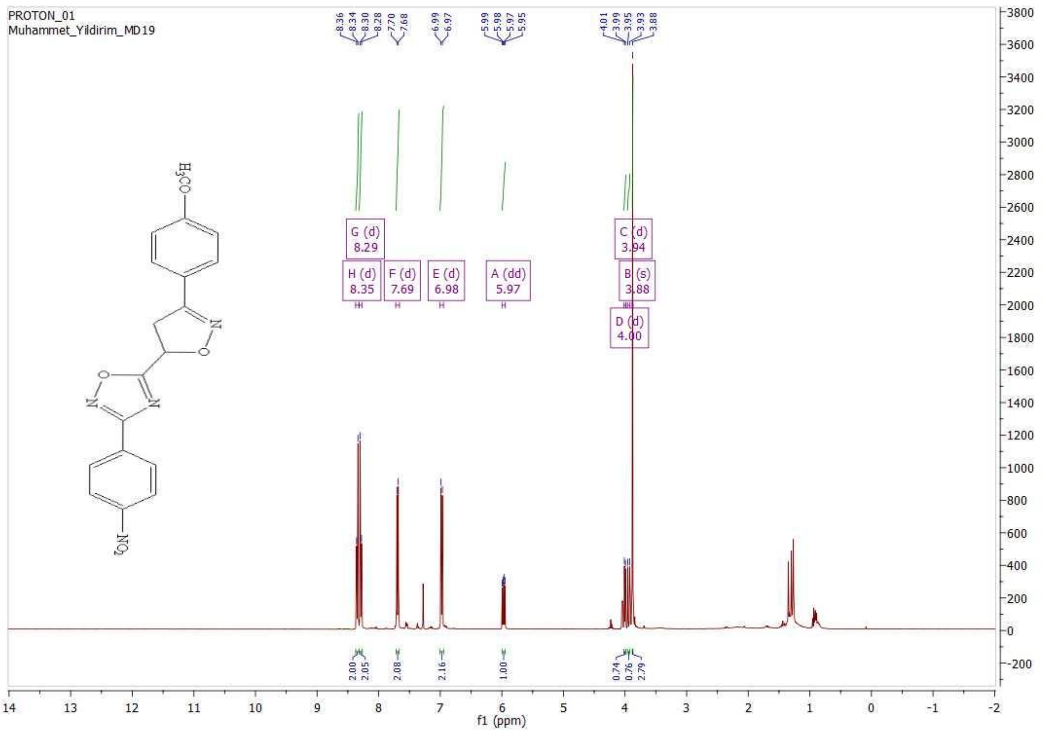

Figure S70. ^{1}H-NMR Spectrum of compound **7h**

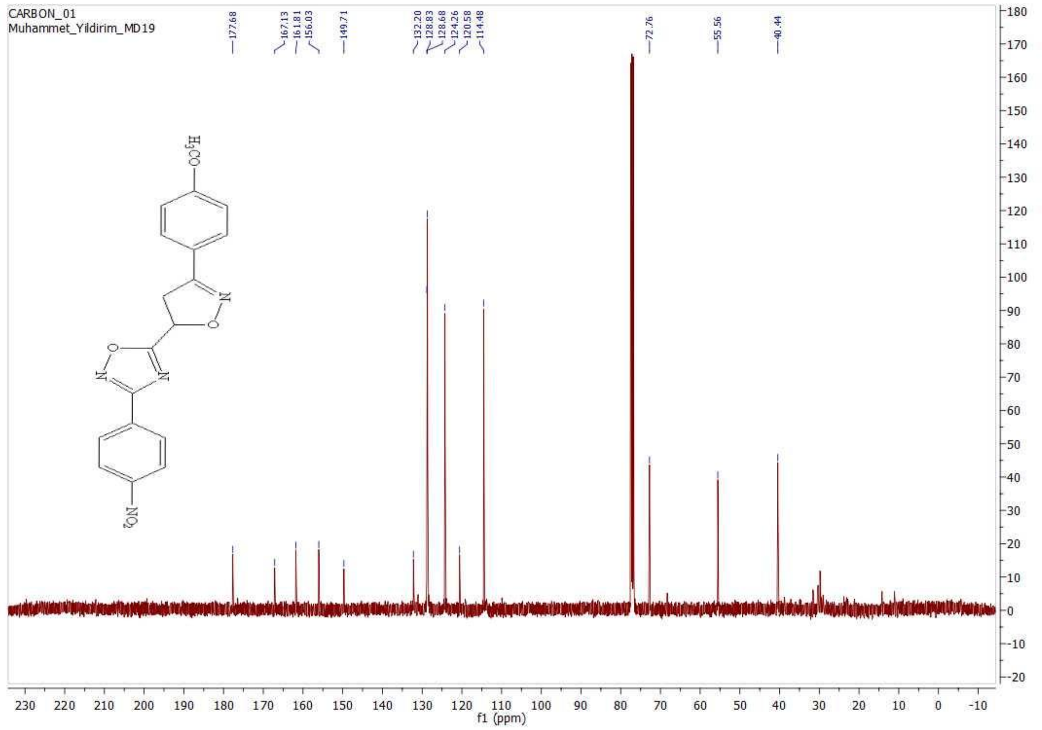

Figure S71. ^{13}C-NMR Spectrum of compound **7h**

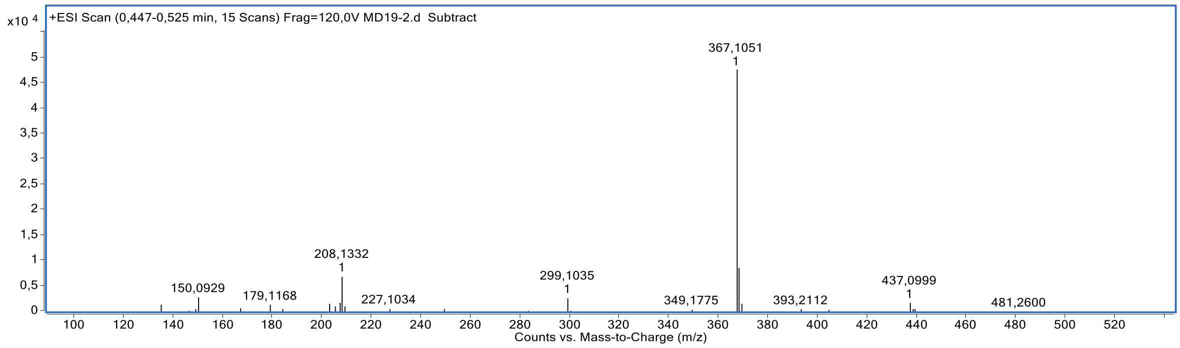

Figure S72. HRMS Spectrum of compound **7h**

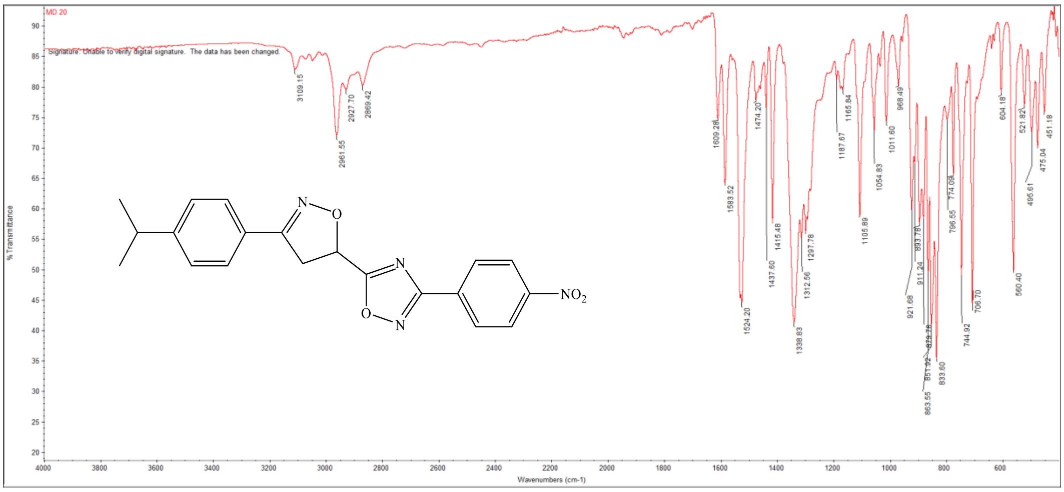

Figure S73. IR Spectrum of compound **7i**

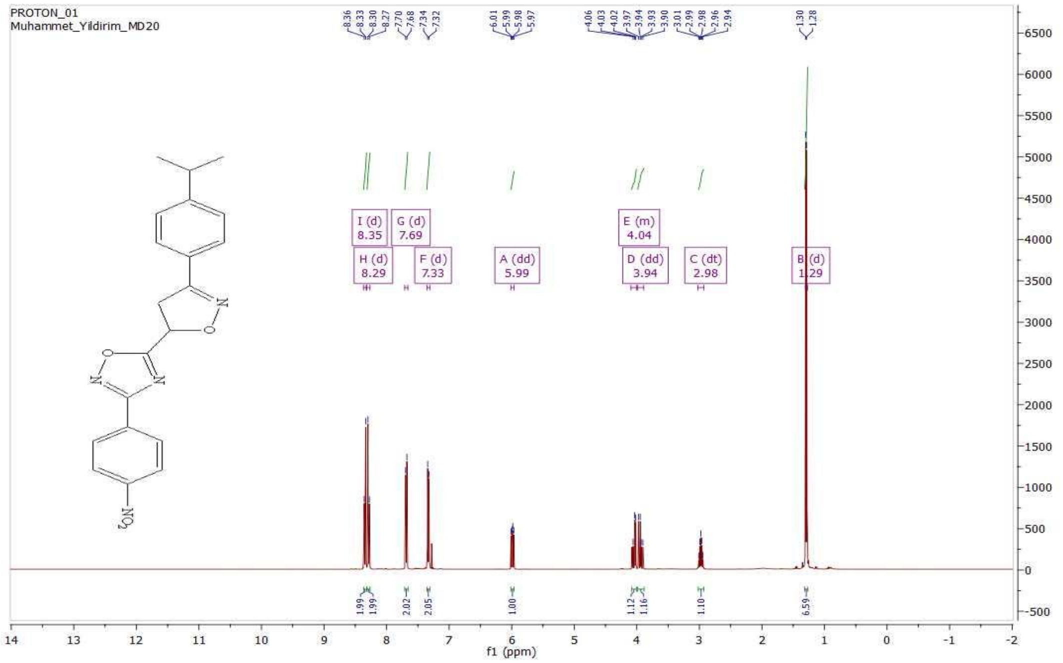

Figure S74. ^{1}H-NMR Spectrum of compound **7i**

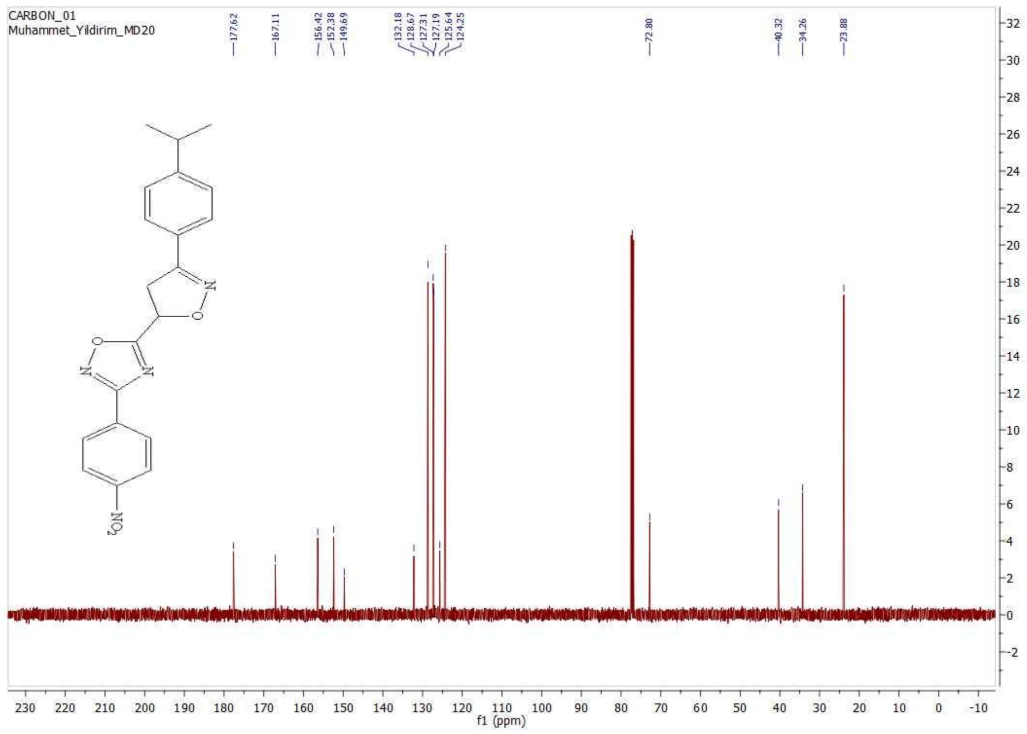

Figure S75. ^{13}C-NMR Spectrum of compound **7i**

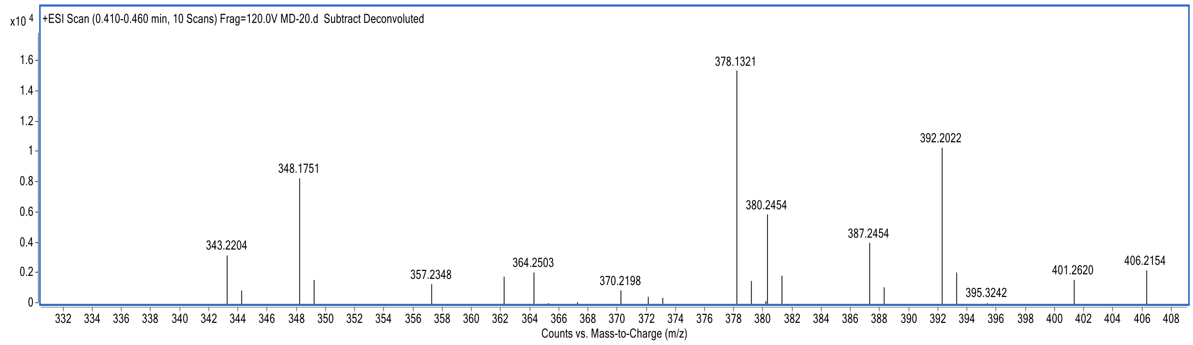

Figure S76. HRMS Spectrum of compound **7i**

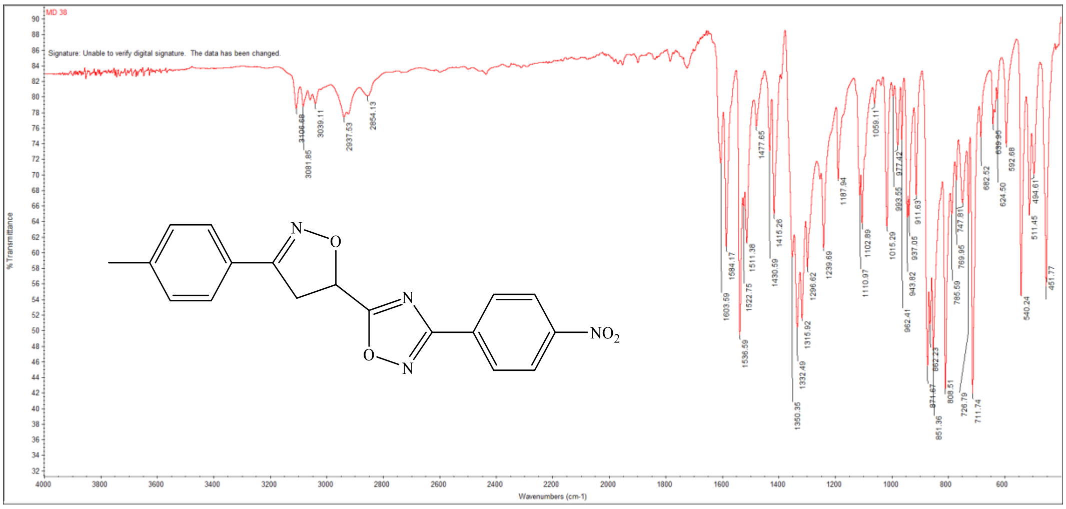

Figure S77. IR Spectrum of compound **7j**

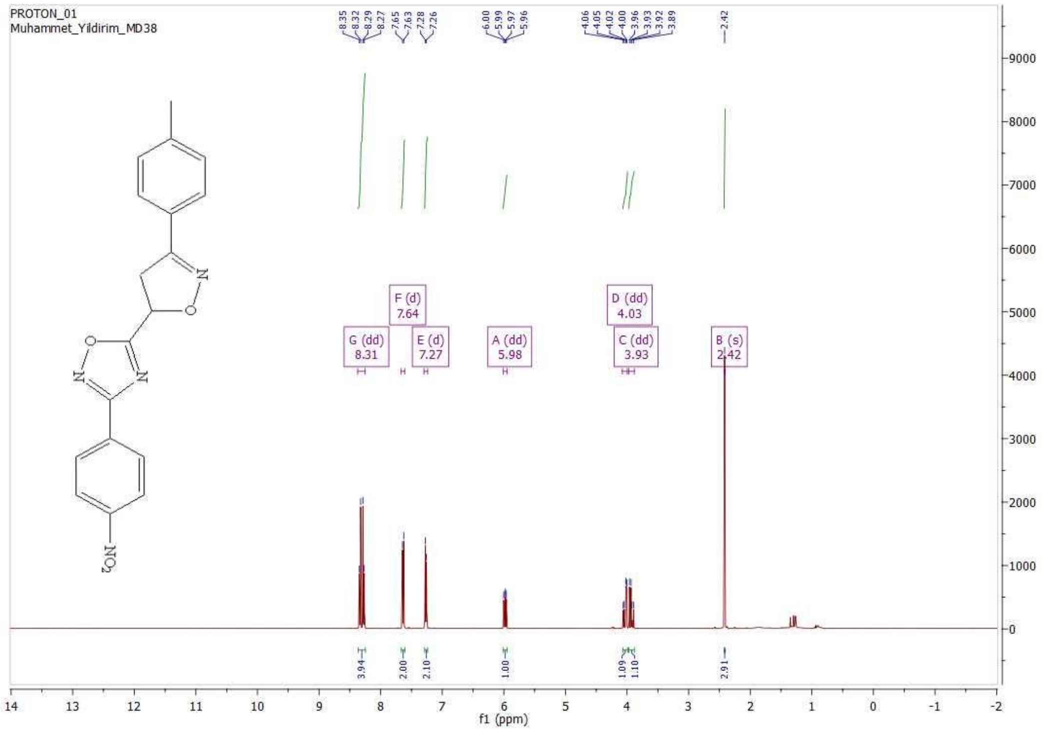

Figure S78. ^{1}H-NMR Spectrum of compound **7j**

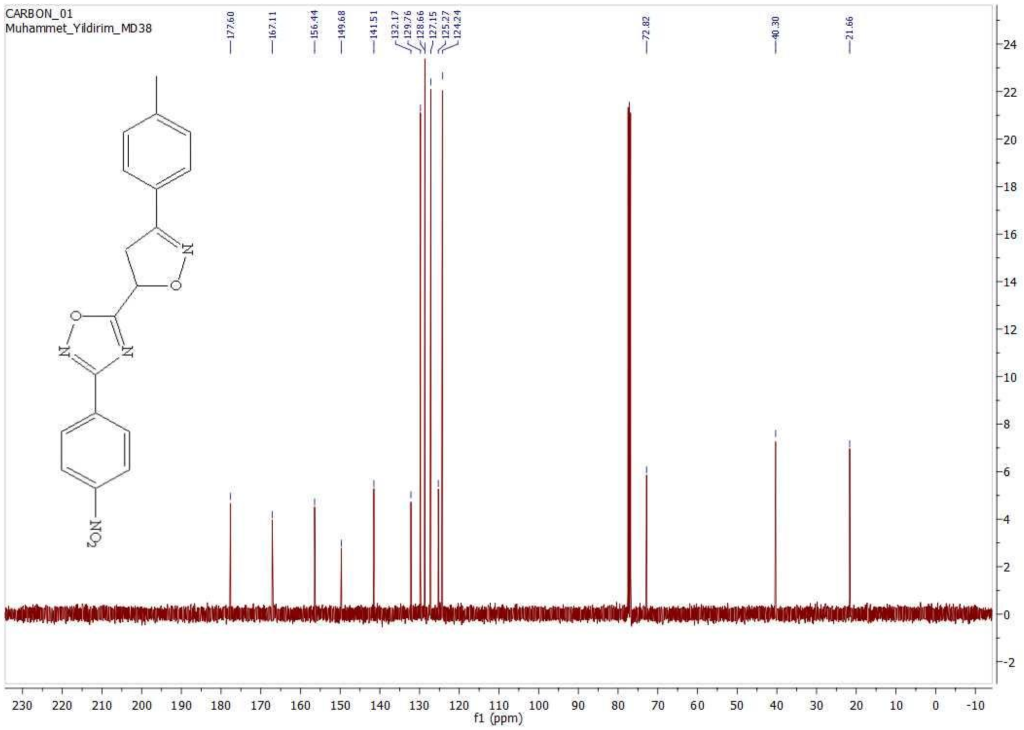

Figure S79. ^{13}C-NMR Spectrum of compound **7j**

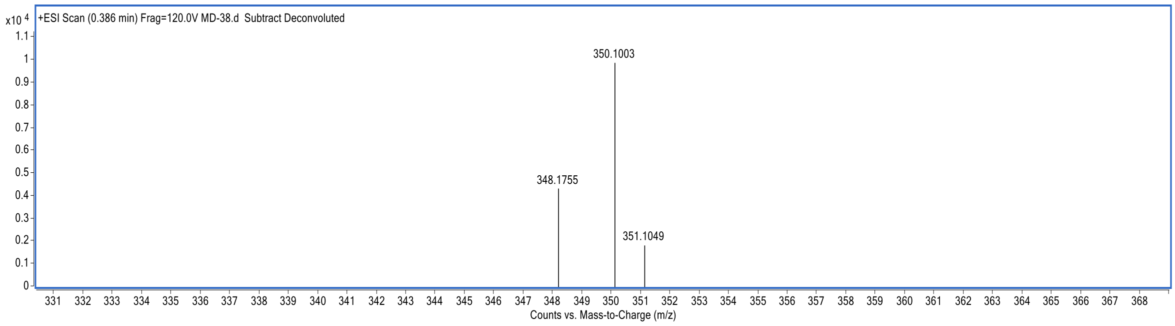

Figure S80. HRMS Spectrum of compound **7j**

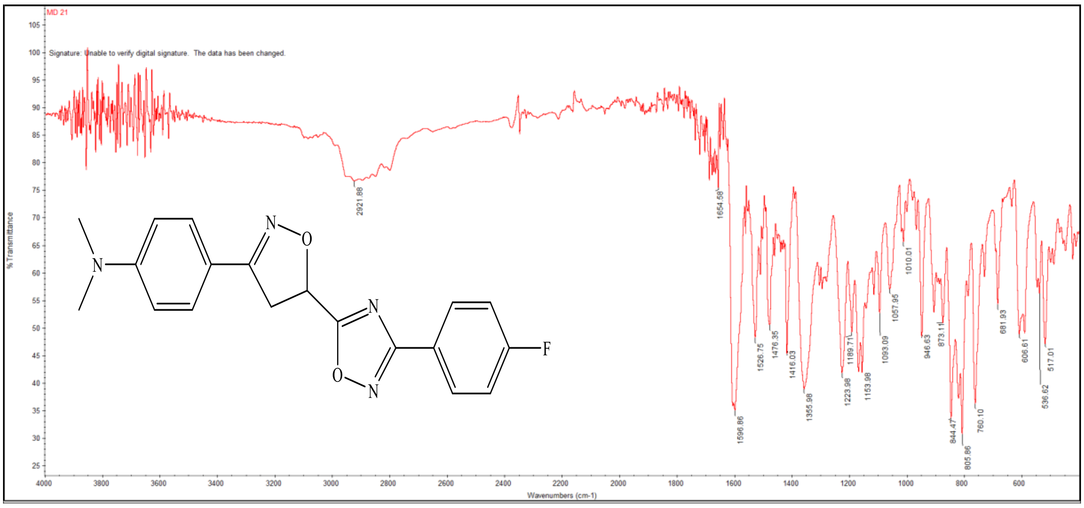

Figure S81. IR Spectrum of compound **7k**

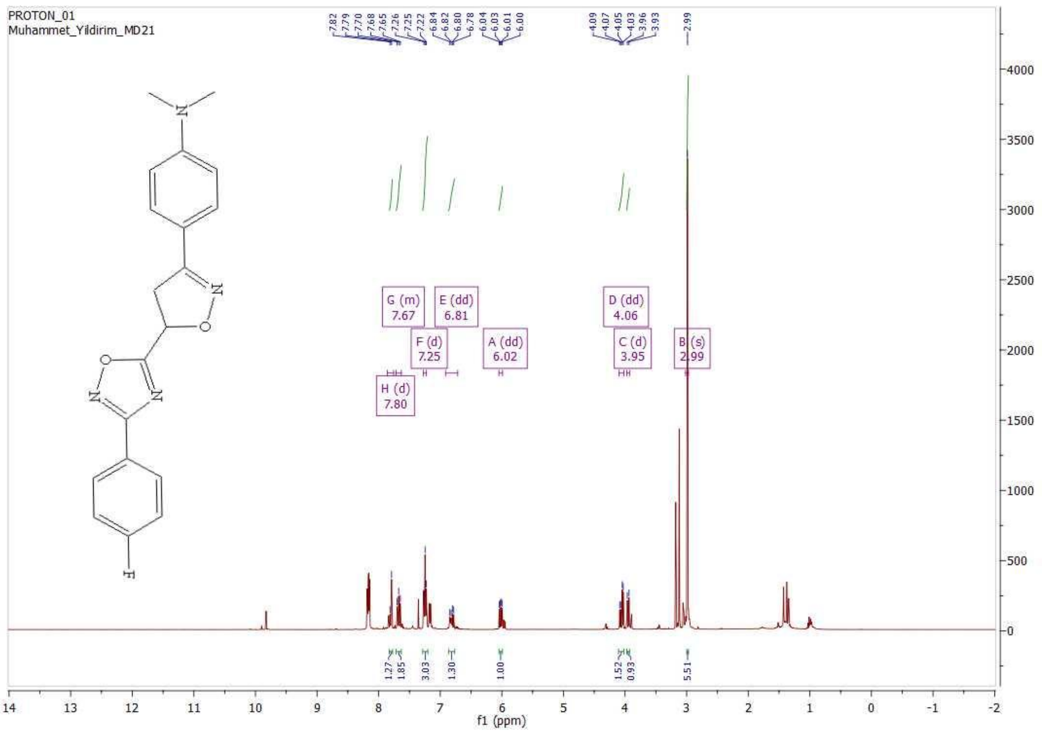

Figure S82. ^{1}H-NMR Spectrum of compound **7k**

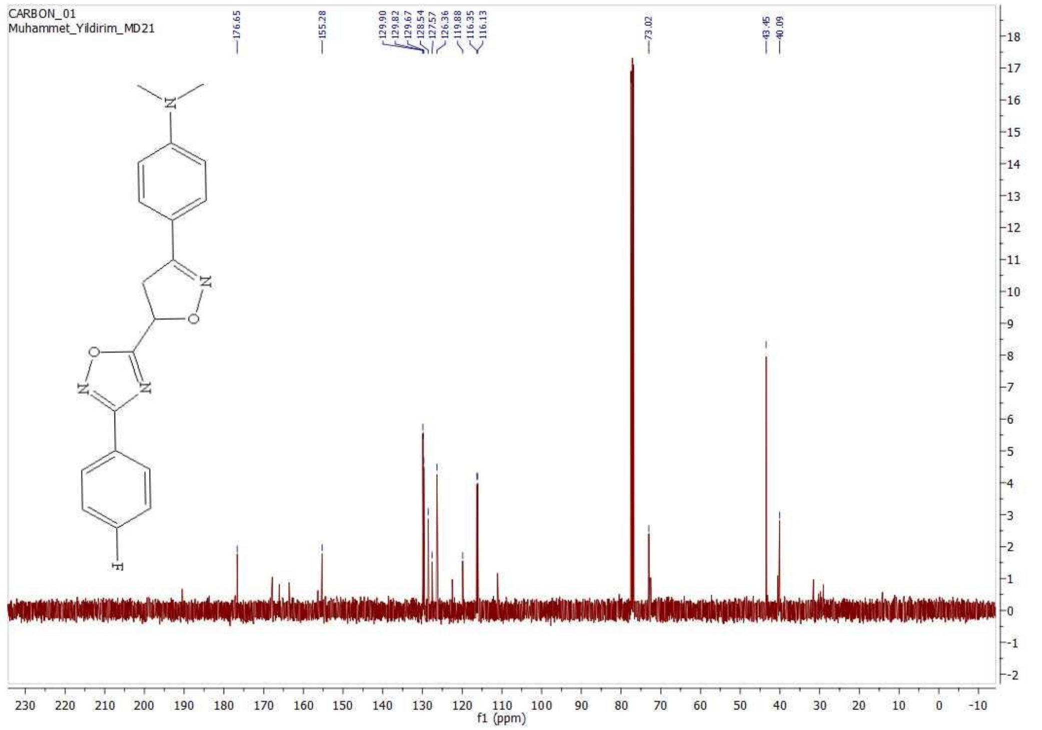

Figure S83. ^{13}C-NMR Spectrum of compound **7k**

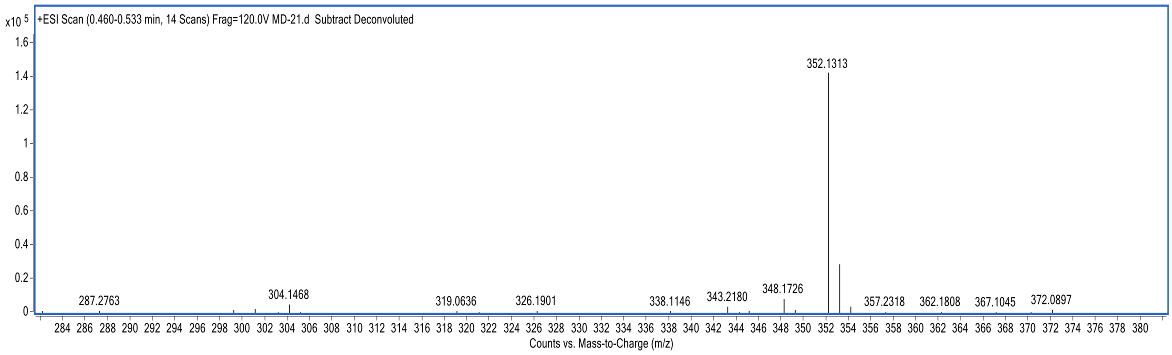

Figure S84. HRMS Spectrum of compound **7k**

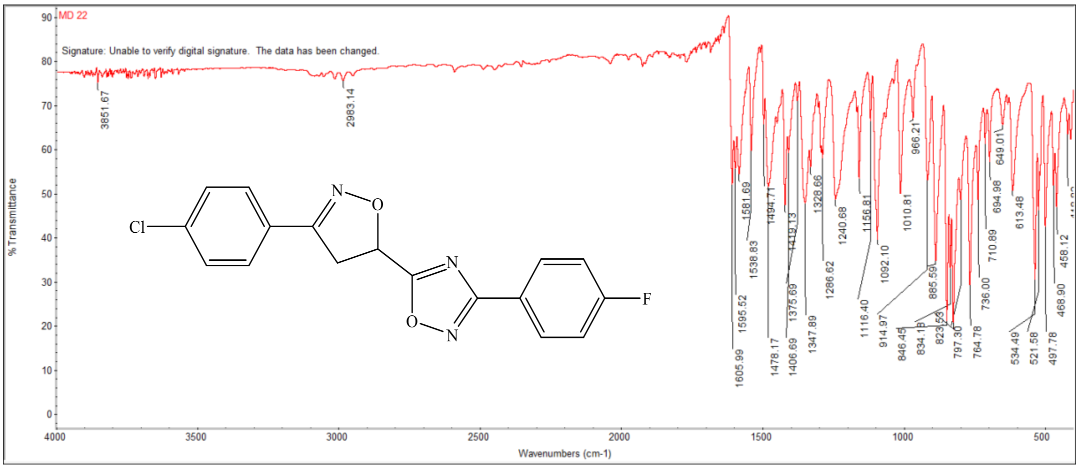

Figure S85. IR Spectrum of compound **7l**

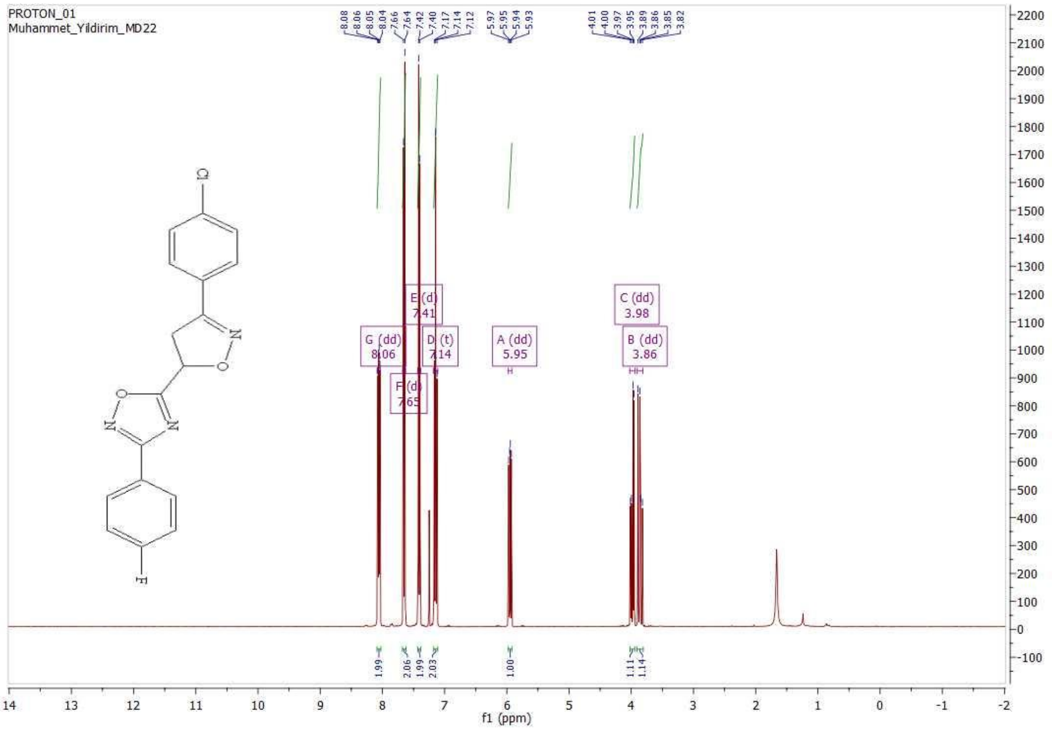

Figure S86. ^{1}H-NMR Spectrum of compound **7l**

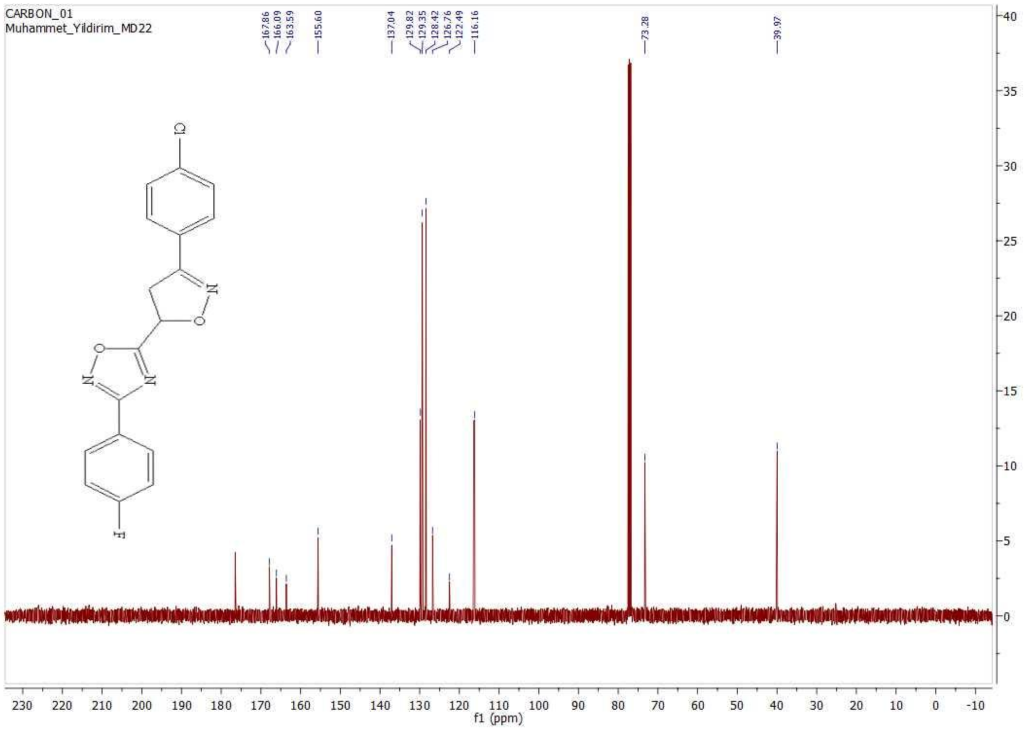

Figure S87. ^{13}C-NMR Spectrum of compound **7l**

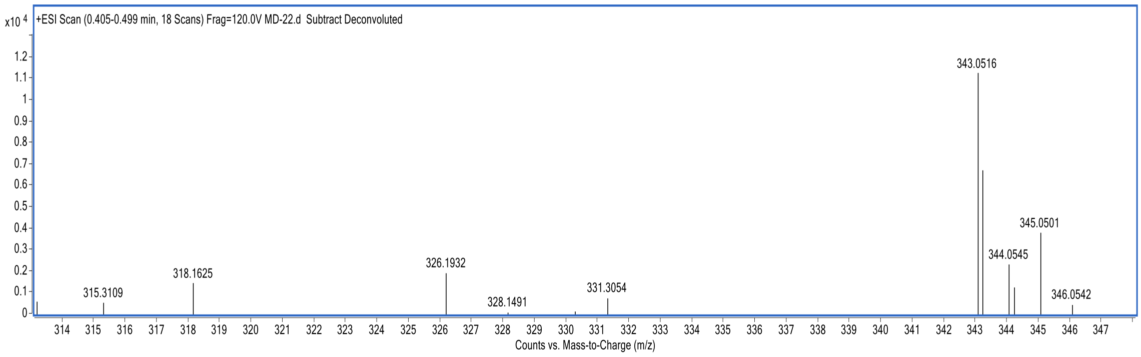

Figure S88. HRMS Spectrum of compound **7l**

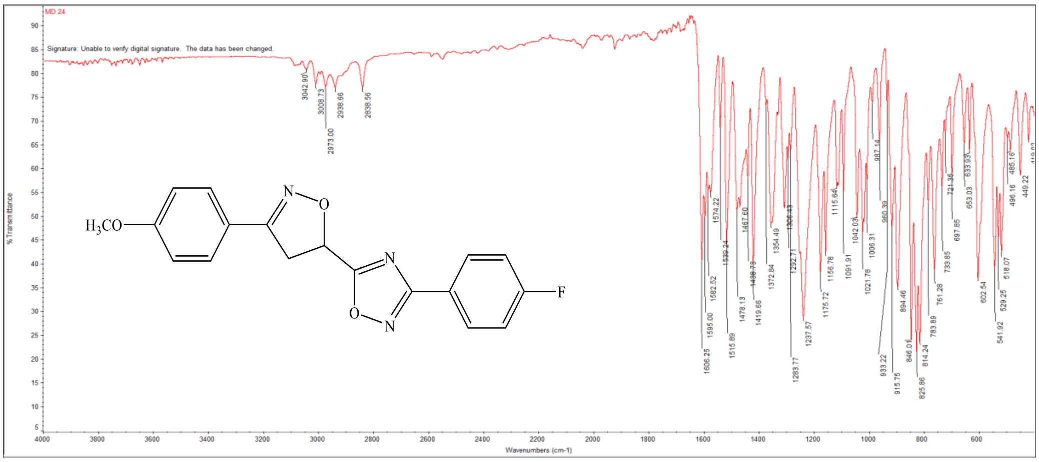

Figure S89. IR Spectrum of compound **7m**

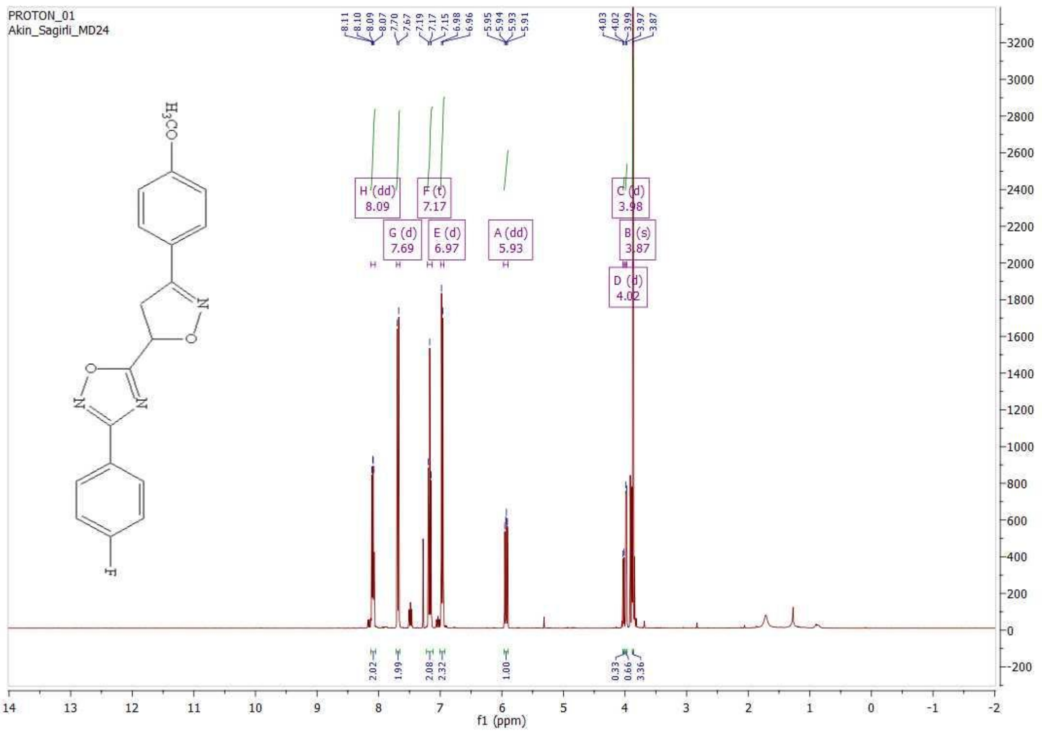

Figure S90. ^{1}H-NMR Spectrum of compound **7m**

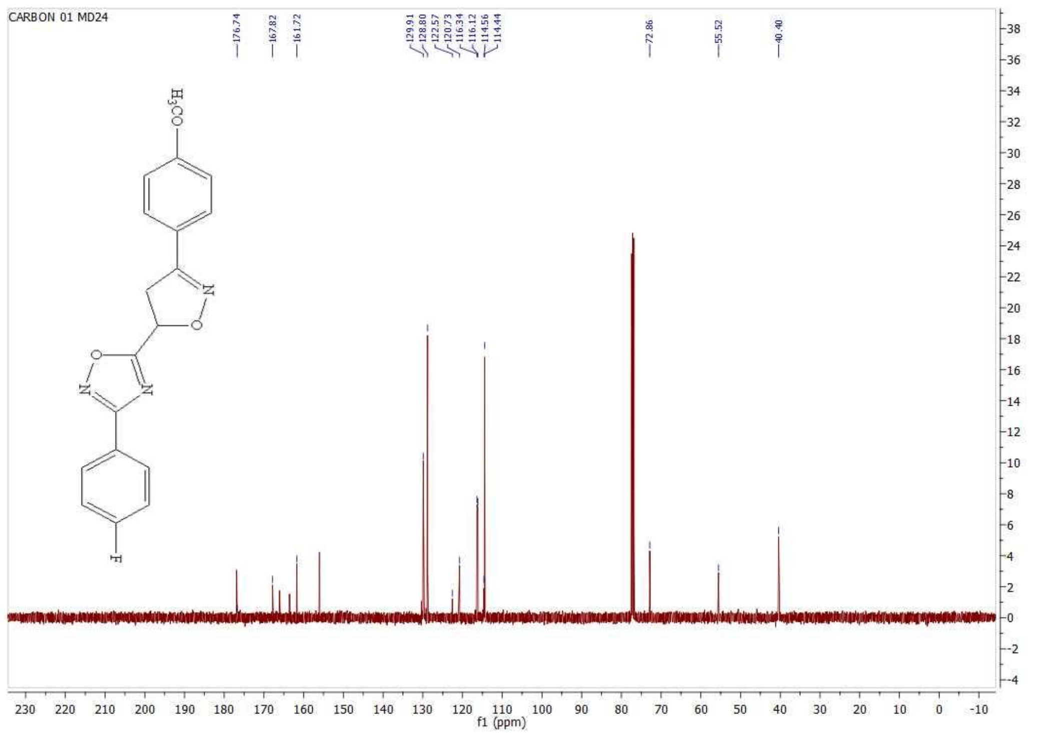

Figure S91.^{13}C-NMR Spectrum of compound **7m**

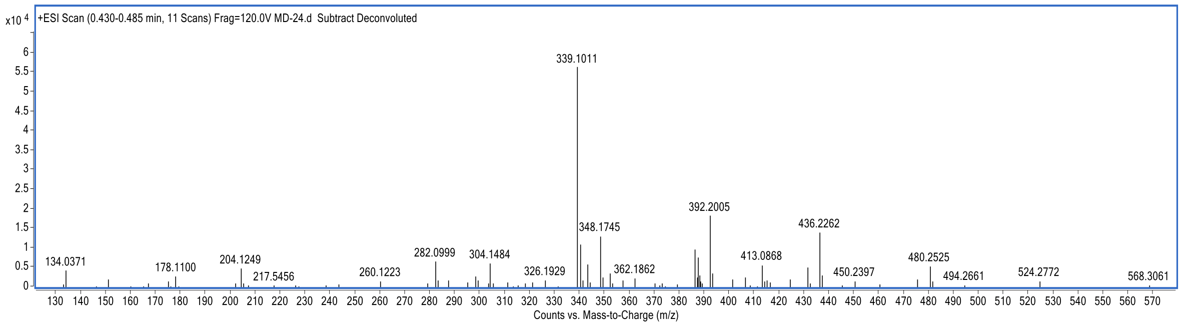

Figure S92. HRMS Spectrum of compound **7m**

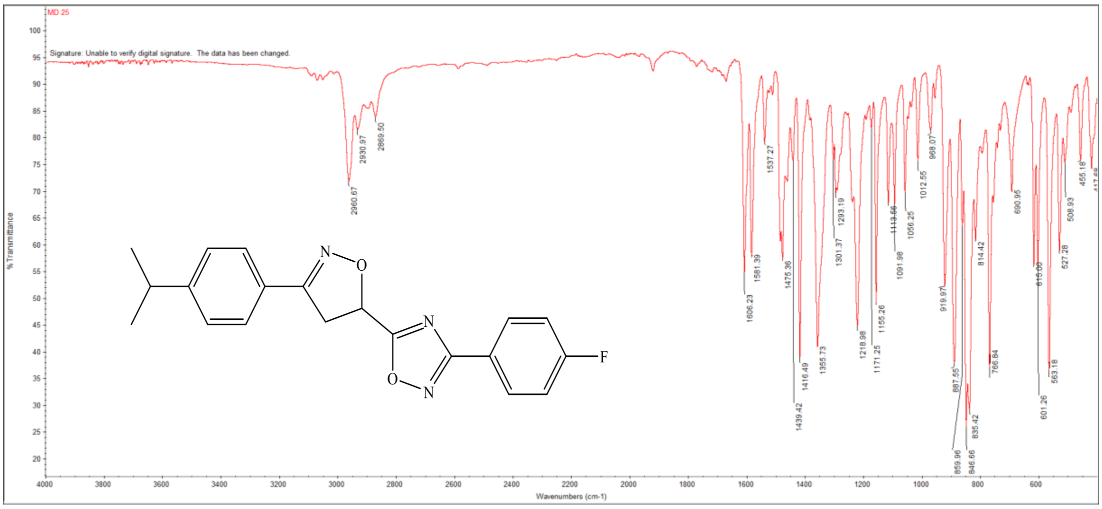

Figure S93. IR Spectrum of compound **7n**

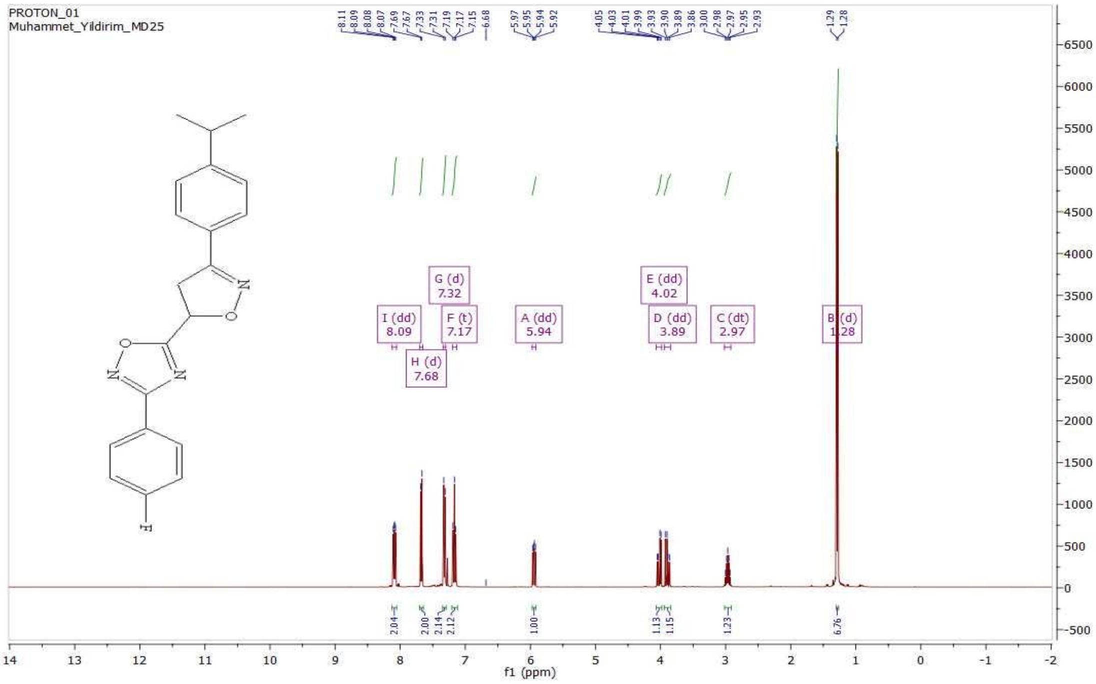

Figure S94. ^{1}H-NMR Spectrum of compound **7n**

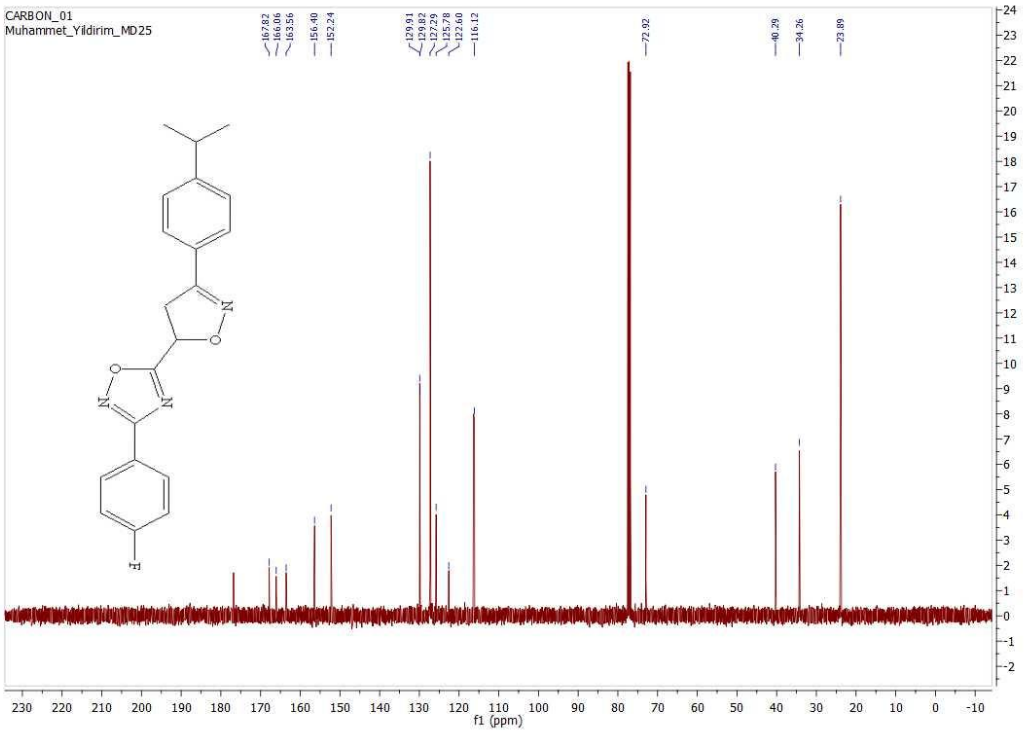

Figure S95. ^{13}C-NMR Spectrum of compound **7n**

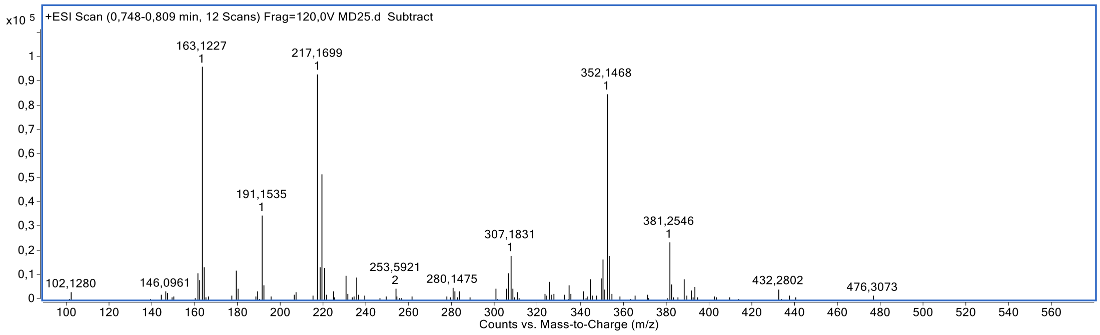

Figure S96. HRMS Spectrum of compound **7n**

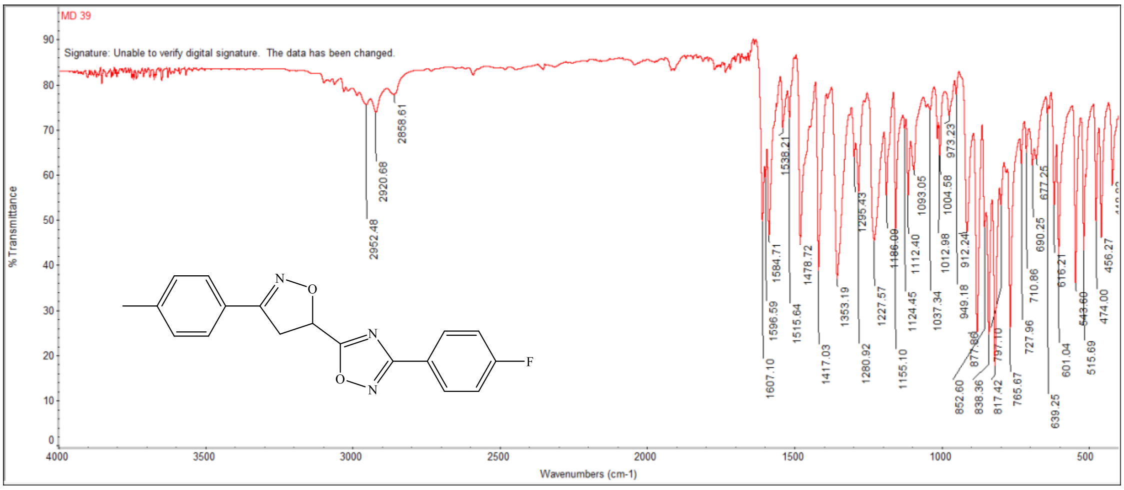

Figure S97. IR Spectrum of compound **7o**

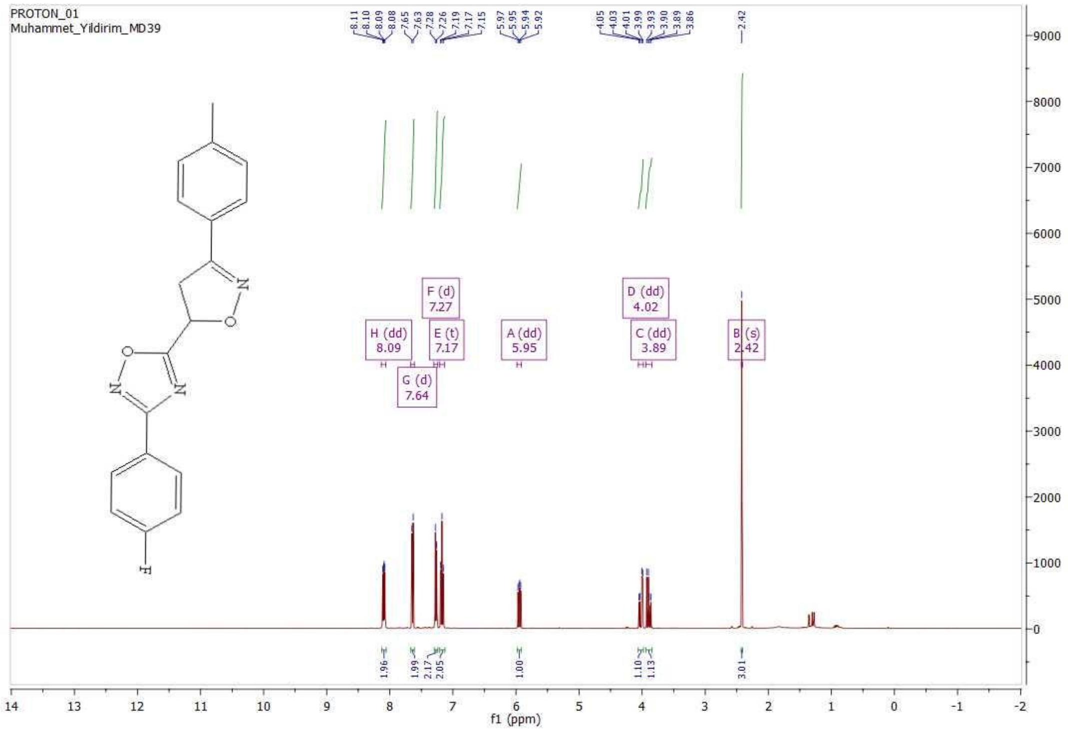

Figure S98. ^{1}H-NMR Spectrum of compound **7o**

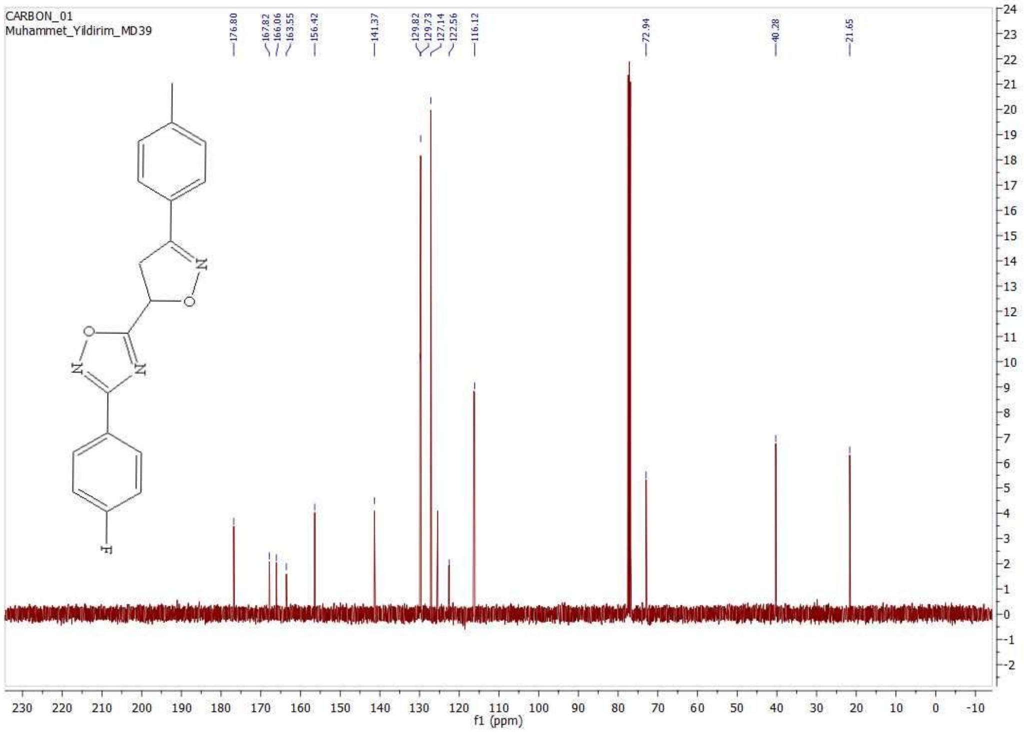

Figure S99. ^{13}C-NMR Spectrum of compound **7o**

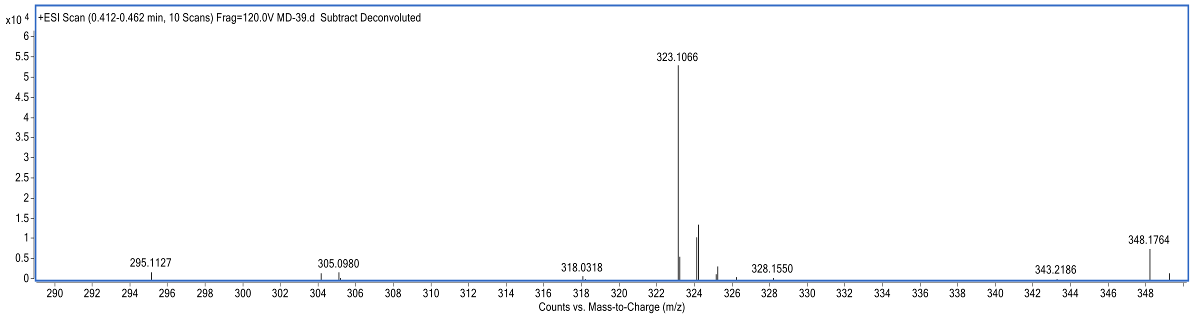

Figure S100. HRMS Spectrum of compound **7o**

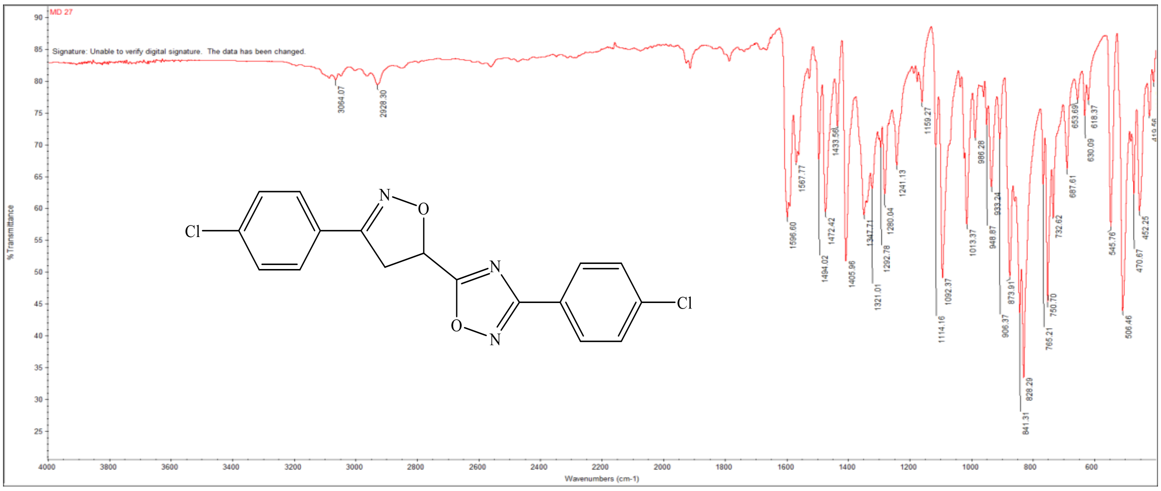

Figure S101. IR Spectrum of compound **7p**

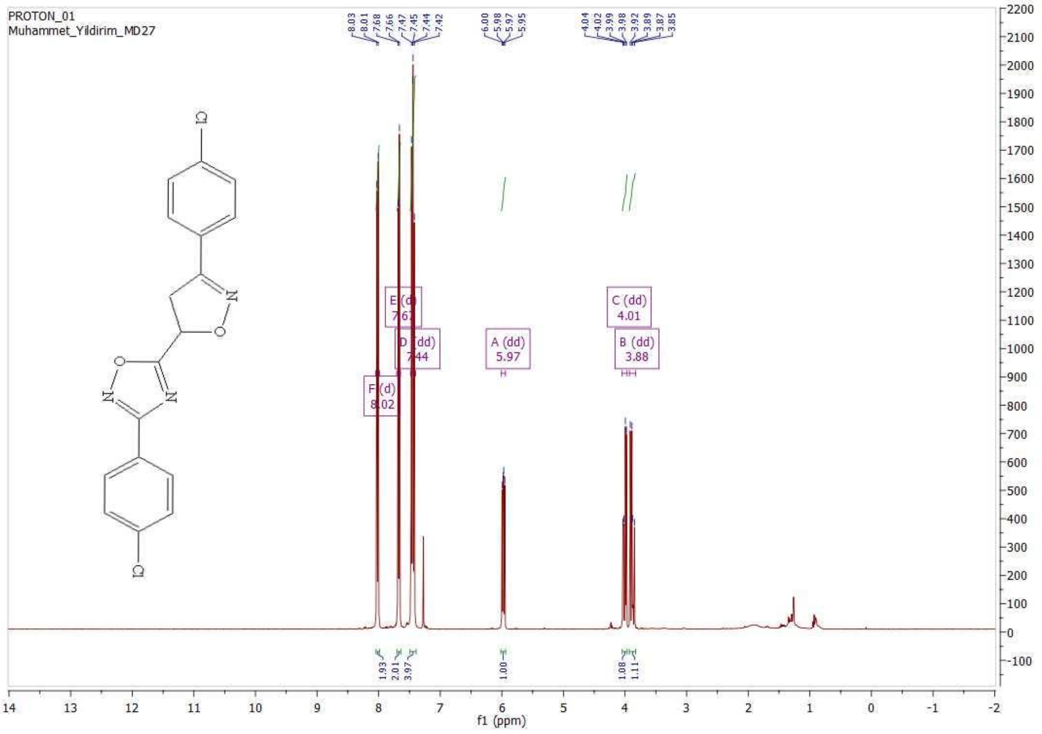

Figure S102. ^{1}H-NMR Spectrum of compound **7p**

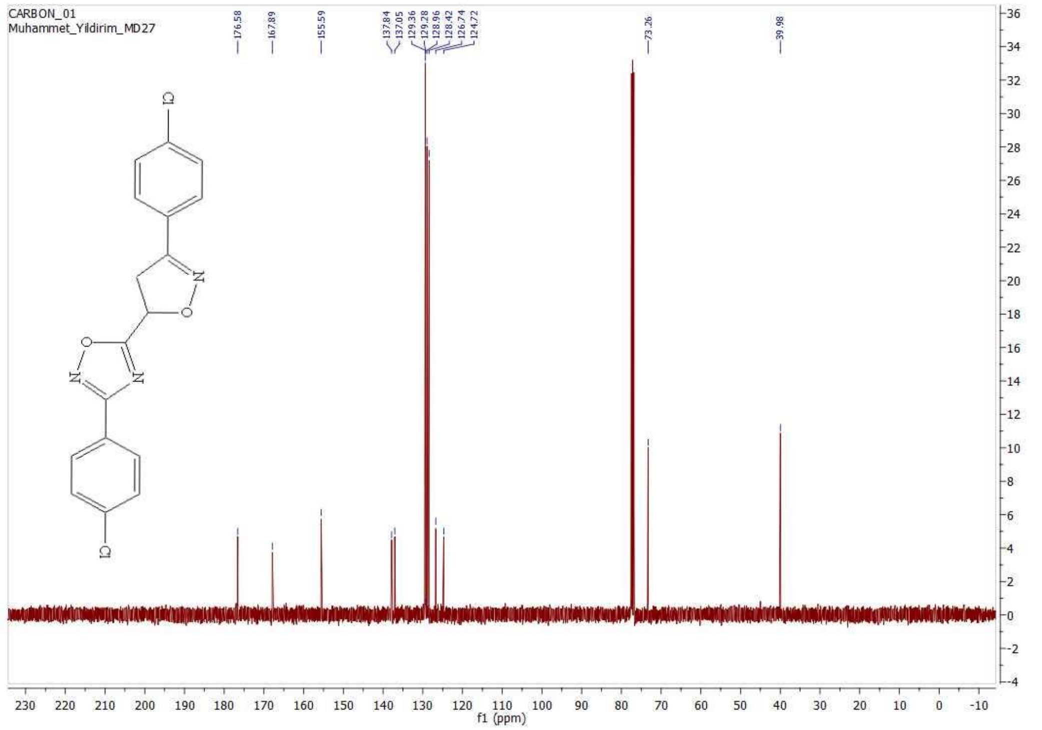

Figure S103. ^{13}C-NMR Spectrum of compound **7p**

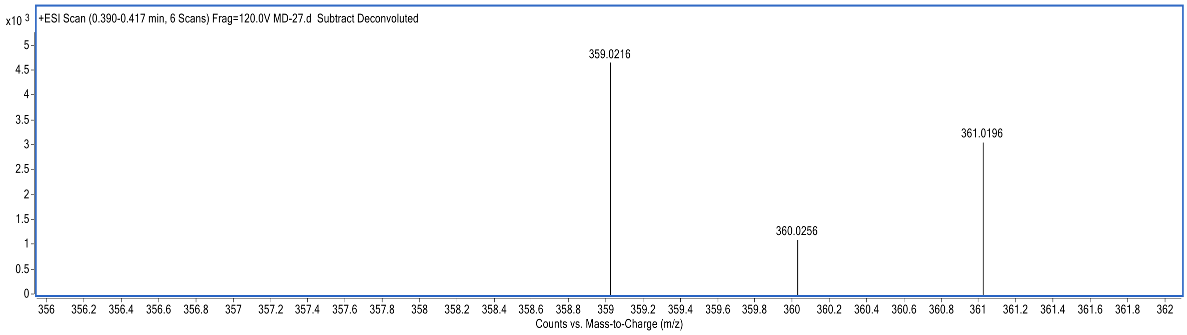

Figure S104. HRMS Spectrum of compound **7p**

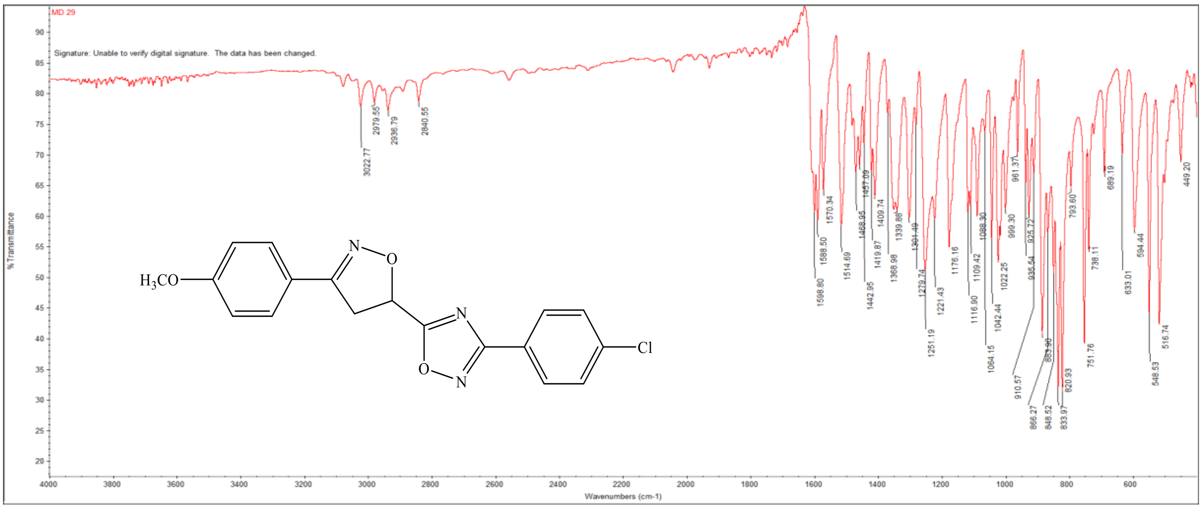

Figure 105. IR Spectrum of compound **7q**

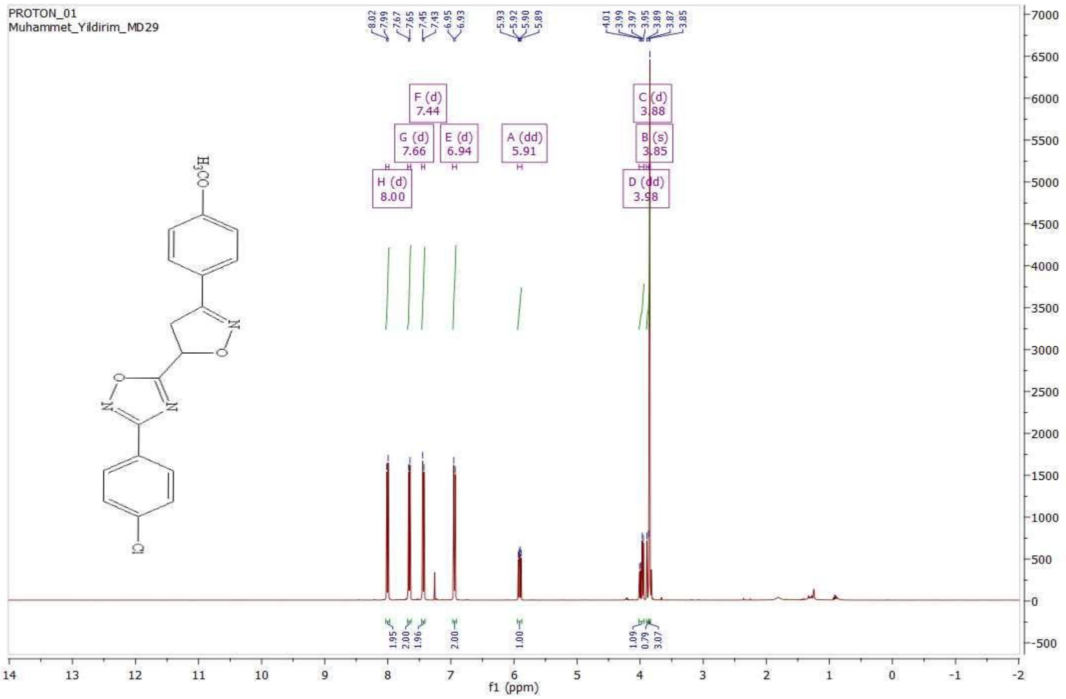

Figure S106. ^{1}H-NMR Spectrum of compound **7q**

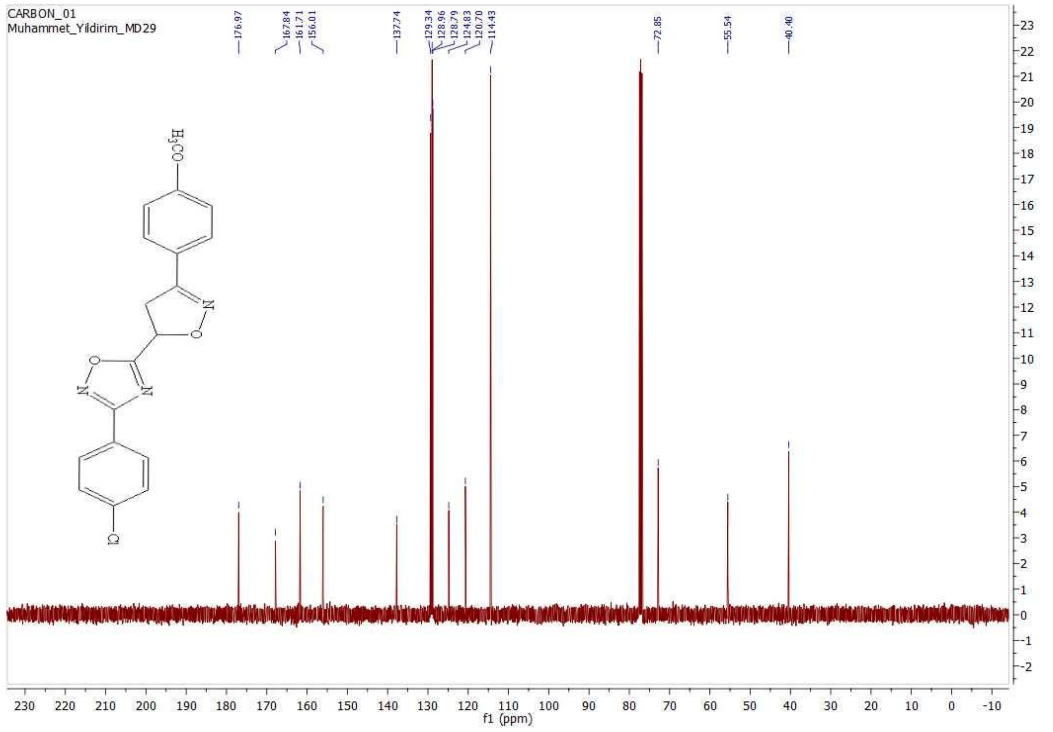

Figure S107. ^{13}C-NMR Spectrum of compound **7q**

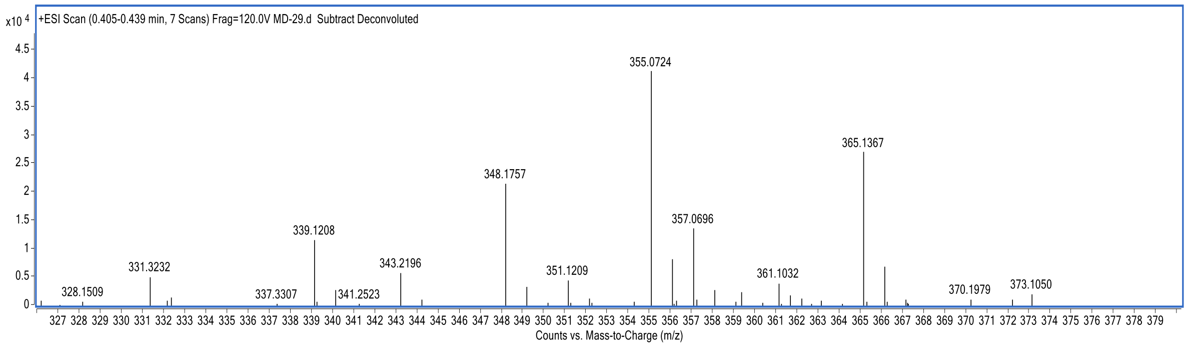

Figure S108. HRMS Spectrum of compound **7q**

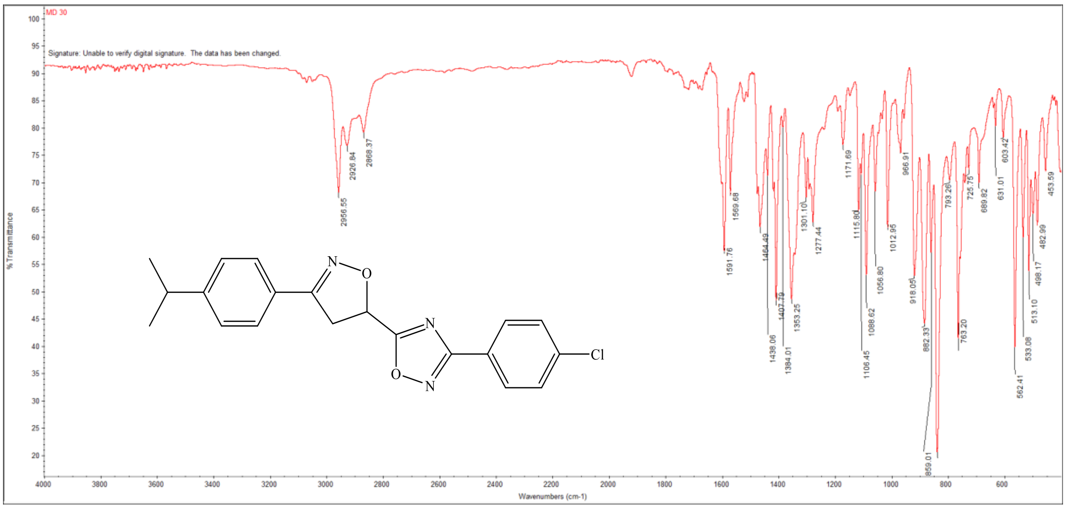

Figure S109. IR Spectrum of compound **7r**

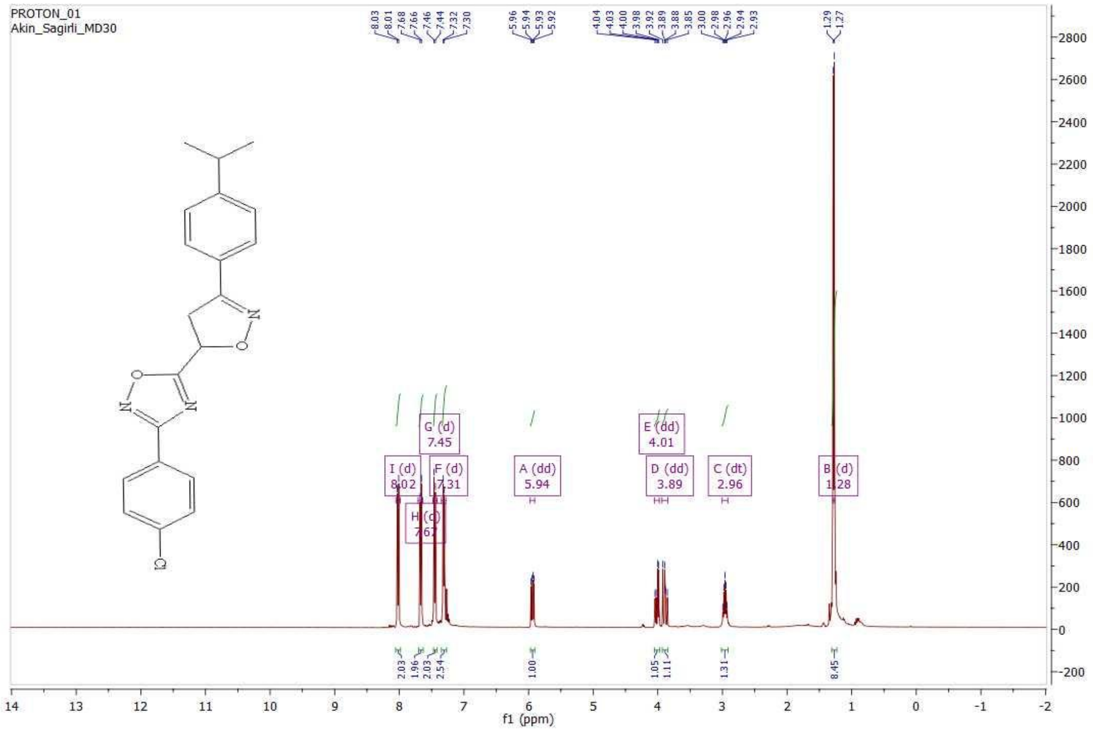

Figure S110. ^{1}H-NMR Spectrum of compound **7r**

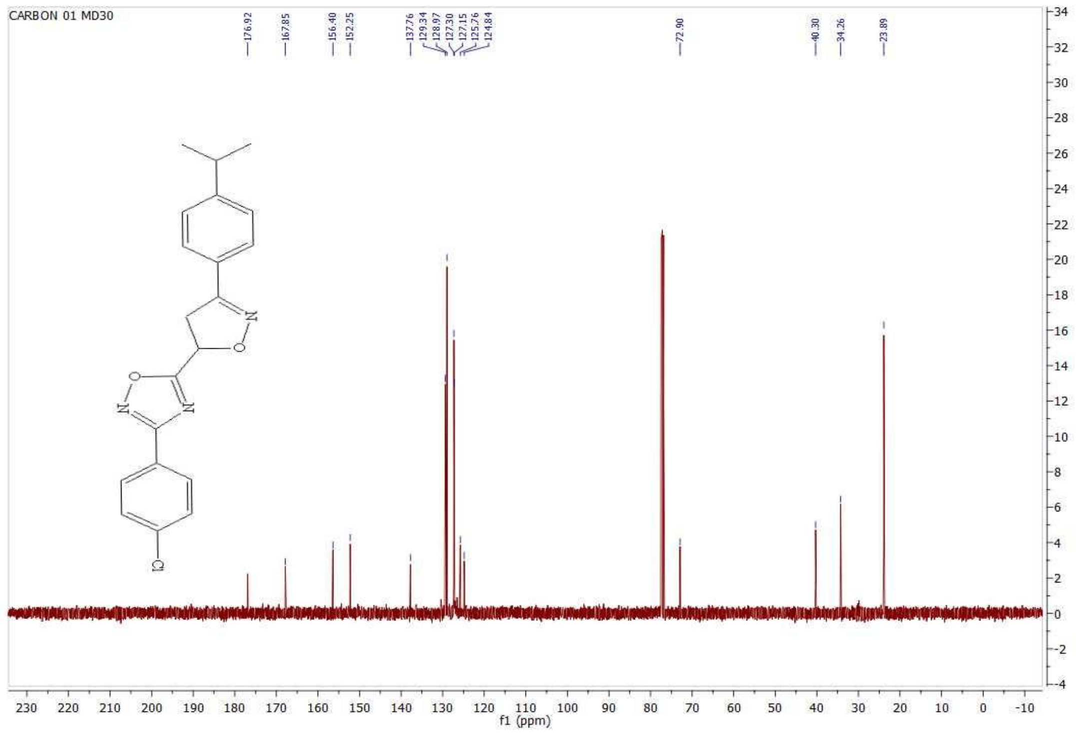

Figure S111. ^{13}C-NMR Spectrum of compound **7r**

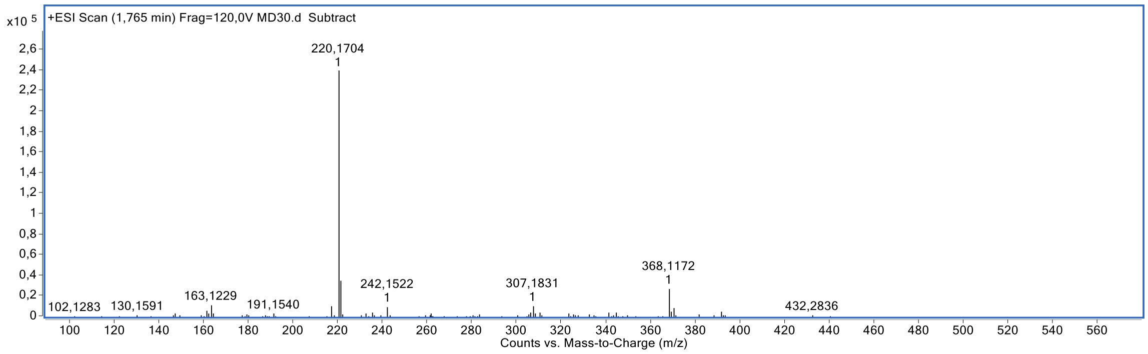

Figure S112. HRMS Spectrum of compound **7r**

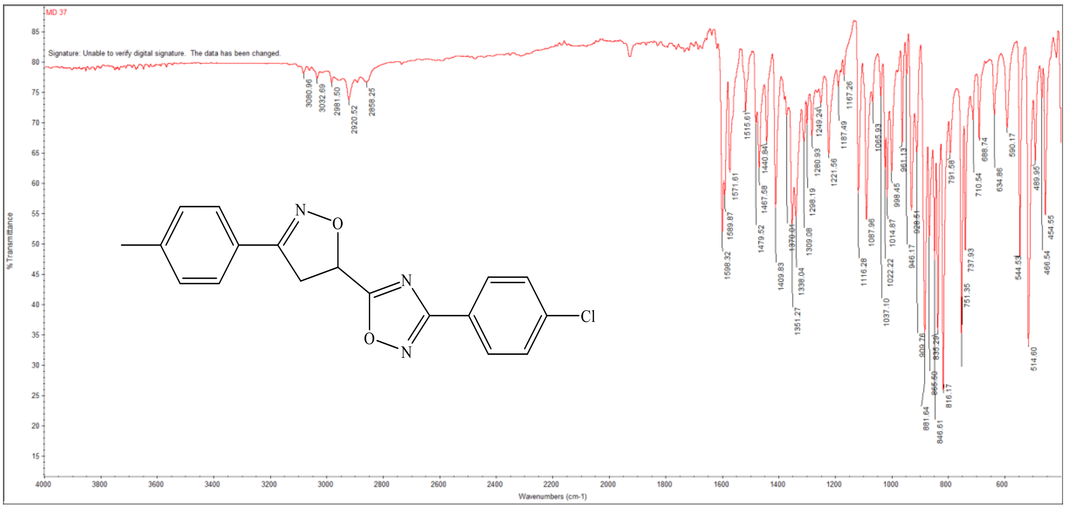

Figure S113. IR Spectrum of compound **7s**

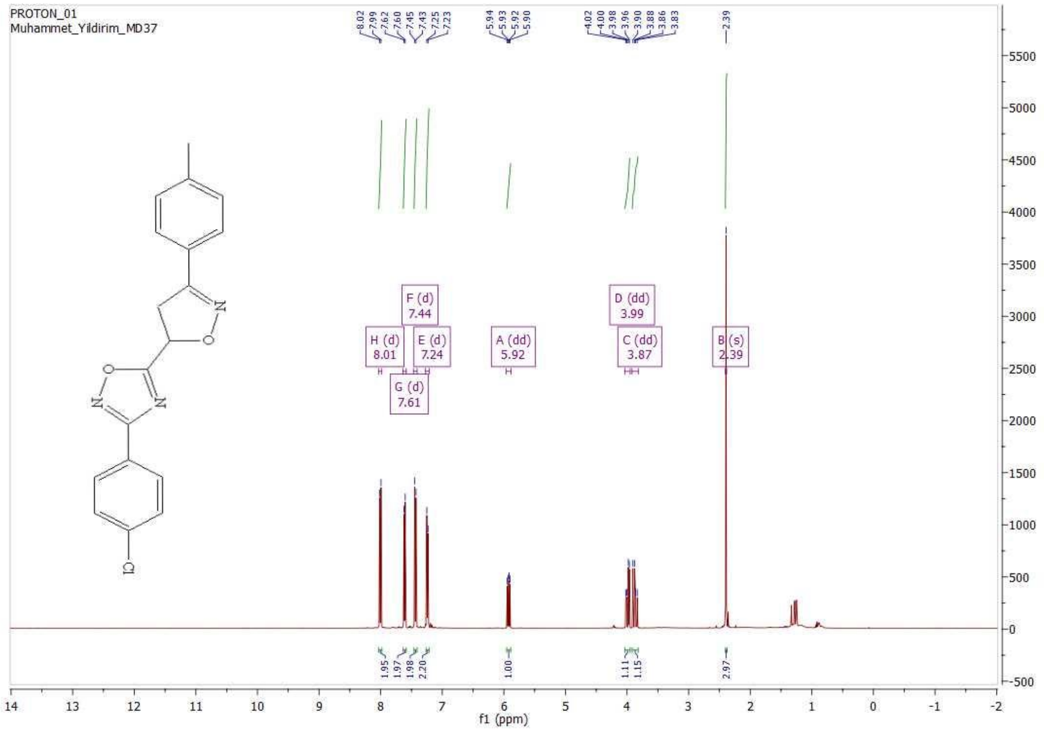

Figure S114. ^{1}H-NMR Spectrum of compound **7s**

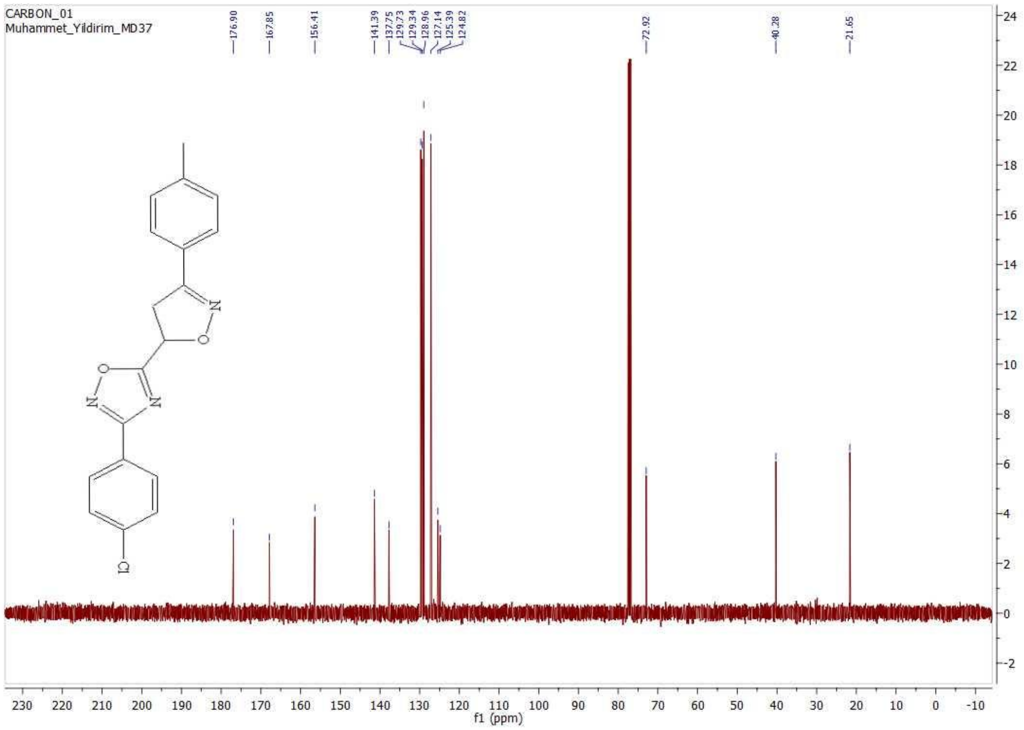

Figure S115. ^{13}C-NMR Spectrum of compound **7s**

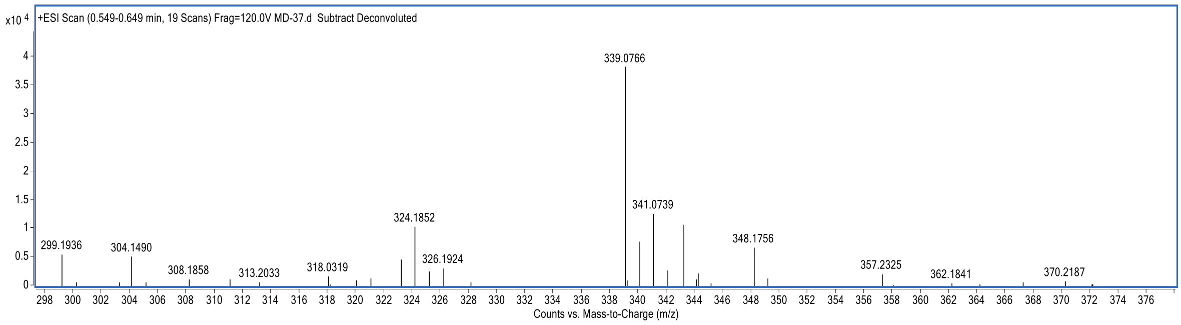

Figure S116. HRMS Spectrum of compound **7s**

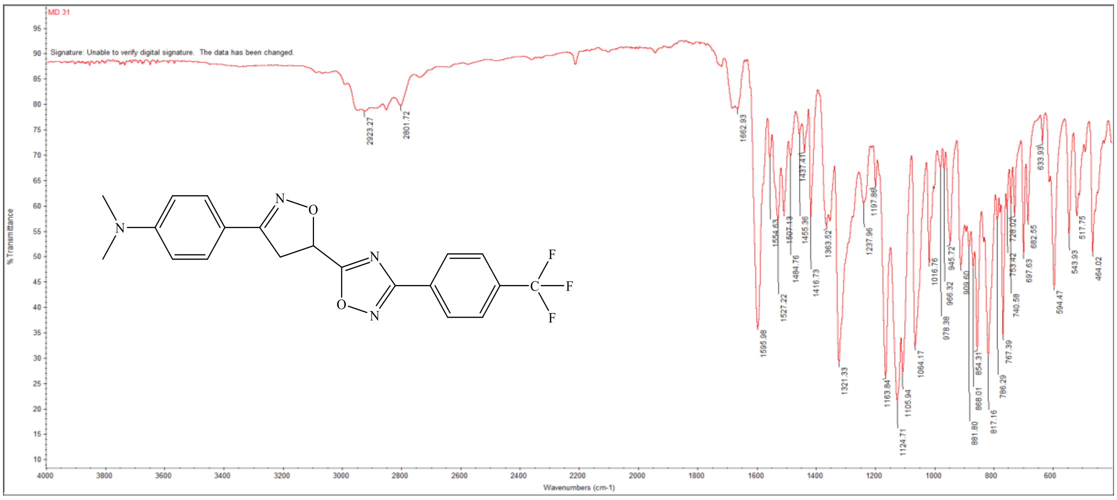

Figure S117. IR Spectrum of compound **7t**

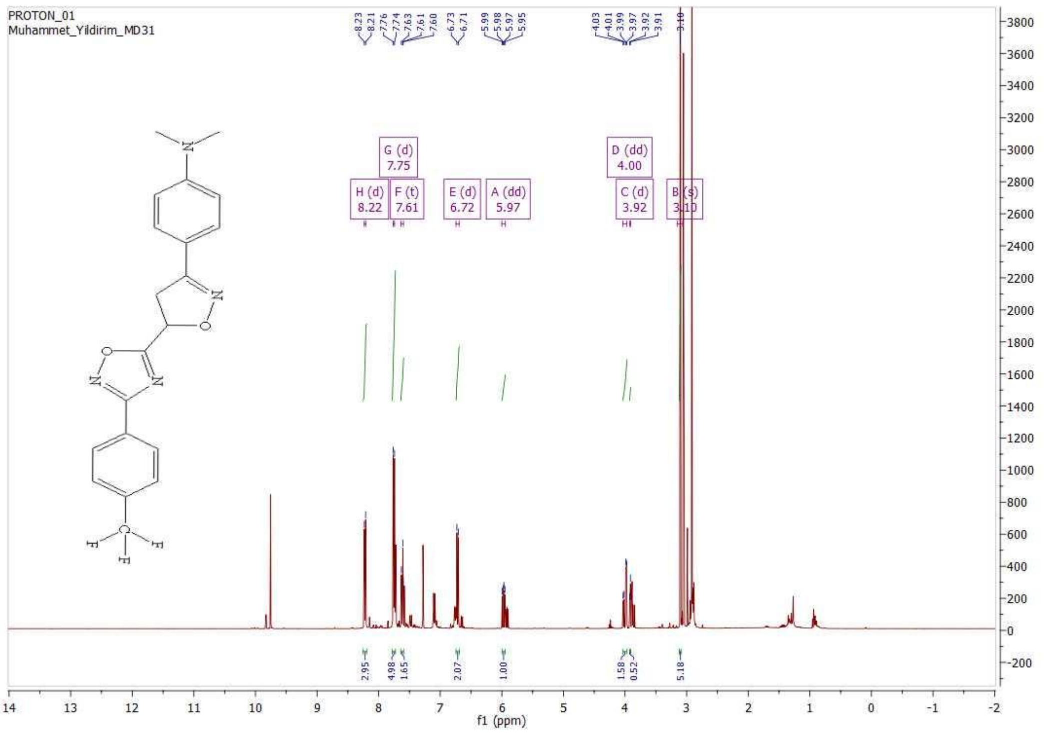

Figure S118. ^{1}H-NMR Spectrum of compound **7t**

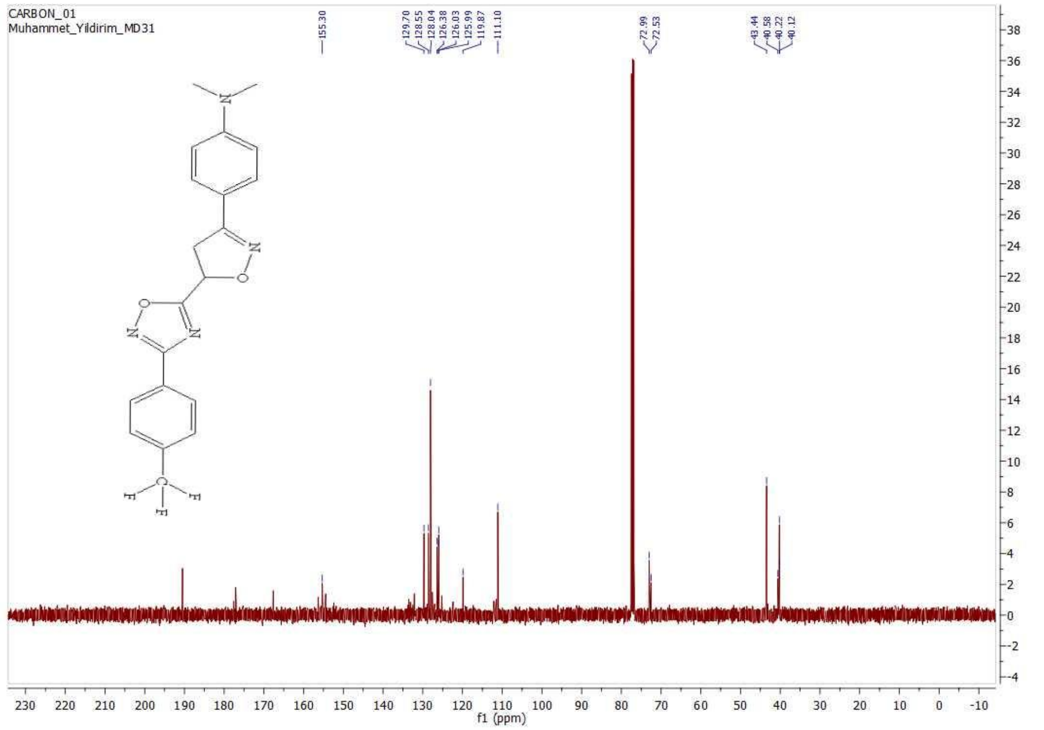

Figure S119. ^{13}C-NMR Spectrum of compound **7t**

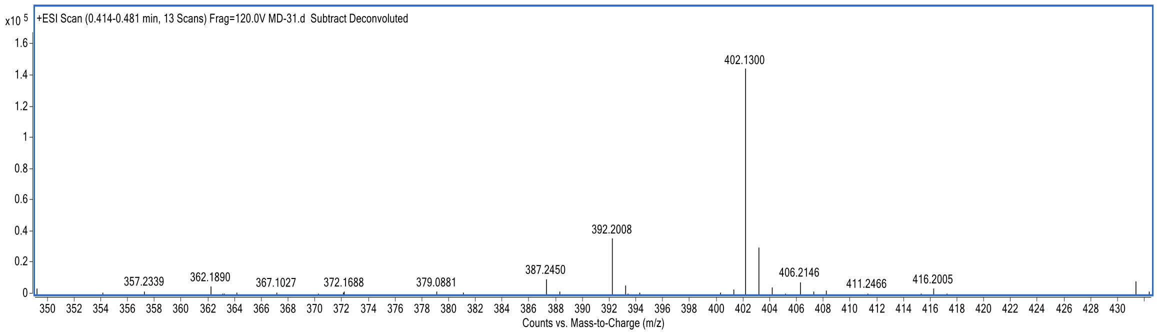

Figure S120. HRMS Spectrum of compound **7t**

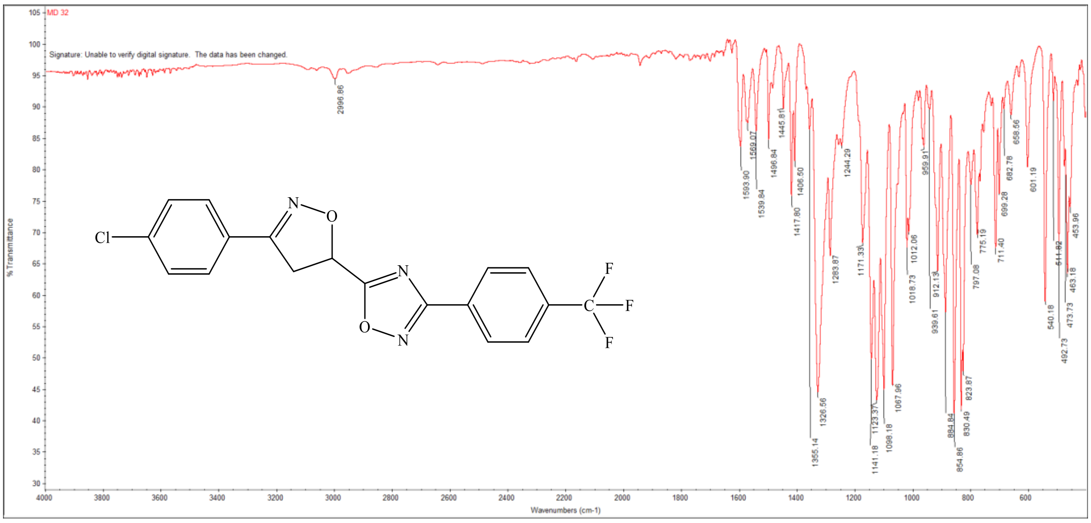

Figure S121. IR Spectrum of compound **7u**

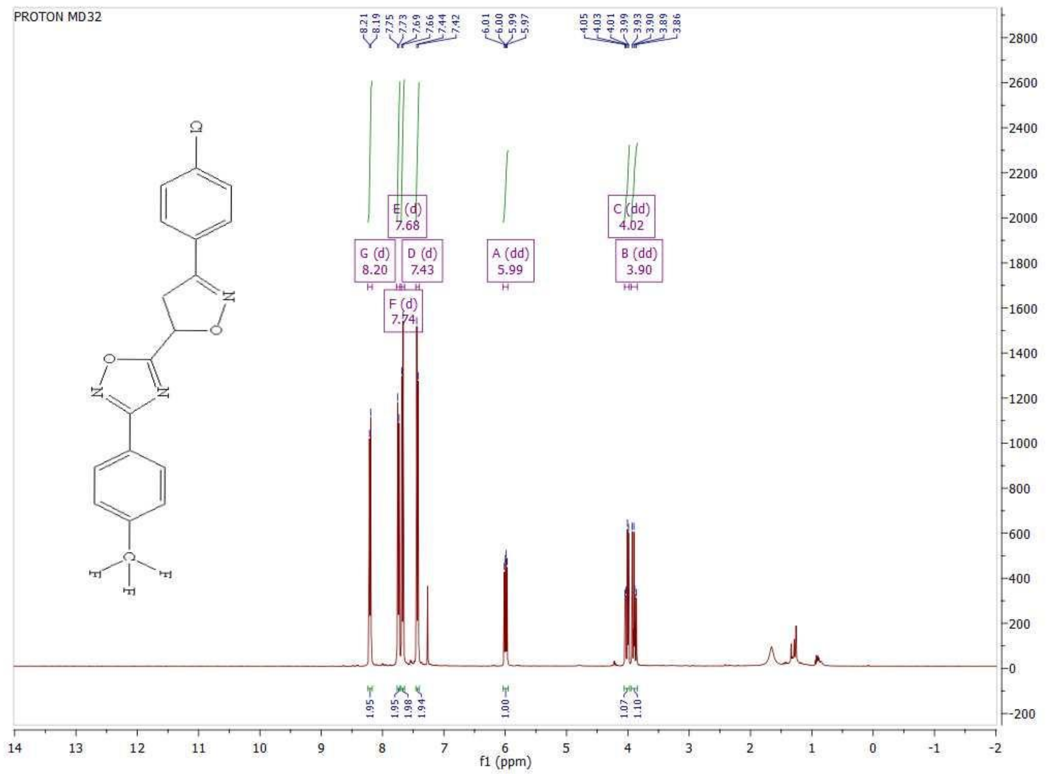

Figure S122. ^{1}H-NMR Spectrum of compound **7u**

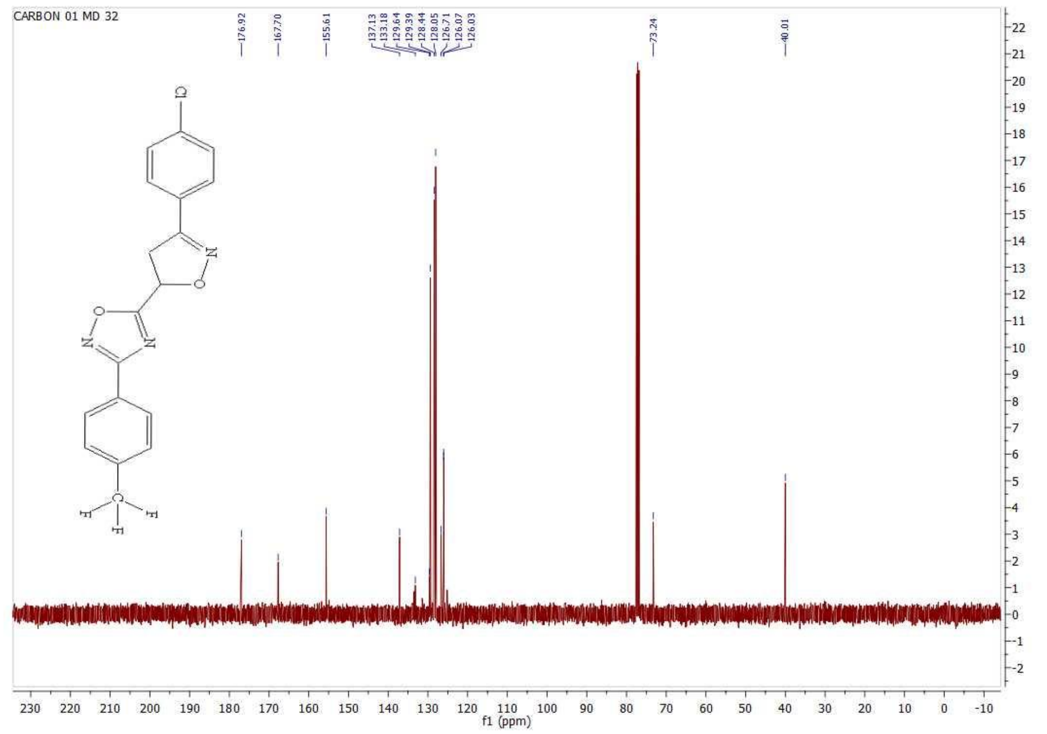

Figure S123. ^{13}C-NMR Spectrum of compound **7u**

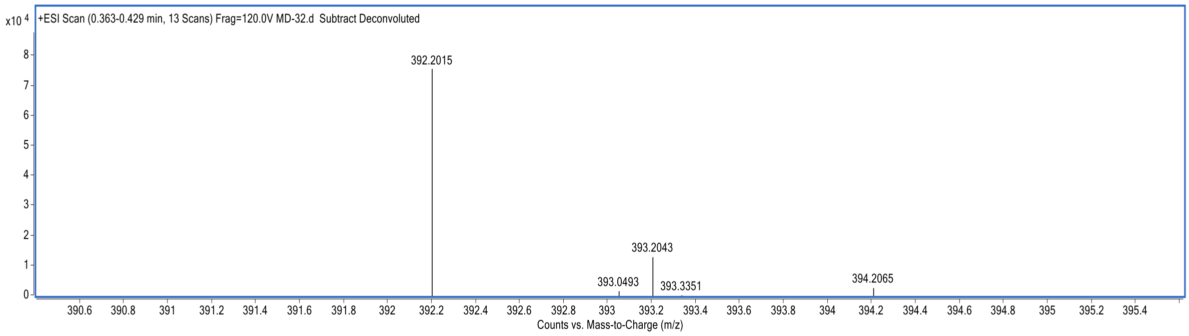

Figure S124. HRMS Spectrum of compound **7u**

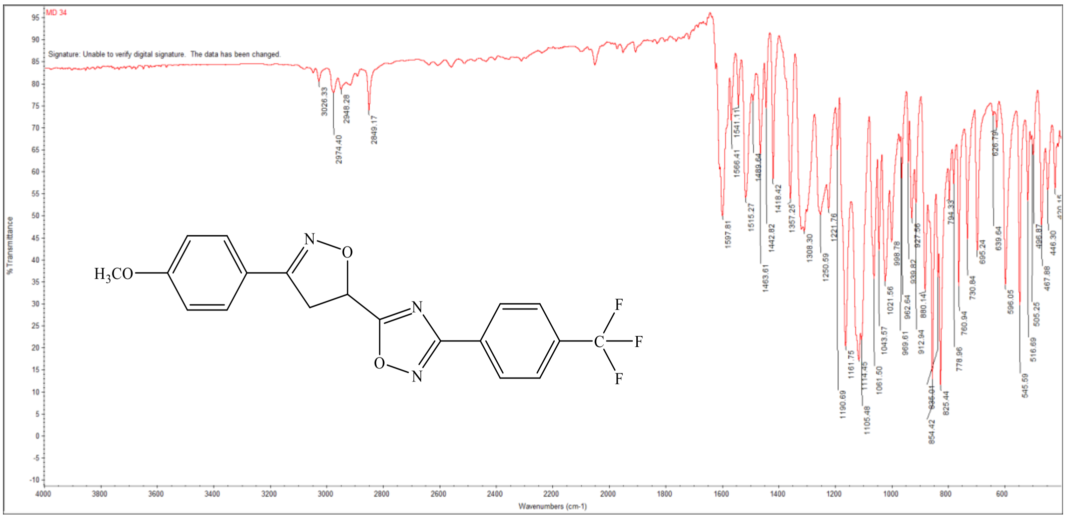

Figure S125. IR Spectrum of compound **7v**

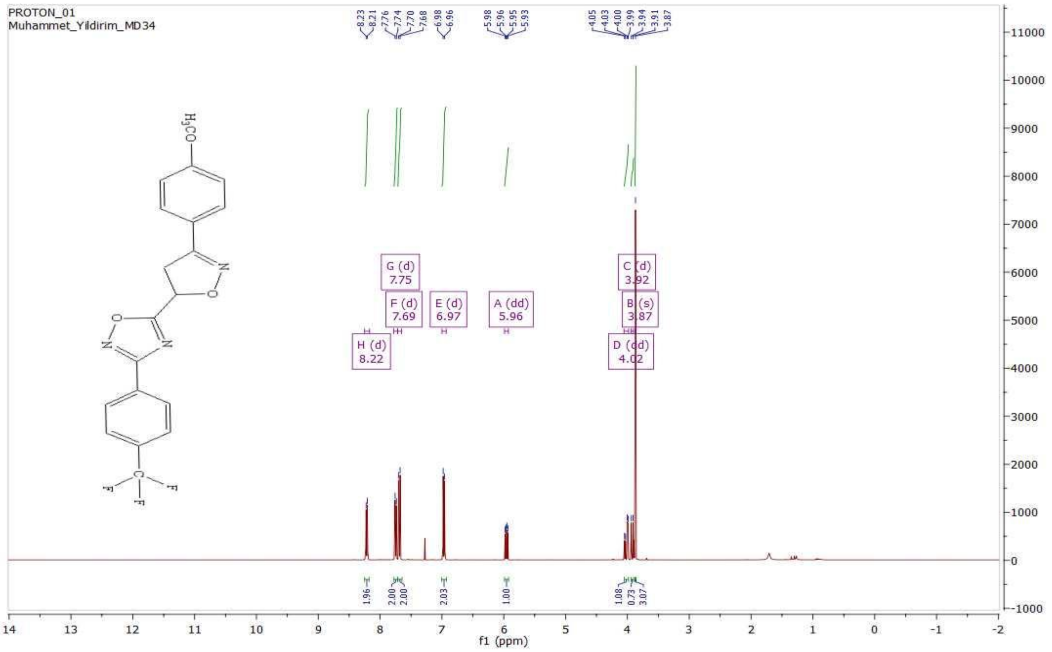

Figure S126. ^{1}H-NMR Spectrum of compound **7v**

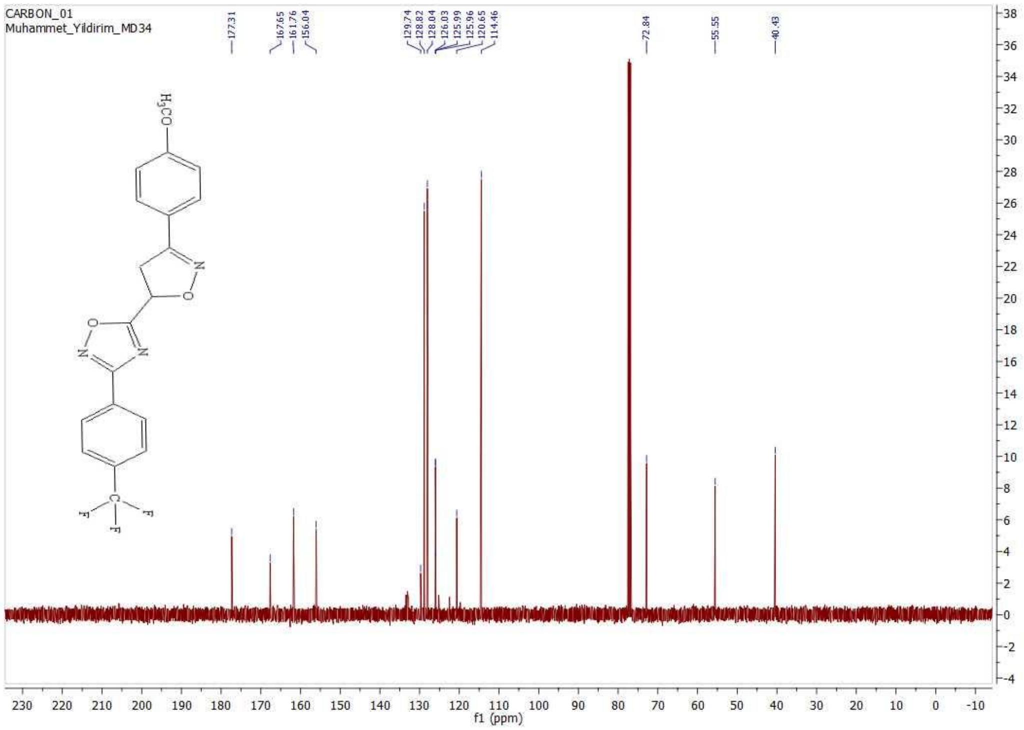

Figure S127. ^{13}C-NMR Spectrum of compound **7v**

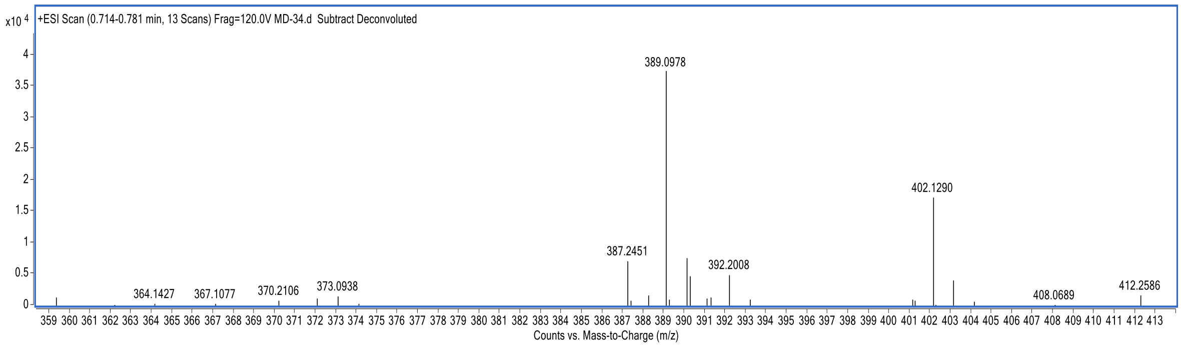

Figure S128. HRMS Spectrum of compound **7v**

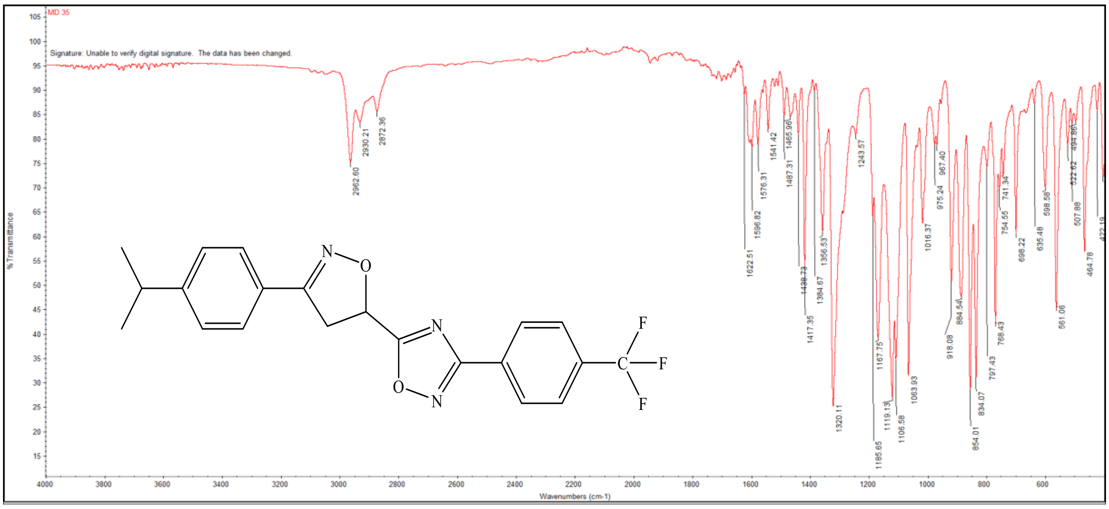

Figure S129. IR Spectrum of compound **7w**

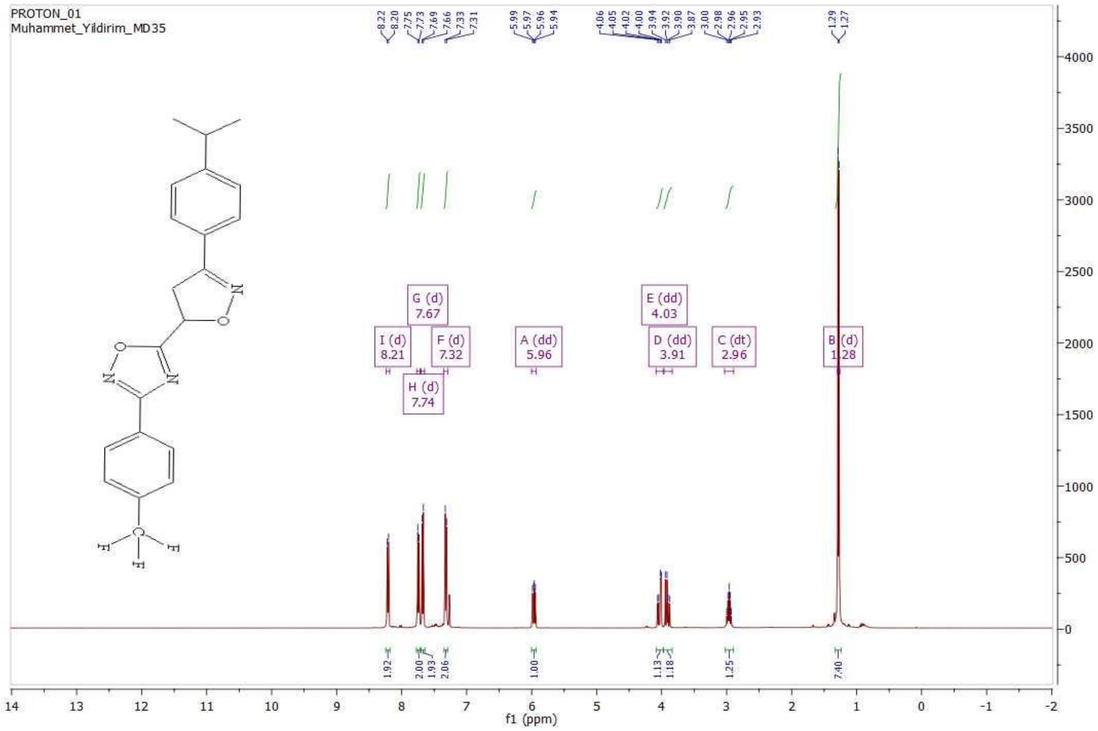

Figure S130. ^{1}H-NMR Spectrum of compound **7w**

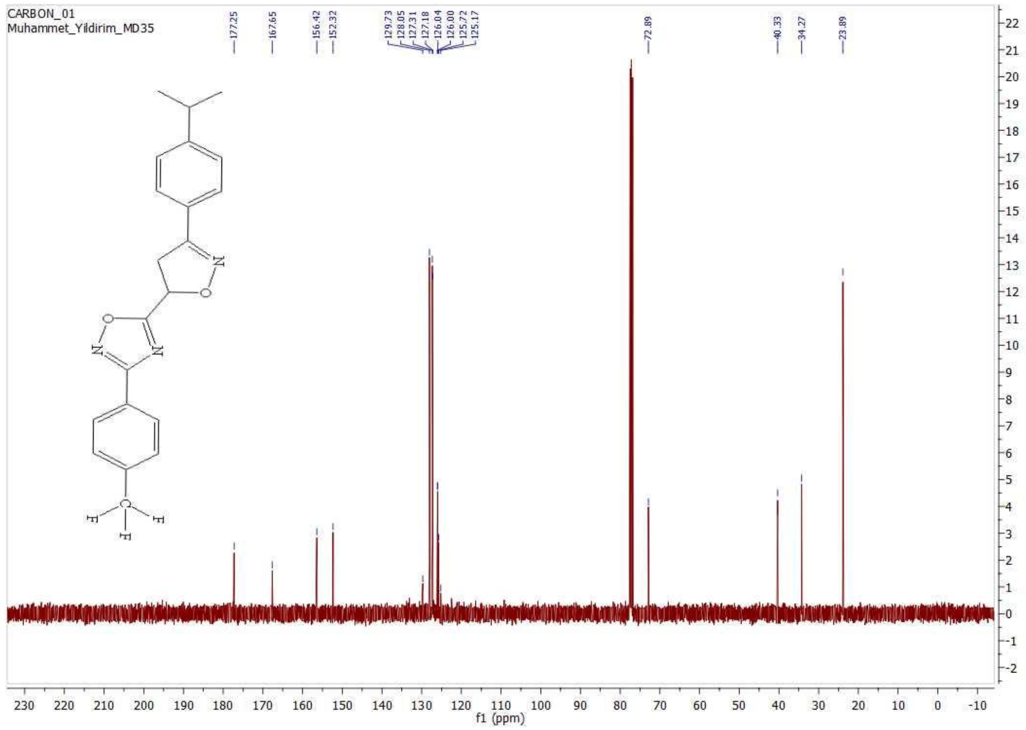

Figure S131. ^{13}C-NMR Spectrum of compound **7w**

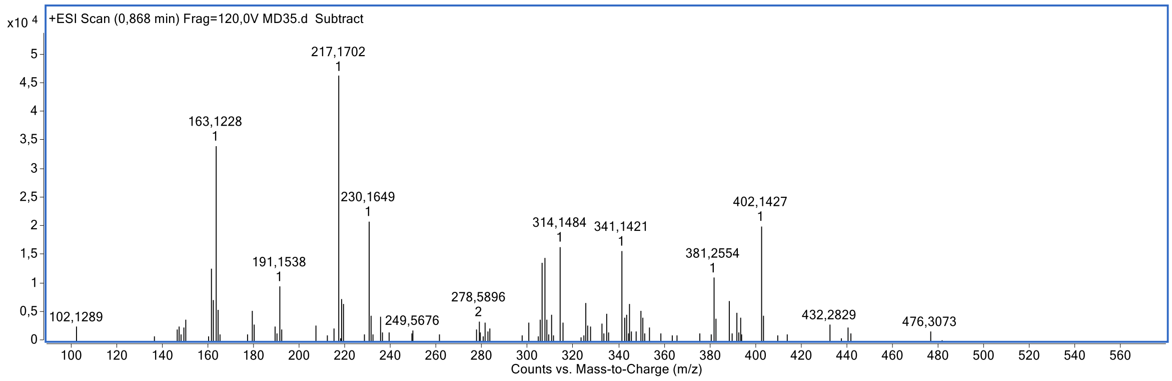

Figure S132. HRMS Spectrum of compound **7w**

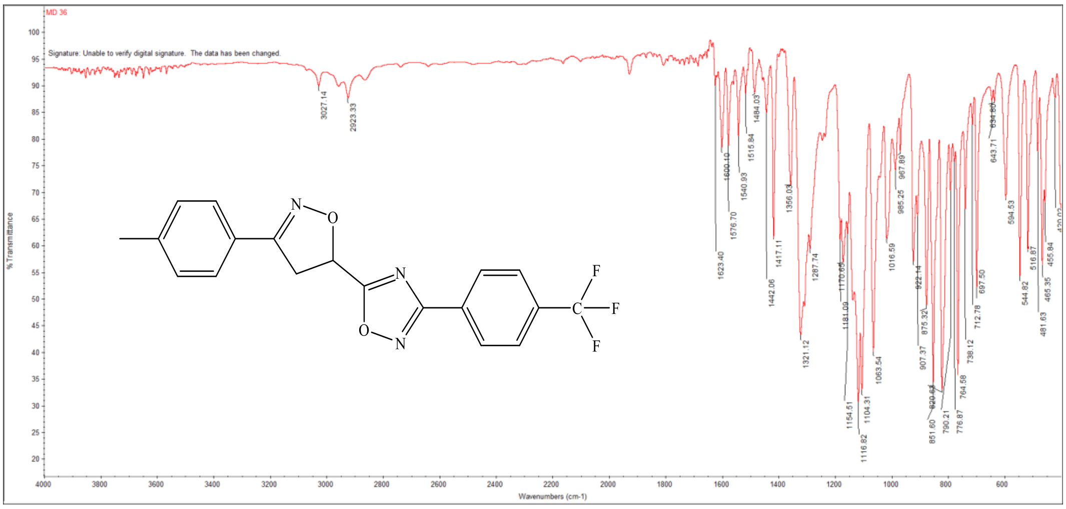

Figure S133. IR Spectrum of compound **7x**

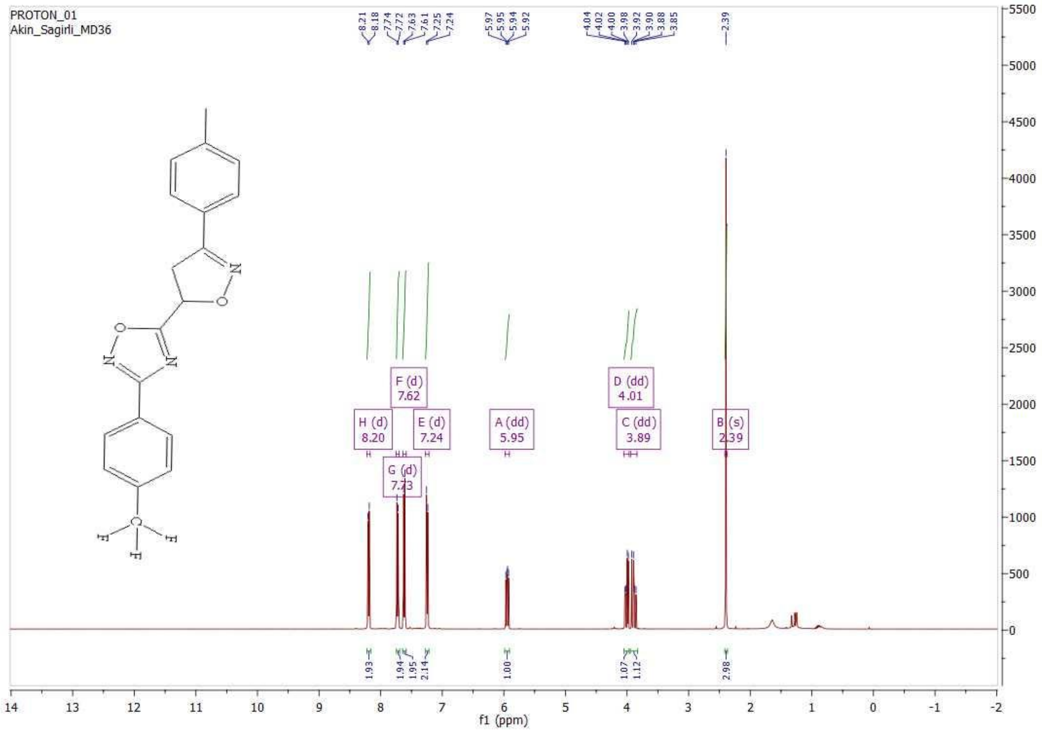

Figure S134. ^{1}H-NMR Spectrum of compound **7x**

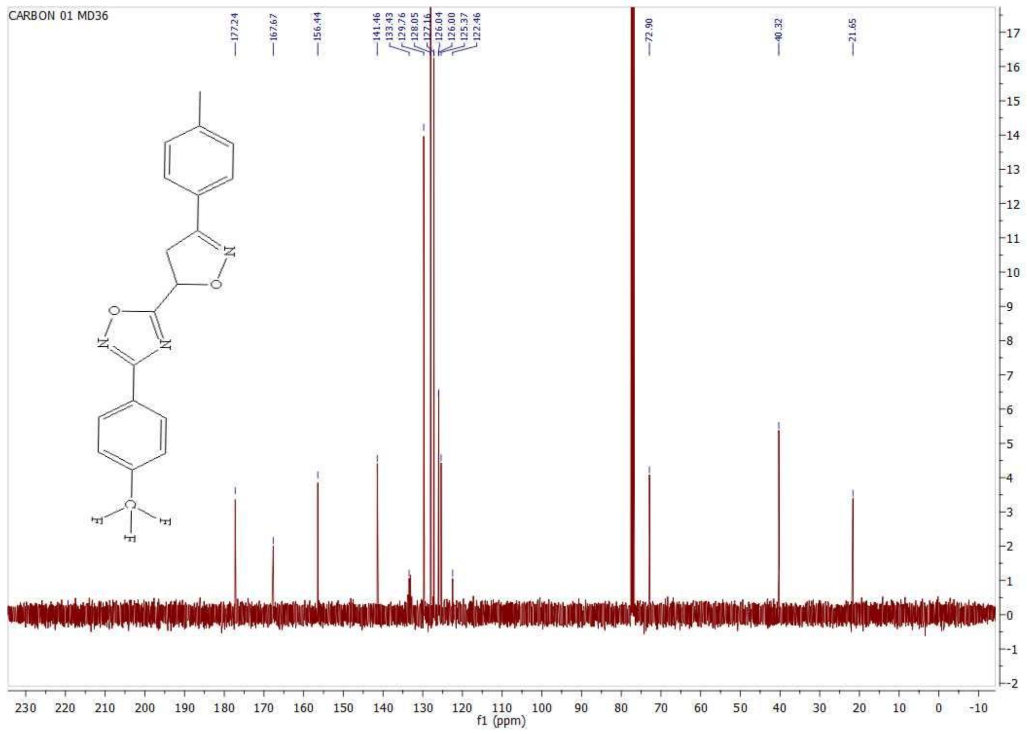

Figure S135. ^{13}C-NMR Spectrum of compound **7x**

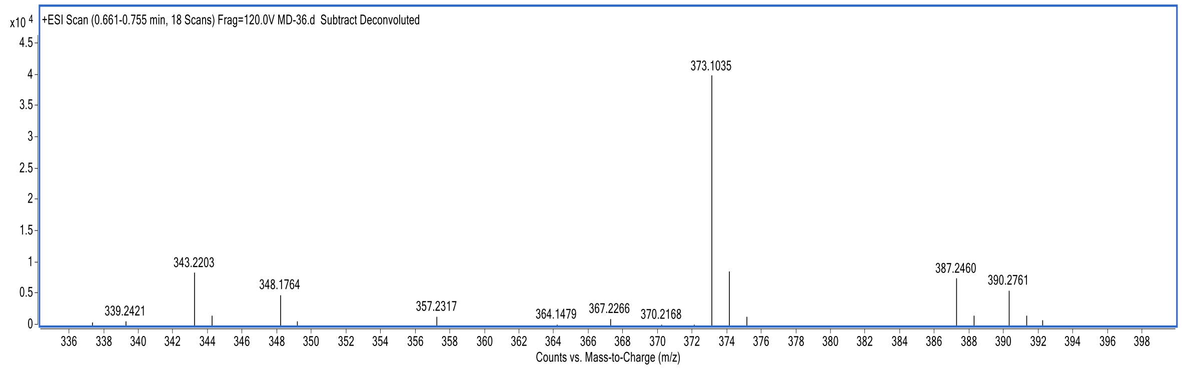

Figure S136. HRMS Spectrum of compound **7x**

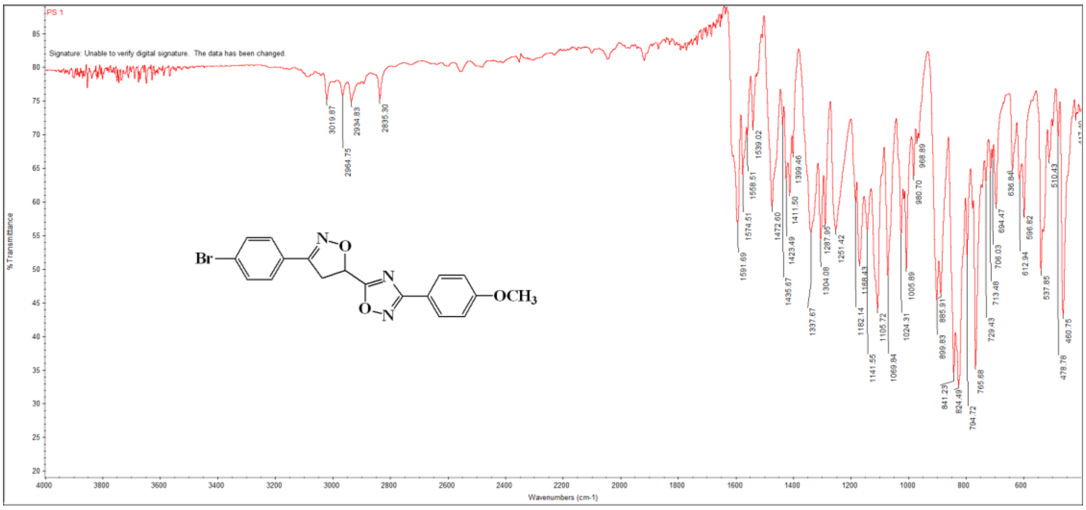

Figure S137. IR Spectrum of compound **7aa**

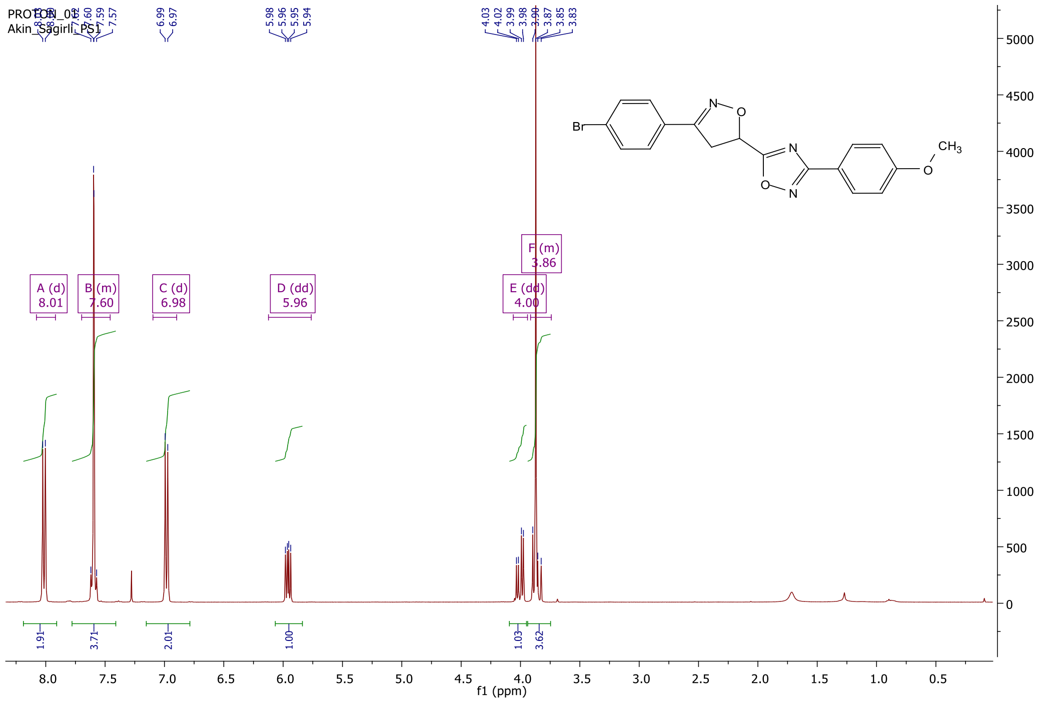

Figure S138. ^{1}H-NMR Spectrum of compound **7aa**

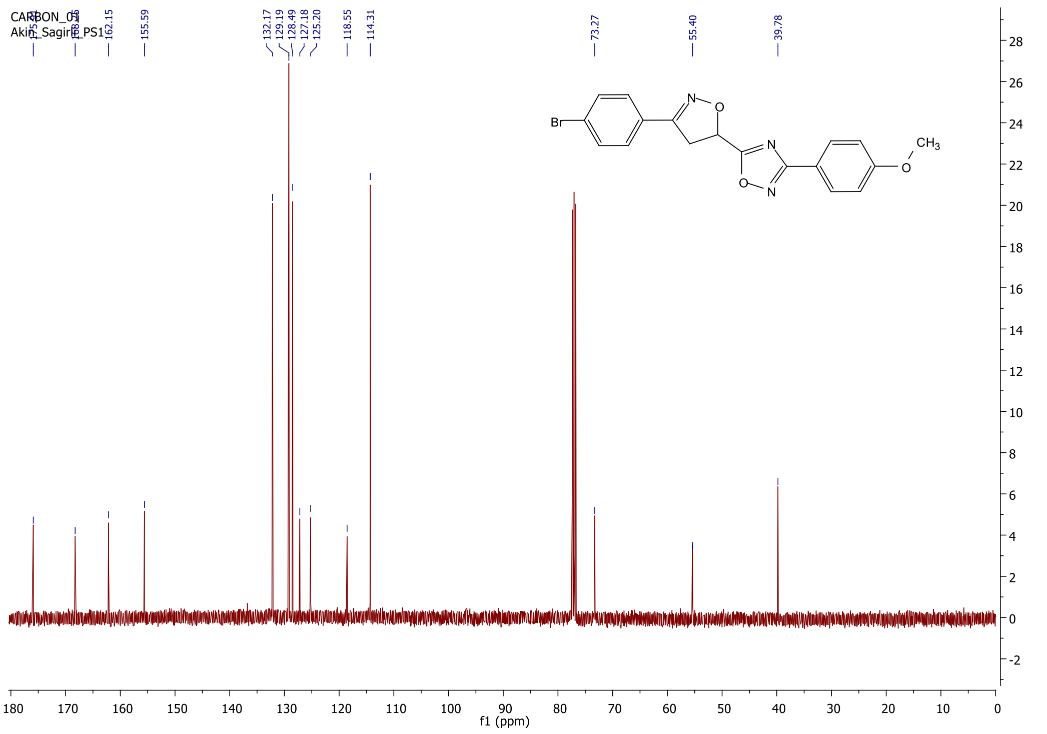

Figure S139. ^{13}C-NMR Spectrum of compound **7aa**

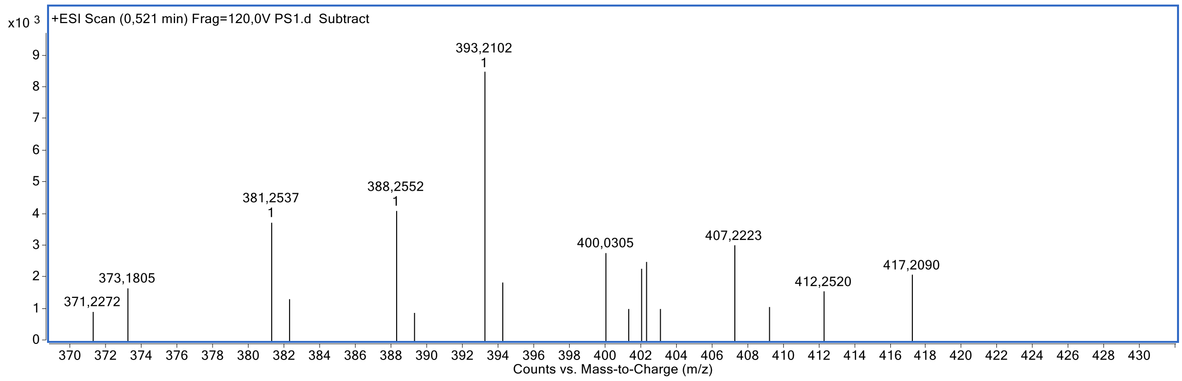

Figure S140. HRMS Spectrum of compound **7aa**

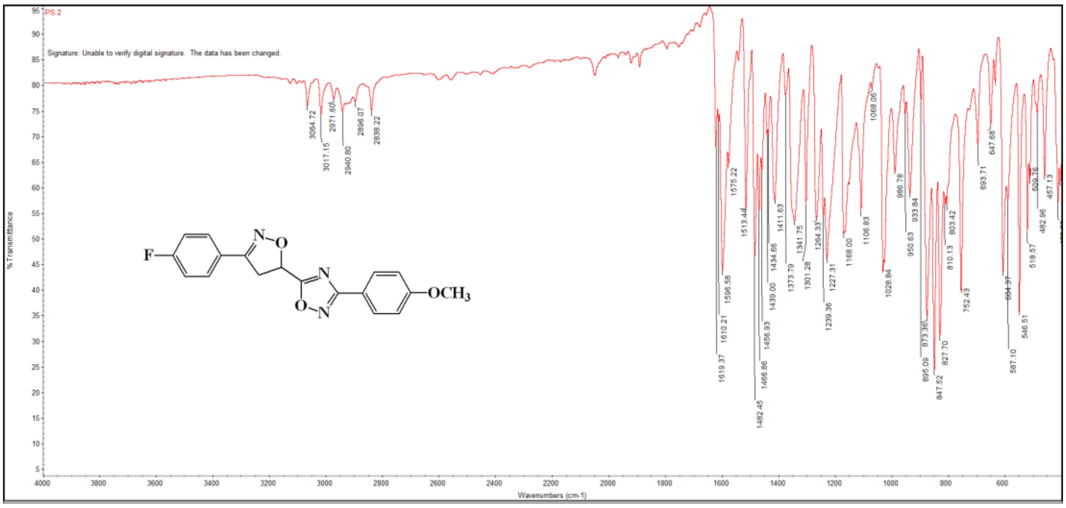

Figure S141. IR Spectrum of compound **7ab**

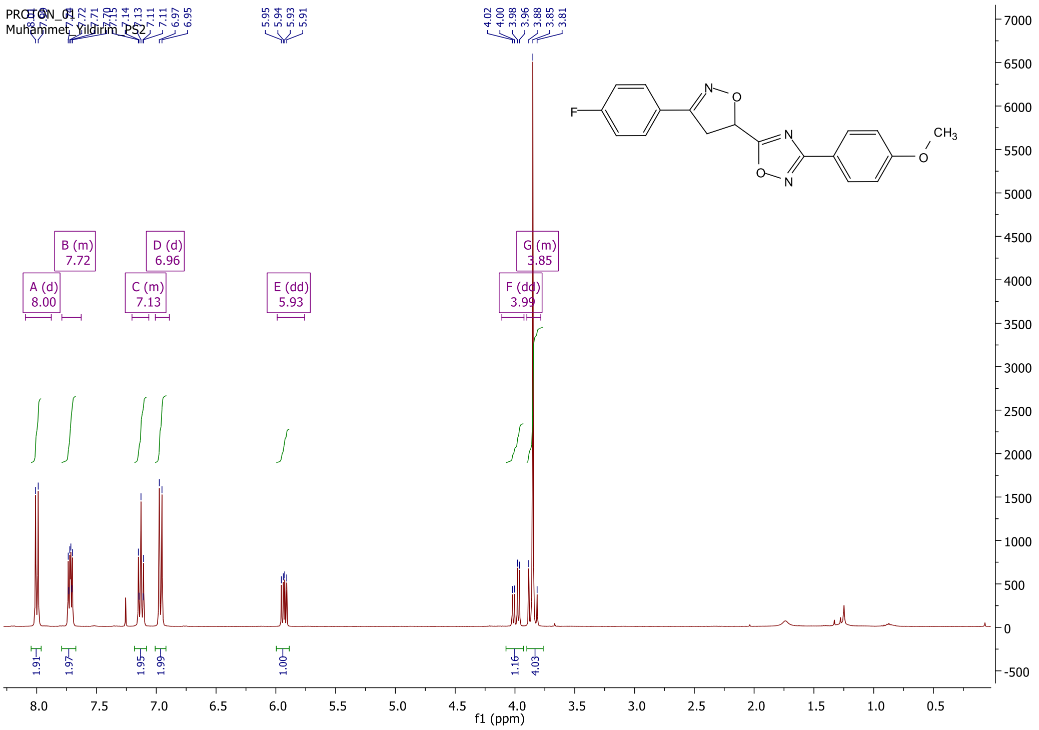

Figure S142. ^{1}H-NMR Spectrum of compound **7ab**

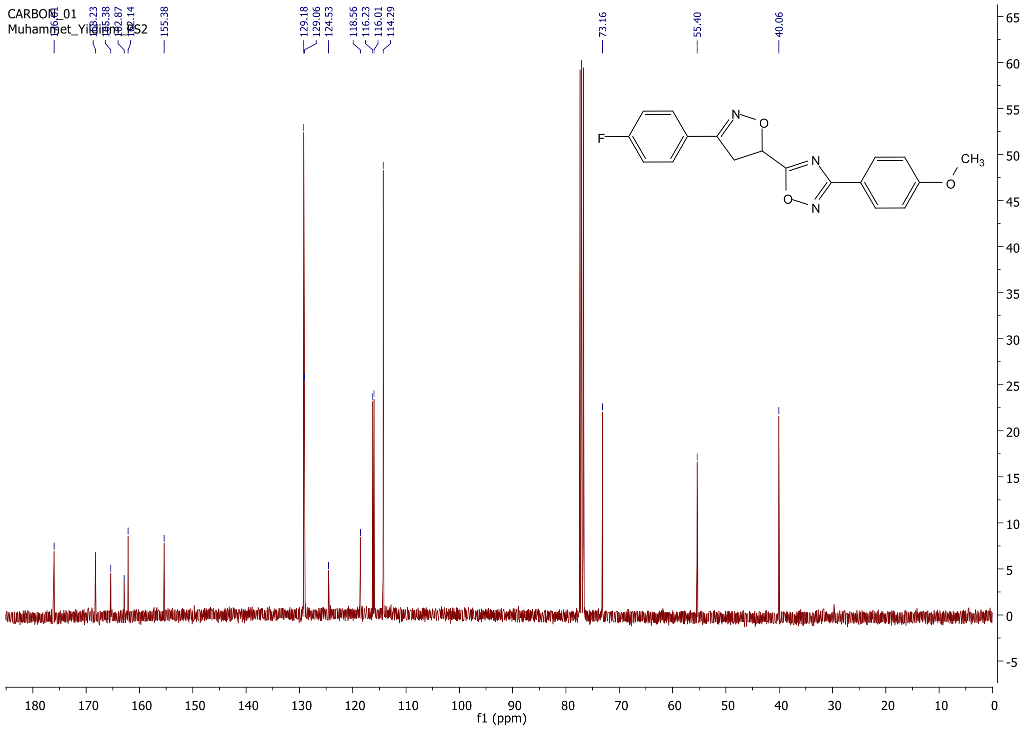

Figure S143. ^{13}C-NMR Spectrum of compound **7ab**

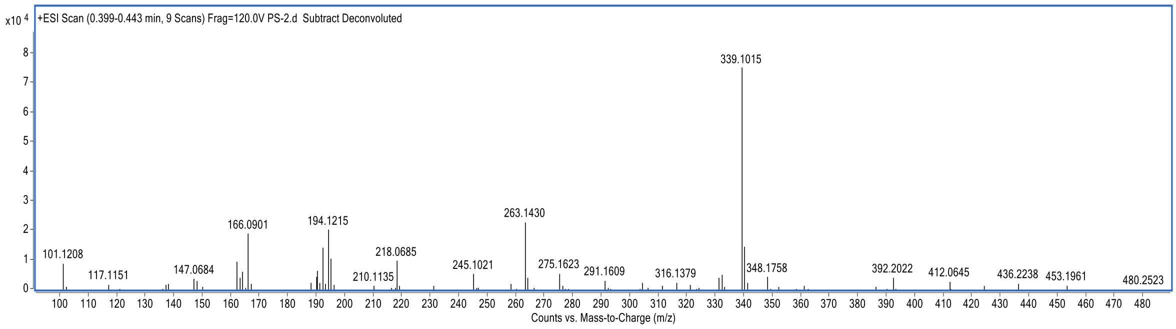

Figure S144. HRMS Spectrum of compound **7ab**

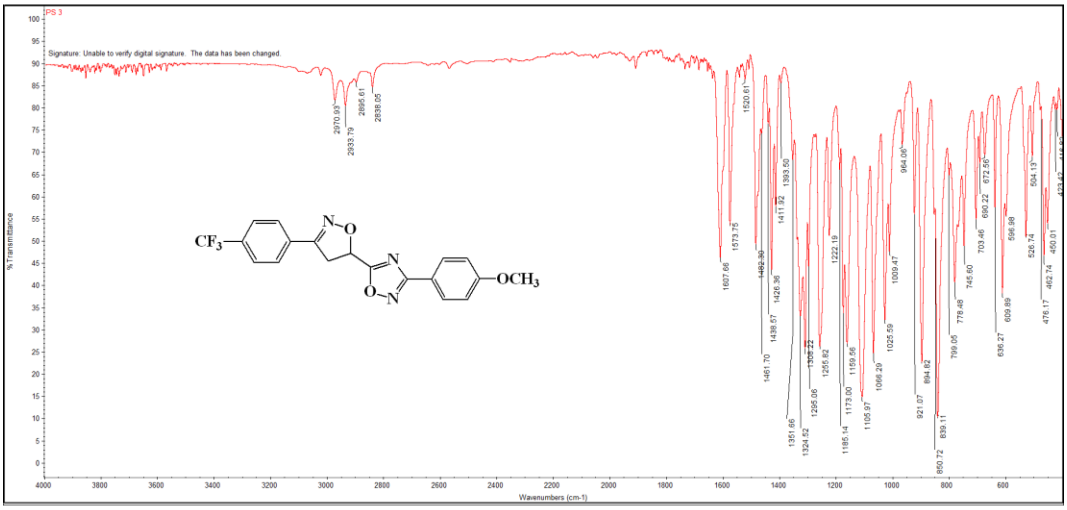

Figure S145. IR Spectrum of compound **7ac**

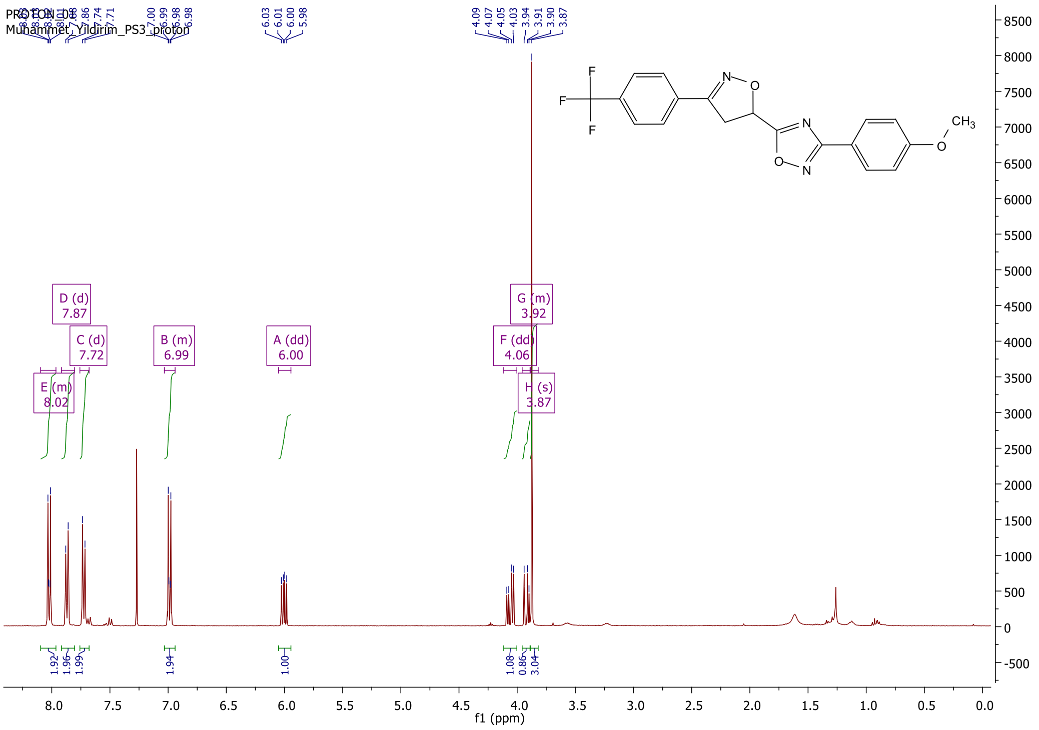

Figure S146. ^{1}H-NMR Spectrum of compound **7ac**

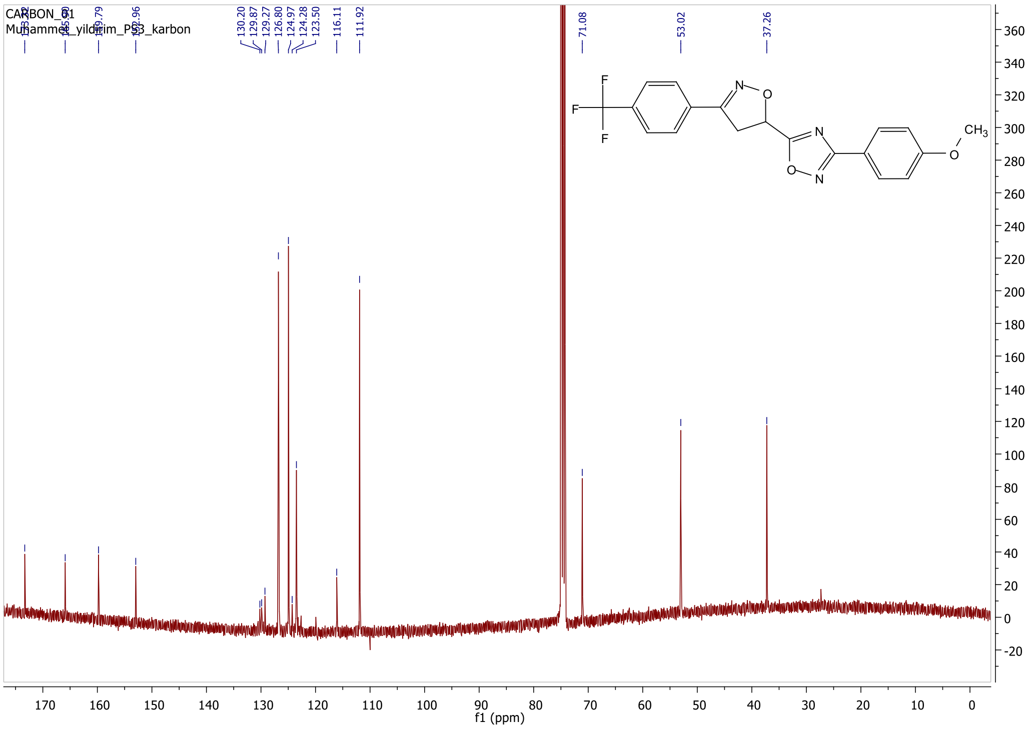

Figure S147. ^{13}C-NMR Spectrum of compound **7ac**

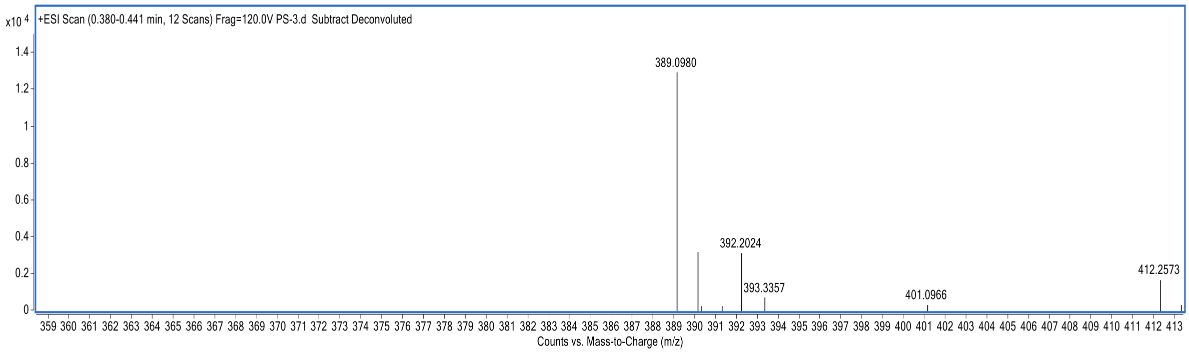

Figure S148. HRMS Spectrum of compound **7ac**

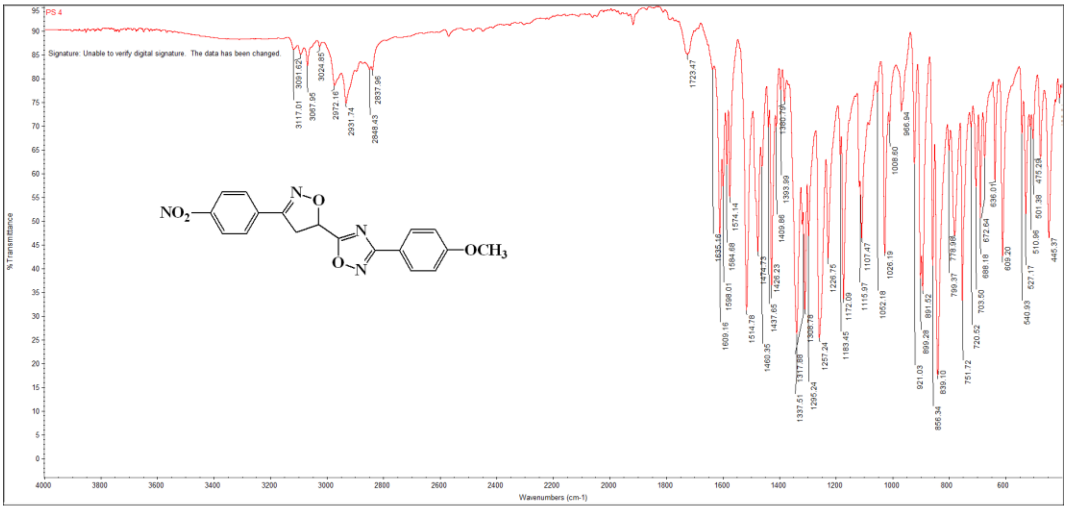

Figure S149. IR Spectrum of compound **7ad**

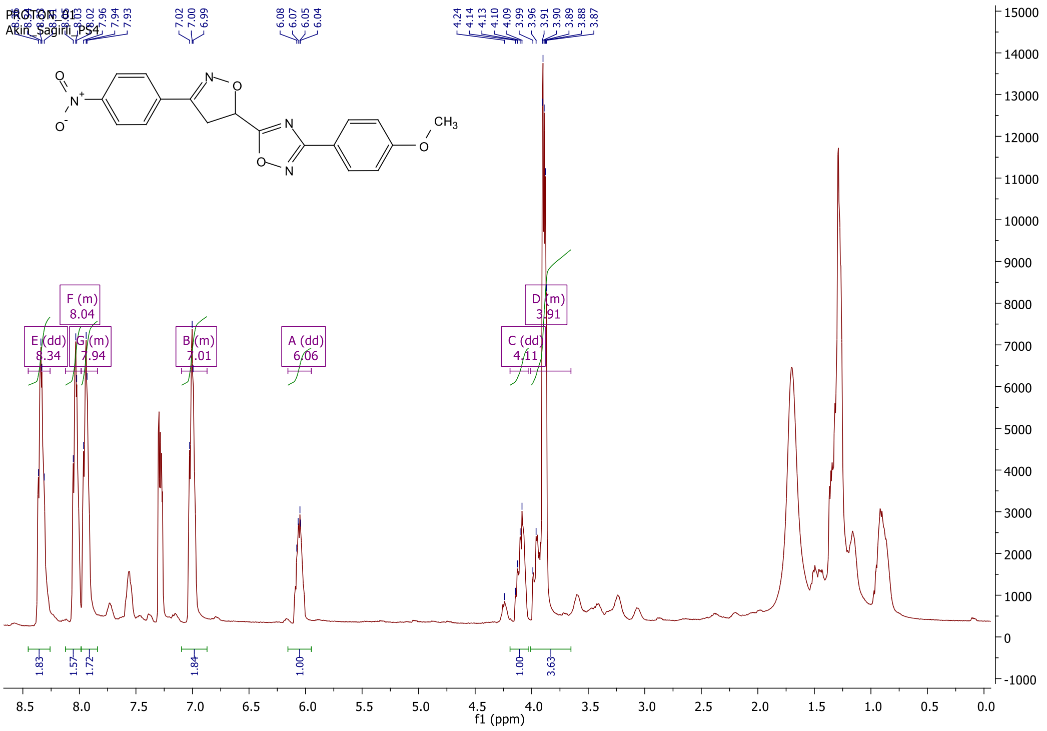

Figure S150. ^{1}H-NMR Spectrum of compound **7ad**

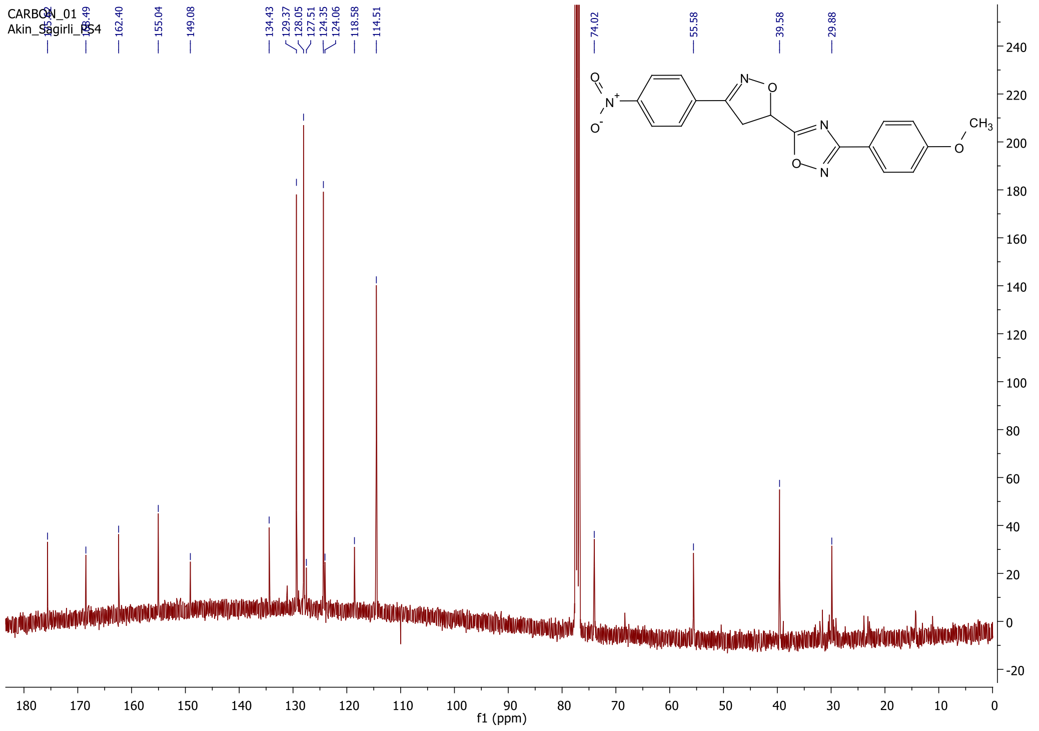

Figure S151. ^{13}C-NMR Spectrum of compound **7ad**

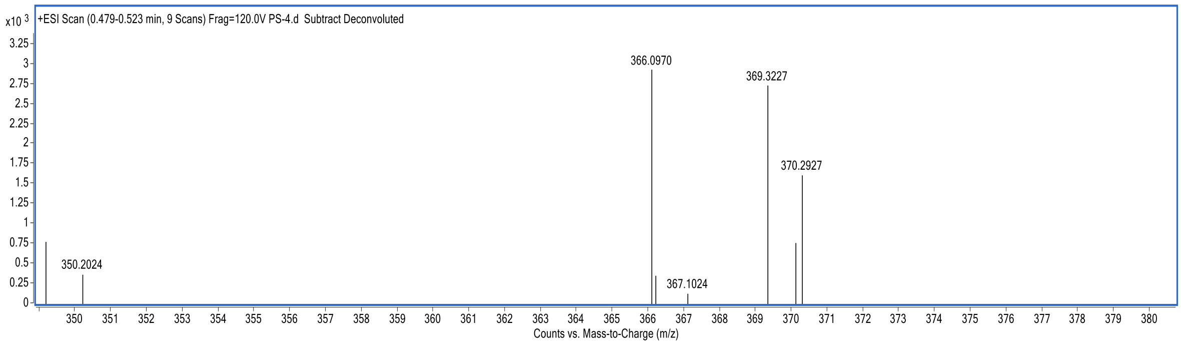

Figure S152. HRMS Spectrum of compound **7ad**

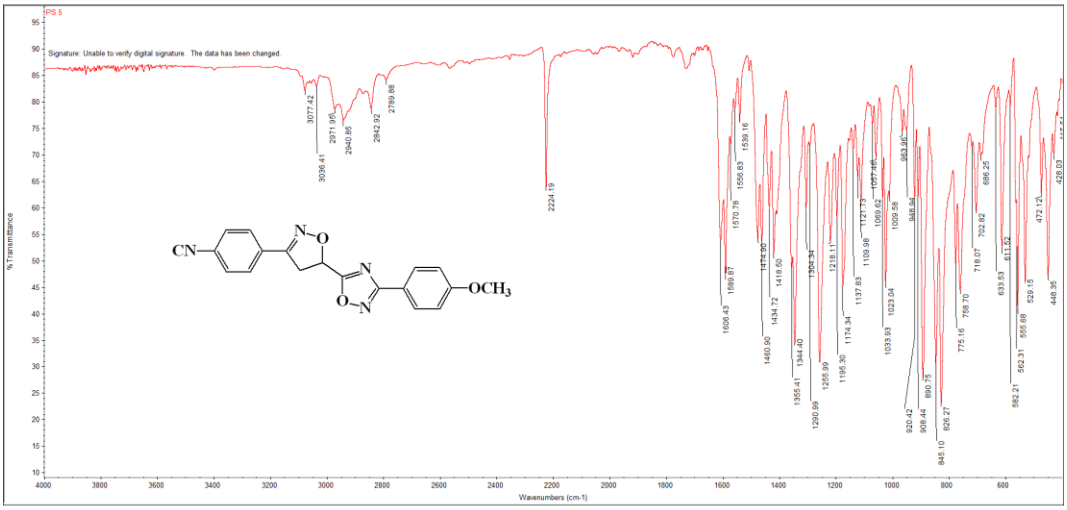

Figure S153. IR Spectrum of compound **7ae**

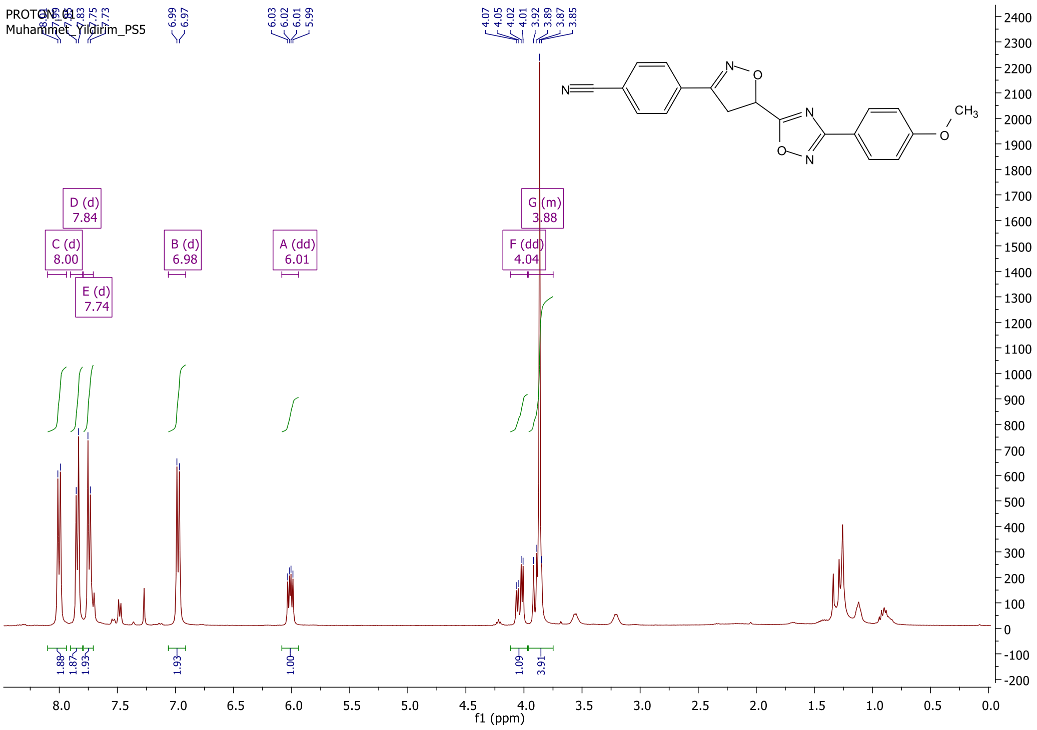

Figure S154. ^{1}H-NMR Spectrum of compound **7ae**

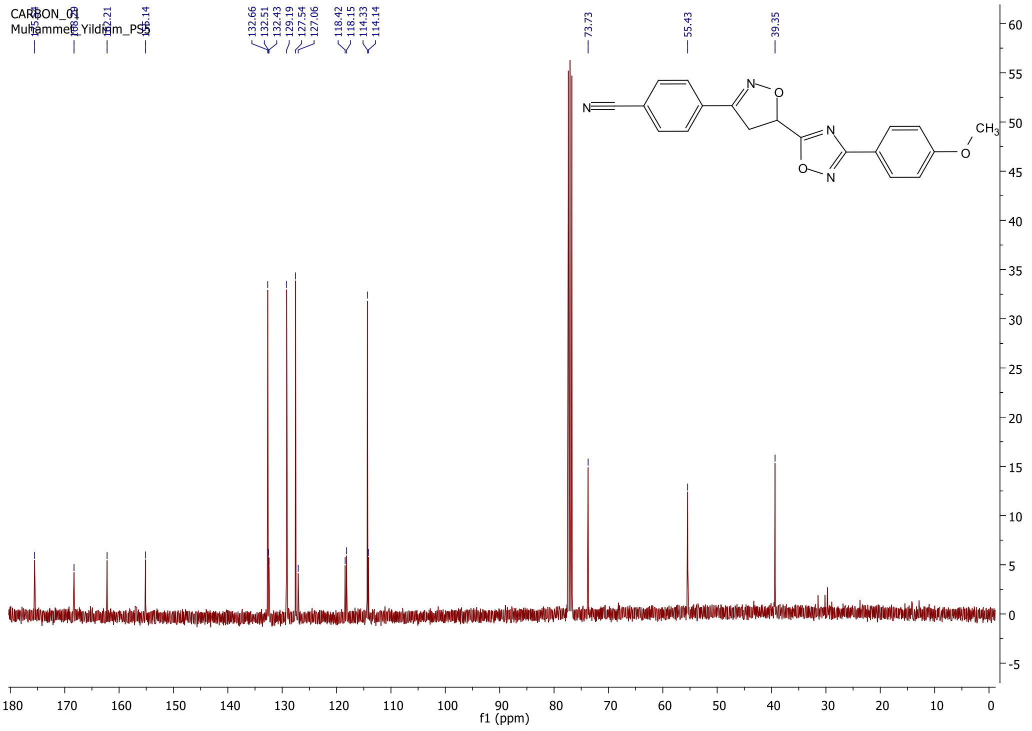

Figure S155. ^{13}C-NMR Spectrum of compound **7ae**

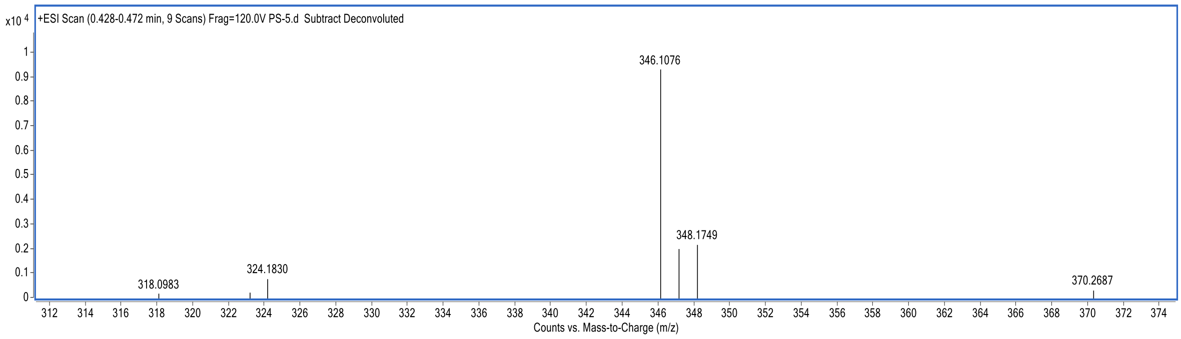

Figure S156. HRMS Spectrum of compound **7ae**

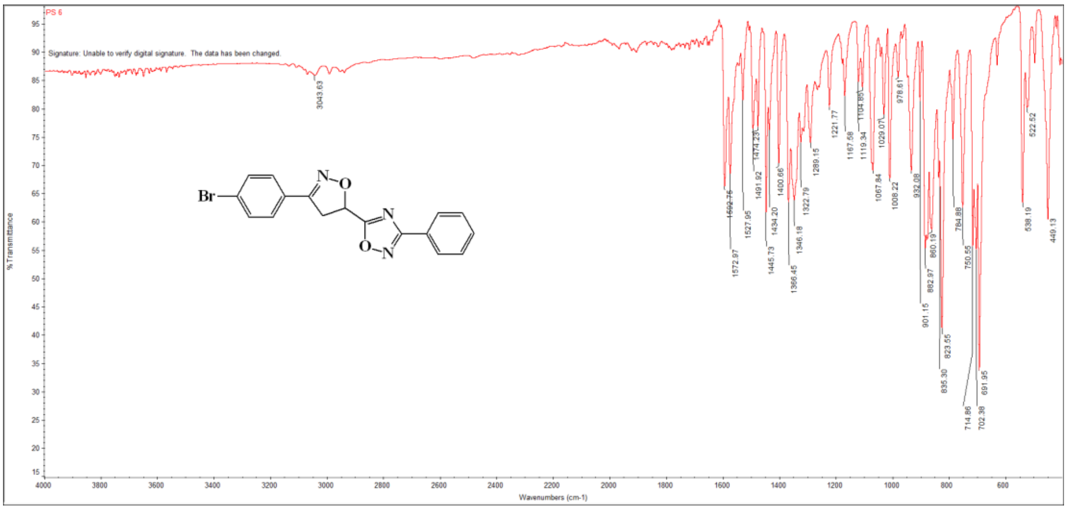

Figure S157. IR Spectrum of compound **7af**

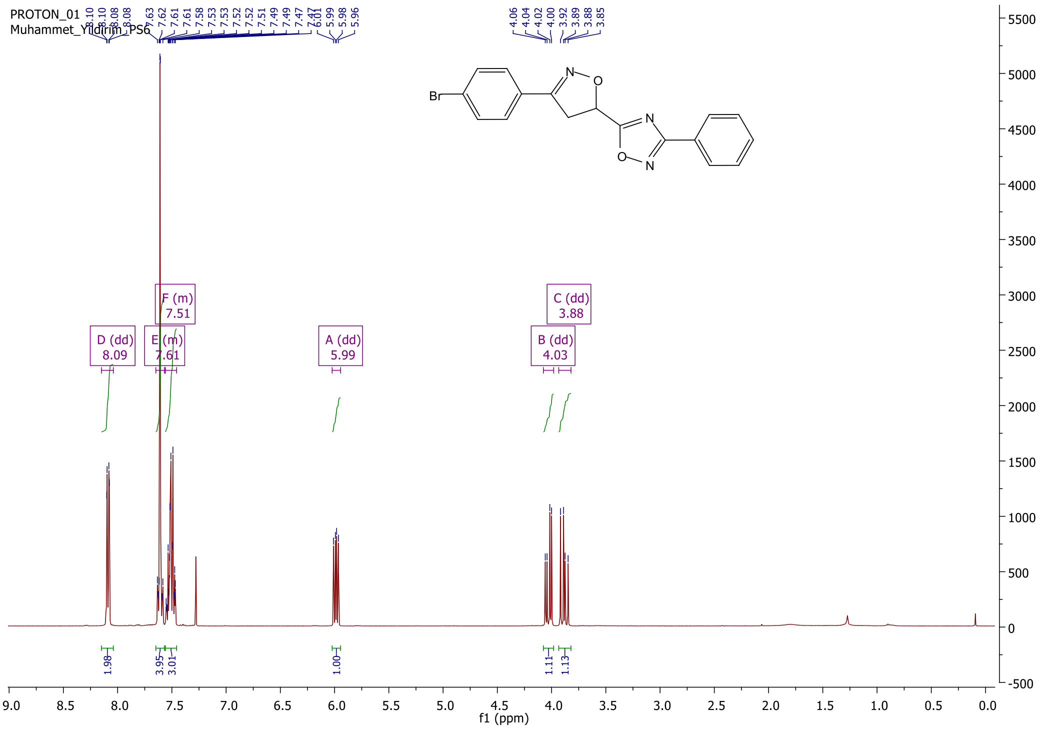

Figure S158. ^{1}H-NMR Spectrum of compound **7af**

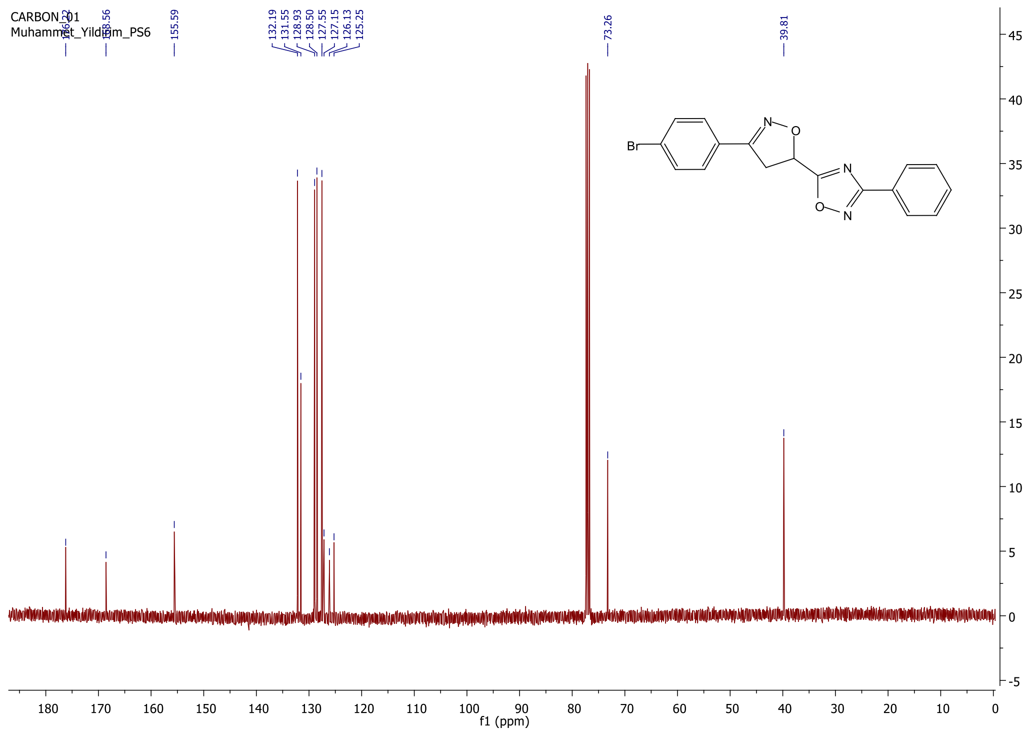

Figure S159. ^{13}C-NMR Spectrum of compound **7af**

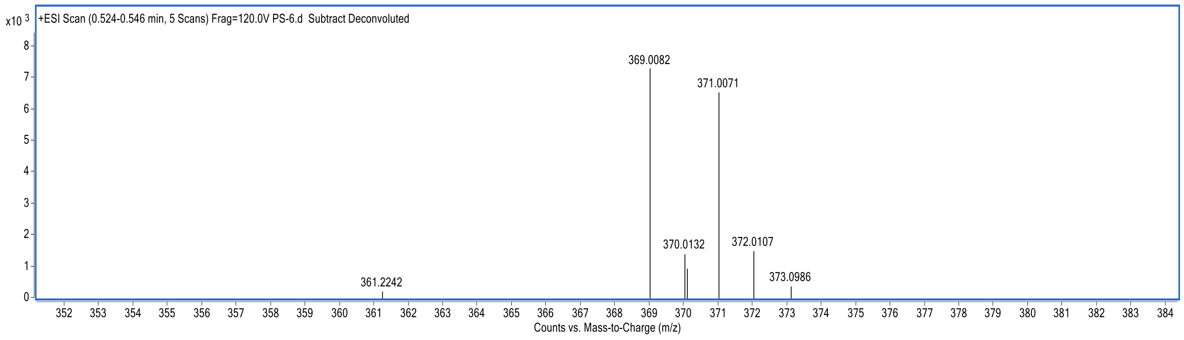

Figure S160. HRMS Spectrum of compound **7af**

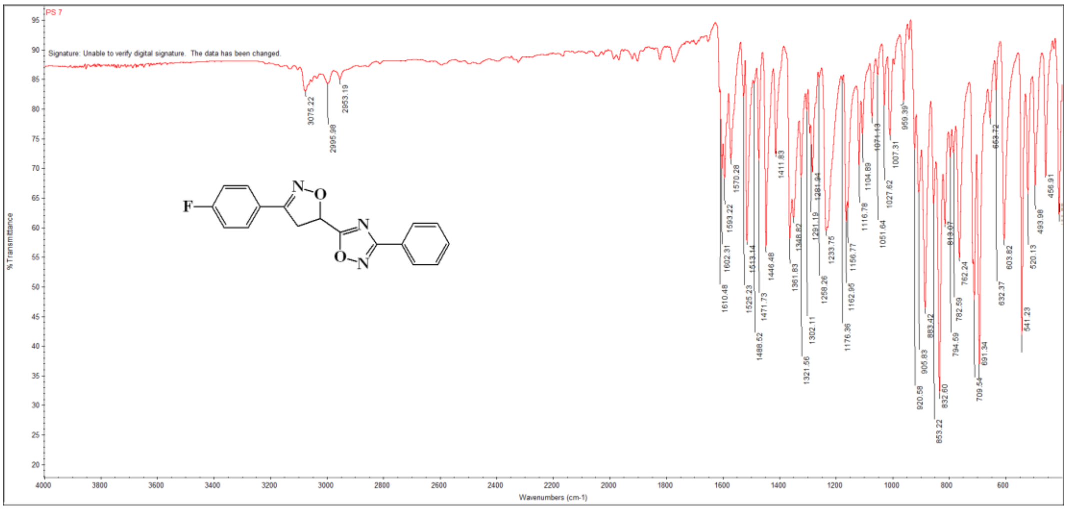

Figure S161. IR Spectrum of compound **7ag**

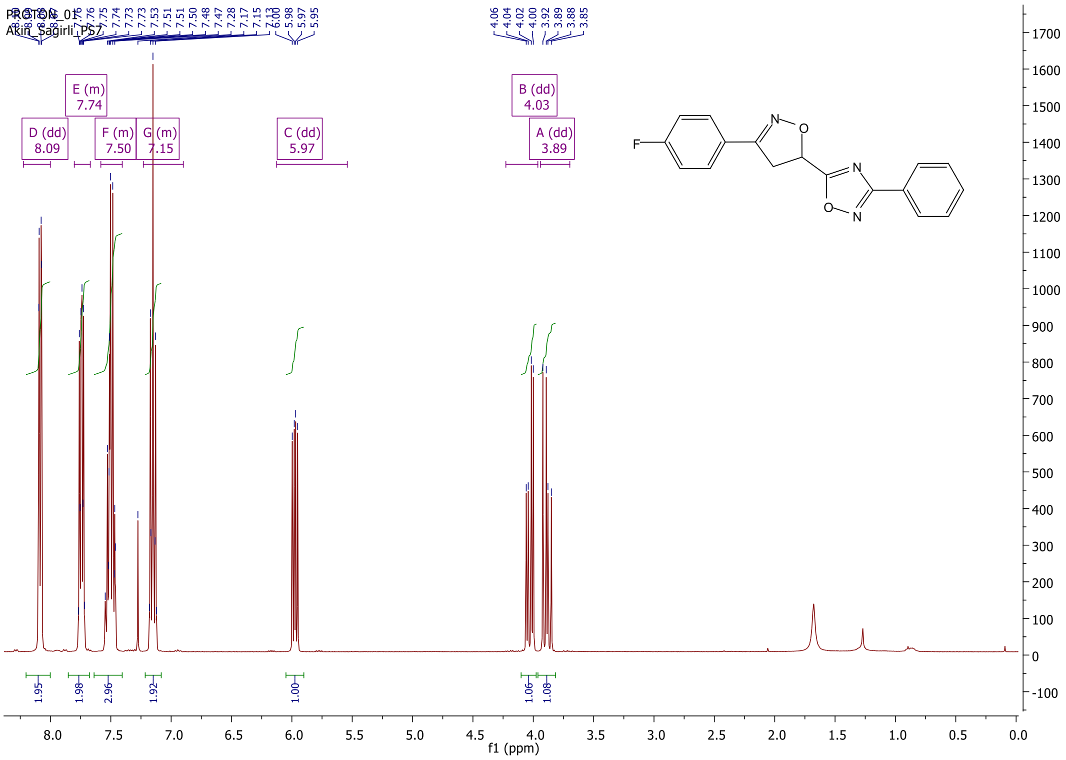

Figure S162. ^{1}H-NMR Spectrum of compound **7ag**

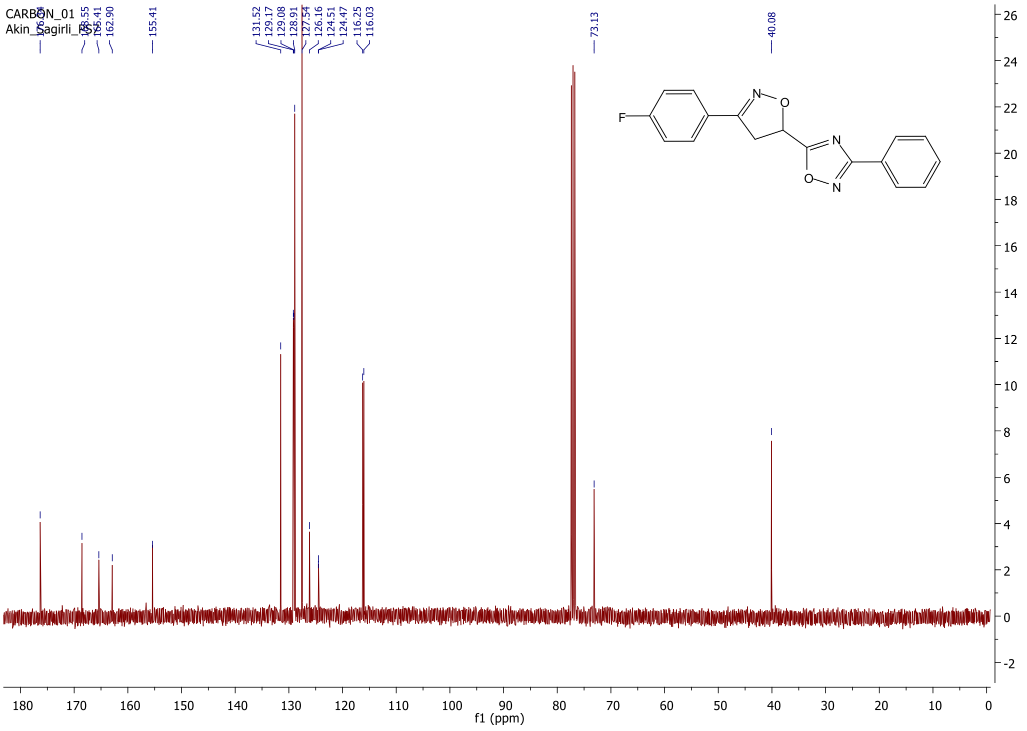

Figure S163. ^{13}C-NMR Spectrum of compound **7ag**

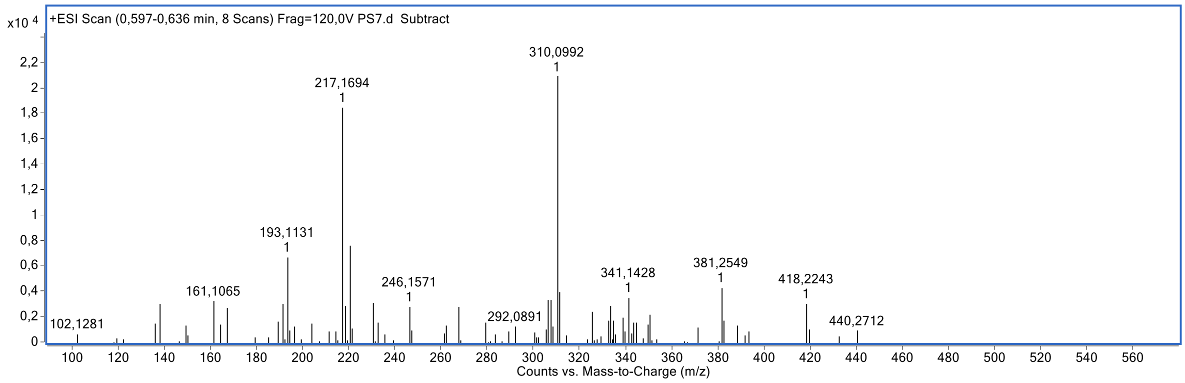

Figure S164. HRMS Spectrum of compound **7ag**

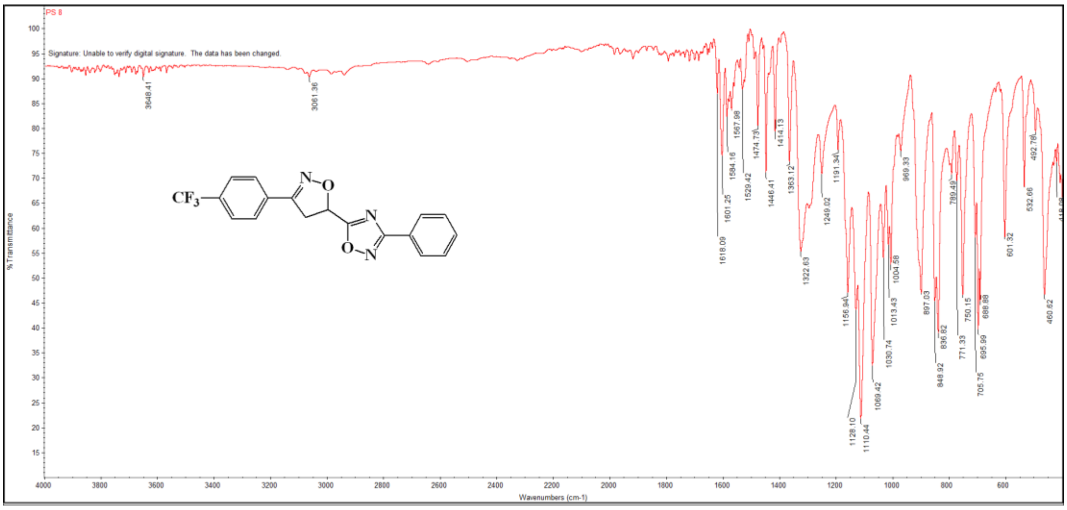

Figure S165. IR Spectrum of compound **7ah**

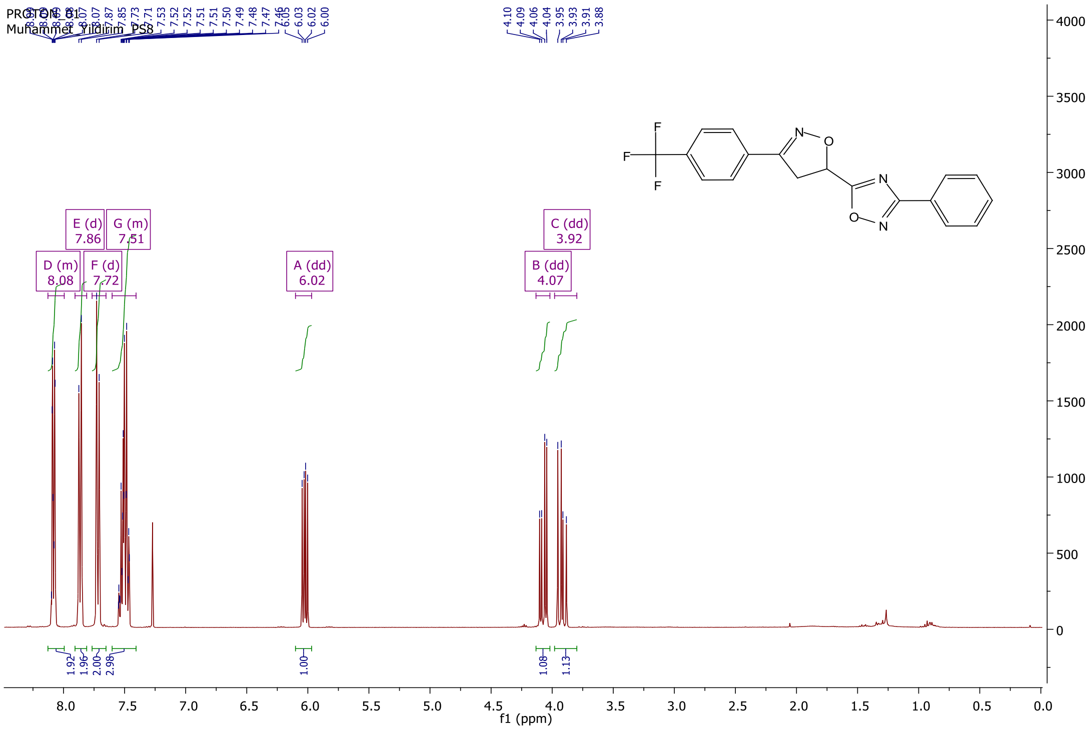

Figure S166. ^{1}H-NMR Spectrum of compound **7ah**

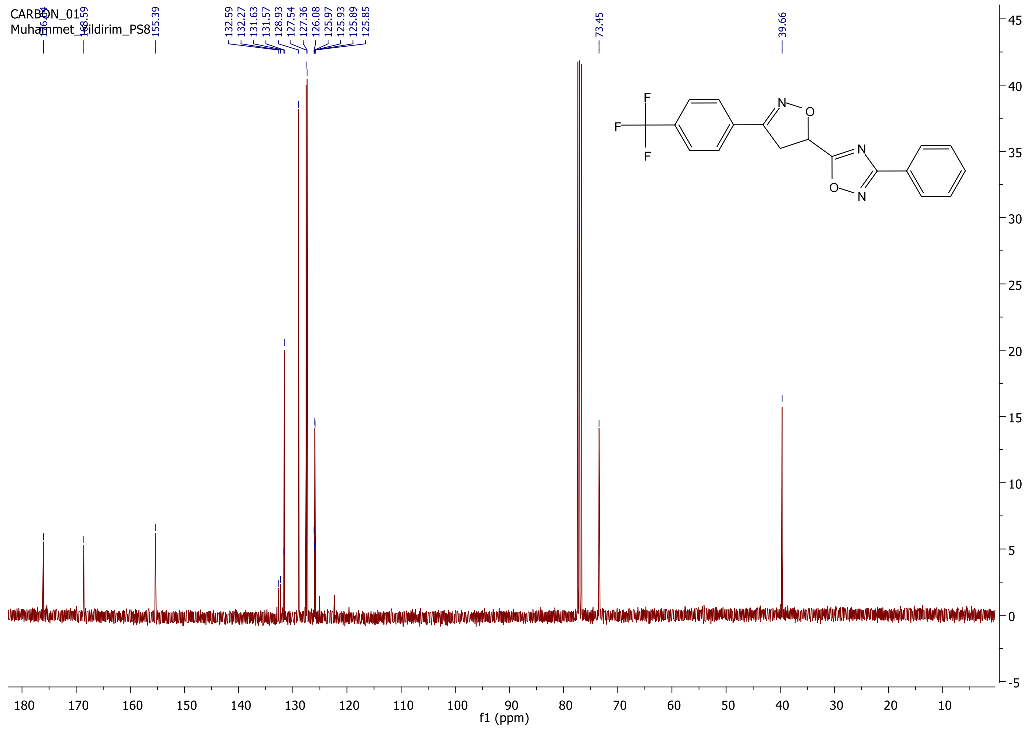

Figure S167. ^{13}C-NMR Spectrum of compound **7ah**

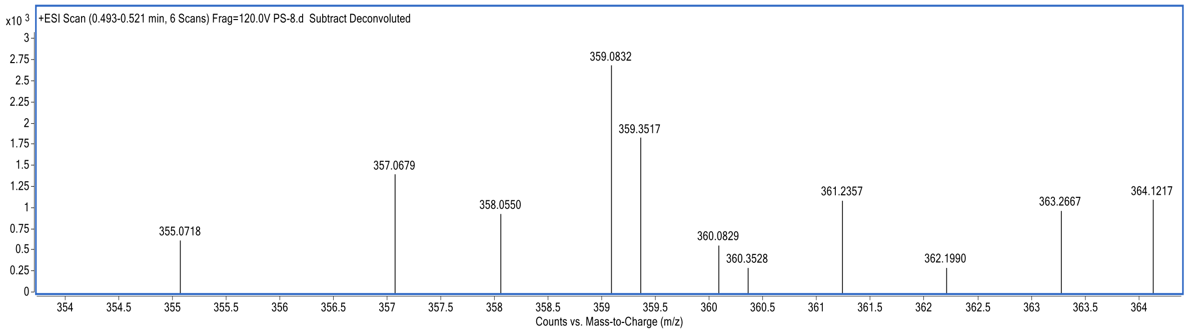

Figure S168. HRMS Spectrum of compound **7ah**

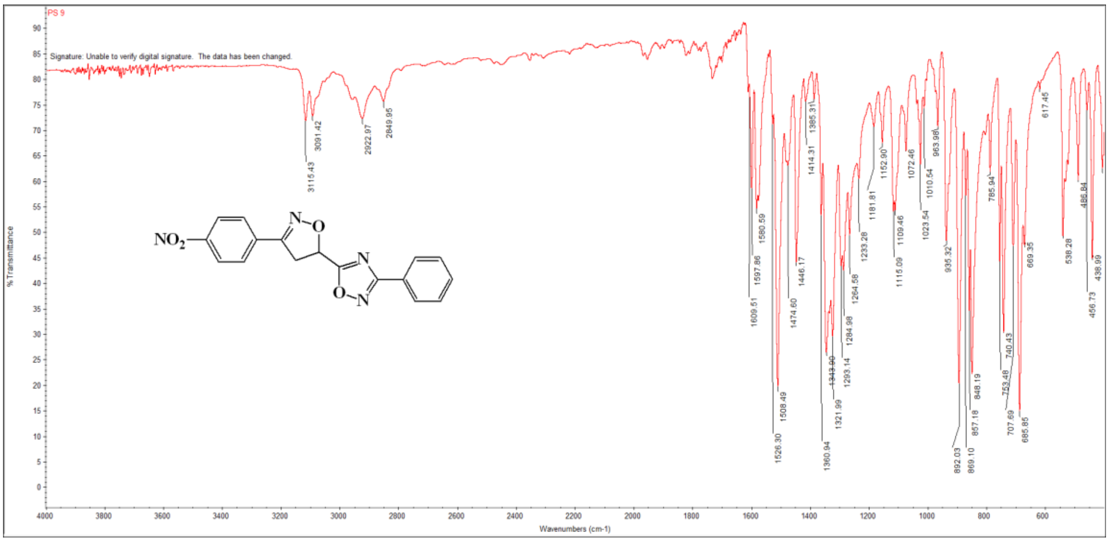

Figure S169. IR Spectrum of compound **7ai**

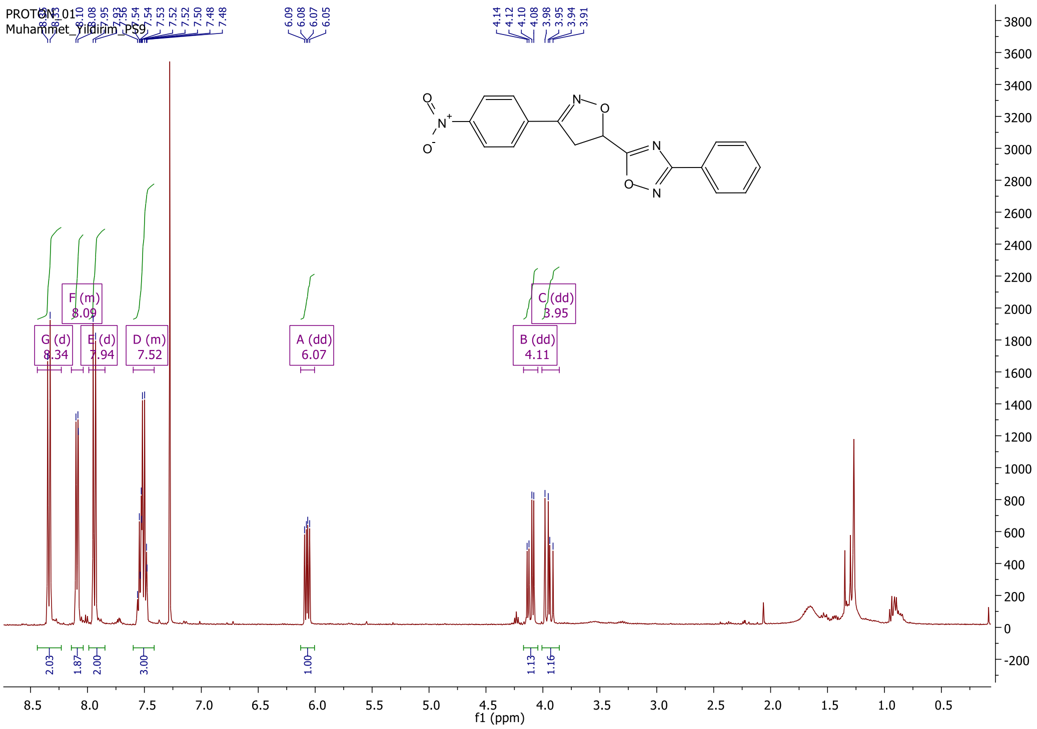

Figure S170. ^{1}H-NMR Spectrum of compound **7ai**

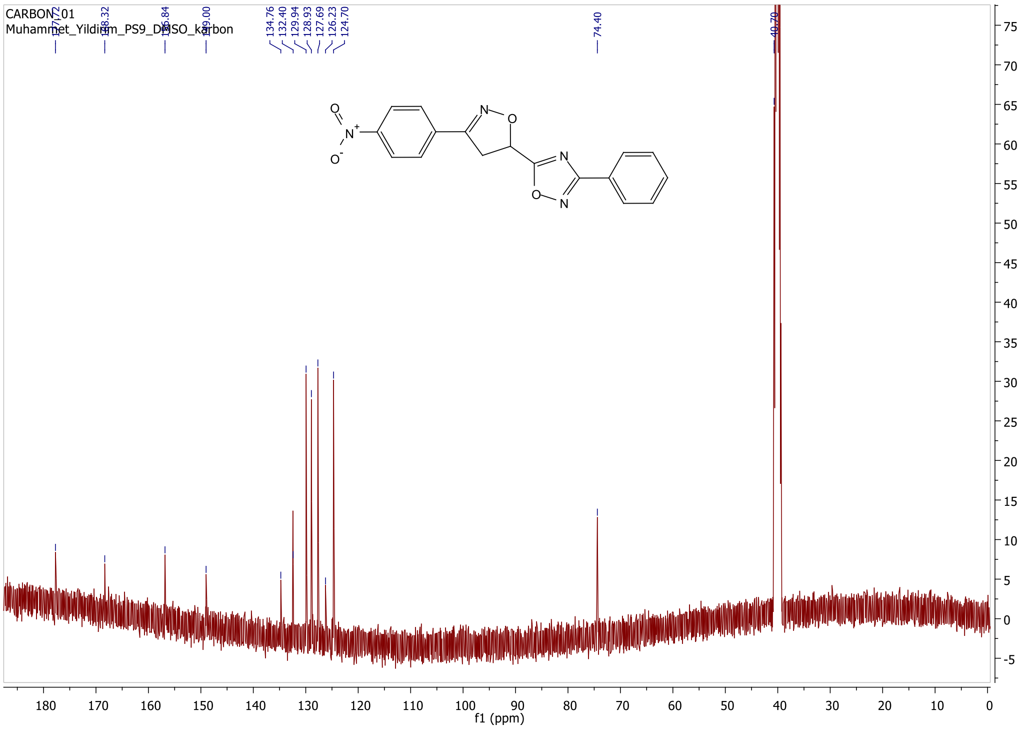

Figure S171. ^{13}C-NMR Spectrum of compound **7ai**

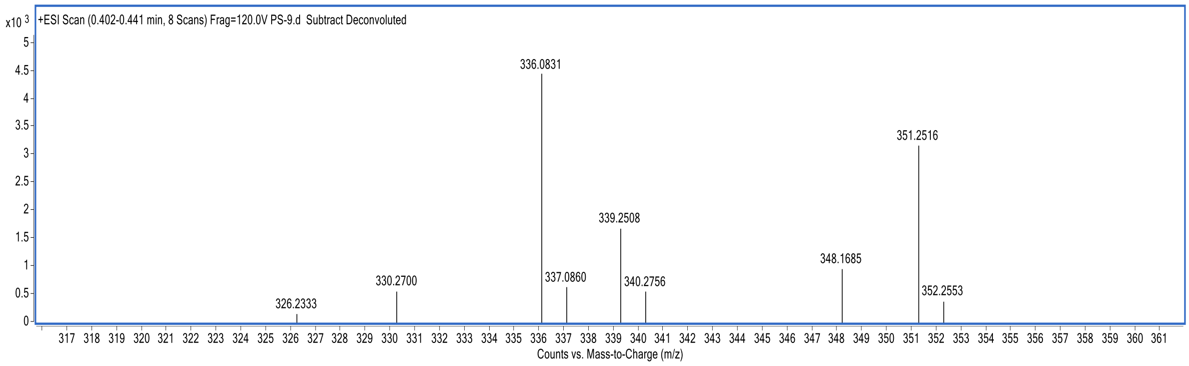

Figure S172. HRMS Spectrum of compound **7ai**

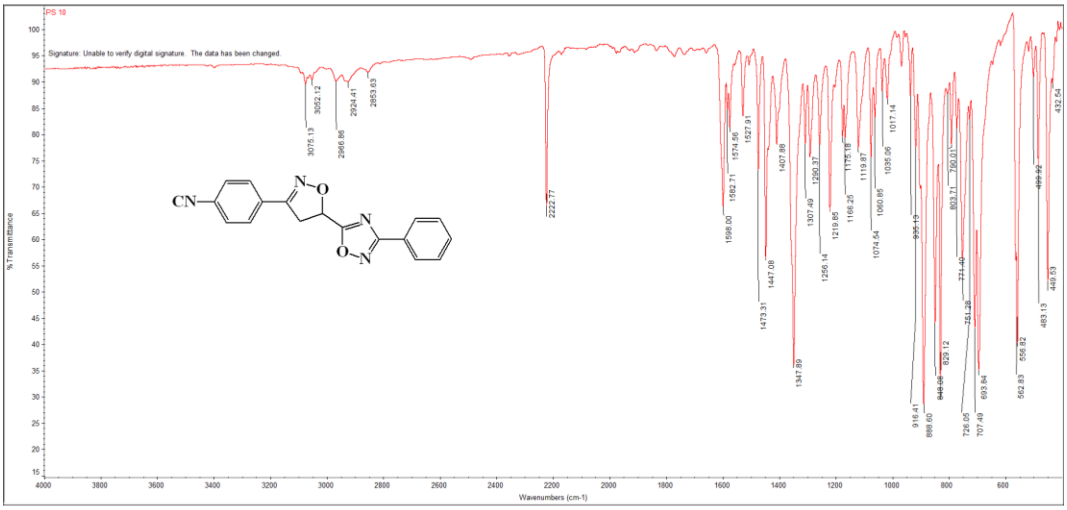

Figure S173. IR Spectrum of compound **7aj**

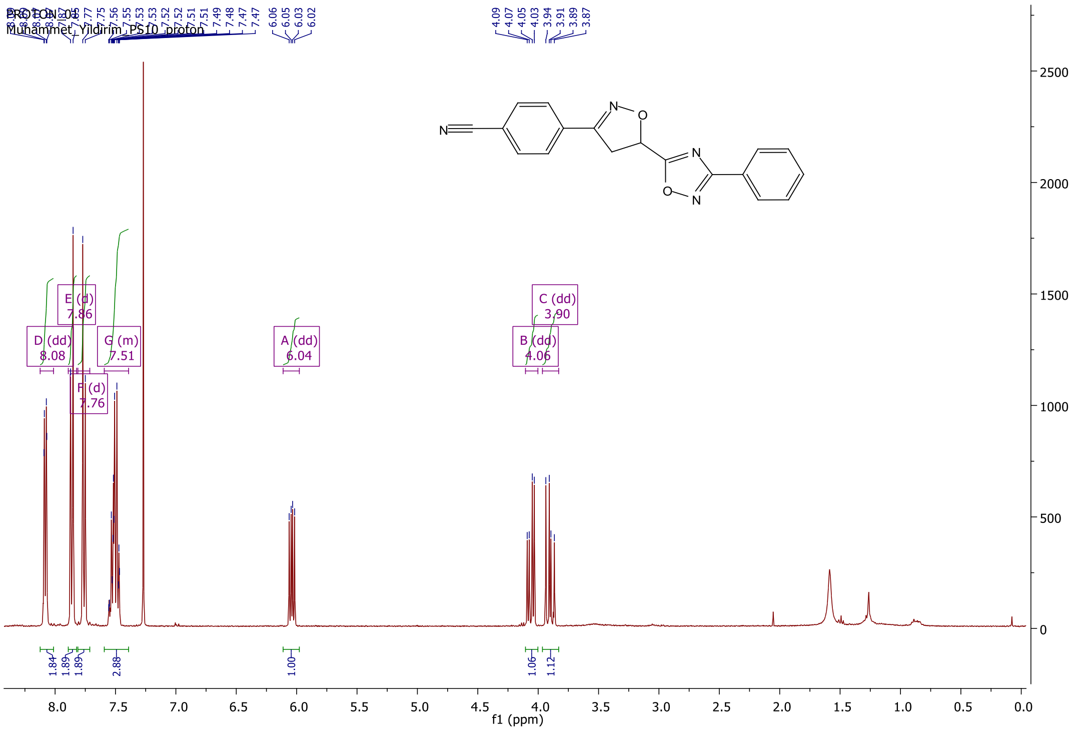

Figure S174. ^{1}H-NMR Spectrum of compound **7aj**

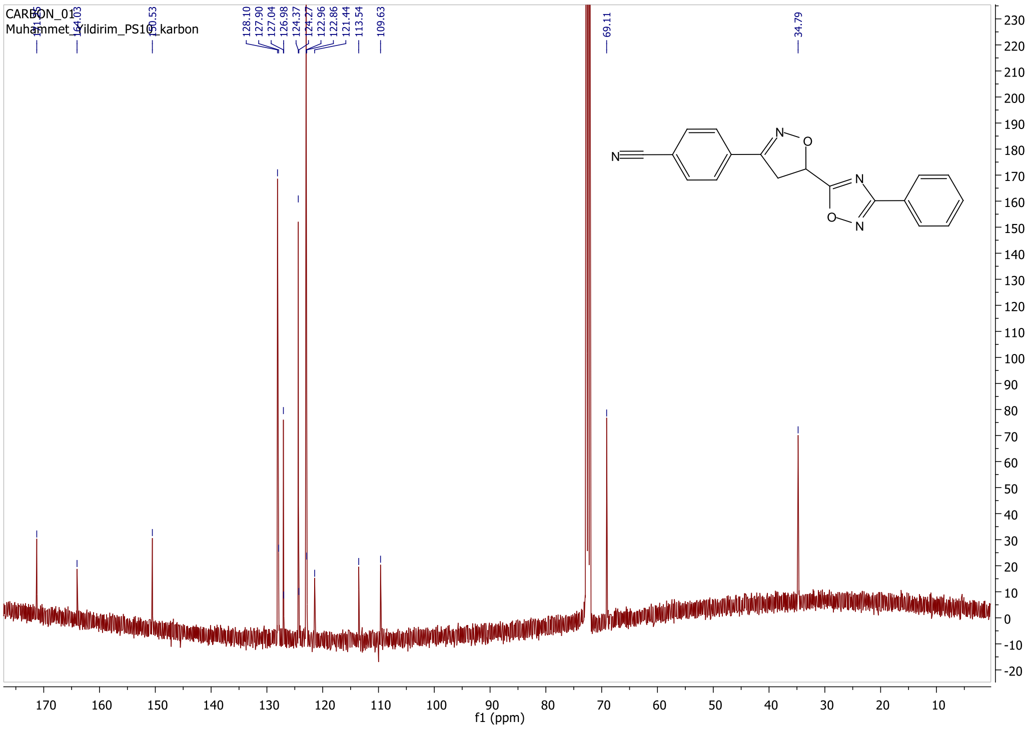

Figure S175. ^{13}C-NMR Spectrum of compound **7aj**

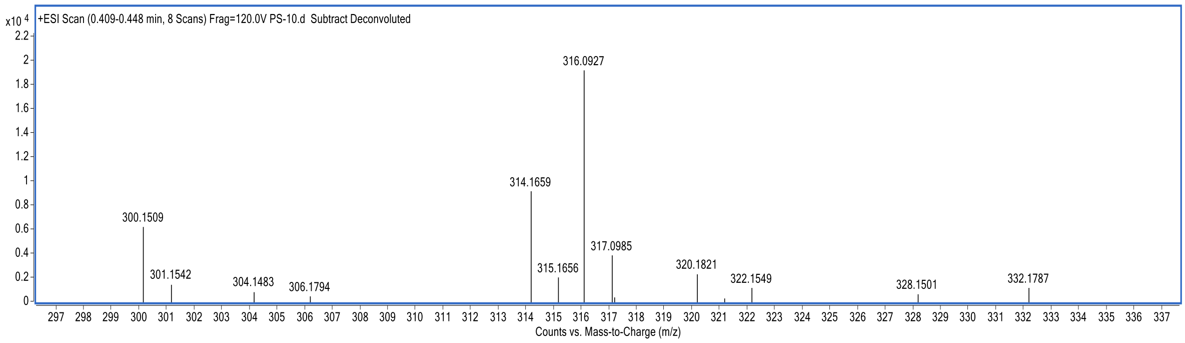

Figure S176. HRMS Spectrum of compound **7aj**

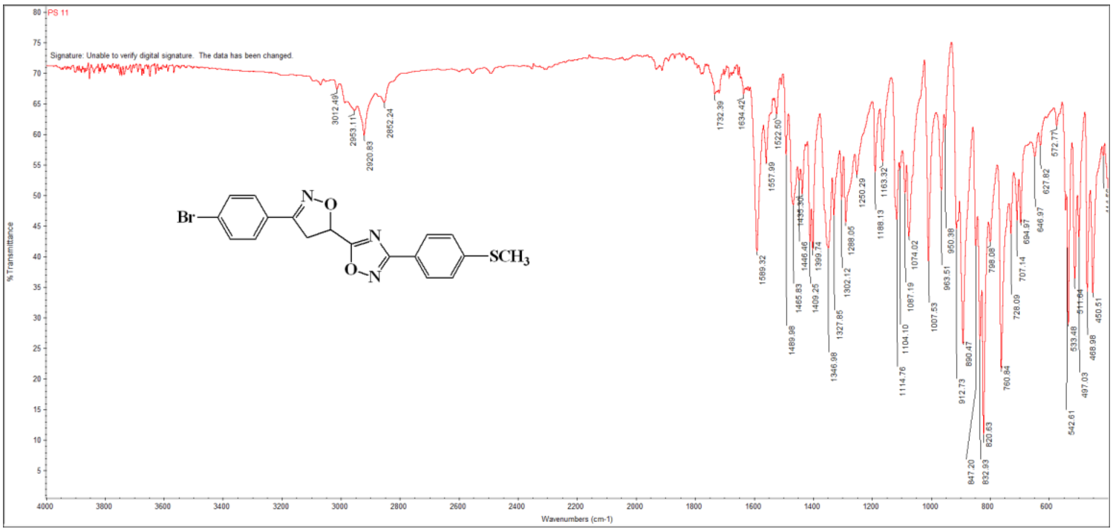

Figure S177. IR Spectrum of compound **7ak**

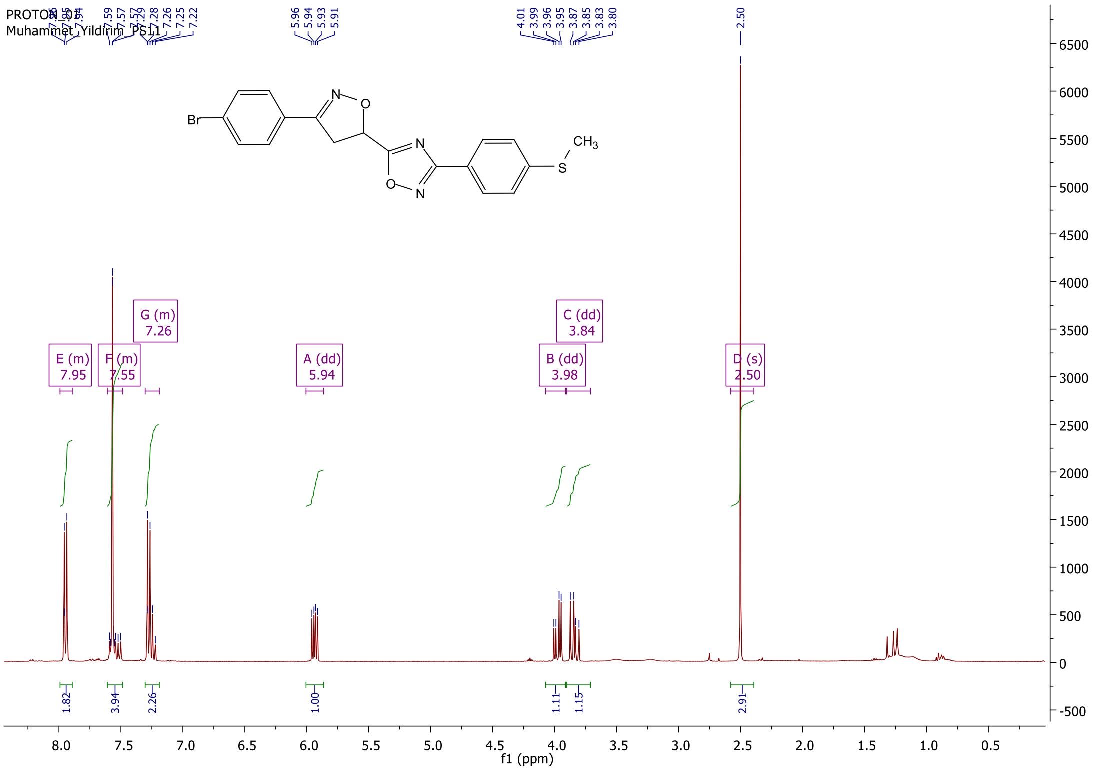

Figure S178. ^{1}H-NMR Spectrum of compound **7ak**

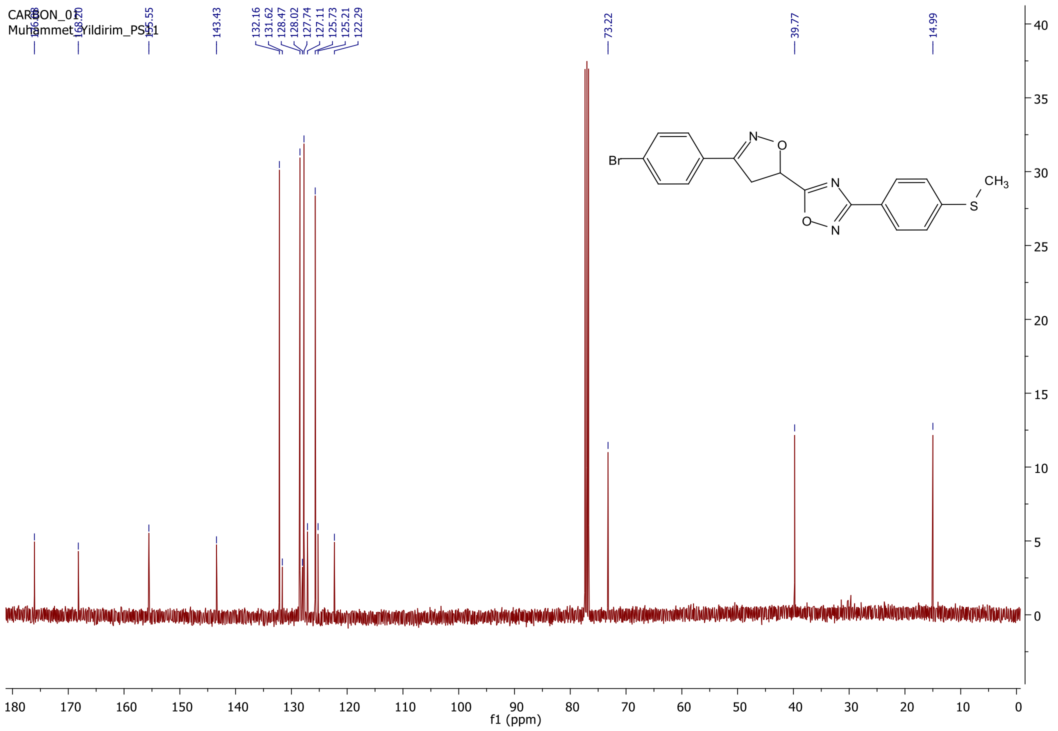

Figure S179. ^{13}C-NMR Spectrum of compound **7ak**

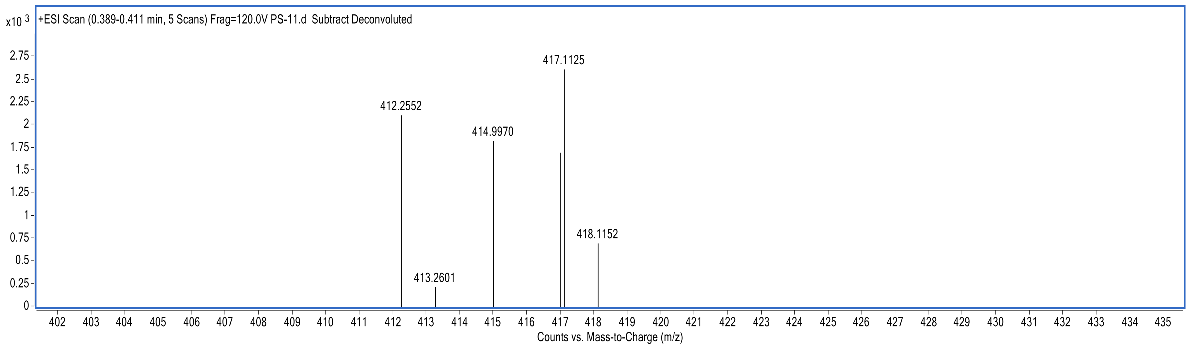

Figure S180. HRMS Spectrum of compound **7ak**

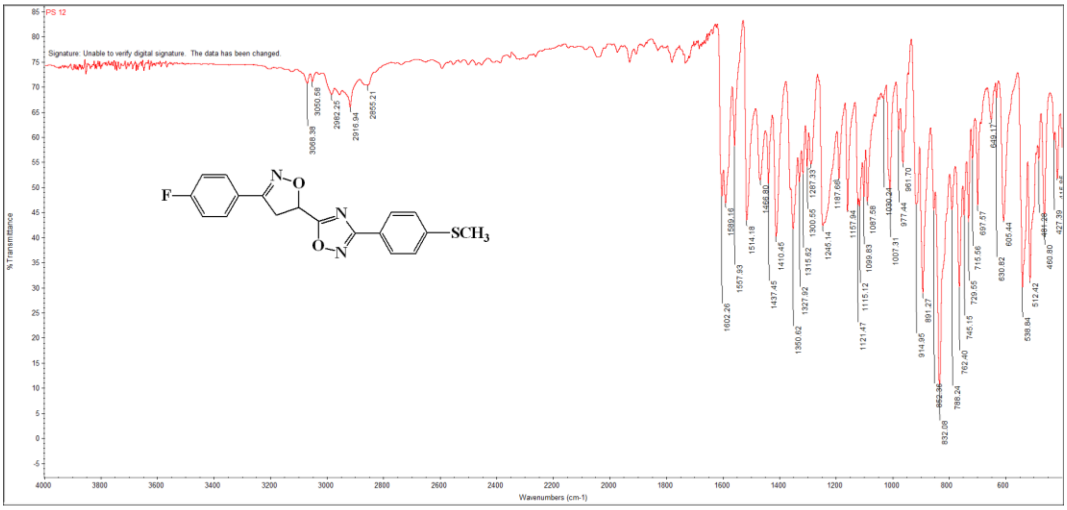

Figure S181. IR Spectrum of compound **7al**

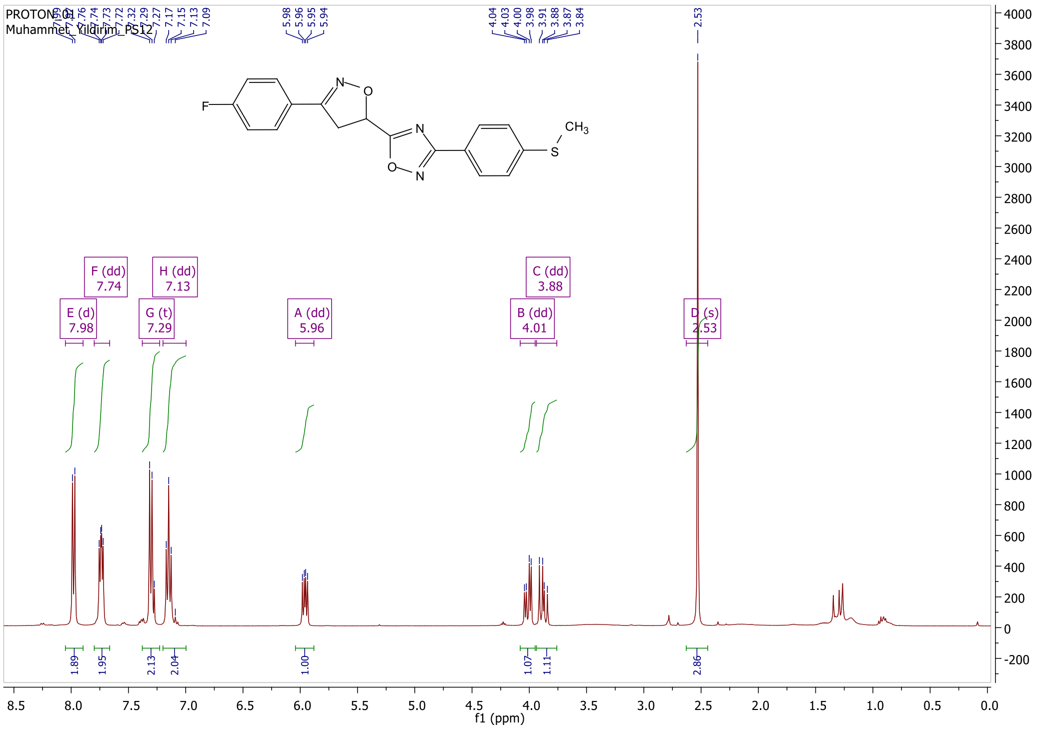

Figure S182. ^{1}H-NMR Spectrum of compound **7al**

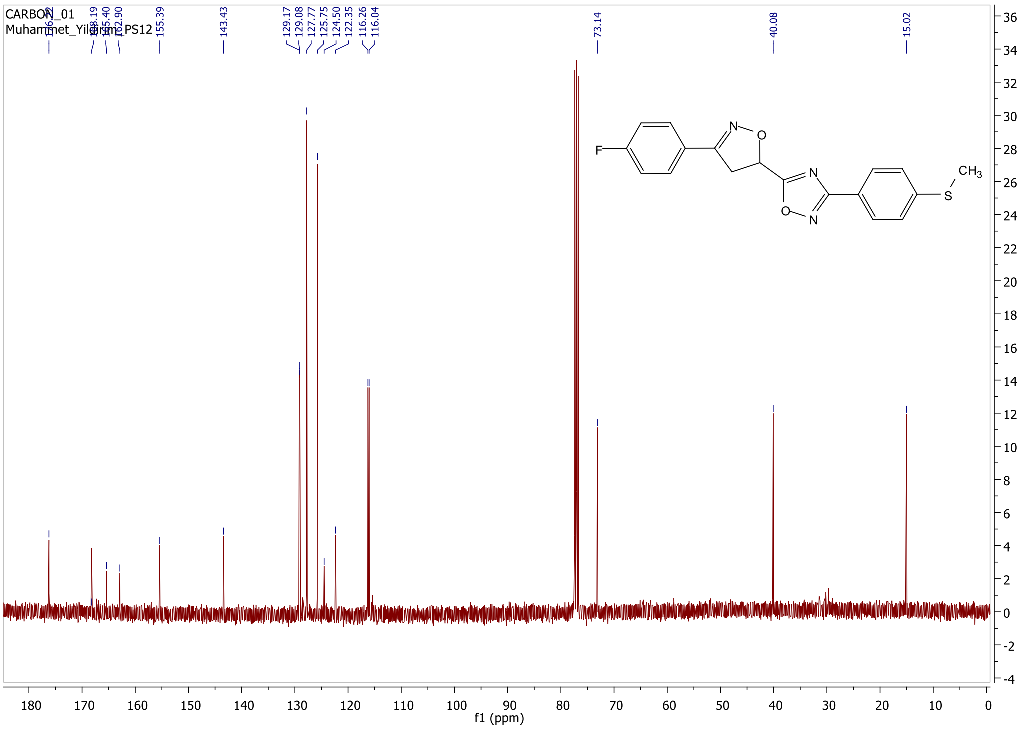

Figure S183. ^{13}C-NMR Spectrum of compound **7al**

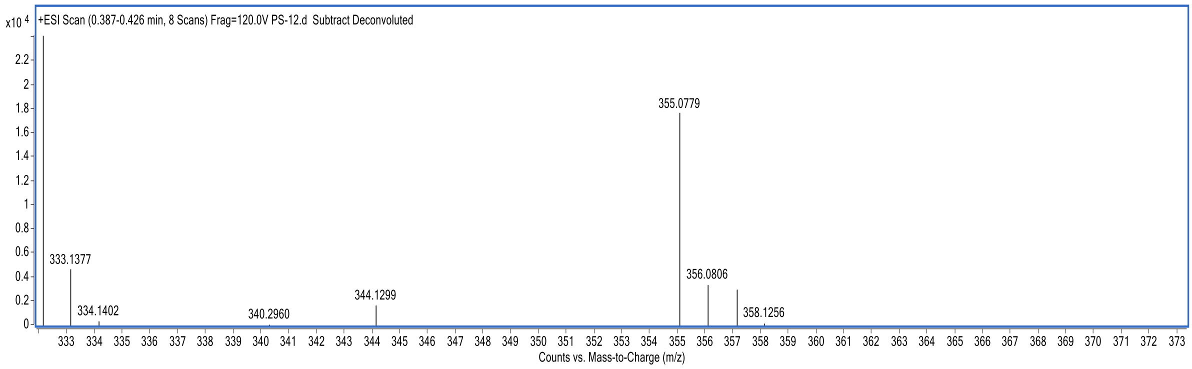

Figure S184. HRMS Spectrum of compound **7al**

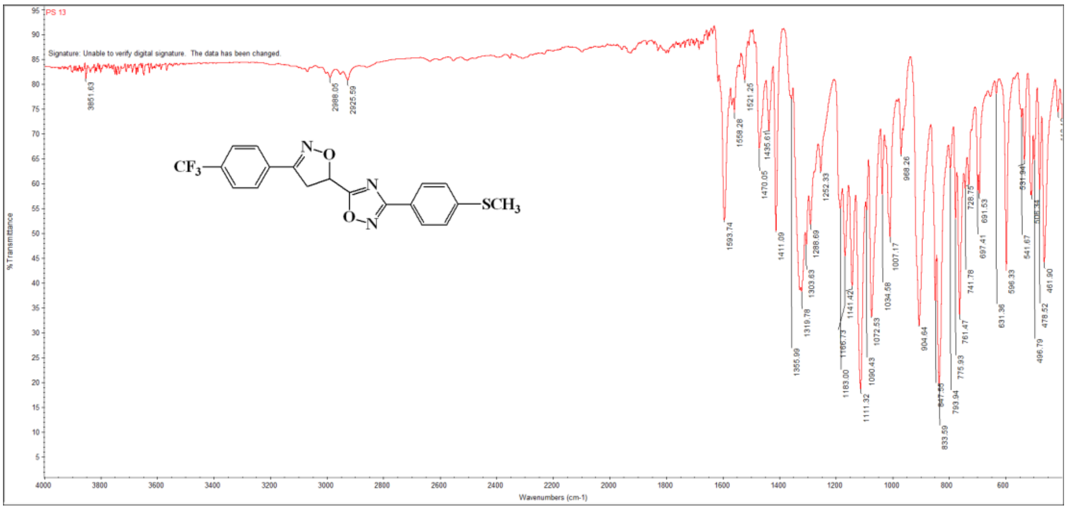

Figure S185. IR Spectrum of compound **7am**

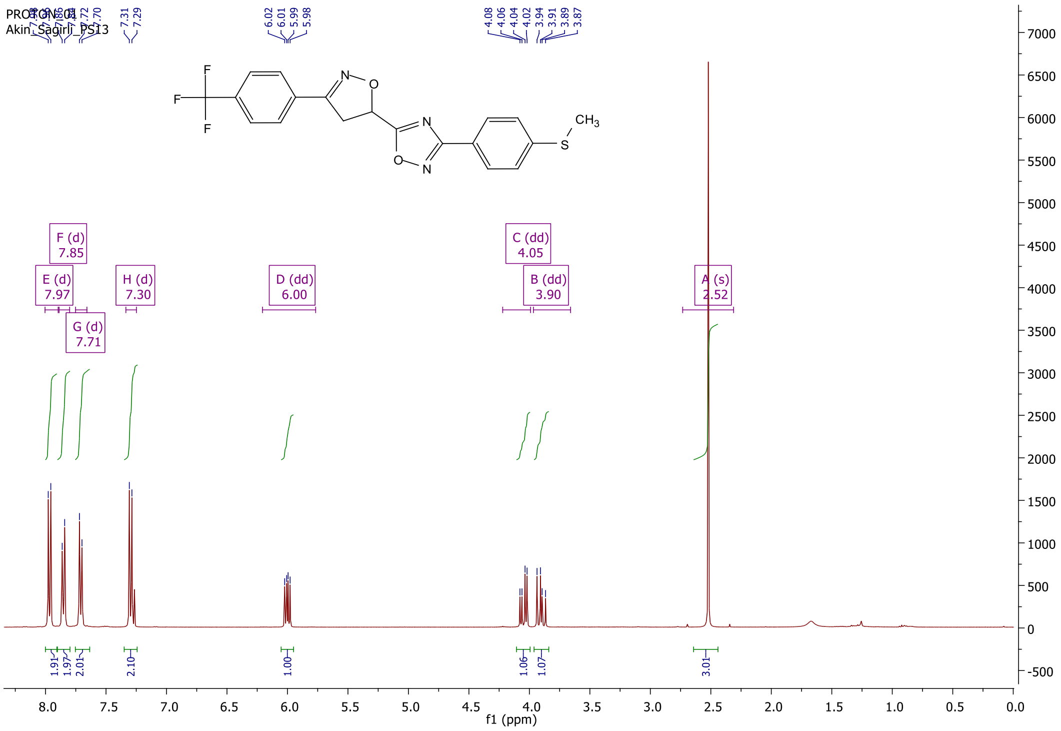

Figure S186. ^{1}H-NMR Spectrum of compound **7am**

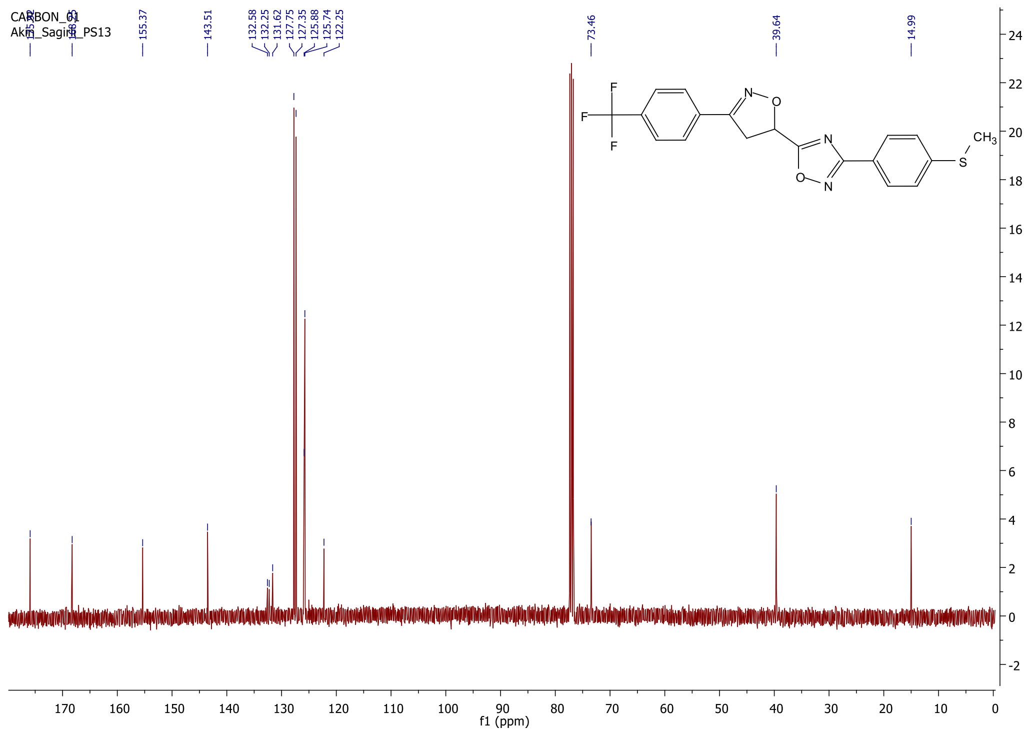

Figure S187. ^{13}C-NMR Spectrum of compound **7am**

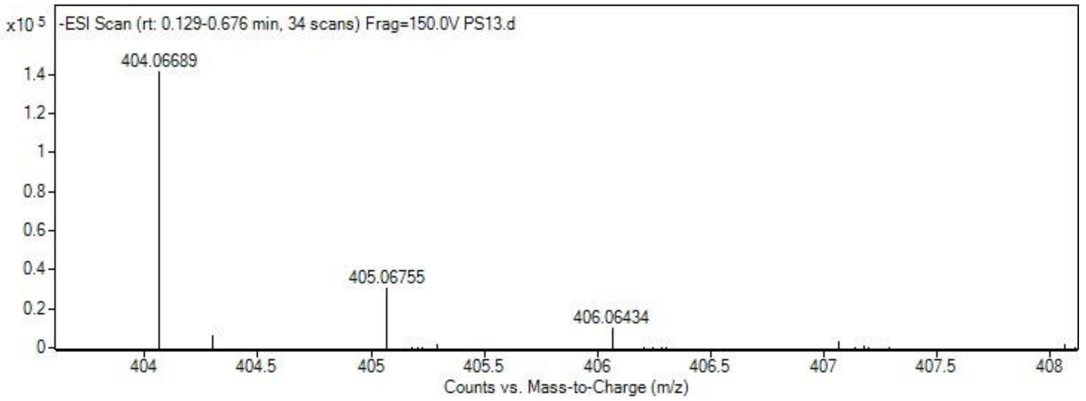

Figure S188. HRMS Spectrum of compound **7am**

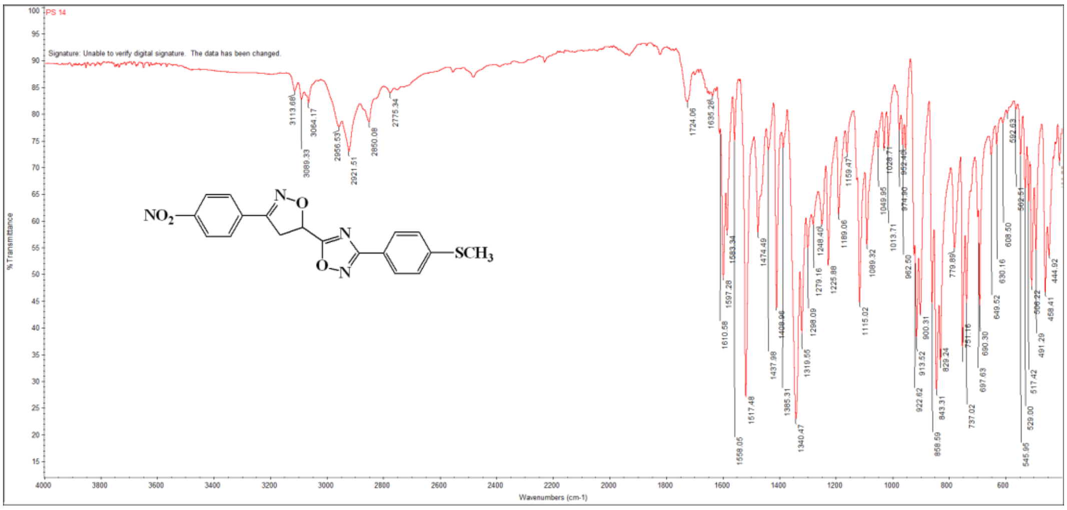

Figure S189. IR Spectrum of compound **7an**

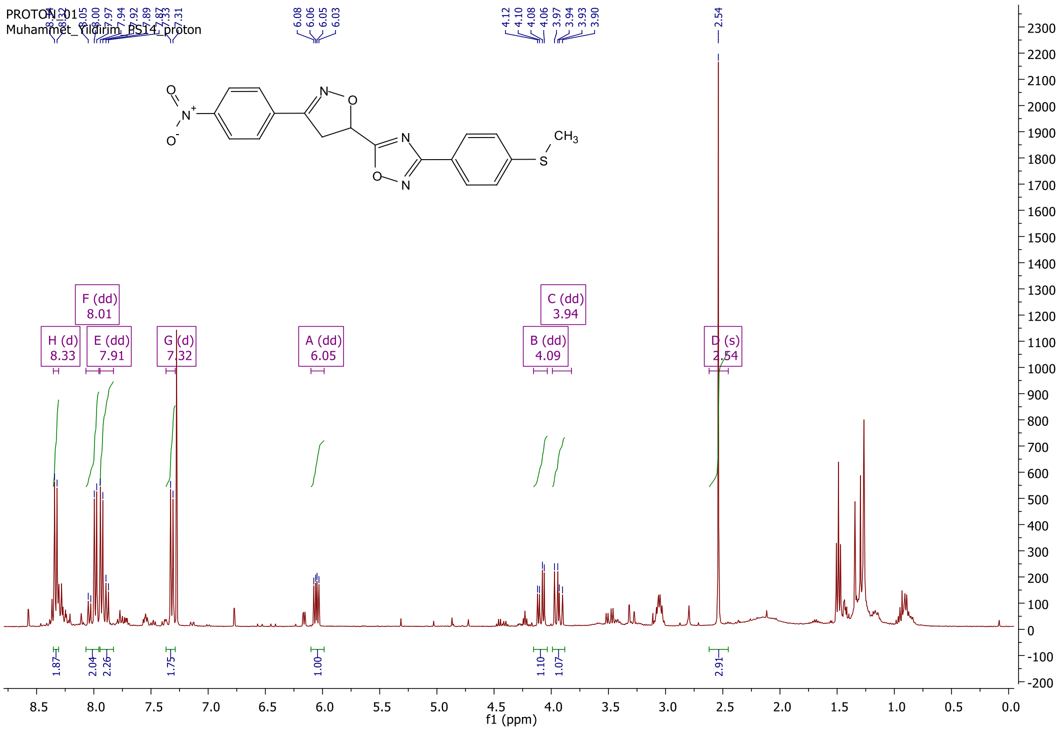

Figure S190. ^{1}H-NMR Spectrum of compound **7an**

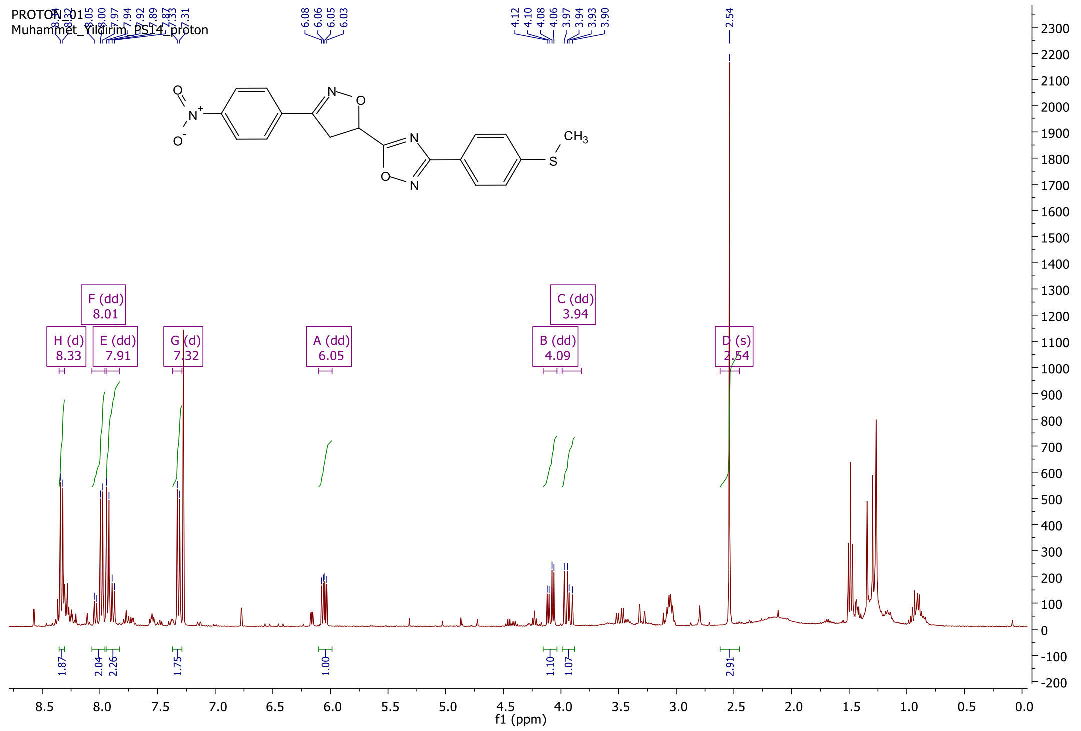

Figure S191. ^{13}C-NMR Spectrum of compound **7an**

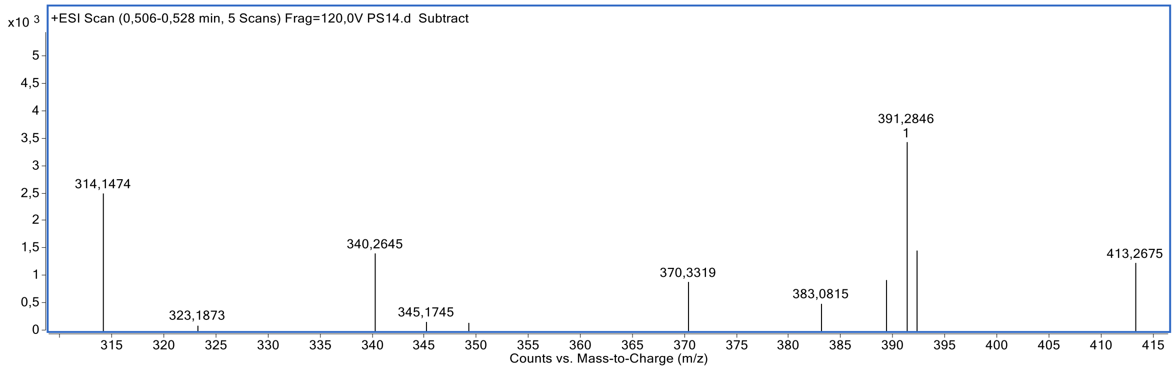

Figure S192. HRMS Spectrum of compound **7an**

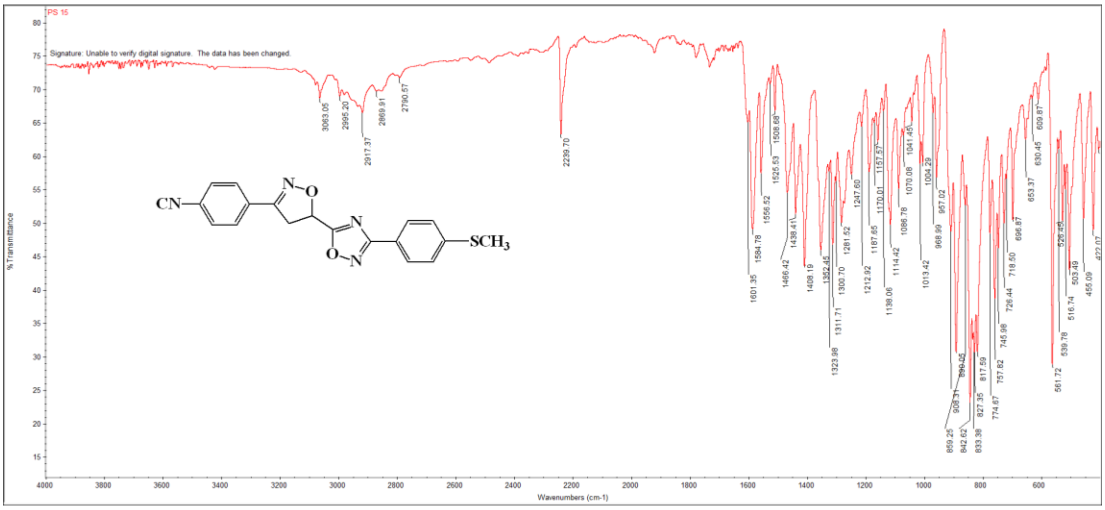

Figure S193. IR Spectrum of compound **7ao**

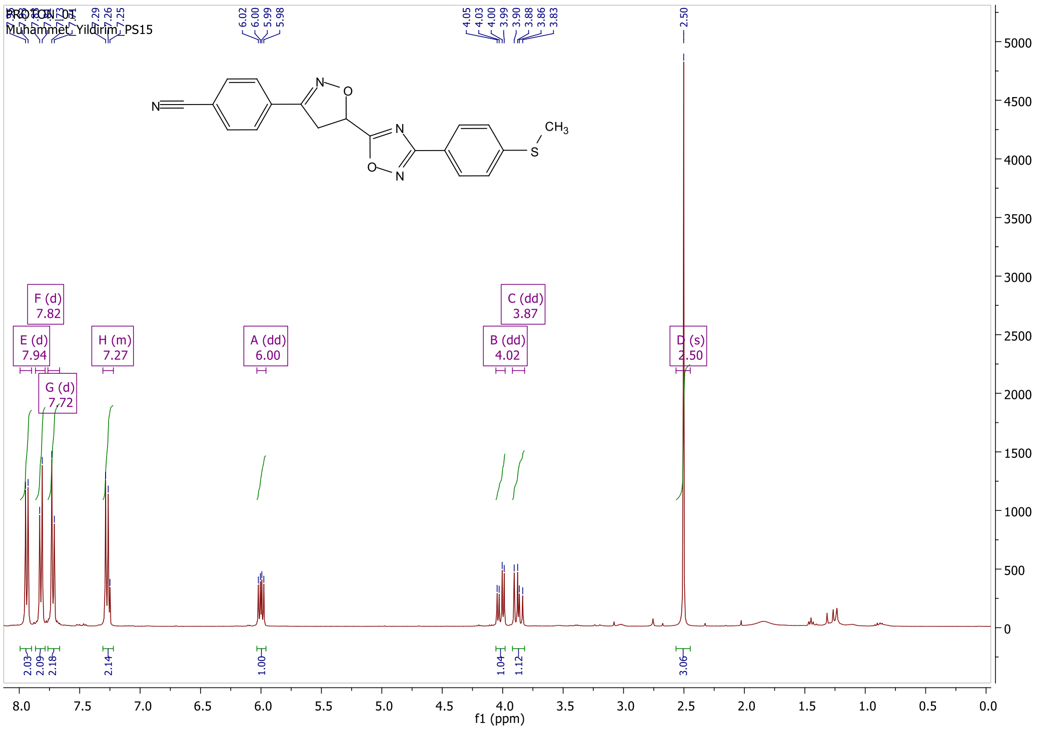

Figure S194. ^{1}H-NMR Spectrum of compound **7ao**

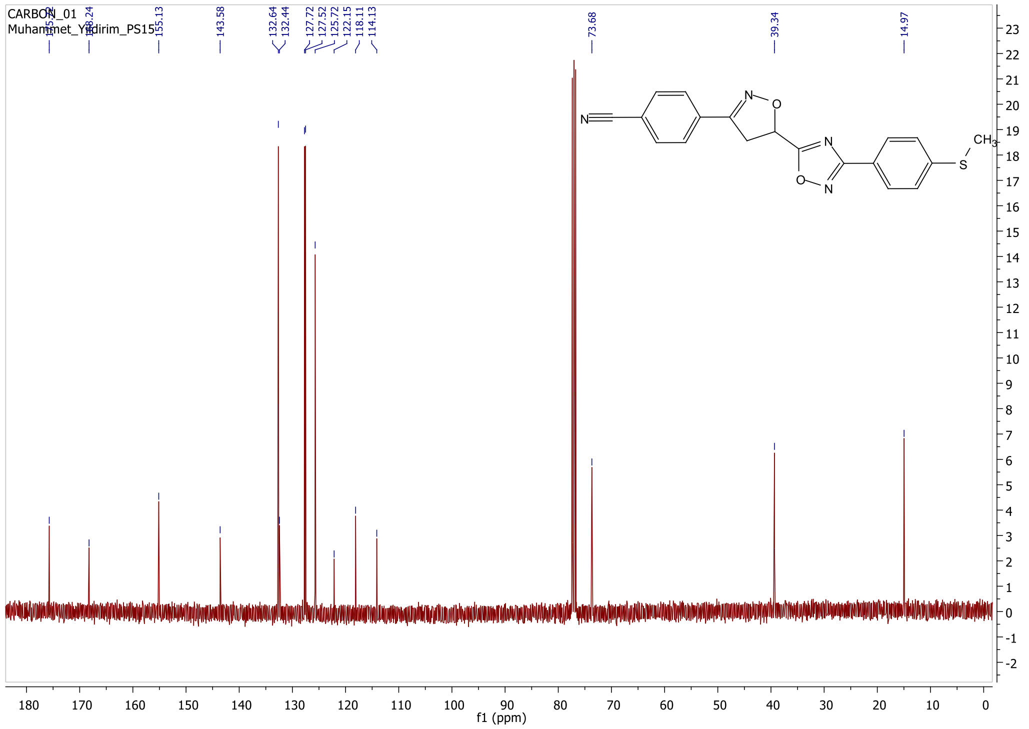

Figure S195. ^{13}C-NMR Spectrum of compound **7ao**

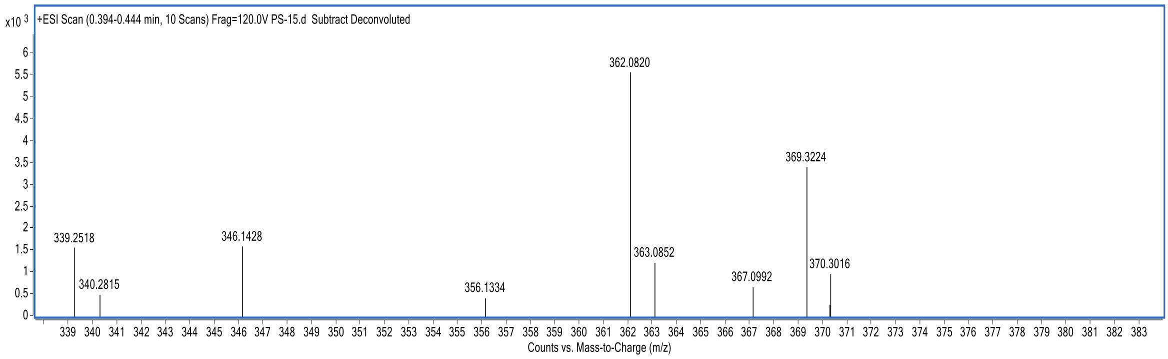

Figure S196. HRMS Spectrum of compound **7ao**

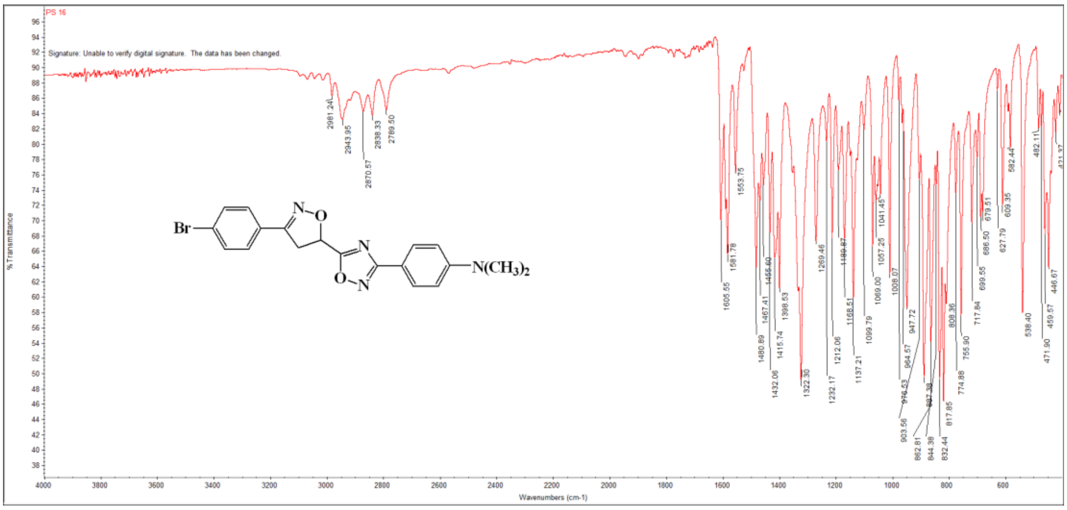

Figure S197. IR Spectrum of compound **7ap**

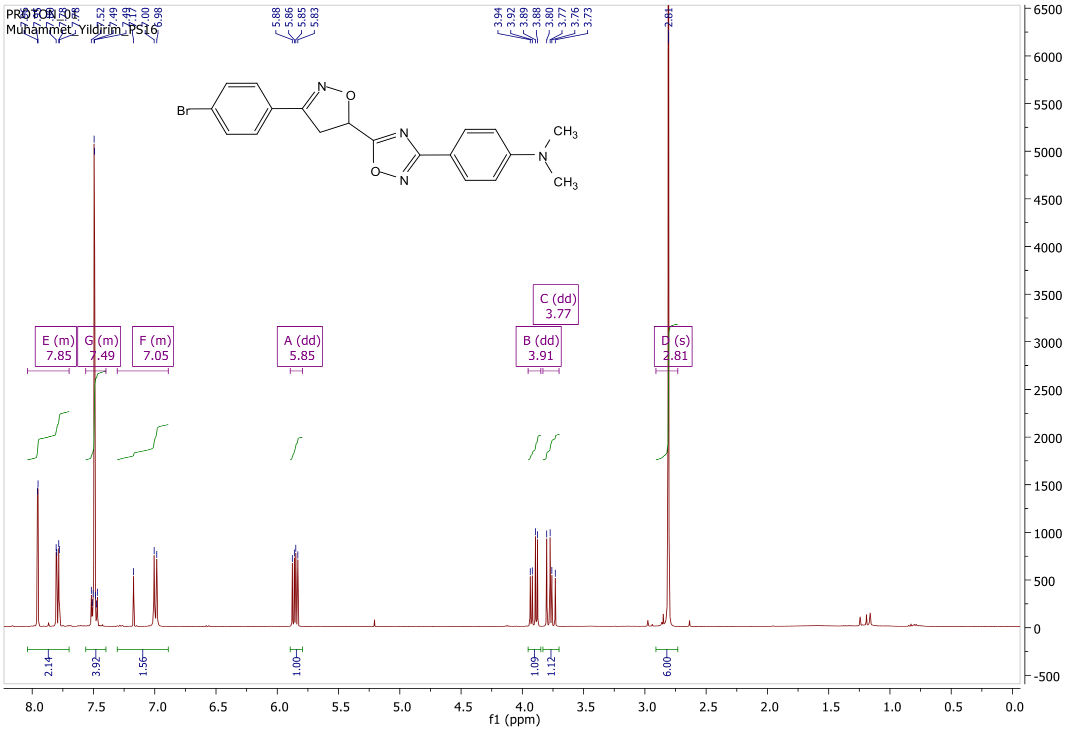

Figure S198. ^{1}H-NMR Spectrum of compound **7ap**

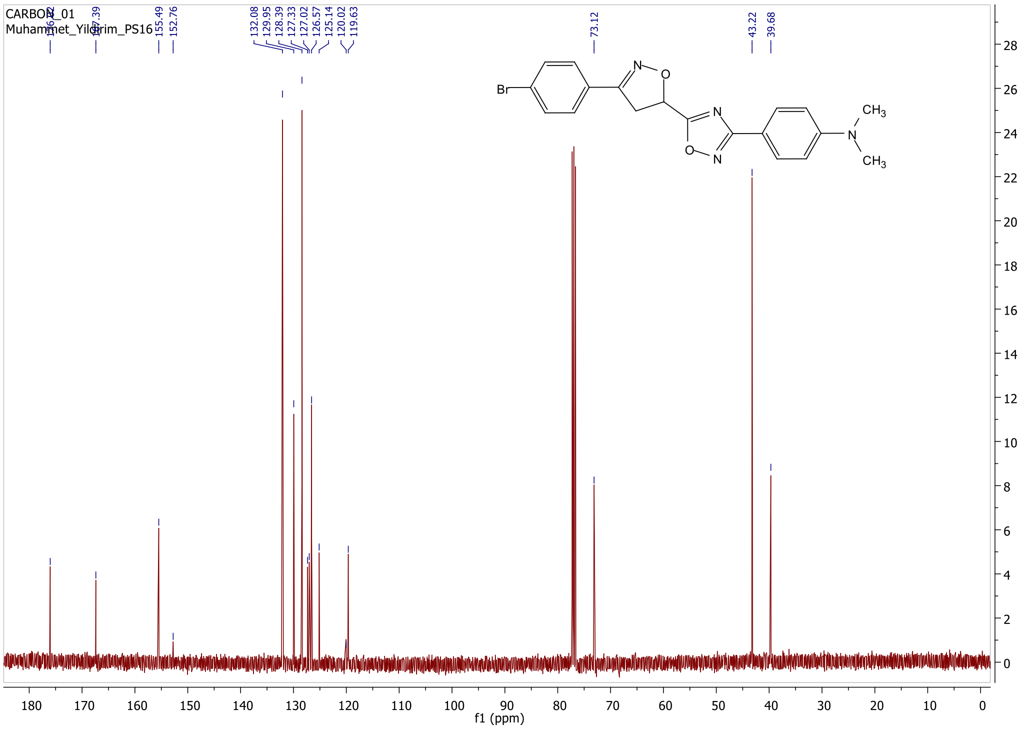

Figure S199. ^{13}C-NMR Spectrum of compound **7ap**

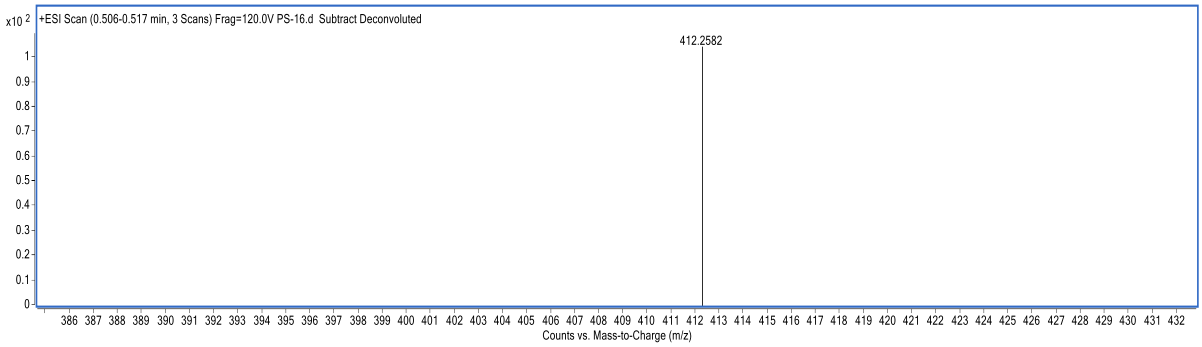

Figure S200. HRMS Spectrum of compound **7ap**

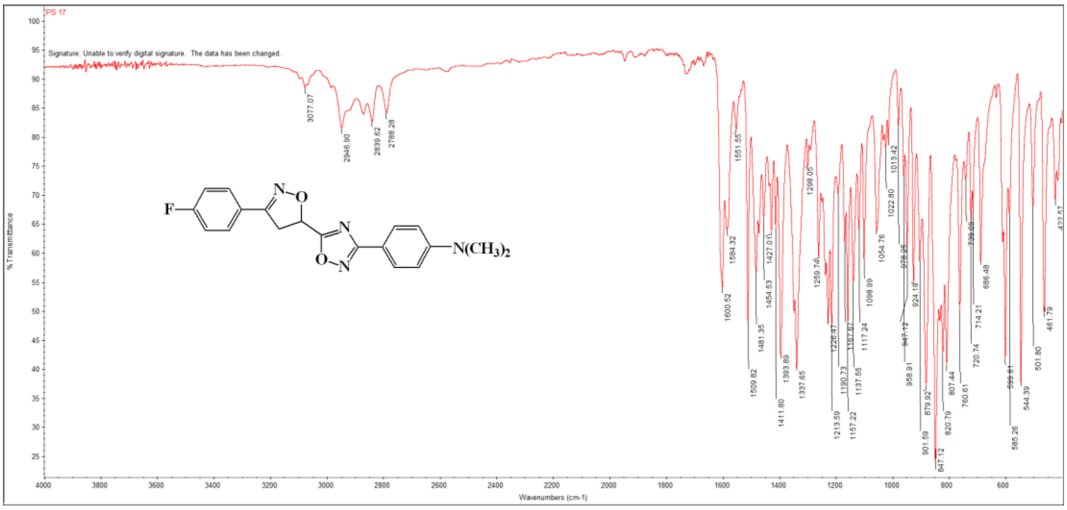

Figure S201. IR Spectrum of compound **7aq**

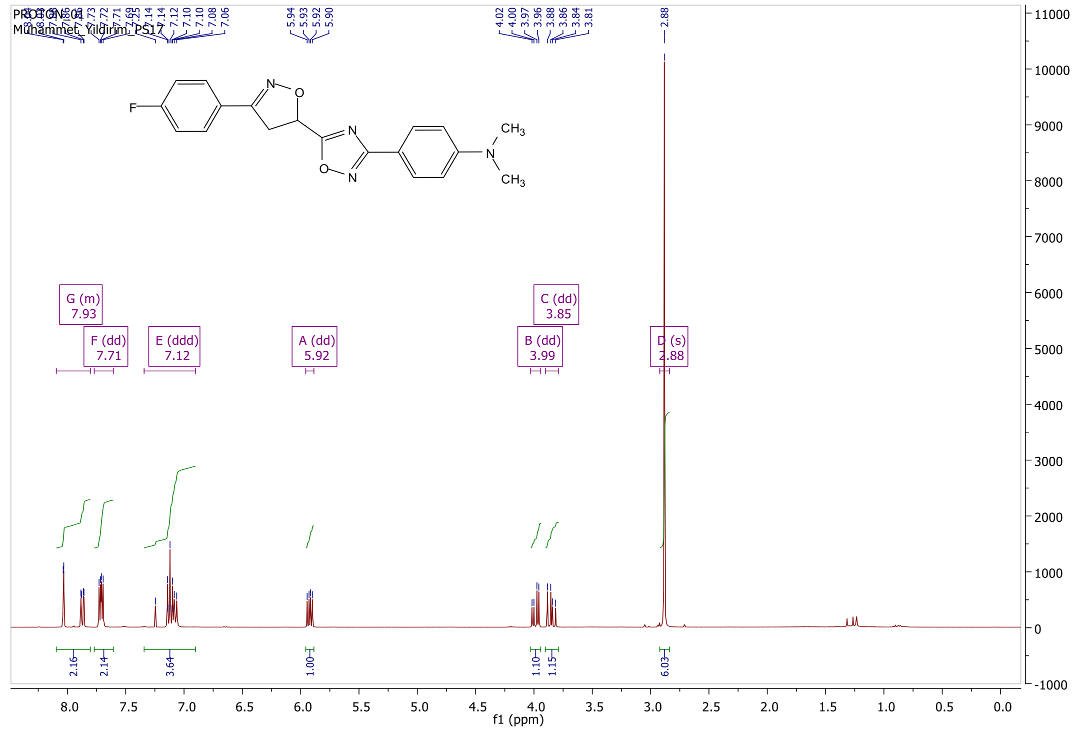

Figure S202. ^{1}H-NMR Spectrum of compound **7aq**

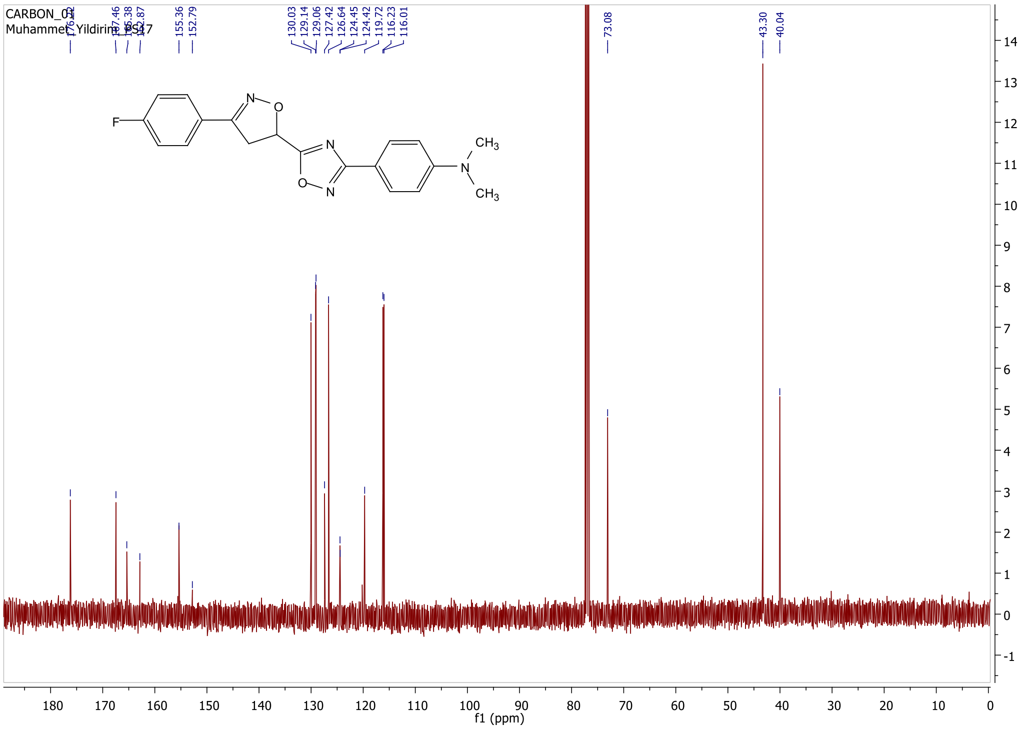

Figure S203. ^{13}C-NMR Spectrum of compound **7aq**

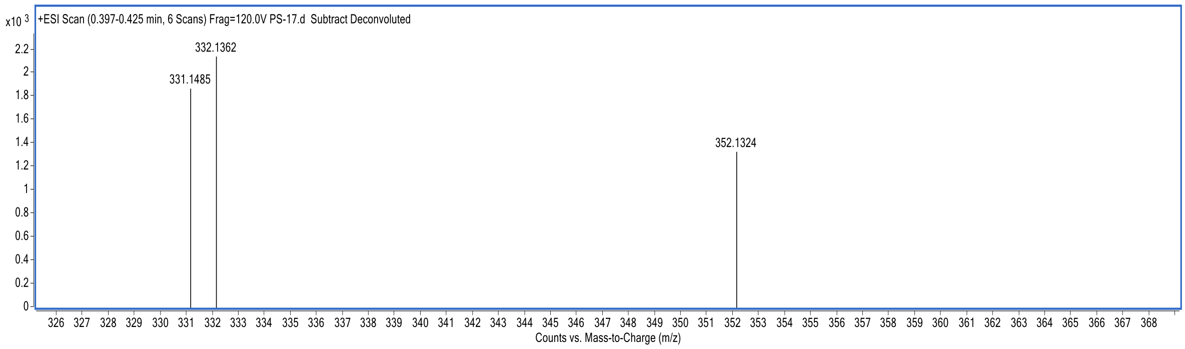

Figure S204. HRMS Spectrum of compound **7aq**

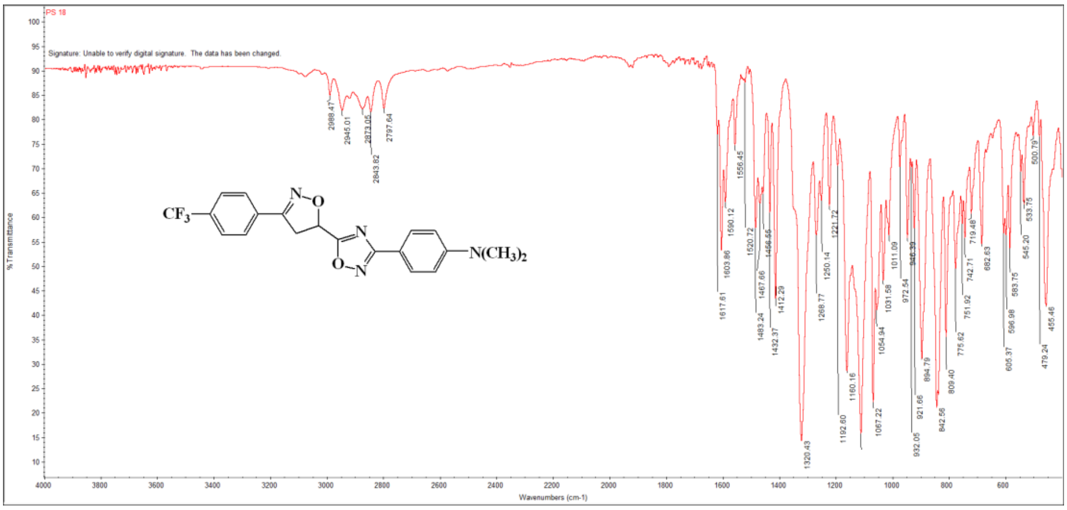

Figure S205. IR Spectrum of compound **7ar**

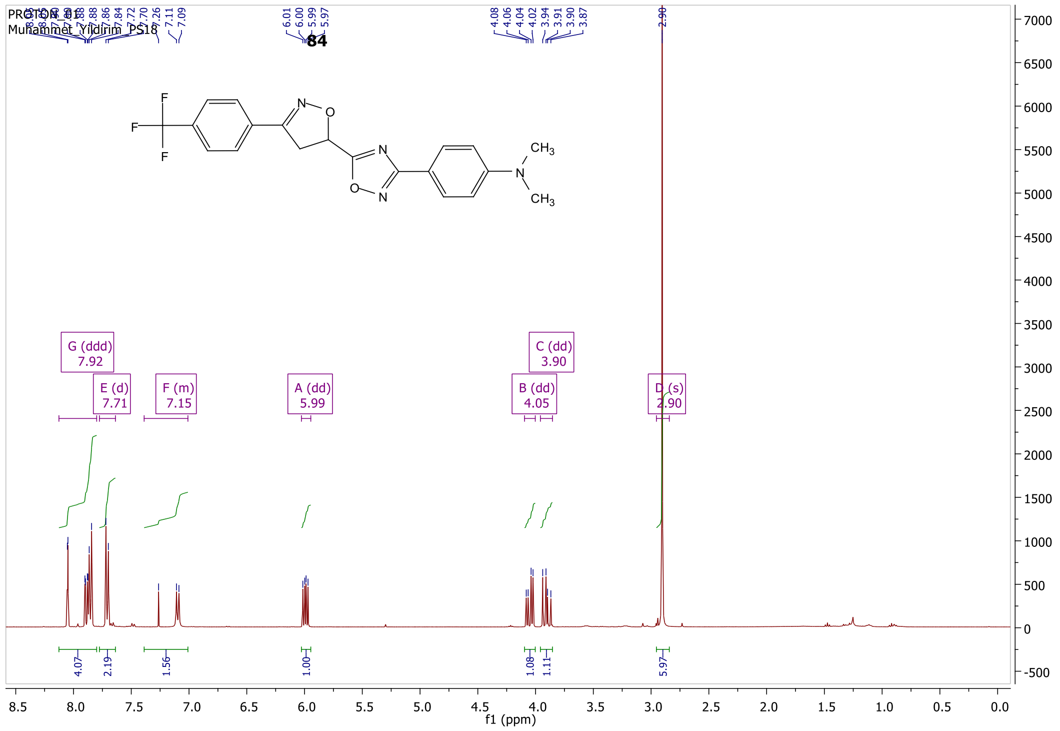

Figure S206. ^{1}H-NMR Spectrum of compound **7ar**

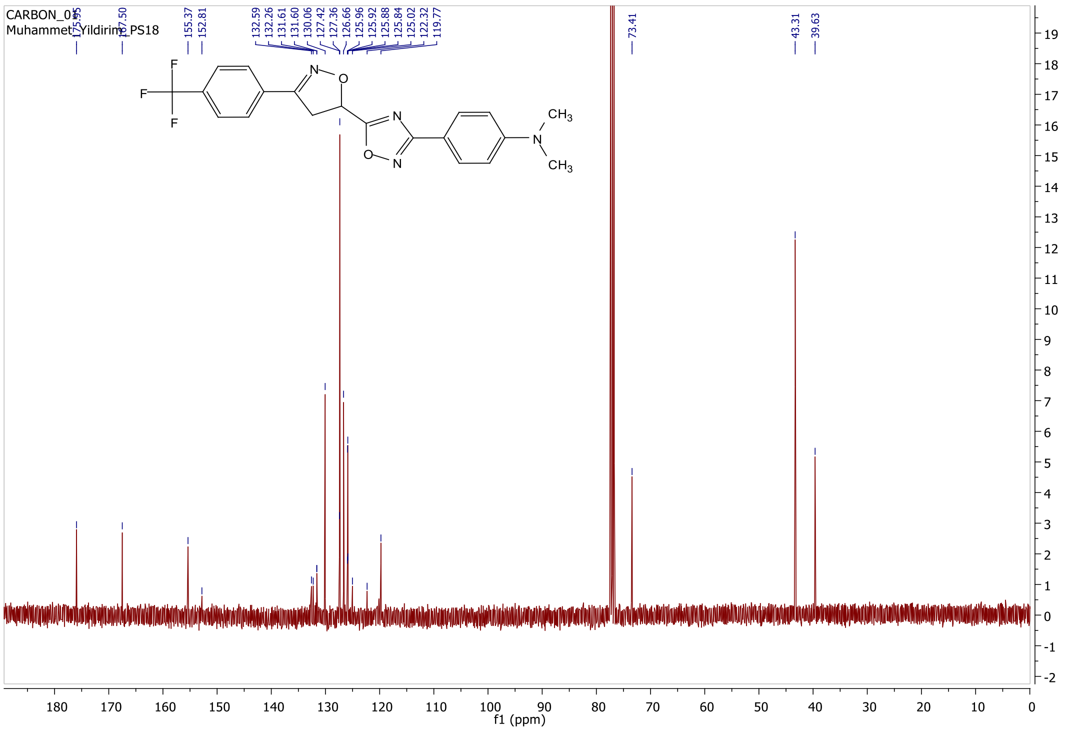

Figure S207. ^{13}C-NMR Spectrum of compound **7ar**

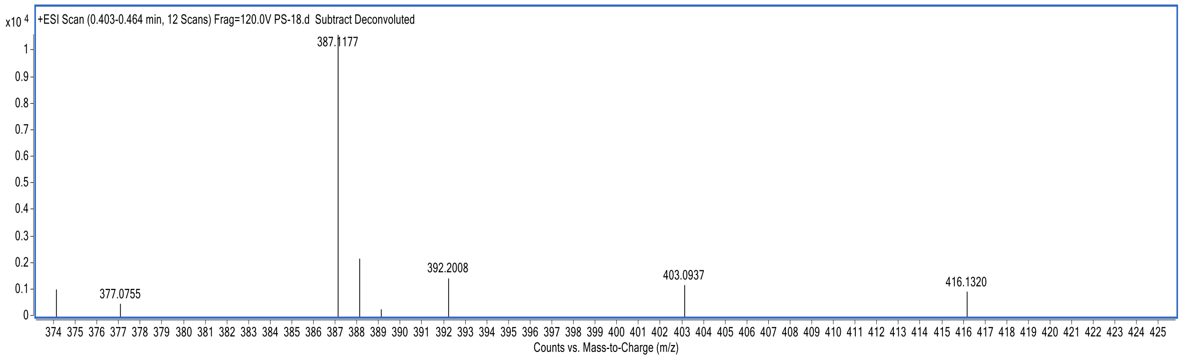

Figure S208. HRMS Spectrum of compound **7ar**

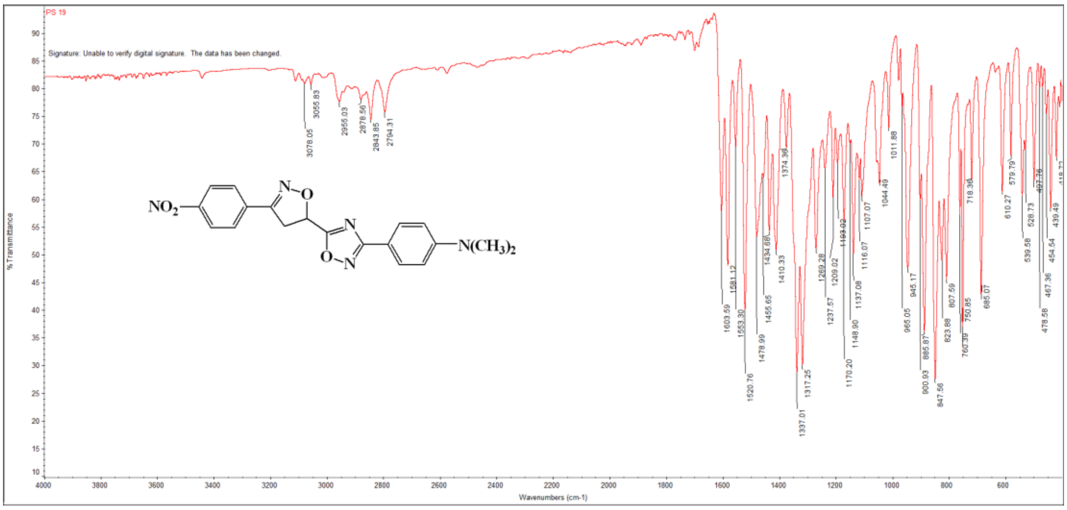

Figure S209. IR Spectrum of compound **7as**

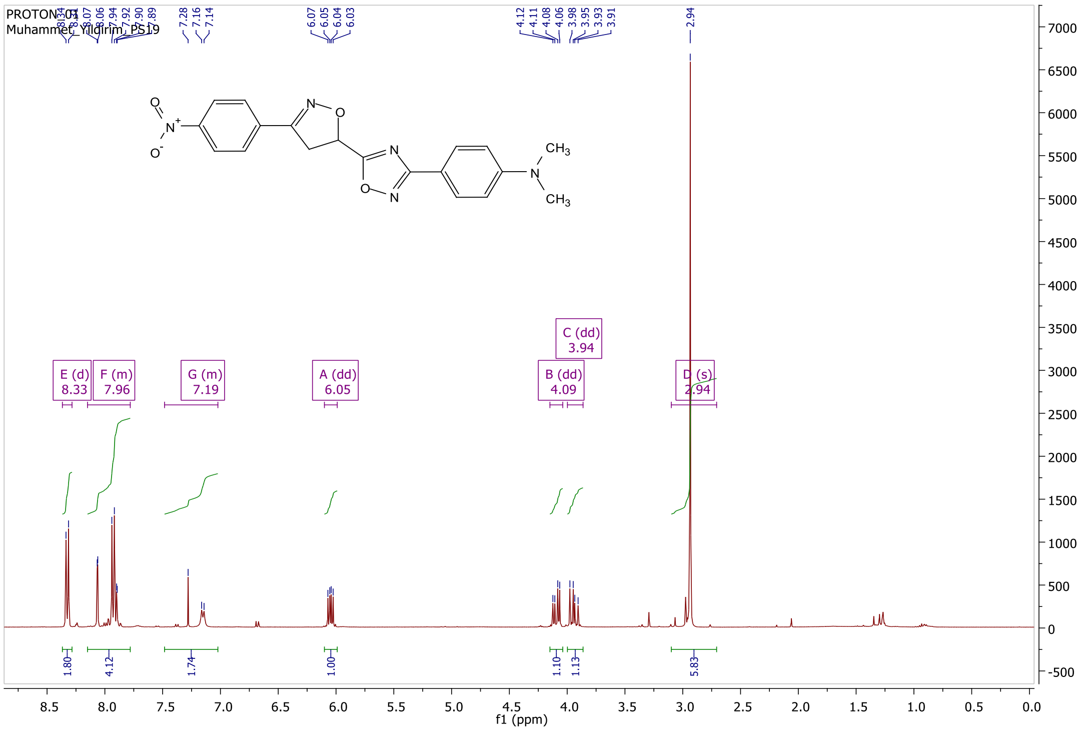

Figure S210. ^{1}H-NMR Spectrum of compound **7as**

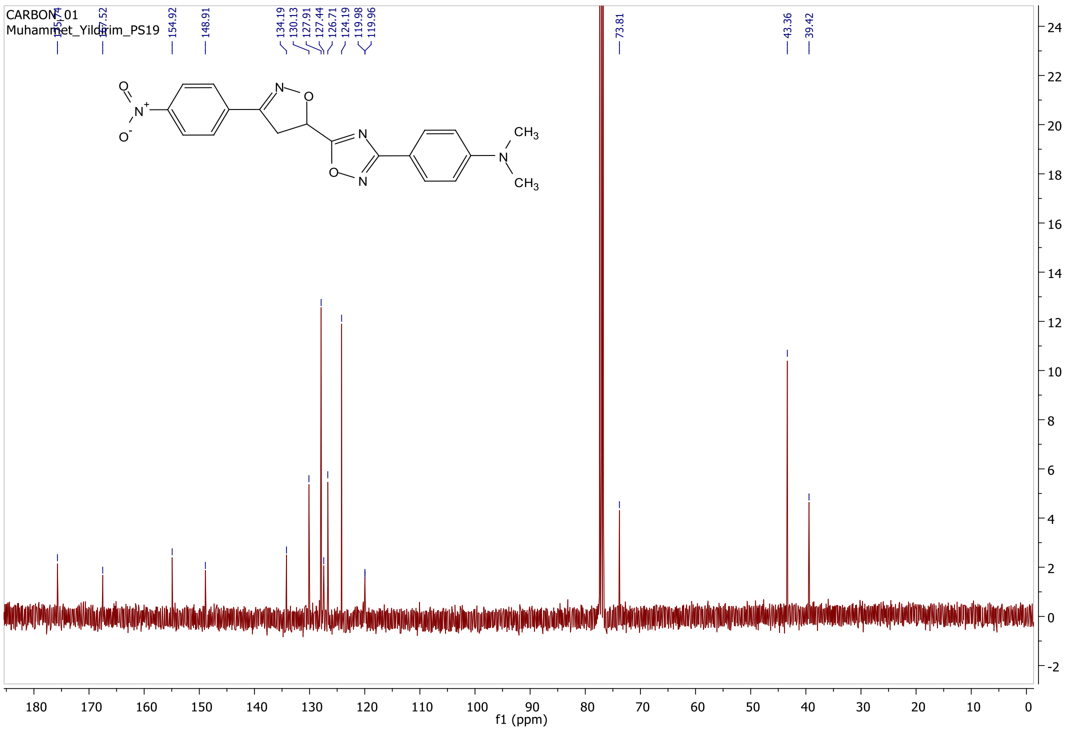

Figure S211. ^{13}C-NMR Spectrum of compound **7as**

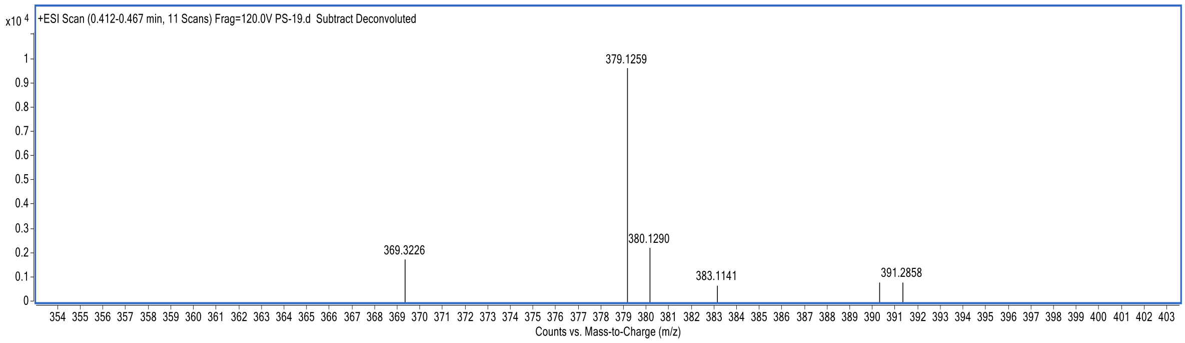

Figure S212. HRMS Spectrum of compound **7as**

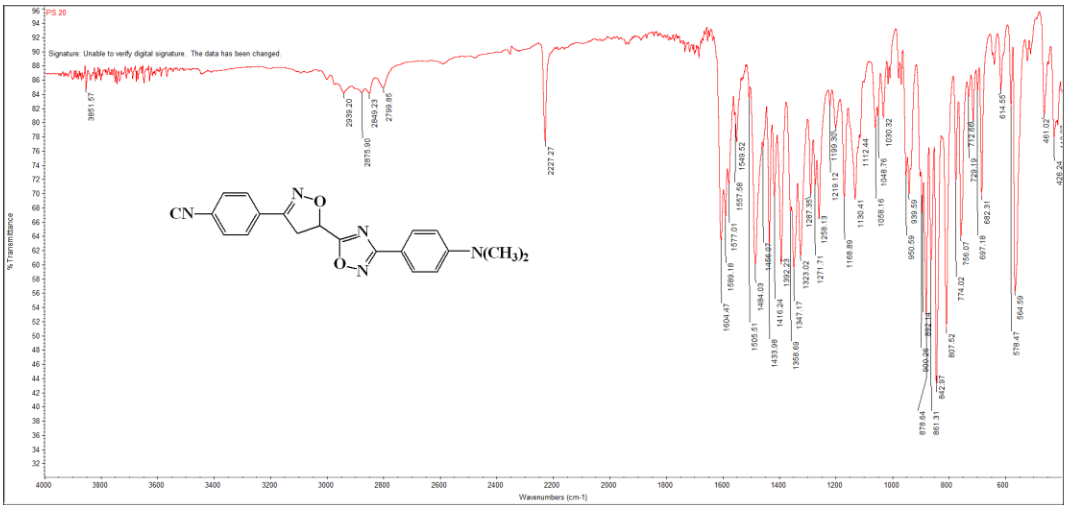

Figure S213. IR Spectrum of compound **7at**

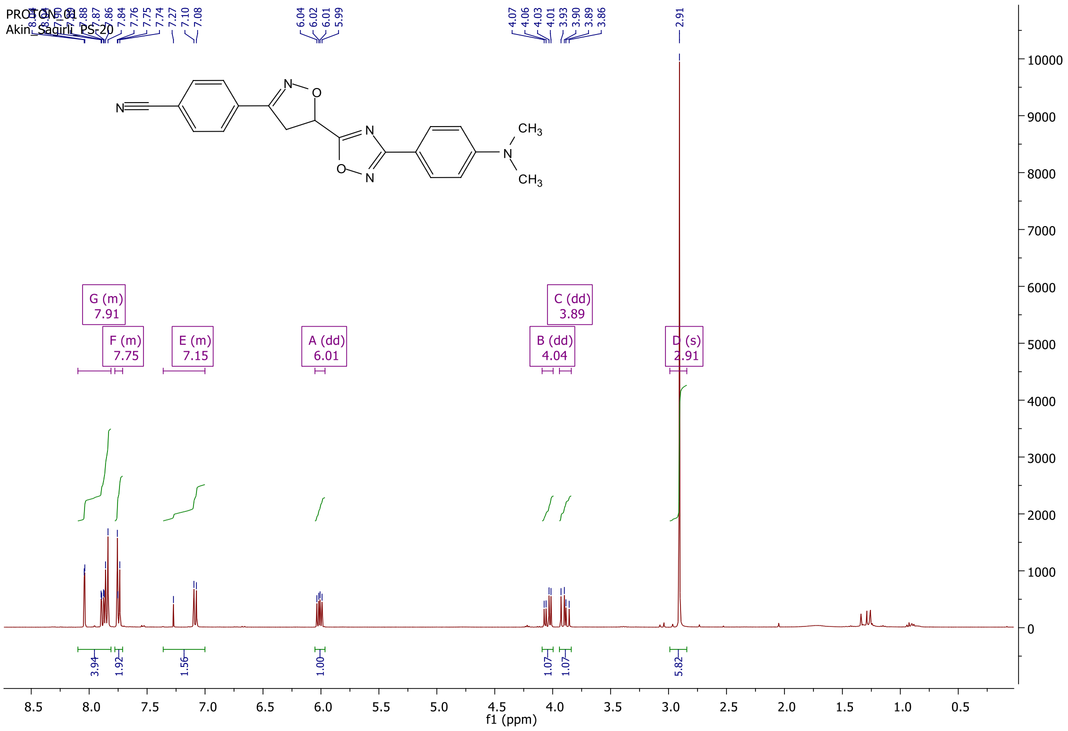

Figure S214. ^{1}H-NMR Spectrum of compound **7at**

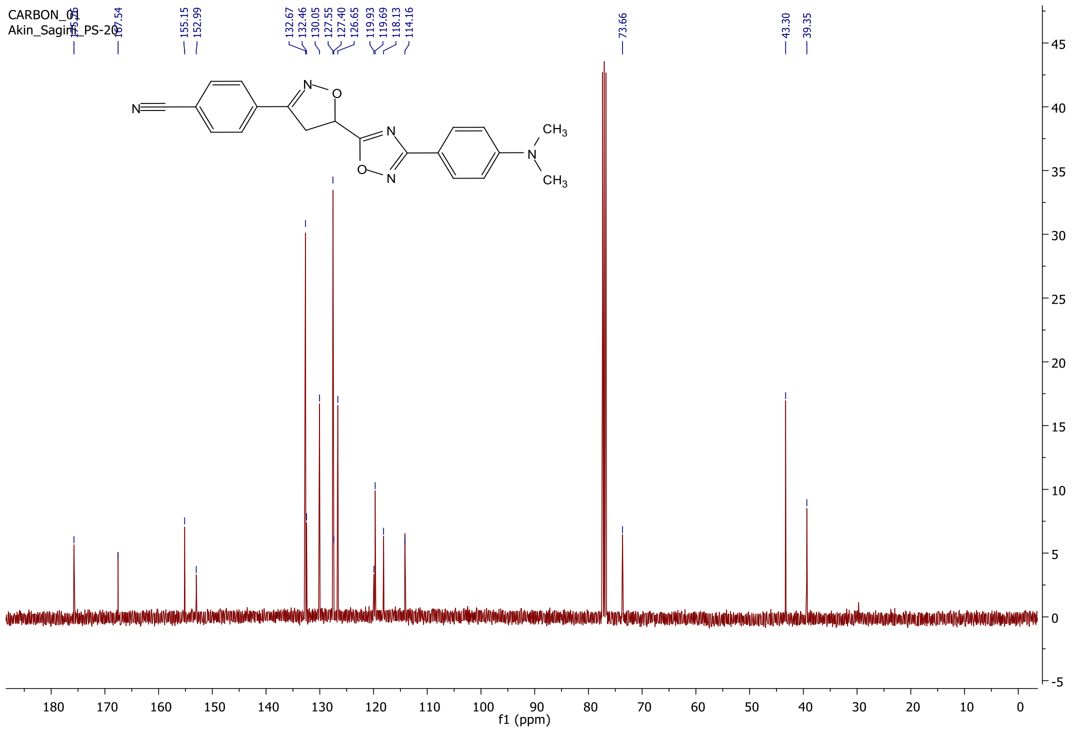

Figure S215. ^{13}C-NMR Spectrum of compound **7at**

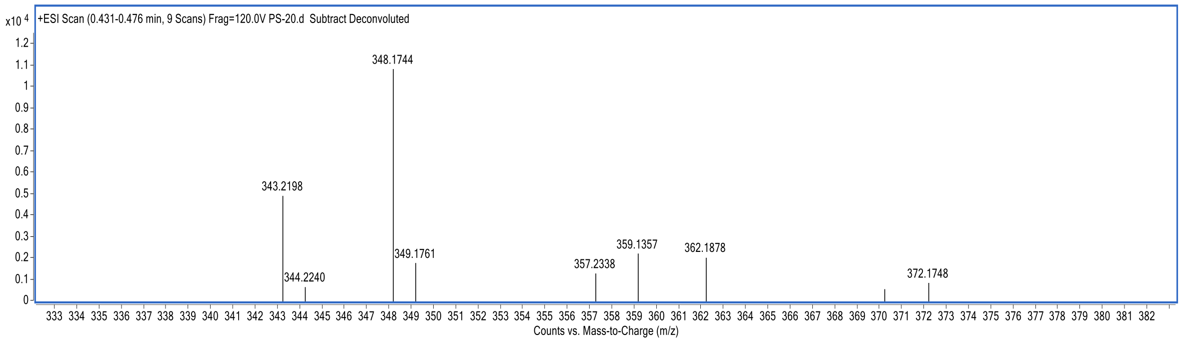

Figure S216. HRMS Spectrum of compound **7at**

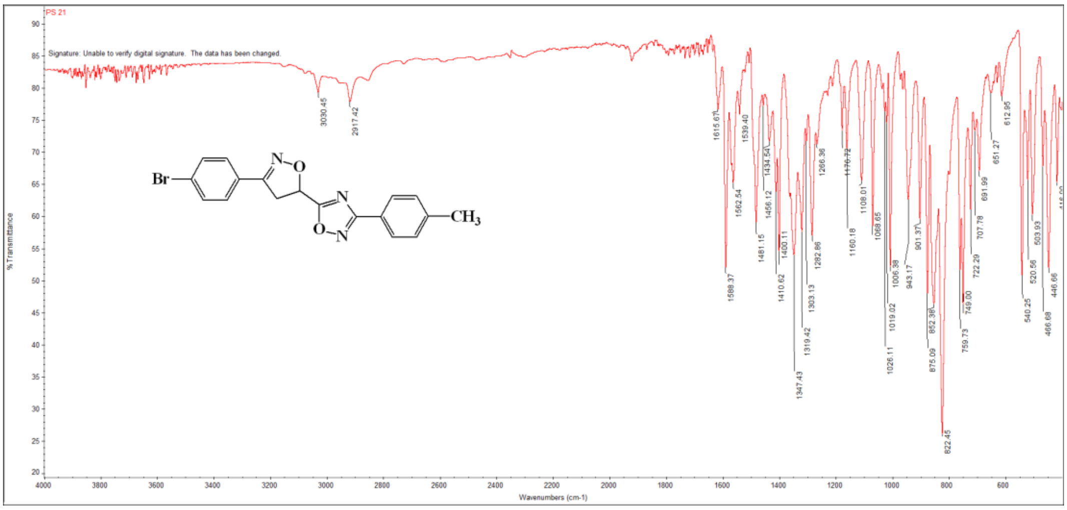

Figure S217. IR Spectrum of compound **7au**

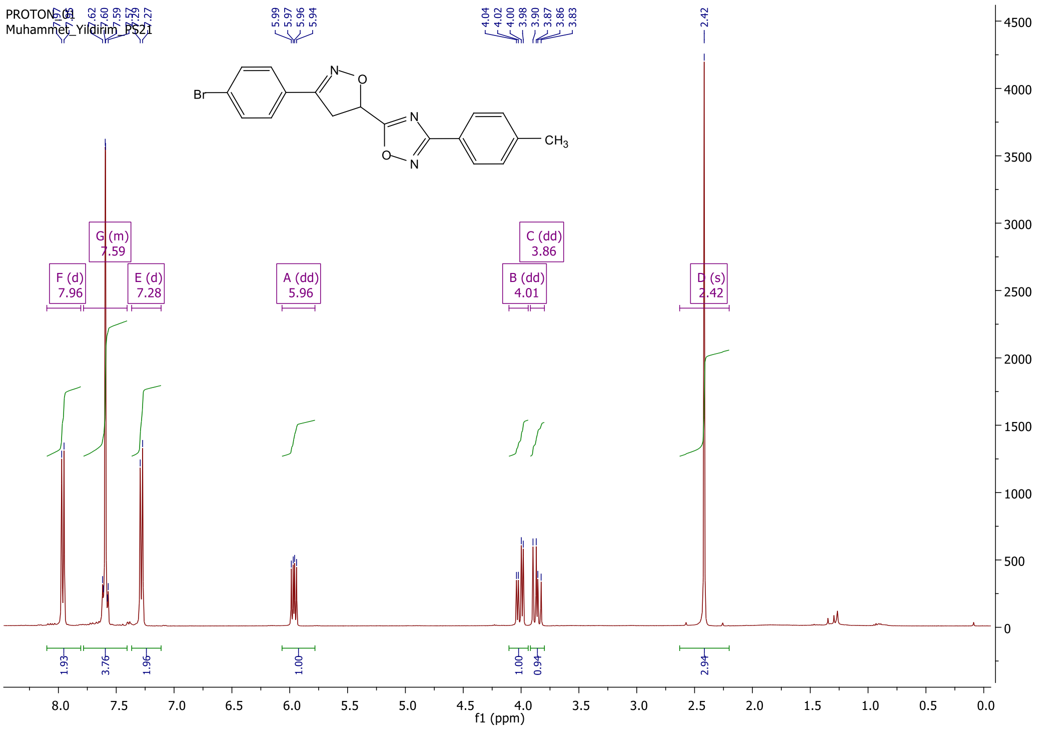

Figure S218. ^{1}H-NMR Spectrum of compound **7au**

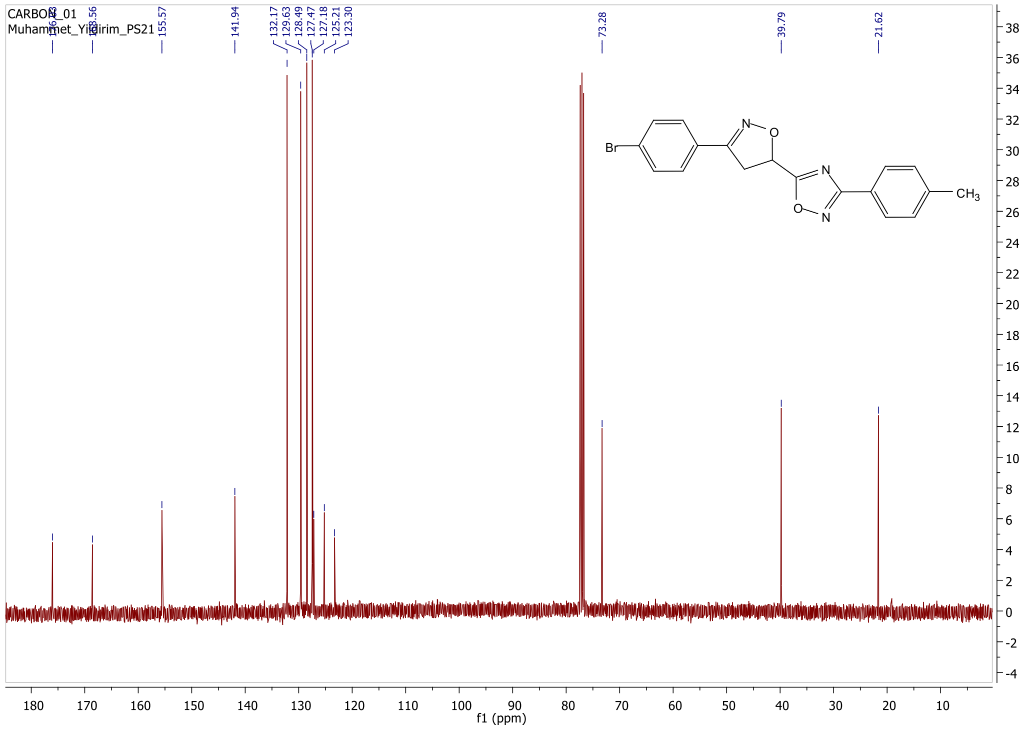

Figure S219. ^{13}C-NMR Spectrum of compound **7au**

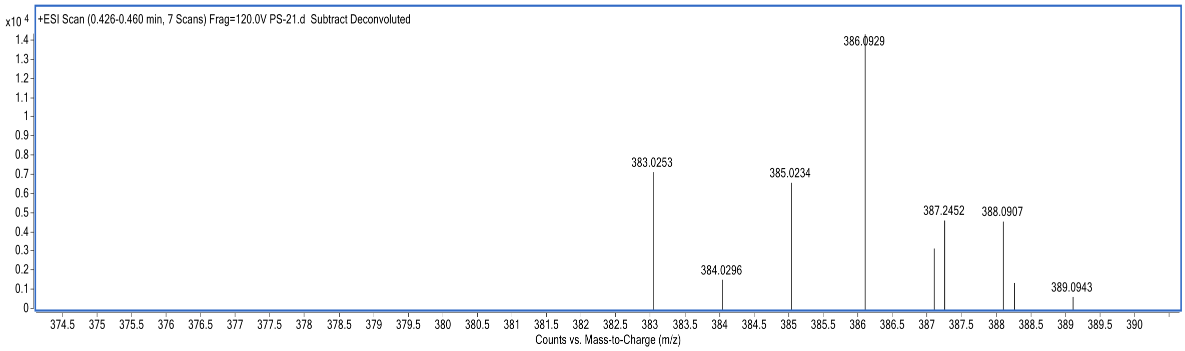

Figure S220. HRMS Spectrum of compound **7au**

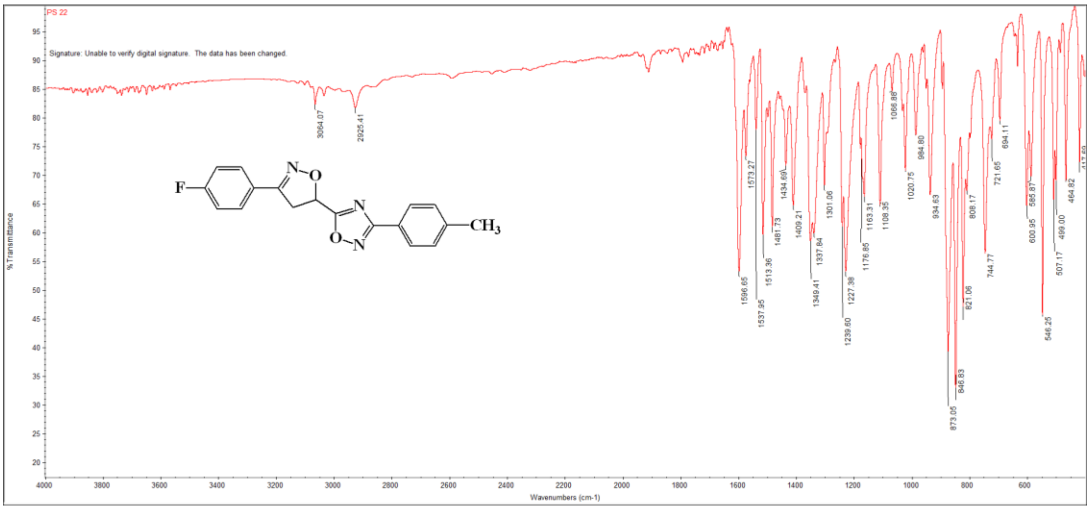

Figure S221. IR Spectrum of compound **7av**

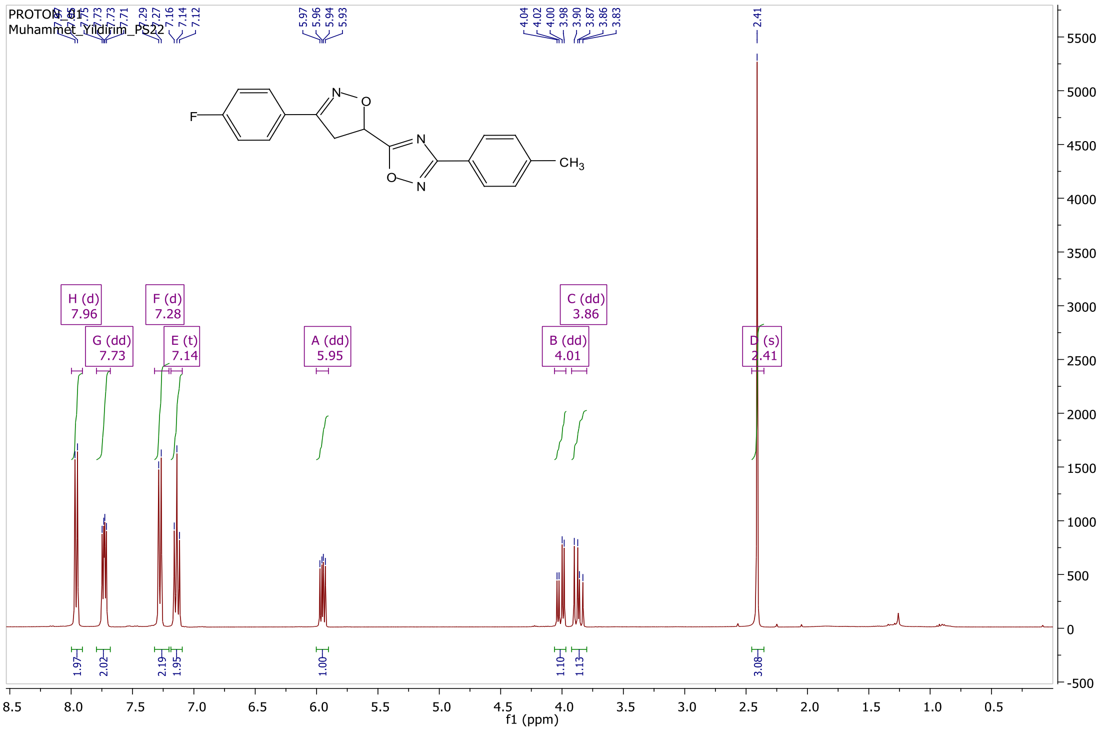

Figure S222. ^{1}H-NMR Spectrum of compound **7av**

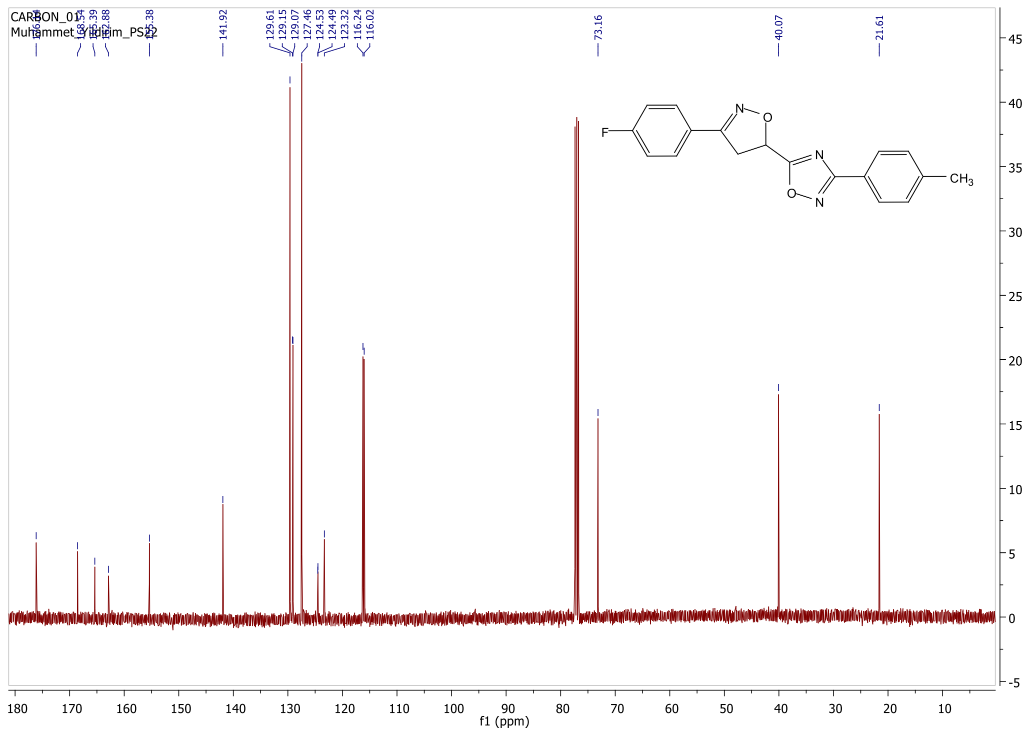

Figure S223. ^{13}C-NMR Spectrum of compound **7av**

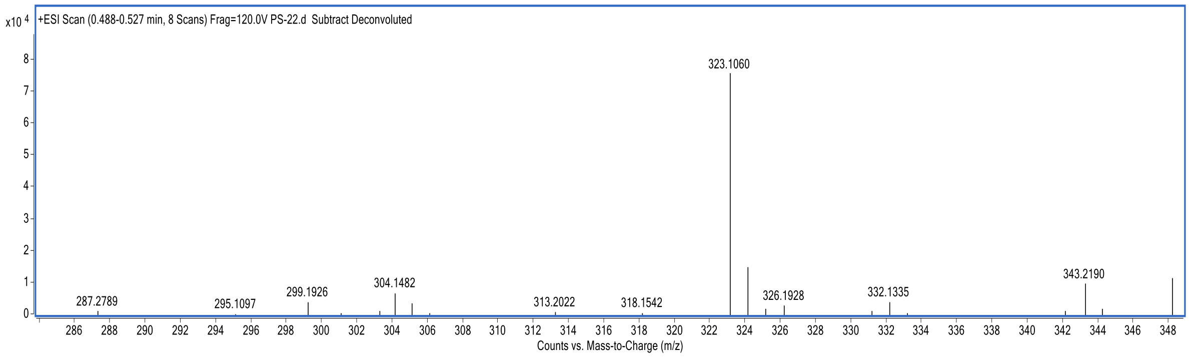

Figure S224. HRMS Spectrum of compound **7av**

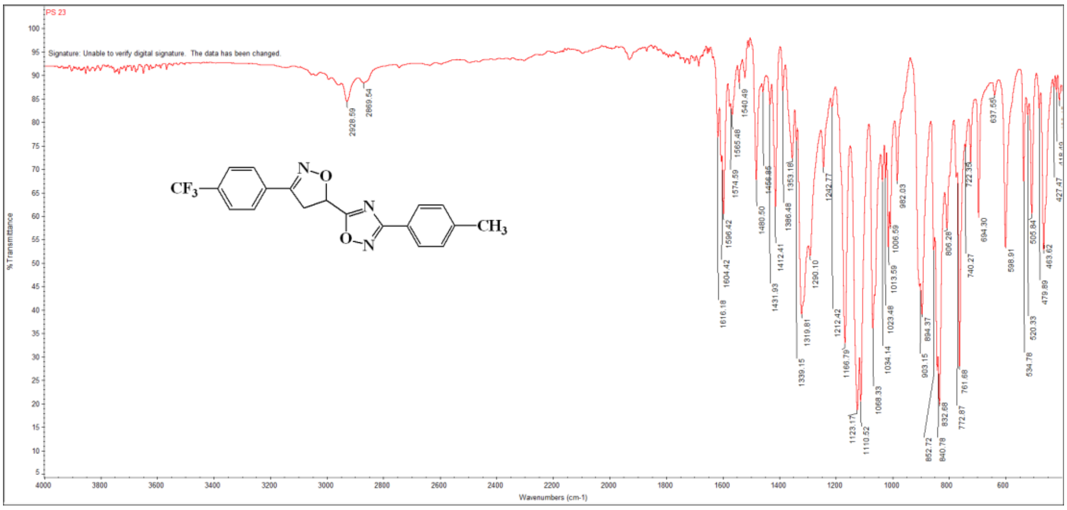

Figure S225. IR Spectrum of compound **7aw**

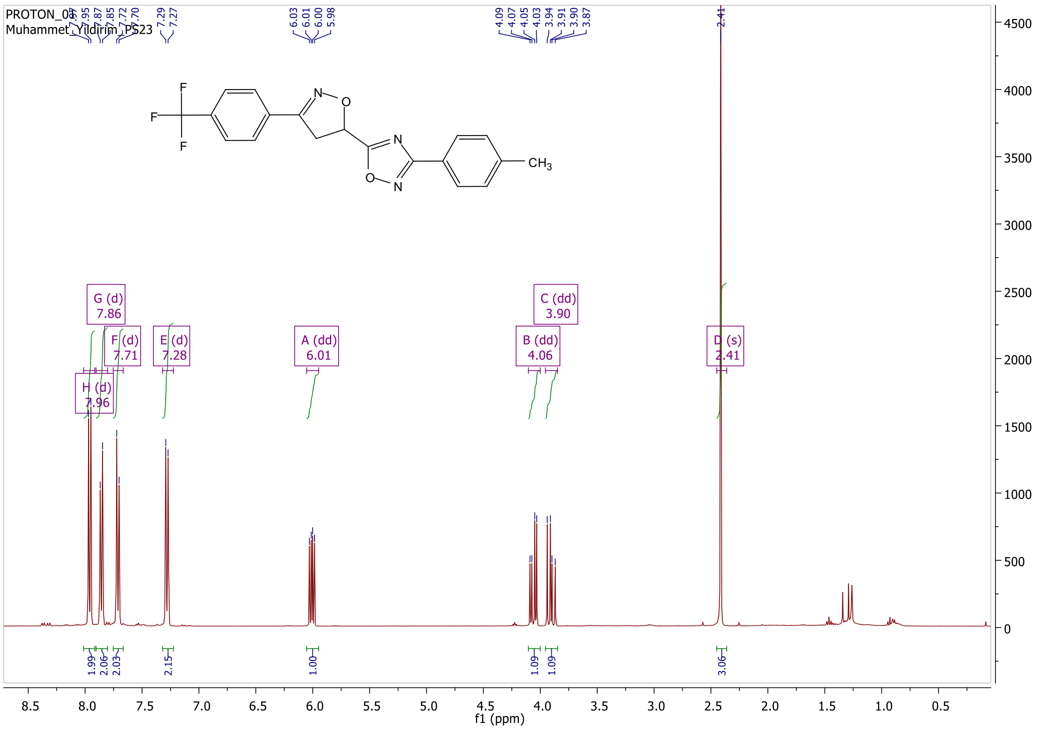

Figure S226. ^{1}H-NMR Spectrum of compound **7aw**

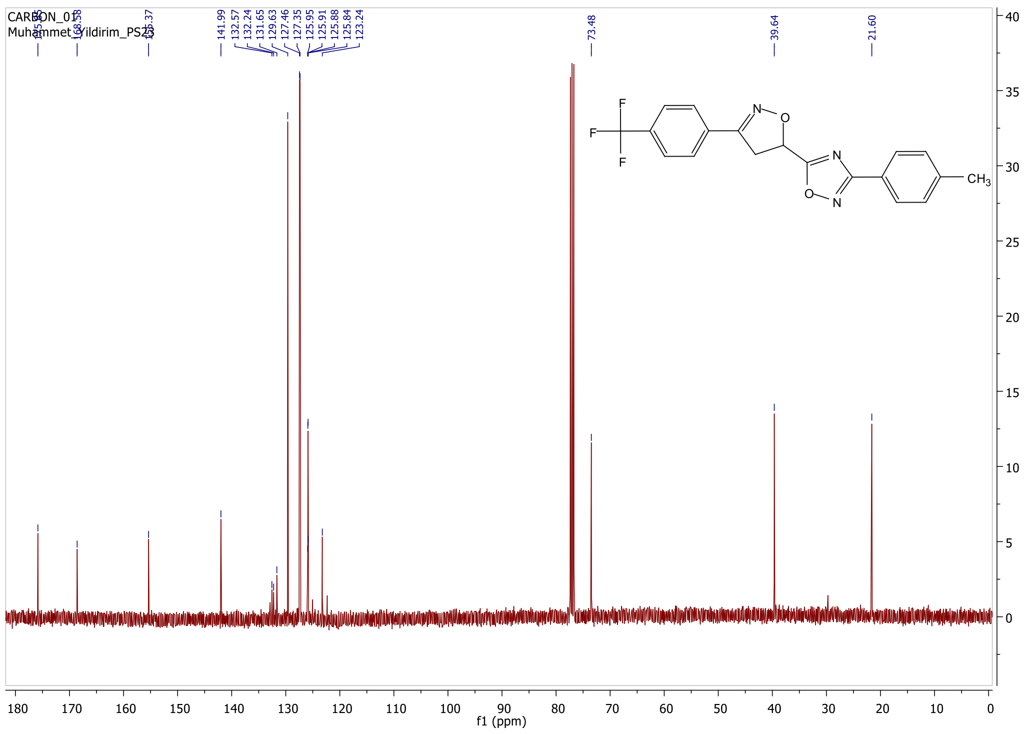

Figure S227. ^{13}C-NMR Spectrum of compound **7aw**

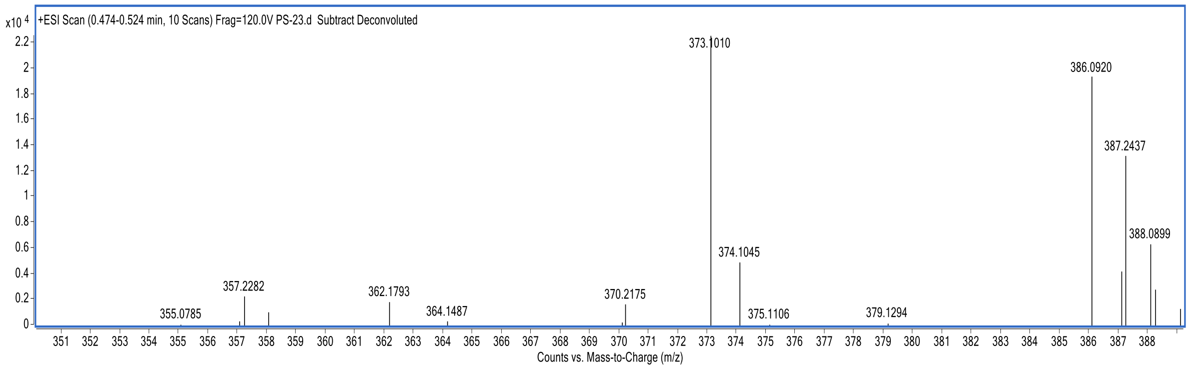

Figure S228. HRMS Spectrum of compound **7aw**

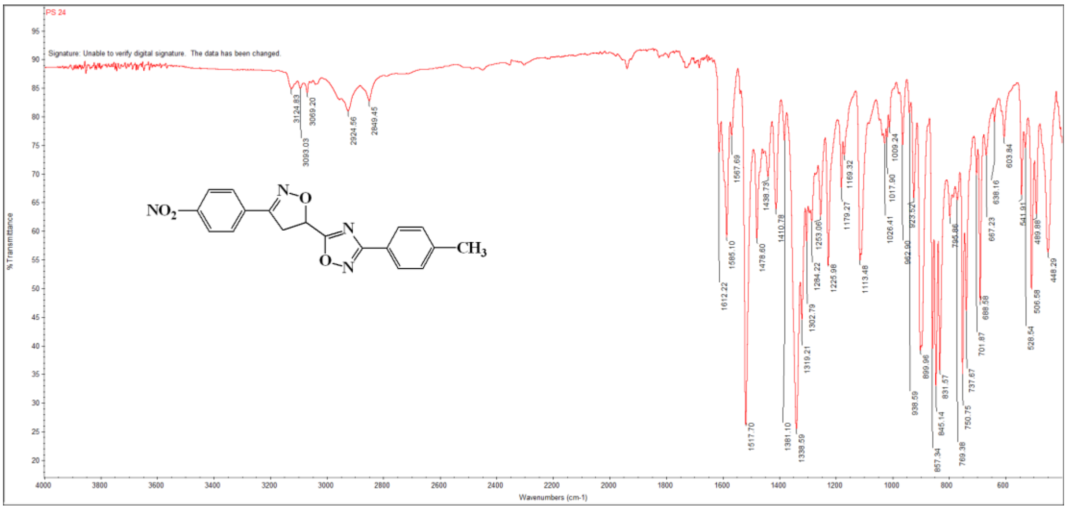

Figure S229. IR Spectrum of compound **7ax**

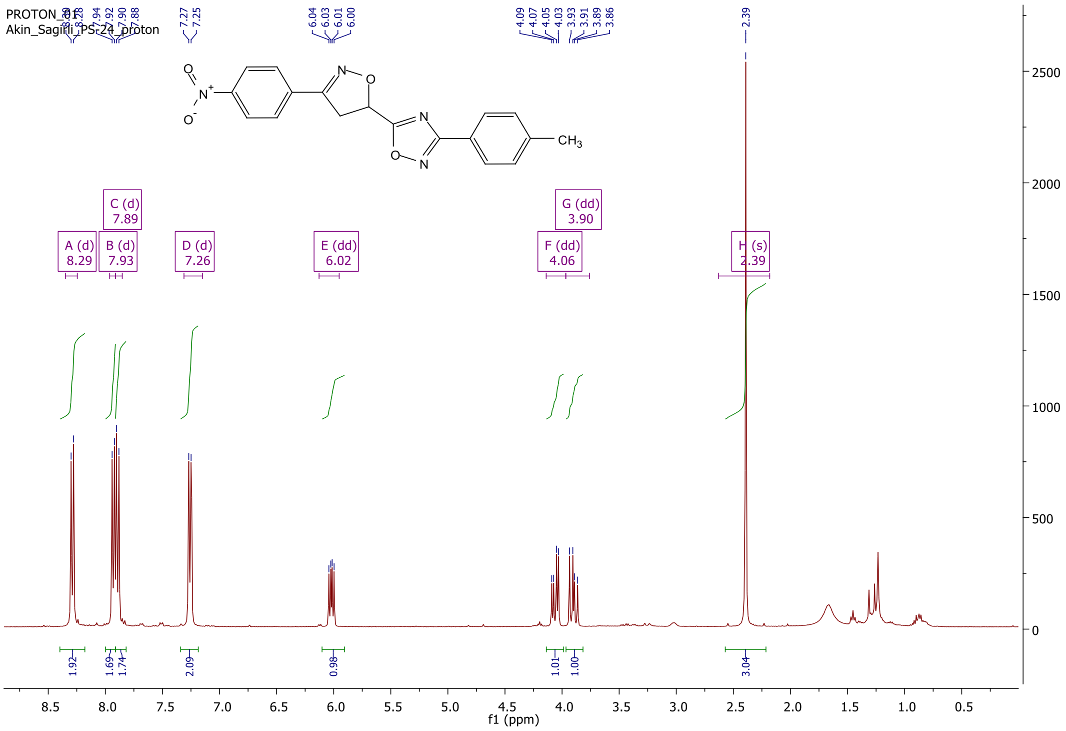

Figure S230. ^{1}H-NMR Spectrum of compound **7ax**

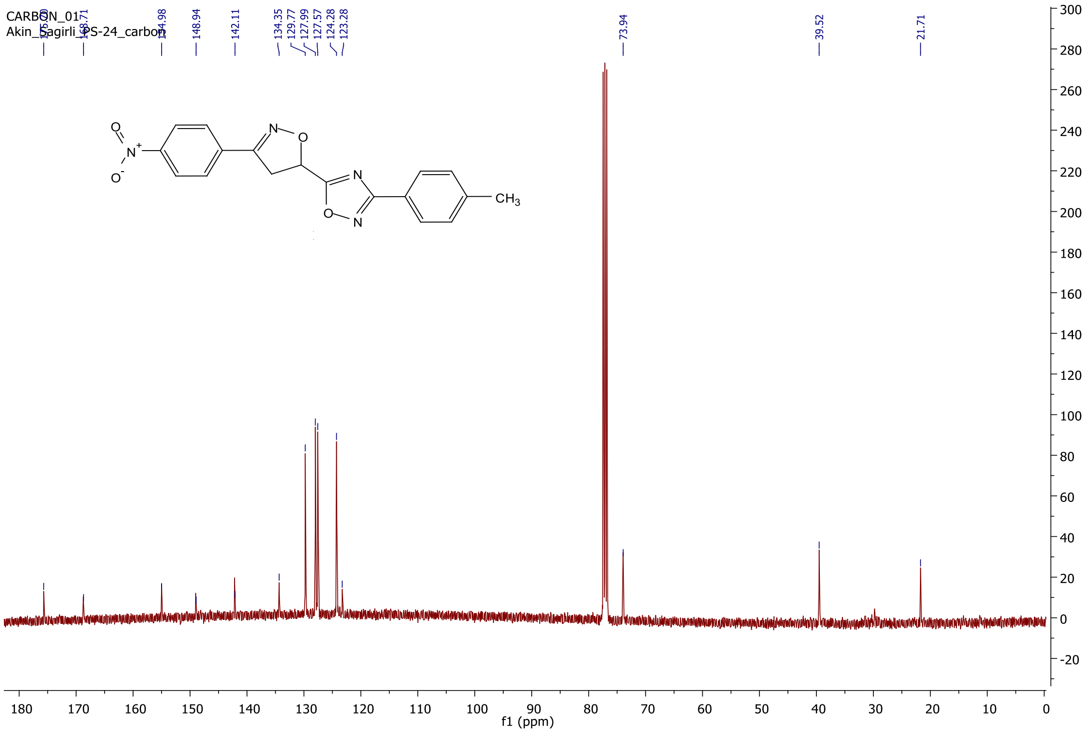

Figure S231. ^{13}C-NMR Spectrum of compound **7ax**

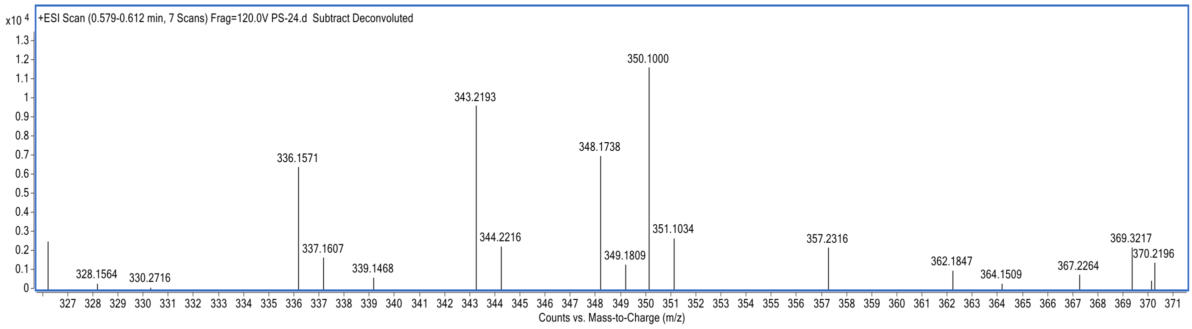

Figure S232. HRMS Spectrum of compound **7ax**

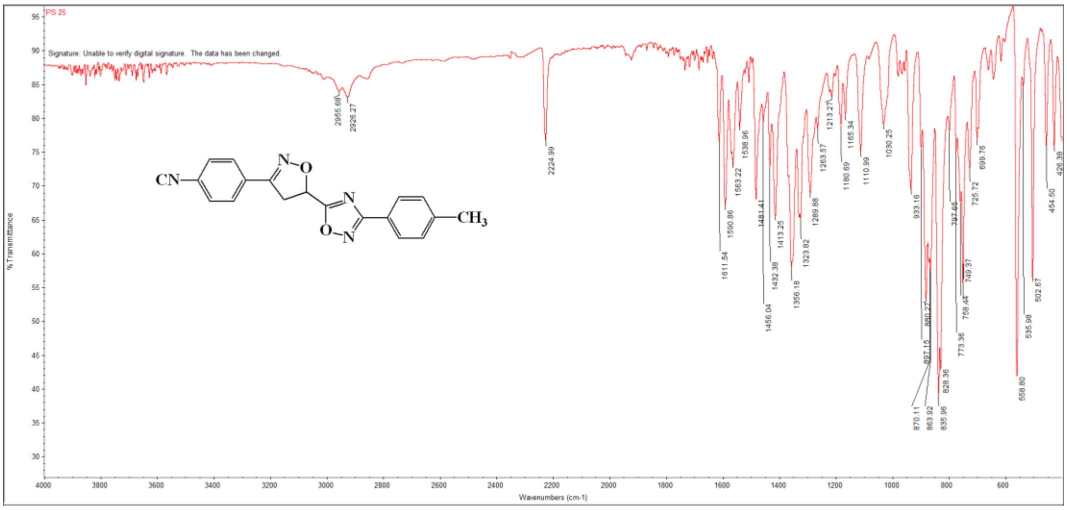

Figure S233. IR Spectrum of compound **7ay**

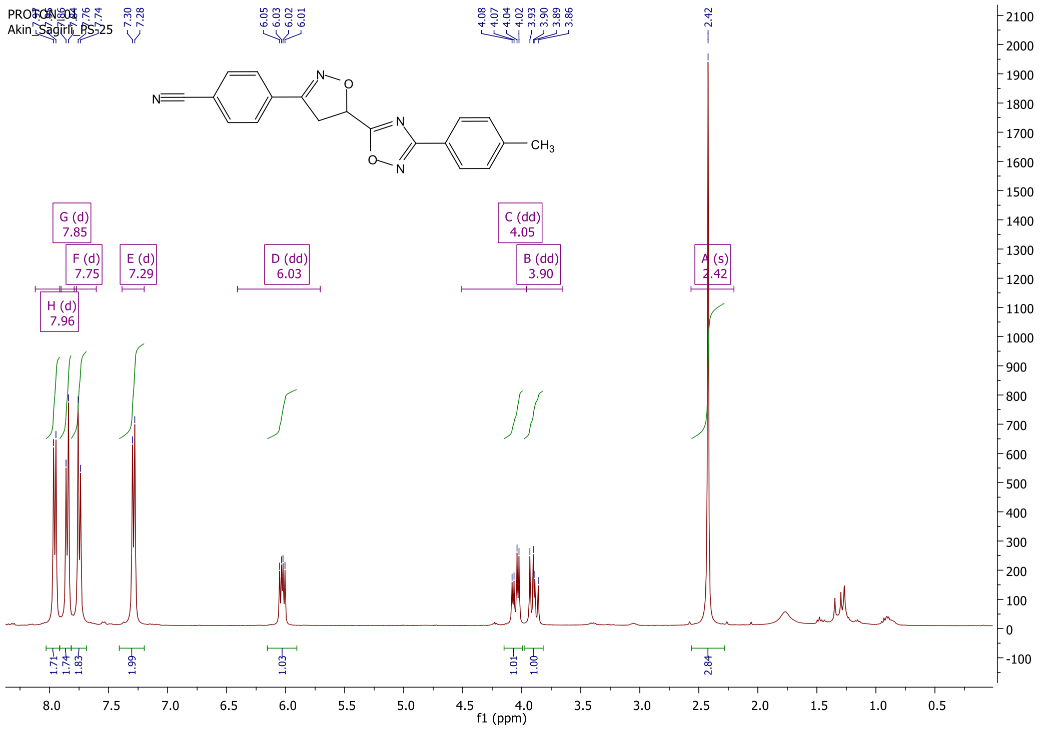

Figure S234. ^{1}H-NMR Spectrum of compound **7ay**

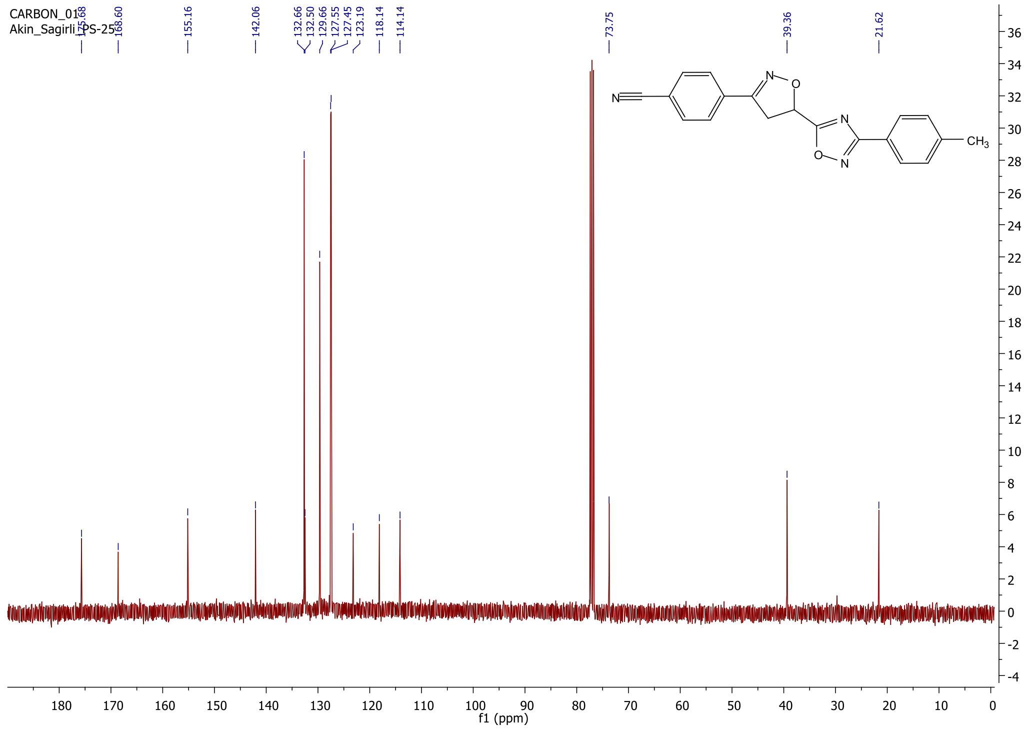

Figure S235. ^{13}C-NMR Spectrum of compound **7ay**

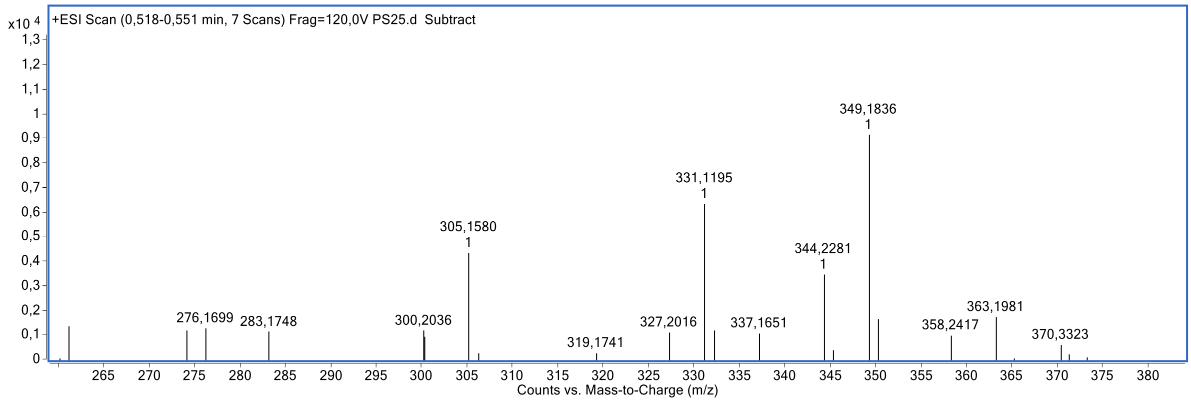

Figure S236. HRMS Spectrum of compound **7ay**
